# Sensing bacterial vibrations and early response to antibiotics with phase noise of a resonant crystal

**DOI:** 10.1038/s41598-017-12063-6

**Published:** 2017-09-22

**Authors:** Ward L. Johnson, Danielle Cook France, Nikki S. Rentz, William T. Cordell, Fred L. Walls

**Affiliations:** 1 Applied Chemicals and Materials Division, National Institute of Standards and Technology, Boulder, CO 80305 USA; 2 Chemical and Biological Engineering, University of Colorado, Boulder, CO 80309 USA

## Abstract

The speed of conventional antimicrobial susceptibility testing (AST) is intrinsically limited by observation of cell colony growth, which can extend over days and allow bacterial infections to advance before effective antibiotics are identified. This report presents an approach for rapidly sensing mechanical fluctuations of bacteria and the effects of antibiotics on these fluctuations. Bacteria are adhered to a quartz crystal resonator in an electronic bridge that is driven by a high-stability frequency source. Mechanical fluctuations of cells introduce time-dependent perturbations to the crystal boundary conditions and associated resonant frequency, which translate into phase noise measured at the output of the bridge. In experiments on nonmotile *E*. *coli* exposed to polymyxin B, cell-generated frequency noise dropped close to zero with the first spectra acquired 7 minutes after introduction of the antibiotic. In experiments on the same bacterial strain exposed to ampicillin, frequency noise began decreasing within 15 minutes of antibiotic introduction and proceeded to drop more rapidly with the onset of antibiotic-induced lysis. In conjunction with cell imaging and post-experiment counting of colony-forming units, these results provide evidence that cell death can be sensed through measurements of cell-generated frequency noise, potentially providing a basis for rapid AST.

## Introduction

Conventional antimicrobial-susceptibility testing (AST) methods are based on observation of growth of bacterial colonies in the presence of test antibiotics and typically take a couple of days or more to provide results. The times required to obtain AST reports can lead to ineffective treatment of infections, improperly and over-prescribed antibiotics, and emergence of antibiotic-resistant bacteria, all of which pose increasingly serious threats to public health. In the U.S., up to 50% of antibiotic prescriptions are nonoptimal or unnecessary, and at least 2 million illnesses and 23,000 deaths result from antibiotic-resistant bacterial infections each year^1^.

This work is motivated by an interest in enabling more rapid clinical AST through direct biophysical sensing. We present a technique for sensing mechanical fluctuations of microbes and demonstrate that it can be used to sense the response of nonmotile *Escherichia coli* (*E. coli*) to two antibiotics, polymyxin B and ampicillin, with different mechanisms of action. This technique takes advantage of the high sensitivity of quartz resonators to surface perturbations and extracts information on vibrational spectra of populations of adhered microbes through measurements of resonator phase noise.

Recently published studies provide evidence for motion of bacteria that decreases with antibiotic exposure. Longo *et al*.^2^, Aghayee *et al*.^3^, and Lissandrello *et al*.^4^ found that the presence of adhered motile *E. coli* on a microcantilever leads to fluctuating forces and associated displacements of the cantilever. Longo *et al*.^2^ also observed such effects with (nonmotile) *Staphylococcus aureus* (*S. aureus*). In both of these studies, the magnitude of cantilever fluctuations decreased when nonresistant bacterial cells were exposed to antibiotics. Longo *et al*. reported that measurable reductions in amplitude occurred within 5 min of the beginning of antibiotic exposure^2^. This led the authors to propose that measurements sensitive to mechanical fluctuations of cells can provide a basis for characterizing the antibiotic response of bacteria more rapidly than traditional growth-based techniques.

Detailed mechanisms responsible for cell-related fluctuations of microcantilevers have not been established. Lissandrello *et al*.^4^ suggested that various types of cell motion may simultaneously contribute to cantilever fluctuations, ranging from constrained flagellar motility to slow diffusion associated with reconfiguration of cell/substrate chemical bonding, and that this superposition may lead to the observed frequency dependence of power spectral densities of cantilever displacements. Longo *et al*.^2^ attributed their cantilever results to metabolically driven movement of cell walls, and this hypothesis is partly supported by the fact that antibiotic-susceptible fluctuations were observed with nonmotile cells (*S. aureus*)^2^. Evidence for a metabolic link was provided by the fact that fluctuations increased when glucose was added to the fluid environment of *E. coli* ^2^. Longo *et al*.^2^ also found that a metabolically driven process is energetically feasible: the power required to drive a microcantilever at the observed levels of displacement is orders of magnitude less than typical rates of energy consumption of *E. coli*.

Syal *et al*.^5^ employed a surface-plasmon scattering technique to measure vibration of individual motile *E. coli* tethered to a surface. They found that power spectral densities of cell vibration increased in glucose and substantially decreased in polymyxin B. These results provide strong evidence for metabolically-linked vibration of cell bodies, although the measured vibrations, in this case, were not determined to be independent of constrained flagellar motility. Direct measurements of cell-wall vibrations of individual nonmotile bacteria have not yet been reported.

The work presented in this paper is inspired by the previous research on sensing the effects of antibiotics on microbial fluctuations and pursues an entirely different measurement approach that employs MHz-range piezoelectric resonators. Cells are adhered to a disk resonator driven by a high-stability source near a crystal resonance, and cell-induced perturbations to the resonant frequency are extracted through measurements of the time-dependent phase of the crystal response. Relative to previous methods explored for sensing microbial fluctuations, MHz-range resonators offer potential advantages for commercial implementation in clinical environments, including cost-effective electronic data acquisition. By sacrificing single-cell information in favor of population-level information, resonant piezoelectric sensors can also be macroscopic and physically robust. For research purposes, the greater number of cells employed with macroscopic resonators is a better match for pairing with traditional plating techniques for assessing cell viability, which can facilitate validation and interpretation of results.

The general principle underlying our measurement approach is that time-dependent mechanical perturbations at the surface of any acoustic resonator induce fluctuations in resonant frequency. If a resonator is driven at a (noiseless) angular frequency ${\omega }_{0}$, the phase $\varphi$ of the mechanical response, relative to the excitation, is given by^6^

1 $$\varphi (t,{\omega }_{0})=\arctan [\frac{{\omega }_{0}{\omega }_{R}(t)/Q}{{\omega }_{R}{(t)}^{2}-{\omega }_{0}^{2}}],$$

where ${\omega }_{R}$ is the resonant angular frequency, $Q$ is the resonator quality factor, and dependence of ${\omega }_{R}$ and ${\rm{\Delta }}\varphi$ on time $t$ (if present) is explicitly indicated. The first-order derivative of $\varphi$ *vs*. ${\omega }_{0}$ leads to the following approximate expression for the shift in phase ${\rm{\Delta }}\varphi (t,{\omega }_{0})$ relative to $\varphi$ at resonance:

2 $${\rm{\Delta }}\varphi (t,{\omega }_{0})\approx 2Q\frac{{\omega }_{0}-{\omega }_{R}(t)}{{\omega }_{R}(t)}\approx 2Q\frac{{\omega }_{0}-{\omega }_{R}(t)}{{\omega }_{0}}.$$

This equation is valid for ${\rm{\Delta }}\varphi (t)\ll 1$ or, equivalently, ${\omega }_{0}-{\omega }_{R}(t)\ll {\omega }_{R}(t)/Q.$

In the specific case of a piezoelectric crystal resonator, an oscillating electric field can be employed for excitation, and the crystal response will involve coupled elastic strain and piezoelectric fields. If a driving voltage at ${\omega }_{0}$ is applied to an electrode on one surface of a piezoelectric disk, the response can be sensed through piezoelectrically induced voltages on an electrode on the opposite surface of the resonator. In this case, the frequency dependence of the relative phase of the sensed voltage differs from that given by Eq. 2 because of a superposition of resonator capacitance and sign reversal associated with piezoelectric polarization. However, after electrical compensation of resonator capacitance, phase shifts of voltages across the resonator are given by Eq. 2, apart from an overall sign change that is irrelevant to measurements of noise spectra presented here^7^.

## Experimental System

**Figure 1:**
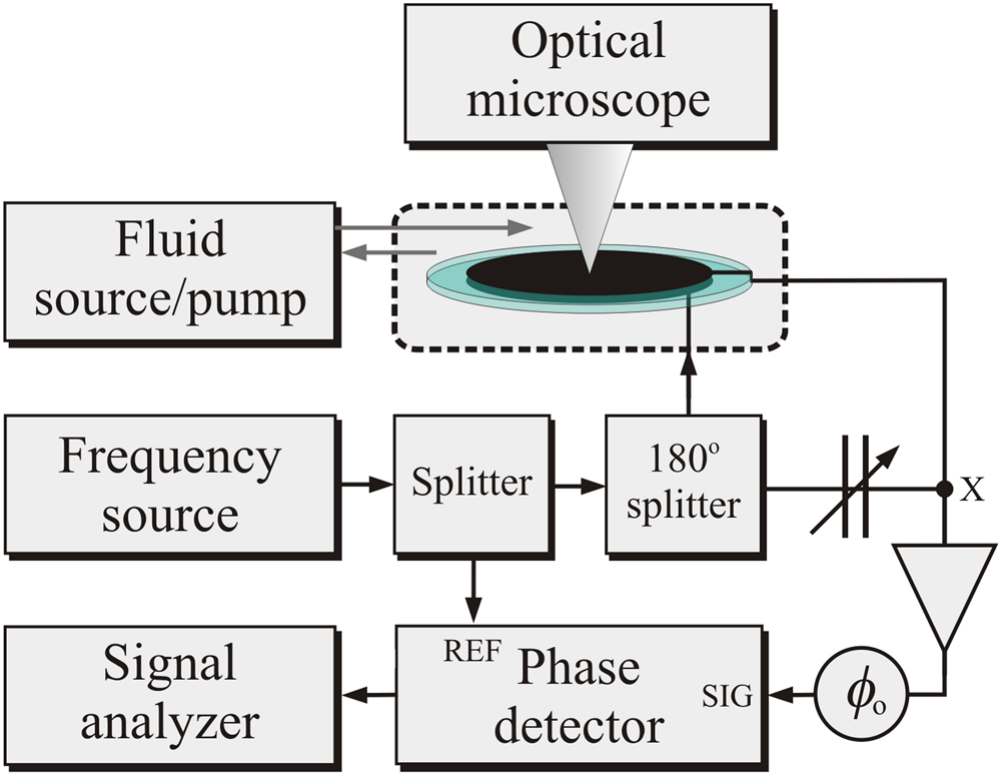
Block diagram of the measurement system.

The experimental system employed in this study drives one electrode of a quartz crystal resonator with a highly stable source near its fundamental resonant frequency ($\approx 5$ MHz) and extracts cell-induced perturbations to the resonant frequency through measurements of the phase of signals from the opposite electrode with an analogue phase detector referenced to the source. A block diagram of the experimental system for measurements on one resonator is shown in Fig. 1. The complete system includes two sets of such hardware, with the exception that only one set includes an optical microscope. The hardware for one resonator includes (1) a quartz-crystal resonator with thin-film gold electrodes on opposite surfaces; (2) a module (Biolin Scientific Q-Sense QWM-401) that supports the resonator, provides electrical contact to its electrodes, and incorporates channels through which fluids can be pumped to and from a chamber on one side of the resonator; (3) a passive electronic bridge with one arm incorporating the quartz-crystal resonator and a second arm incorporating a capacitor that balances the off-resonance capacitance of the resonator; (4) a high-stability tunable frequency source that drives the input of the electronic bridge; (5) a phase detector that converts the instantaneous phase difference between the bridge output and the driving source to a voltage; (6) a signal analyzer (Hewlett Packard HP 89410 A) comprised of a two channel fast Fourier Transform (FFT) spectrum analyzer that provides the power spectral density of the output voltage from the phase detector; and (7) an optical microscope that enables imaging of a central region of the resonator through a window in the resonator module. The two resonator modules are mounted on a custom-built temperature-controlled aluminum base, and all elements of the system, with the exceptions of elements (4) and (6), are configured on an optical table to provide isolation from external vibration. Details of electronic circuitry, resonators, signal analysis, and microscopy are presented in *Methods*.

The primary outputs from each experiment were a time series of power spectral densities (PSDs) of phase-detector output voltage ${S}_{V}$ and images of cells on resonator surfaces. One resonator was employed to test the effect of an antibiotic on adhered cells and another resonator was employed as a control with cells not exposed to the antibiotic.

## Results

*E. coli* was chosen as a model organism to demonstrate proof of concept of the sensing approach, and a nonmotile strain was used to eliminate potential contributions to the biophysical signal arising from cellular motility. Representatives from two major antibiotic classes were chosen for their differing modes of action. Polymyxin B (PMB), a cationic peptide with a long hydrophobic tail, acts to permeabilize the outer membrane of gram-negative bacteria. At high concentrations ($>$32 $\mu$g/mL), PMB can additionally depolarize the cytoplasmic membrane^8,9^, resulting in rapid cell death. Ampicillin acts quite differently, as a representative for the large group of broad spectrum beta-lactam antibiotics that share the mechanism of action of inhibiting synthesis of new peptidoglycans for the cell wall^10^.

All experiments were performed on *E. coli* HCB136 (with paralyzed flagella), which were graciously donated by the Howard Berg laboratory at Harvard University^11,12^. Lack of motility was verified by growth in 0.2% motility agar (Hardy Diagnostics, cat. no. Q11), with motile *E. coli* K12 (ATCC 23716) used as a positive control: HCB136 showed growth distinctly contained to the stab site, while K12 showed diffuse growth moving away from the stab site.

### Sensing response to polymyxin B

**Figure 2:**
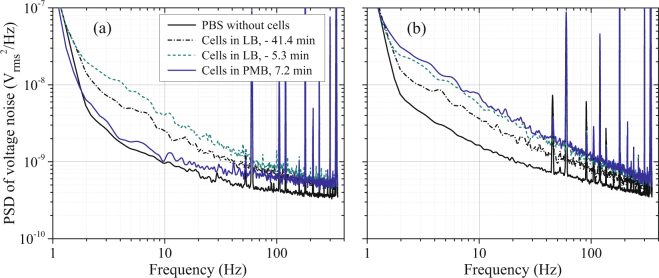
Power spectral densities of voltage noise ${S}_{V}(f)$ from the test resonator (**a**) and control resonator (**b**) in PBS (without cells), at two times with cells in LB before introduction of PMB, and 7.2 min after introduction of PMB to the test resonator. The zero for times listed in the legend is the beginning of PMB exposure.

Each experiment began with measurements of two resonators held at 37 °C in phosphate-buffered saline (PBS), providing background phase-noise spectra in the absence of cells. Figure 2 shows examples of PSDs of voltage fluctuations ${S}_{V}(f)$ from the phase detectors as a function of offset frequency $f$ (frequency of phase fluctuations) acquired during one experiment. The solid black traces in Fig. 2(a,b) show ${S}_{V}(f)$ from the test and control resonators, respectively, in PBS without cells. Each of these plotted spectra is an average of 200 individual spectra with a 1 Hz resolution bandwidth, acquired over a period of 2.5 min. These and all other spectra were acquired in the absence of fluid flow after a time delay that enabled recovery from fluid-induced changes in temperature.

The PBS background spectra in Fig. 2(a,b) are understood to be dominated above several Hz by noise contributions from the amplifier at the output of the bridge. Amplifier flicker noise, with a $\mathrm{1/}f$ dependence, is superimposed on frequency-independent white noise (Johnson noise)^7^. The $\mathrm{1/}f$ amplifier contribution is independent of acoustic properties of the resonator. However, the white noise contribution is inversely proportional to the power transmitted from the bridge to the amplifier, and this power is proportional to ${Q}^{2}$ of the resonator^6,7^. A stronger frequency dependence of the phase noise is observed below roughly 2 Hz, due to leakage in the 1 Hz FFT digital data filter of the signal analyzer. We restrict data analysis to 4 Hz and above to avoid any bias from this effect.

Each of the spectra has a number of sharp spurs above 45 Hz that arise primarily from pickup of line power at harmonics of 60 Hz and artifacts in the driving frequency source. A notable exception is the spur at 340 Hz which is intentionally introduced through modulation of the source and used for calibration and compensation for varying amplifier white-noise levels (*Methods: Electronics and signal analysis*).

After acquisition of background spectra, *E. coli* in PBS were introduced to the resonator modules. The modules were then drained of fluid to enhance cell adhesion, refilled with PBS, and flushed with Lysogeny broth (LB) to provide nutrients for the cells (*Methods: Cell and fluid handling*). During this sequence, cells adhered by interaction with a poly-L-lysine (PLL) layer that was deposited on each resonator before the experiment (*Methods: Resonators*), and unadhered cells were rinsed away. As shown in Fig. 2(a,b), the noise levels from both resonators increased when cells were introduced (curves labeled “Cells in LB, −41.4 min”) and increased further with time in LB as the cell populations grew (curves labeled “Cells in LB, −5.3 min”).

At time *t* = 0, polymyxin B (PMB, Sigma Aldrich) at a concentration of 200 $\mu$g/mL in LB was introduced to the test-resonator chamber, and LB without the antibiotic was simultaneously refreshed in the control-resonator chamber (*Methods: Cell and fluid handling*). At this antibiotic concentration, which is 100 times the minimum inhibitory concentration (MIC) reported for motile *E. coli* K12 (MG1655, wild-type)^13^, PMB permeabilizes the outer membrane of cells and depolarizes the cell membrane. Cells become slightly shorter and leak some intracellular contents, but their superstructure remains intact^8,9^. As shown in Fig. 2(a,b), ${S}_{V}(f)$ of the test resonator decreased to a level close to the background with the first spectrum acquired after PMB introduction (*t* = 7.2 min), while ${S}_{V}(f)$ of the unexposed control resonator continued to rise.

**Figure 3:**
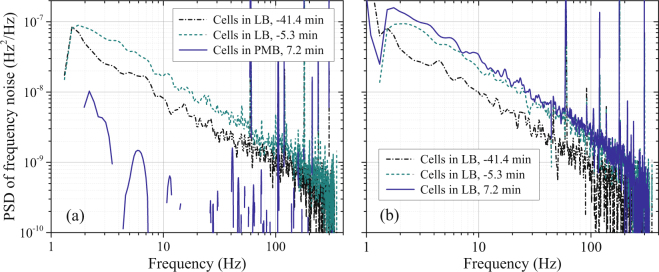
Power spectral densities of frequency noise ${S}_{{\rm{\Delta }}\nu }(f)$ of the test resonator (**a**) and control resonator (**b**) determined from the data on voltage fluctuations in Fig. 2 with compensation for variations in frequency sensitivity and white-noise levels and subtraction of the PBS background.

To extract cell-related contributions to the measured noise, the background spectra from the resonators in PBS (without cells) were subtracted. The height of the 340 Hz reference tone for each spectrum was also used to compensate for variations in the sensitivity of the system to resonant frequency noise and variations in the white noise level associated with changes in $Q$ during the experiment (*Methods: Electronics and signal analysis*). Figure 3 shows a conversion of the ${S}_{V}(f)$ data in Fig. 2 to PSDs of resonant frequency fluctuations ${S}_{{\rm{\Delta }}\nu }(f)$ associated with cells, where ${\rm{\Delta }}\nu \equiv ({\omega }_{R}-{\omega }_{0}\mathrm{)/2}\pi$ ^7^. In addition to the background subtraction, white-noise compensation, and compensation for time-varying sensitivity, this conversion involves an initial calibration of the sensitivity of the phase detectors to variations in resonant frequency (*Methods: Electronics and signal analysis*). The cell-related noise plotted in Fig. 3(a) for the test resonator shows an even more dramatic decrease with the introduction of PMB than the raw data of Fig. 2(a), primarily because of compensation for shifts in amplifier white noise that are apparent in the spectra of Fig. 2(a). Gaps in the plot of ${S}_{{\rm{\Delta }}\nu }(f)$ with cells in PMB (Fig. 3(a)) correspond to small negative values (after the background/white-noise subtraction).

**Figure 4:**
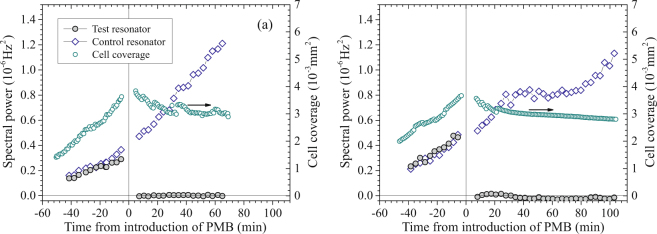
Results from two experiments on *E. coli* exposed to PMB: Spectral power of frequency noise (${S}_{{\rm{\Delta }}\nu }$) between 4 Hz and 20 Hz *vs*. time for the test and control resonators (*left axis*) and cell coverage area *vs*. time in the imaged region of the test resonator (*right axis*).

While the examples of spectra in Fig. 3 illustrate the changes in ${S}_{\Delta \nu }(f)$ when *E. coli* are exposed to growth medium and antibiotic, analysis of all spectra collected throughout the experiment (roughly every three minutes) gives a more complete picture of the time dependence. To provide a scalar measure of changes in ${S}_{\Delta \nu }(f)$ with time, spectra acquired before and after PMB exposure were integrated from 4 Hz to 20 Hz. Figure 4(a) shows this spectral power *vs*. experiment time $t$, referenced to the time of introduction of PMB. The spectral power of the frequency noise of both resonators increased with time before *t* = 0 as cells multiplied. For the test resonator, the absolute value of the first measurement of spectral power after PMB introduction (*t* = 7.2 min) was less than 1.6% of the last measurement before PMB introduction (a negative value, corresponding to the *Q*-compensated spectrum in PMB being slightly below the background). For the remainder of the experiment, the spectral power for this resonator remained less than 2% of the pre-exposure value. The spectral power of the control resonator continuously increased throughout the experiment.

Each of four additional experiments involving *E. coli* exposed to PMB showed a similar reduction in integrated spectral power to a few percent of the last values measured before antibiotic introduction. An example of these additional results is shown in Fig. 4(b). In this experiment, the absolute value of the first measurement of spectral power after PMB introduction (at *t* = 8.0 min) was less than 2.0% of the value before exposure to PMB, and values measured over the remaining $\approx 100$ min of the experiment were less than 6% of the pre-exposure value.

During each of these experiments, images of a 0.024 mm^2^ region close to the center of the test resonator were acquired at approximately one-minute intervals, not including the period when antibiotic was introduced. These images were used to calculate the cell coverage area in the imaged region through the use of the automated Otsu algorithm^14^, as implemented in the ImageJ software package^15,16^ (*Methods: Imaging*). Coverage area was chosen over direct counting as a measure of cell density because complete identification of individual cells was impractical in images with many clustered cells. A coverage area of $2.6\times {10}^{-3}$ mm^2^ (11% of the imaged region) corresponds to an areal cell density of (2.0 ± 0.3) × 10^4^ cells/mm^2^ on the resonator (where the uncertainty is determined from variations between experiments and spatial variations in coverage) or a total of approximately 2 million cells on the resonator (*Methods: Imaging*).

Values determined for coverage area of the imaged region are plotted *vs*. time in Fig. 4(a,b). These data reflect continuous growth and multiplication of cells in LB before antibiotic, modest changes in coverage during the gap in imaging when PMB was introduced (+5.9% and $-4.2$ % in Fig. 4(a,b), respectively), and gradually decreasing coverage after introduction of PMB. Abrupt steps in calculated coverage *vs*. time, most obvious after PMB introduction, arise from single-bit shifts in the digital grey-scale threshold determined by the automated Otsu algorithm.

**Figure 5:**
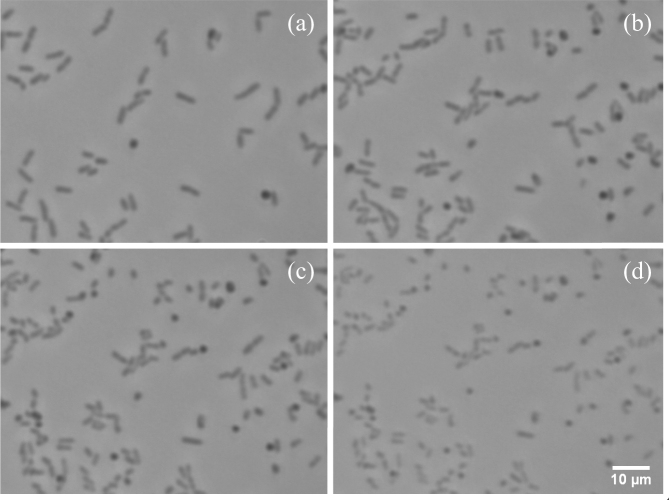
Images of an area near the center of the test resonator in the experiment of Fig. 4(a), chosen to represent key points in the cellular response: (**a**) cells growing actively in LB at $t$ = −33.3 min (relative to the time of introduction of PMB), (**b**) cells growing actively in LB a few minutes before the introduction of antibiotic ($t$ = −5.0 min), (**c**) cells a few minutes after the introduction of PMB ($t$ = 4.8 min), and (**d**) dead cells, visibly more faint after 69 min of exposure to PMB ($t$ = 69.0 min).

Figure 5 shows selected images acquired before and after introduction of PMB during the experiment of Fig. 4(a). To more clearly show the individual cells, these images include only one fourth of the full 0.024 mm^2^ imaged area that was used for calculating cell coverage. As illustrated in Fig. 5(c,d), the number of cells remained essentially constant after introduction of the antibiotic. However, cell images became more faint and decreased in size. The dimming of images during this period led the Otsu algorithm to sequentially reduce the grey-scale threshold used for coverage-area calculation, and the reductions in visible cell size led to the downward drift of the coverage area plotted in Fig. 4(a). The reductions in darkness and visible cell size may be associated with PMB-induced leakage of cellular proteins^8^.

The Supplemental Information includes a time-sequence video (*SI PMB* 1) of all the images from this experiment, along with real time videos captured just before and after the introduction of PMB (*SI PMB* 2 and *PMB* 3). These videos show visible growth, division, and tethered movement of cells in LB before the introduction of PMB and show no visible growth or movement after introduction of PMB.

After completion of phase noise measurements, each resonator was removed from its module and flushed with LB to suspend cells for serial dilution plating on LB agar. After overnight growth at 37 °C, the numbers of colony-forming units (CFUs) from the test resonator were compared to those from the control resonator to characterize killing efficiency of the antibiotic during the phase-noise experiment. PMB almost completely eradicated cells from the test crystal in each experiment. For the two experiments of Fig. 4(a,b), the CFUs on the test resonators were reduced by a factor of ≈10^7^ relative to the corresponding control resonator.

### Sensing response to ampicillin

Similar experiments were performed to study *E. coli* HCB136 exposed to 100 $\mu$g/mL ampicillin (Corning cellgro), an antibiotic with a mode of action very different from PMB. Ampicillin interferes with new cell wall synthesis. Affected cells initially continue to grow and elongate but are unable to complete cell division. Eventually the cell membranes bulge through gaps in the cell walls and cells rupture^10^.

**Figure 6:**
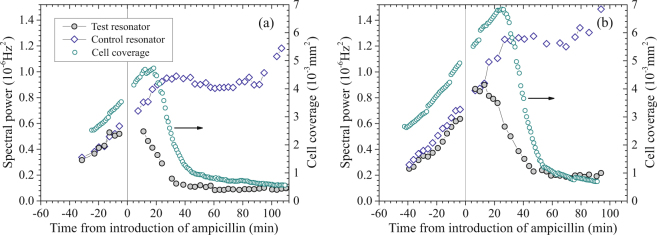
Results from two experiments on *E. coli* exposed to ampicillin: ${S}_{{\rm{\Delta }}{\rm{\nu }}}$ between 4 Hz and 20 Hz *vs*. time for the test and control resonators (*left axis*) and cell coverage area *vs*. time in the imaged region of the test resonator (*right axis*).

**Figure 7:**
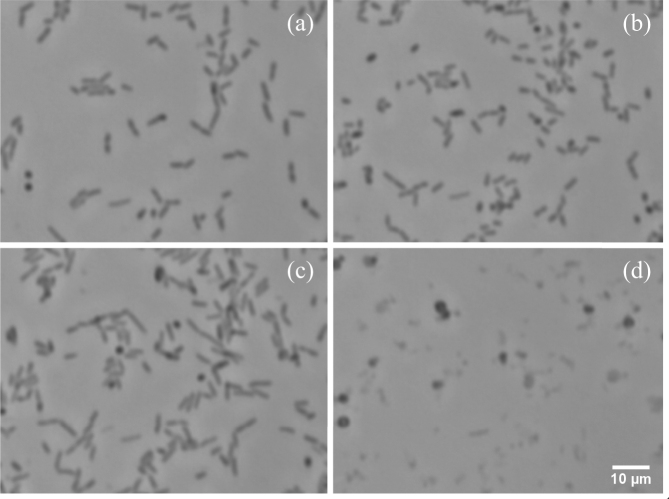
Images of an area near the center of the test resonator in the experiment of Fig. 6(a), chosen to represent key points in the cellular response: (**a**) cells growing actively in LB at $t$ = −24.7 min, (**b**) cells growing actively in LB a few minutes before the introduction of ampicillin ($t$ = −4.2 min), (**c**) elongated cells soon after the introduction of ampicillin ($t$ = 16.2 min), and (**d**) residual cells and debris after most cells have burst open in ampicillin ($t$ = 109.2 min).

Figure 6 shows the spectral power from 4 Hz to 20 Hz and cell coverage *vs*. time from two tests with ampicillin. As in the PMB tests, spectral power was strongly correlated with cell coverage over the period in LB (before antibiotic exposure), when cells were multiplying on the resonator surface. After ampicillin was added, the action of the antibiotic was clearly reflected in the measured cell coverage, which initially increased as individual cells visibly increased in length and then began dropping after 19–27 minutes as cells began rupturing. This sequence can also be seen in the images in Fig. 7, taken from the experiment corresponding to Fig. 6(a), and in a full time-lapse video (*SI Ampicillin* 1) from this experiment.

Interestingly, the spectral power in both experiments shown in Fig. 6 began decreasing even before decreases in cell coverage. The trend of the spectral power of each test resonator can be seen diverging from that of the corresponding control within $\approx$15 minutes of the beginning of antibiotic exposure, a point when antibiotic effects are clearly seen in the elongation of cells (Fig. 7(c)) but when cells are still capable of recovery upon removal of the antibiotic^10^. After an hour in ampicillin, lower spectral power of the test resonators was clearly correlated with lower numbers of intact cells.

While frequency noise of the test resonators decreased substantially in ampicillin, the recovery towards the baseline was less complete than with PMB and occurred over a longer period of time. The levels of spectral power 70 min after introduction of ampicillin in the two experiments of Fig. 6 were 17% to 21% of the maximum spectral powers of the test resonators. The longer period of the drop, relative to that with PMB, is consistent with the slower mode of action of ampicillin.

As illustrated in Fig. 7, images taken towards the end of the experiment (after the lysis of most cells) show approximately circular dark cells, which account for most of the residual calculated cell coverage. Although these dark spots are similar in appearance to spots on images taken before ampicillin exposure (e.g., Fig. 7(b)), the earlier spots are generally smaller and can be identified in the time-lapse video (*SI Ampicillin* 1) as cells that simply rotated to have their long axis perpendicular to the resonator surface and, in many cases, proceeded to rotate back to a parallel orientation. The persistence of the larger circular cells in ampicillin is much greater than the typical $\approx$3 minute lifetime of cell-wall bulges that form immediately before lysis^10^. This situation is suggestive of spheroplast formation. Spheroplasts are approximately spherical metabolically active cells that, unlike bulging *E. coli*, are unable to recover and generate new growth after ampicillin removal^10^.

The time-lapse video (*SI Ampicillin* 1) and real-time video taken at the end of the experiment (*SI Ampicillin* 4) show continuing micron-scale movement of residual cells and cell debris. This movement is consistent with there being significant residual frequency noise at the end of the experiment (Fig. 6(a)).

Serial dilution plating of cells at the end of the ampicillin experiments showed on the order of 500 CFUs remaining on exposed resonators, representing a reduction of at least five orders of magnitude relative to control resonators.

## Discussion

The power spectral densities of frequency noise (${S}_{{\rm{\Delta }}\nu }(f)$) of the resonators were found to be highly correlated with cell coverage during the periods when cell populations were growing in LB before introduction of an antibiotic (Figs 4 and 6). ${S}_{{\rm{\Delta }}\nu }(f)$ dropped after the introduction of PMB or ampicillin. The overall time scales of decreases in ${S}_{{\rm{\Delta }}\nu }(f)$ reflected those of antibiotic action, with PMB acting very rapidly and ampicillin taking longer to induce lysis of cells.

The correlation of ${S}_{{\rm{\Delta }}\nu }(f)$ with cell coverage in LB shows that the noise above the initial baseline is associated with the presence of cells. Time-dependent forces exerted by cells on a resonator are understood to perturb the boundary conditions of the resonator and lead to fluctuations of the resonant frequency. However, the observed correlations of frequency noise and cell density in LB do not, by themselves, demonstrate that cell-related frequency noise is linked with cell metabolism. In particular, we note that thermal energy (e.g., from motion of the surrounding fluid in the absence of pumping) can contribute to the motion of bacteria tethered to a surface. Even with the PBS background subtracted in the calculation of ${S}_{{\rm{\Delta }}\nu }(f)$, some fraction of the noise could conceivably arise from cell-mediated changes in the magnitude of forces transfered from thermal fluid motion to the surface of the resonator. However, the fact that the observed spectral power of frequency noise rapidly dropped by a factor of 50 or more after PMB exposure (Fig. 4), while cells initially remain mostly intact (Fig. 5(c)), provides evidence that changes in resonator surface perturbations from thermal fluid fluctuations, if they occur, are a relatively small contribution to ${S}_{{\rm{\Delta }}\nu }(f)$. Therefore, we conclude that the dominant contributions to the measured ${S}_{{\rm{\Delta }}\nu }(f)$ before PMB exposure are generated by active cells.

This conclusion is supported by evidence from previous studies. Single-cell measurements on motile *E. coli* by Syal *et al*.^5^ indicate that the magnitude of thermally driven movement of cells is much less than that of live cells before PMB exposure, and baseline measurements presented by Lissandrello *et al*.^4^ show that thermal contributions to power spectral densities of cantilever deflections in the absence of cells are at least an order of magnitude smaller than the contributions from motile *E. coli* below $\approx$6 Hz. Interpretation of these results is complicated by constrained flagellar motility being a source of cell-generated motion. However, Longo *et al*.^2^ also showed baseline cantilever deflections to be much less than deflections with live nonmotile *S. aureus* cells present.

The number of cells adhered to resonators in this study is several orders of magnitude greater than the number of cells on cantilevers in the studies of Longo *et al*.^2^, Aghayee *et al*.^3^ and Lissandrello *et al*.^4^. One might intuitively expect that surface perturbations from many cells with uncorrelated mechanical fluctuations would lead to a net cancellation of the effects on the resonant frequency, but this is not the case. As with other types of incoherent noise sources and waves, the PSD of resonant-frequency perturbations from cells will be additive, even though, on average, they linearly cancel one another^17^.

The ratios of spectral power to cell coverage varied from one experiment to another in the period before antibiotic exposure. For example, the last ratios measured before antibiotic introduction varied by a factor of $\approx 1.9$ between the experiments represented in Figs 4 and 6. The magnitude of these variations is not explained by our estimate of uncertainties in the ratio of the imaged coverage to the average cell density on the resonator (*Methods: Imaging*). We posit that the ratio of spectral power to imaged cell coverage is also affected by variations in the PLL adhesion layer, which affect the strength of transmission of cell vibrations to the resonators and, therefore, change the magnitude of cell-generated frequency perturbations for a given coverage of microbes.

Another distinctive feature that varied between experiments is a leveling off of spectral power of the control resonator *vs*. time (Figs 4(b) and 6(a,b)). This saturation could be associated with changes in cell configurations or morphology at higher densities, such as increased three-dimensional stacking of cells, with a potential dependence on the strength of PLL adhesion. Further exploration of the time dependence of spectral power in the absence of antibiotics, including the observed resumption of increases after leveling off, will likely require detailed microscopic studies of cell growth patterns.

Cell-generated ${S}_{{\rm{\Delta }}\nu }(f)$ is shown in Fig. 3 to be approximately proportional to $\mathrm{1/}f$ over the measured frequency range. In studies of motile *E. coli* with cantilever and surface-plasmon scattering techniques^4,5^, measured noise was also found to increase at lower values of $f$. Analysis of the dependence on $f$ will likely be important for understanding biophysical mechanisms of cell-generated noise, but an exploration of this subject is beyond the scope of the present paper.

The results obtained for the time dependence of integrated spectral power and cell coverage with *E. coli* exposed to PMB show the cell-generated frequency noise dropping close to zero as soon as the first phase-noise measurement was completed after antibiotic introduction (within 7 to 8 minutes in Fig. 4(a,b)). At this point in each experiment, the cell coverage was within 6% of the value measured before antibiotic exposure. Therefore, the dramatic change in spectral power reflects a change in cell-generated perturbations to the resonator unrelated to changes in cell number.

As illustrated in Fig. 6, the situation with cells exposed to ampicillin is more complex. The cell-related spectral power of each of the test resonators began dropping within 15 minutes of the beginning of antibiotic exposure, while that of the corresponding control resonator continued to rise. At this point in the experiments, the cell coverage on the test resonators continued to increase as cells elongated, with division inhibited by the antibiotic. Therefore, the phase-noise measurements detected an antibiotic-induced change in fluctuations of the cells. Once cells began lysing, as reflected in decreasing cell coverage, ${S}_{{\rm{\Delta }}\nu }(f)$ dropped more rapidly and, then, began leveling off towards the end of each experiment (Fig. 6).

The visual appearance and persistence of approximately circular cells at the end of the ampicillin experiments has led to the tentative conclusion that these cells are spheroplasts^10^. This conclusion is consistent with the plating results showing five orders of magnitude reduction in CFUs on the test resonators relative to the control resonators, since spheroplasts are unable to generate colonies^10^. The residual frequency noise at the end of the experiments is partly attributed to the movement of these circular cells, which is visually apparent in images acquired at the ends of the experiments. The level of this residual noise, relative to that of the control, is approximately three orders of magnitude greater than that expected from viable cells at the end of the experiments, based on the plating results. Observed movement of cellular debris in the images also may have contributed to frequency noise at the end of the ampicillin experiments. Thermally excited motion of these particles could be a more significant contribution to spectral power than thermal excitation of larger intact cells, but any conclusions about this require further study.

The imaging and plating results from the experiments with both antibiotics show that decreases in frequency noise are linked with cell death. A link with cell viability is shown in the ampicillin experiments to be less straightforward, because residual noise at the end of the experiments is greater than that expected from the remaining viable cells. Under the hypothesis that the residual noise primarily arises from spheroplasts, the lack of time dependence of the noise towards the end of the experiments is a signature of the lack of growth and associated nonviability of most cells that remain after the lysis stage of the population is essentially complete.

## Conclusions

The results of this study support the conclusion that phase-noise measurements on resonant piezoelectric substrates can be used to sense antibiotic-induced changes in mechanical fluctuations of cells on a time scale orders of magnitude shorter than times typically required for traditional growth-based AST.

The use of PMB as one of the test antibiotics has substantially strengthened conclusions. Since the action of PMB leaves the cellular superstructure essentially intact after exposure, the low level of frequency noise (after background subtraction) in PMB indicates that changes in thermal contributions to resonator noise are much less than noise generated by the *E. coli*. The use of a bacterial strain with paralyzed flagella has also enabled stronger conclusions about the source of phase noise than if experiments had been performed on a motile strain. Despite the absence of constrained flagellar motility, cell-generated frequency noise was found to be well above background noise levels. This result provides evidence that the technique can detect resonator perturbations from mechanical fluctuations of cell walls (not necessarily generated within the walls) in nonmotile bacteria.

The measurement approach presented here offers the potential of broad applicability to sensing efficacy of antibiotics through their effects on mechanical cellular activity. The experiments have focused on only one nonmotile gram-negative bacterial species. However, the perceived potential of the measurement approach is enhanced when considered in conjunction with results from other labs that demonstrated antibiotic-induced reductions in cellular fluctuations in motile gram-negative (*E. coli*)^2–5^ and nonmotile gram-positive (*S. aureus*)^2^ species. The results presented here also have included only two antibiotics, but these antibiotics have different mechanisms of action (membrane disruption and beta-lactam cell-wall inhibition) and represent a significant fraction of available drug types. Tests with representatives from two remaining major drug classes, DNA/RNA polymerase inhibitors and ribosomal inhibitors, would round out the variety of detectable killing mechanisms studied. Therefore, the situation at the end of this study is that an assessment of the breadth of applicability of the sensing approach will require tests of additional representative bacterial species exposed to antibiotics with several representative modes of action.

## Methods

### Electronics and signal analysis

The basic elements of the passive bridge for each resonator are depicted in Fig. 1. A 180° splitter at the input of the bridge leads to approximately opposite signs of oscillating voltages at the bridge output from the balancing capacitor and the electrical capacitive part of the crystal resonator. Prior to an experiment, the capacitor is tuned to provide minimal transmission through the bridge with the driving frequency far enough from the mechanical resonance that the resonance has no significant effect (relative to the background noise level) on the transmitted power from the bridge.

The signal from the bridge is passed through an amplifier to an analogue phase detector (Femtosecond Systems model FSS 1000E). As shown in Fig. 1, a constant phase ${\varphi }_{0}$ is introduced by a digitally tunable delay line (Femtosecond Systems Model FSS 1011 A) before the input to the phase detector to bring the phase difference between the signal and the reference close to the center of a phase sensitive quadrant.

The phase stability of the driving (reference) frequency source must be exceptionally high to enable sensing of cell-induced fluctuations of the bridge output. The system employs a pair of signal generators (Hewlett Packard model HP 8663 A; one for each resonator circuit) that have excellent overall phase stability but insufficiently low phase-noise background in the single-Hertz range. To further reduce background noise levels, the signal generators were operated at frequencies near 80 MHz and reduced to the fundamental crystal-resonator frequency of $\approx$5 MHz through the use of low noise frequency dividers. The resultant reduction in PSD of phase noise of the source was approximately ${\mathrm{(80/5)}}^{2}$, or 24 dB.

At the beginning of each experiment, the frequency of each source was set to the peak of its respective resonator by modulating the frequency at 2 kHz and tuning the center frequency to minimize amplitude modulation of the phase detector output. The 2 kHz modulation was then terminated and ${\varphi }_{0}$ was adjusted with the delay line to provide zero DC output voltage from the phase detector. The delay line was then left unchanged throughout the remainder of the experiment.

As an experiment proceeds, the frequency of each resonator slowly changes due to interaction with the cells, and this leads to drifting DC output from the respective phase detector. The frequency of each source was adjusted immediately before acquisition of each set of averaged spectra to bring the DC output back to the center of the dynamic range of the phase detector.

The raw spectral data from the signal analyzer are in units of PSD of voltage noise ${S}_{V}(f)$. These data are converted to corresponding ${S}_{{\rm{\Delta }}\nu }(f)$ through the use of a measured sensitivity factor. The frequency-to-voltage sensitivity at the beginning of each experiment with the resonator in PBS (without cells) was determined by stepping the source frequency by ±10 Hz and measuring the resultant change in DC voltage from the phase detector. As an experiment proceeds, the $Q$ of a resonator varies, due to changes in damping by the cells as they grow or respond to an antibiotic. This affects the sensitivity of phase-noise measurements (Eq. 2) and the electronic white-noise background. To compensate for such variation during subsequent data analysis, a constant-amplitude 340 Hz tone was used to continuously frequency modulate (FM) the high-stability source. The action of the resonator is to demodulate this FM signal to produce a signal at 340 Hz that is proportional to ${Q}^{2}$ (Eq. 2). Therefore, the height of this 340 Hz peak in the acquired ${S}_{V}(f)$ can be used to track changes in sensitivity and corresponding${Q}^{2}$ throughout an experiment.

The contribution of the white noise level of the bridge amplifier to the detected signal ${S}_{V}(f)$ is inversely proportional to the detected power level at the input of the amplifier (at point X in Fig. 1). Since this power level is proportional to ${Q}^{2}$, the height of the 340 Hz signal can be used to calculate changes in the white noise contribution of the amplifier relative to that measured before the introduction of the cells. This additional electronic noise is subtracted from every data point in ${S}_{V}(f)$ during the conversion from ${S}_{V}(f)$ to ${S}_{\Delta \nu }(f)$.

### Resonators

The quartz-crystal resonators in this study (manufactured by Biolin Scientific) had AT-cut crystal orientation, fundamental thickness-shear resonances near 5 MHz, a diameter of 14 mm, and noncontoured (plano-plano) surfaces. Circular gold electrodes on the crystal surfaces had diameters of 12 mm and 5 mm. In addition, gold films extended from these circular electrodes to regions near the edges of one side of each crystal (wrapping around the crystal edge from the larger electrode) to enable electrical contact to both electrodes from one side of the crystal.

Resonators were cleaned before experiments in sequential five-minute washes of 2% sodium dodecyl sulfate (SDS), acetone, methanol, and isopropyl alcohol with light agitation. The resonators were then dried in air and exposed to a UV/ozone treatment for 30 minutes. The effectiveness of this cleaning protocol was confirmed through optical tests that showed near-zero contact angles of water droplets (i.e., complete spreading of a droplet over the surface of a resonator).

A 200 $\mu$L droplet of 0.01% poly-L-lysine (PLL, molecular weight 15 k to 30 k) in water was applied to the center of the larger-diameter gold resonator electrode and allowed to sit for five minutes. Excess fluid was then wicked away at the edge of the resonator, and the remaining fluid was allowed to dry in air. Resonators coated with PLL were stored in ambient air until used in an experiment within 24 hours.

Each resonator was mounted for experiments in a module (Biolin Scientific model QWM 401) with concentric O-rings that provided mechanical support and enabled fluids and cells to be confined to one side of the resonator (the side with the larger-diameter electrode) within a 0.10 mL chamber. The inner diameter of the O-ring on the fluid-covered surface was 11.6 mm.

### Cell and fluid handling

Before experiments, cells were grown overnight at 37 °C in agitated Lysogeny Broth (LB) (10 grams tryptone, 5 grams yeast extract, and 10 grams of NaCl per liter, pH 7.4). A few hours before the experiment, the culture was diluted by a factor of 500 and allowed to grow to an optical density at 600 nm (OD_600*nm*_) of approximately 0.05. Cells were collected on a cellulose acetate filter with a nominal maximum pore size of 0.22 $\mu$m, then backflushed with a smaller volume of phosphate-buffered saline (PBS; pH 7.4). The final suspension used for testing was in 4 mL PBS with OD_600*nm*_ = 0.36 ± 0.02.

All PBS and LB used for cell processing and filling of modules during experiments were passed through 0.22 $\mu$m filters to remove any particles. A few minutes before each experiment, PBS for filling modules was degassed in a flask that was evacuated by a scroll pump to a pressure less than 1300 Pa (10 torr). A peristaltic pump and tygon tubing were used to pass fluids and cells in fluid through the channels in each module that led to and from the chamber above the resonators. In Table 1, flow rates and durations are listed in the order of the steps employed in each experiment. Steps involving filling with PBS and draining of cells in PBS were performed only long enough to completely fill or drain modules and were not timed. The time from start of fluid pumping to complete filling of modules was ($1.9\pm 0.1$) min.

**Table 1:** Rates and durations of flow during the steps of fluid exchange in experiments, listed in sequential order.

Experimental step	Rate (mL/min)	Duration (min)
PBS	0.4	
PBS with cells	0.2	7.5
PBS/cell draining	0.1	
PBS refill	0.1	
LB	0.1	6.0
LB with/without antibiotic	0.1	6.0

### Imaging

An upright optical microscope with a 50x/0.55 objective was used to image a 178 $\mu$m $\times$ 133 $\mu$m (0.0237 mm^2^) area on the surface of the antibiotic-exposed resonator with polarized white light that was reflected from the surface through a sapphire window at the top of the fluid chamber of the module. Image acquisition software linked with a 1392 × 1040 pixel 12-bit camera was used to acquire images at one-minute intervals throughout each experiment, which were saved in 8-bit gray-scale format. The corresponding resolution of the images was 7.8 pixels/$\mu$m.

Collected images were subsequently analyzed with ImageJ software^15,16^. The gray-scale levels of the images were inverted, and a background image taken before the introduction of cells was subtracted from each of the subsequent images. The automated Otsu algorithm^14^ was employed to select a gray-scale threshold for each image to distinguish cells from background. This threshold was used as a cut-off for converting each image to a binary format, where all white pixels represented cells and all dark pixels represented background. These binary images were then analyzed to determine cell coverage area within each image. Cell coverage was considered to be a generally more comprehensive and reliable metric of the biomass on a resonator throughout an experiment than direct cell counting, because greater numbers of closely spaced cells after growth in LB made reliable visual identification of individual cells impractical. In addition, cell count would not accurately reflect changes in biomass during exposure to ampicillin, because cells initially grew in length without dividing.

An approximate factor converting cell coverage area to cell number density was determined by counting cells in images with a relatively low cell coverage of $2.6\times {10}^{-3}$ mm^2^ (11%) acquired early in the four experiments in this study. Based on this analysis, this imaged coverage area corresponds to an areal number density of (2.0 $\pm$ 0.2) × 10^4^ cells/mm^2^. To provide a more complete estimate of the uncertainty in this conversion, a separate test was performed in which 64 randomly selected 0.024 mm^2^ regions of the resonator surface were imaged during a 40-minute period of population growth in LB. Coverage areas from these images were fit to an exponential function versus time, which yielded a standard deviation of $0.3\times {10}^{-3}$ mm^2^ relative to the fit. This standard deviation is taken as an additional uncertainty in the estimation of overall areal density on the resonator from imaged coverage area, leading to a total uncertainty in areal density of $0.3\times {10}^{4}$ cells/mm^2^ for a coverage of 11%. We note that the imaged area in a phase-noise experiment is not actually selected randomly, being chosen as visually representative of a number of regions viewed by the experimenter. Therefore, the use of the standard deviation as an estimate of the contribution of coverage nonuniformity to the uncertainty of the estimation of total cell number is generous.

In addition to still images, 20 s videos with 6 frames per second were acquired after initial cell adhesion, immediately before introduction of antibiotic, after antibiotic flow to the module stopped, and at the end of the phase-noise experiment.

### Disclaimer

Certain trade names and company products are identified to adequately describe experimental methods. Such identification does not imply recommendation or endorsement by the National Institute of Standards and Technology, nor does it imply that the products are necessarily the best for the purpose.

### Data availability

All of the data depicted in the figures are provided in the Supplementary Information.

## Electronic supplementary material

Supplementary Information

PMB video 1

PMB video 2

PMB video 3

Ampicillin video 1

Ampicillin video 2

Ampicillin video 3

Ampicillin video 4
