# Supplementary material for: Sensing bacterial vibrations and early response to antibiotics with phase noise of a resonant crystal
Source: Sci Rep. 2017 Sep 22;7:12138. doi: 10.1038/s41598-017-12063-6 (PMC5610186; doi:10.1038/s41598-017-12063-6)
Supplement: Supplementary file 1 — Supplementary Information [file 41598_2017_12063_MOESM1_ESM.doc]

**Sensing bacterial vibrations and early response to antibiotics with phase noise of a resonant crystal**

Ward L. Johnson, Danielle Cook France, Nikki S. Rentz, William T. Cordell, and

Fred L. Walls

**Supplemental Information**

This file contains tabulations of data that are plotted in Figs. 2, 3, 4, and 6, with column headers corresponding to the axis labels and legends shown in each of the graphs.

The Supplemental Information also includes video files of images acquired during experiments, as described in *Methods*:

- Three videos of images acquired during the PMB experiment corresponding to Fig. 4(a):
- *PMB 1*: Timelapse video of all still images taken over a period of 120 min during the experiment, animated at 2 frames per second. The acquisition time of each frame, relative to the time of introduction of PMB, is shown in the upper right corner.
- *PMB 2*: Video with a duration of 20.6 s acquired 6 min before PMB was introduced. Animated at real time (6 fps).
- *PMB 3*: Video with a duration of 20.6 s acquired 5 min after PMB was introduced. Animated at real time (6 fps).
- Four videos acquired during the ampicillin experiment corresponding to Fig. 6(a):
- *Ampicillin 1*: Timelapse video of all still images taken over a period of 134 min during the experiment, animated at 2 frames per second.
- *Ampicillin 2*: Video with a duration of 20.5 s acquired 3 min before ampicillin was introduced. Animated at real time (6 fps).
- *Ampicillin 3*: Video with a duration of 20.5 s acquired 4 min after ampicillin was introduced. Animated at real time (6 fps).
- *Ampicillin 4*: Video with a duration of 19.8 s acquired 109 min after ampicillin was introduced. Animated at real time (6 fps).

**Data plotted in FIG 2(a)**

Frequency PBS without cells Cells in LB, -41.4 min Cells in LB, -5.3 min Cells in PMB, 7.2 min

0.2188 2.181E-06 3.764E-06 3.058E-06 4.355E-06

0.4375 1.566E-06 2.530E-06 2.095E-06 2.913E-06

0.6563 8.977E-07 1.277E-06 1.099E-06 1.449E-06

0.8750 4.115E-07 4.767E-07 4.383E-07 5.132E-07

1.0938 1.562E-07 1.451E-07 1.450E-07 1.267E-07

1.3125 5.496E-08 5.818E-08 5.771E-08 3.037E-08

1.5313 2.114E-08 3.628E-08 3.540E-08 1.474E-08

1.7500 9.518E-09 2.244E-08 2.532E-08 9.013E-09

1.9688 5.371E-09 1.457E-08 2.015E-08 6.417E-09

2.1875 4.033E-09 1.164E-08 1.800E-08 5.624E-09

2.4063 3.467E-09 9.938E-09 1.640E-08 4.662E-09

2.6250 3.104E-09 8.572E-09 1.490E-08 3.942E-09

2.8438 2.841E-09 7.723E-09 1.370E-08 3.659E-09

3.0625 2.577E-09 7.156E-09 1.270E-08 3.318E-09

3.2813 2.307E-09 6.752E-09 1.198E-08 2.868E-09

3.5000 2.088E-09 6.442E-09 1.167E-08 2.473E-09

3.7188 1.929E-09 6.046E-09 1.155E-08 2.167E-09

3.9375 1.815E-09 5.551E-09 1.126E-08 1.989E-09

4.1563 1.724E-09 5.141E-09 1.062E-08 1.940E-09

4.3750 1.639E-09 4.947E-09 9.734E-09 1.918E-09

4.5938 1.562E-09 4.945E-09 8.894E-09 1.869E-09

4.8125 1.504E-09 4.997E-09 8.366E-09 1.824E-09

5.0313 1.467E-09 4.972E-09 8.195E-09 1.808E-09

5.2500 1.443E-09 4.851E-09 8.209E-09 1.823E-09

5.4688 1.422E-09 4.704E-09 8.164E-09 1.843E-09

5.6875 1.394E-09 4.578E-09 7.931E-09 1.841E-09

5.9063 1.362E-09 4.478E-09 7.573E-09 1.816E-09

6.1250 1.334E-09 4.414E-09 7.240E-09 1.785E-09

6.3438 1.314E-09 4.405E-09 7.011E-09 1.750E-09

6.5625 1.295E-09 4.422E-09 6.851E-09 1.705E-09

6.7813 1.269E-09 4.388E-09 6.695E-09 1.645E-09

7.0000 1.238E-09 4.250E-09 6.513E-09 1.579E-09

7.2188 1.211E-09 4.042E-09 6.297E-09 1.515E-09

7.4375 1.197E-09 3.827E-09 6.014E-09 1.462E-09

7.6563 1.192E-09 3.620E-09 5.632E-09 1.417E-09

7.8750 1.179E-09 3.383E-09 5.167E-09 1.370E-09

8.0938 1.151E-09 3.110E-09 4.719E-09 1.323E-09

8.3125 1.115E-09 2.863E-09 4.415E-09 1.284E-09

8.5313 1.082E-09 2.719E-09 4.307E-09 1.258E-09

8.7500 1.057E-09 2.687E-09 4.325E-09 1.237E-09

8.9688 1.035E-09 2.702E-09 4.335E-09 1.198E-09

9.1875 1.011E-09 2.692E-09 4.261E-09 1.130E-09

9.4063 9.851E-10 2.637E-09 4.135E-09 1.055E-09

9.6250 9.655E-10 2.570E-09 4.037E-09 1.012E-09

9.8438 9.569E-10 2.528E-09 4.021E-09 1.023E-09

10.0625 9.603E-10 2.508E-09 4.083E-09 1.081E-09

10.2813 9.717E-10 2.475E-09 4.192E-09 1.164E-09

10.5000 9.846E-10 2.407E-09 4.295E-09 1.240E-09

10.7188 9.917E-10 2.306E-09 4.338E-09 1.290E-09

10.9375 9.885E-10 2.189E-09 4.298E-09 1.309E-09

11.1563 9.749E-10 2.069E-09 4.212E-09 1.311E-09

11.3750 9.545E-10 1.963E-09 4.130E-09 1.301E-09

11.5938 9.317E-10 1.888E-09 4.042E-09 1.271E-09

11.8125 9.098E-10 1.861E-09 3.875E-09 1.218E-09

12.0313 8.906E-10 1.878E-09 3.590E-09 1.150E-09

12.2500 8.756E-10 1.914E-09 3.265E-09 1.086E-09

12.4688 8.669E-10 1.937E-09 3.045E-09 1.043E-09

12.6875 8.651E-10 1.941E-09 2.999E-09 1.025E-09

12.9063 8.679E-10 1.941E-09 3.050E-09 1.027E-09

13.1250 8.704E-10 1.949E-09 3.066E-09 1.041E-09

13.3438 8.677E-10 1.965E-09 2.986E-09 1.057E-09

13.5625 8.583E-10 1.984E-09 2.856E-09 1.078E-09

13.7813 8.455E-10 2.004E-09 2.769E-09 1.103E-09

14.0000 8.342E-10 2.018E-09 2.775E-09 1.125E-09

14.2188 8.266E-10 2.016E-09 2.853E-09 1.123E-09

14.4375 8.212E-10 2.002E-09 2.942E-09 1.084E-09

14.6563 8.153E-10 2.000E-09 2.984E-09 1.020E-09

14.8750 8.076E-10 2.031E-09 2.956E-09 9.614E-10

15.0938 7.960E-10 2.078E-09 2.877E-09 9.267E-10

15.3125 7.790E-10 2.101E-09 2.790E-09 9.145E-10

15.5313 7.594E-10 2.069E-09 2.745E-09 9.130E-10

15.7500 7.466E-10 1.991E-09 2.772E-09 9.176E-10

15.9688 7.506E-10 1.904E-09 2.853E-09 9.336E-10

16.1875 7.708E-10 1.839E-09 2.928E-09 9.641E-10

16.4063 7.941E-10 1.797E-09 2.933E-09 9.977E-10

16.6250 8.046E-10 1.760E-09 2.850E-09 1.013E-09

16.8438 7.974E-10 1.716E-09 2.715E-09 9.982E-10

17.0625 7.815E-10 1.675E-09 2.596E-09 9.619E-10

17.2813 7.688E-10 1.649E-09 2.549E-09 9.288E-10

17.5000 7.632E-10 1.641E-09 2.582E-09 9.185E-10

17.7188 7.609E-10 1.643E-09 2.644E-09 9.327E-10

17.9375 7.593E-10 1.653E-09 2.651E-09 9.585E-10

18.1563 7.624E-10 1.669E-09 2.556E-09 9.787E-10

18.3750 7.742E-10 1.687E-09 2.394E-09 9.791E-10

18.5938 7.889E-10 1.708E-09 2.257E-09 9.522E-10

18.8125 7.938E-10 1.720E-09 2.205E-09 9.053E-10

19.0313 7.825E-10 1.704E-09 2.224E-09 8.635E-10

19.2500 7.625E-10 1.641E-09 2.250E-09 8.526E-10

19.4688 7.467E-10 1.541E-09 2.245E-09 8.744E-10

19.6875 7.390E-10 1.441E-09 2.208E-09 9.027E-10

19.9063 7.315E-10 1.375E-09 2.156E-09 9.096E-10

20.1250 7.144E-10 1.352E-09 2.093E-09 8.906E-10

20.3438 6.855E-10 1.363E-09 2.028E-09 8.622E-10

20.5625 6.519E-10 1.395E-09 1.978E-09 8.407E-10

20.7813 6.247E-10 1.433E-09 1.964E-09 8.319E-10

21.0000 6.125E-10 1.462E-09 1.984E-09 8.359E-10

21.2188 6.168E-10 1.465E-09 2.026E-09 8.496E-10

21.4375 6.307E-10 1.441E-09 2.078E-09 8.639E-10

21.6563 6.418E-10 1.400E-09 2.128E-09 8.654E-10

21.8750 6.397E-10 1.354E-09 2.165E-09 8.475E-10

22.0938 6.250E-10 1.313E-09 2.181E-09 8.186E-10

22.3125 6.087E-10 1.281E-09 2.182E-09 7.966E-10

22.5313 6.025E-10 1.263E-09 2.182E-09 7.940E-10

22.7500 6.077E-10 1.262E-09 2.190E-09 8.090E-10

22.9688 6.157E-10 1.278E-09 2.218E-09 8.298E-10

23.1875 6.174E-10 1.306E-09 2.270E-09 8.473E-10

23.4063 6.125E-10 1.330E-09 2.316E-09 8.609E-10

23.6250 6.087E-10 1.333E-09 2.295E-09 8.725E-10

23.8438 6.129E-10 1.312E-09 2.173E-09 8.790E-10

24.0625 6.247E-10 1.286E-09 2.001E-09 8.734E-10

24.2813 6.368E-10 1.284E-09 1.898E-09 8.541E-10

24.5000 6.424E-10 1.315E-09 1.941E-09 8.286E-10

24.7188 6.413E-10 1.366E-09 2.096E-09 8.084E-10

24.9375 6.374E-10 1.411E-09 2.249E-09 7.992E-10

25.1563 6.321E-10 1.428E-09 2.306E-09 7.996E-10

25.3750 6.216E-10 1.408E-09 2.258E-09 8.055E-10

25.5938 6.018E-10 1.369E-09 2.151E-09 8.152E-10

25.8125 5.748E-10 1.346E-09 2.032E-09 8.277E-10

26.0313 5.504E-10 1.355E-09 1.920E-09 8.386E-10

26.2500 5.400E-10 1.378E-09 1.823E-09 8.401E-10

26.4688 5.471E-10 1.384E-09 1.753E-09 8.286E-10

26.6875 5.641E-10 1.365E-09 1.721E-09 8.111E-10

26.9063 5.778E-10 1.329E-09 1.716E-09 8.033E-10

27.1250 5.803E-10 1.281E-09 1.708E-09 8.160E-10

27.3438 5.733E-10 1.219E-09 1.682E-09 8.417E-10

27.5625 5.634E-10 1.159E-09 1.652E-09 8.591E-10

27.7813 5.550E-10 1.128E-09 1.642E-09 8.514E-10

28.0000 5.501E-10 1.138E-09 1.658E-09 8.207E-10

28.2188 5.526E-10 1.167E-09 1.675E-09 7.832E-10

28.4375 5.708E-10 1.182E-09 1.680E-09 7.547E-10

28.6563 6.104E-10 1.178E-09 1.678E-09 7.421E-10

28.8750 6.632E-10 1.175E-09 1.687E-09 7.430E-10

29.0938 7.066E-10 1.191E-09 1.710E-09 7.495E-10

29.3125 7.201E-10 1.215E-09 1.745E-09 7.567E-10

29.5313 7.017E-10 1.222E-09 1.792E-09 7.721E-10

29.7500 6.687E-10 1.208E-09 1.848E-09 8.087E-10

29.9688 6.407E-10 1.194E-09 1.889E-09 8.648E-10

30.1875 6.253E-10 1.197E-09 1.878E-09 9.156E-10

30.4063 6.189E-10 1.203E-09 1.805E-09 9.325E-10

30.6250 6.164E-10 1.185E-09 1.701E-09 9.105E-10

30.8438 6.165E-10 1.134E-09 1.623E-09 8.723E-10

31.0625 6.205E-10 1.077E-09 1.603E-09 8.460E-10

31.2813 6.272E-10 1.051E-09 1.629E-09 8.420E-10

31.5000 6.330E-10 1.065E-09 1.662E-09 8.508E-10

31.7188 6.339E-10 1.102E-09 1.675E-09 8.561E-10

31.9375 6.278E-10 1.141E-09 1.671E-09 8.499E-10

32.1563 6.162E-10 1.169E-09 1.667E-09 8.350E-10

32.3750 6.038E-10 1.183E-09 1.660E-09 8.179E-10

32.5938 5.949E-10 1.181E-09 1.630E-09 7.992E-10

32.8125 5.891E-10 1.169E-09 1.569E-09 7.742E-10

33.0313 5.828E-10 1.157E-09 1.506E-09 7.420E-10

33.2500 5.748E-10 1.156E-09 1.483E-09 7.121E-10

33.4688 5.689E-10 1.172E-09 1.510E-09 6.980E-10

33.6875 5.698E-10 1.200E-09 1.557E-09 7.052E-10

33.9063 5.770E-10 1.218E-09 1.589E-09 7.282E-10

34.1250 5.846E-10 1.200E-09 1.595E-09 7.575E-10

34.3438 5.881E-10 1.143E-09 1.584E-09 7.854E-10

34.5625 5.876E-10 1.074E-09 1.565E-09 8.030E-10

34.7813 5.856E-10 1.034E-09 1.541E-09 7.988E-10

35.0000 5.821E-10 1.037E-09 1.514E-09 7.671E-10

35.2188 5.746E-10 1.060E-09 1.495E-09 7.191E-10

35.4375 5.620E-10 1.072E-09 1.496E-09 6.787E-10

35.6563 5.491E-10 1.064E-09 1.525E-09 6.632E-10

35.8750 5.428E-10 1.060E-09 1.577E-09 6.713E-10

36.0938 5.460E-10 1.082E-09 1.627E-09 6.897E-10

36.3125 5.542E-10 1.122E-09 1.644E-09 7.084E-10

36.5313 5.613E-10 1.142E-09 1.615E-09 7.261E-10

36.7500 5.651E-10 1.118E-09 1.560E-09 7.433E-10

36.9688 5.673E-10 1.066E-09 1.520E-09 7.575E-10

37.1875 5.696E-10 1.027E-09 1.520E-09 7.647E-10

37.4063 5.706E-10 1.022E-09 1.544E-09 7.620E-10

37.6250 5.677E-10 1.038E-09 1.561E-09 7.512E-10

37.8438 5.599E-10 1.046E-09 1.545E-09 7.379E-10

38.0625 5.481E-10 1.029E-09 1.494E-09 7.278E-10

38.2813 5.339E-10 1.001E-09 1.420E-09 7.229E-10

38.5000 5.197E-10 9.831E-10 1.337E-09 7.211E-10

38.7188 5.085E-10 9.894E-10 1.261E-09 7.199E-10

38.9375 5.030E-10 1.014E-09 1.211E-09 7.187E-10

39.1563 5.037E-10 1.044E-09 1.198E-09 7.166E-10

39.3750 5.082E-10 1.071E-09 1.224E-09 7.111E-10

39.5938 5.125E-10 1.094E-09 1.280E-09 7.014E-10

39.8125 5.125E-10 1.105E-09 1.356E-09 6.938E-10

40.0313 5.064E-10 1.092E-09 1.435E-09 7.018E-10

40.2500 4.960E-10 1.046E-09 1.493E-09 7.373E-10

40.4688 4.870E-10 9.824E-10 1.511E-09 8.000E-10

40.6875 4.854E-10 9.382E-10 1.491E-09 8.750E-10

40.9063 4.918E-10 9.436E-10 1.464E-09 9.389E-10

41.1250 4.996E-10 9.937E-10 1.456E-09 9.690E-10

41.3438 5.021E-10 1.048E-09 1.457E-09 9.532E-10

41.5625 5.001E-10 1.066E-09 1.437E-09 8.994E-10

41.7813 4.995E-10 1.037E-09 1.387E-09 8.342E-10

42.0000 5.043E-10 9.907E-10 1.328E-09 7.832E-10

42.2188 5.119E-10 9.593E-10 1.286E-09 7.519E-10

42.4375 5.182E-10 9.495E-10 1.265E-09 7.281E-10

42.6563 5.219E-10 9.442E-10 1.263E-09 7.031E-10

42.8750 5.237E-10 9.299E-10 1.281E-09 6.822E-10

43.0938 5.234E-10 9.135E-10 1.315E-09 6.740E-10

43.3125 5.201E-10 9.082E-10 1.344E-09 6.763E-10

43.5313 5.149E-10 9.145E-10 1.337E-09 6.796E-10

43.7500 5.100E-10 9.195E-10 1.293E-09 6.796E-10

43.9688 5.057E-10 9.119E-10 1.234E-09 6.810E-10

44.1875 5.004E-10 8.907E-10 1.188E-09 6.896E-10

44.4063 4.946E-10 8.618E-10 1.160E-09 7.048E-10

44.6250 4.934E-10 8.324E-10 1.147E-09 7.212E-10

44.8438 5.005E-10 8.109E-10 1.152E-09 7.337E-10

45.0625 5.130E-10 8.063E-10 1.184E-09 7.424E-10

45.2813 5.230E-10 8.233E-10 1.250E-09 7.522E-10

45.5000 5.248E-10 8.572E-10 1.336E-09 7.683E-10

45.7188 5.190E-10 8.951E-10 1.410E-09 7.905E-10

45.9375 5.091E-10 9.234E-10 1.437E-09 8.123E-10

46.1563 4.985E-10 9.343E-10 1.406E-09 8.262E-10

46.3750 4.889E-10 9.287E-10 1.336E-09 8.296E-10

46.5938 4.823E-10 9.161E-10 1.265E-09 8.237E-10

46.8125 4.807E-10 9.108E-10 1.220E-09 8.110E-10

47.0313 4.858E-10 9.235E-10 1.200E-09 7.935E-10

47.2500 4.968E-10 9.527E-10 1.191E-09 7.710E-10

47.4688 5.092E-10 9.859E-10 1.199E-09 7.413E-10

47.6875 5.164E-10 1.009E-09 1.239E-09 7.051E-10

47.9063 5.148E-10 1.015E-09 1.303E-09 6.707E-10

48.1250 5.061E-10 1.003E-09 1.348E-09 6.504E-10

48.3438 4.951E-10 9.793E-10 1.335E-09 6.519E-10

48.5625 4.844E-10 9.552E-10 1.267E-09 6.736E-10

48.7813 4.742E-10 9.482E-10 1.192E-09 7.058E-10

49.0000 4.652E-10 9.679E-10 1.171E-09 7.341E-10

49.2188 4.597E-10 1.002E-09 1.245E-09 7.463E-10

49.4375 4.594E-10 1.019E-09 1.405E-09 7.405E-10

49.6563 4.633E-10 9.994E-10 1.587E-09 7.248E-10

49.8750 4.691E-10 9.522E-10 1.701E-09 7.059E-10

50.0938 4.763E-10 9.083E-10 1.693E-09 6.822E-10

50.3125 4.845E-10 8.873E-10 1.578E-09 6.512E-10

50.5313 4.930E-10 8.817E-10 1.422E-09 6.183E-10

50.7500 4.999E-10 8.742E-10 1.291E-09 5.972E-10

50.9688 5.044E-10 8.599E-10 1.213E-09 6.030E-10

51.1875 5.062E-10 8.480E-10 1.182E-09 6.414E-10

51.4063 5.069E-10 8.485E-10 1.176E-09 6.988E-10

51.6250 5.131E-10 8.674E-10 1.176E-09 7.488E-10

51.8438 5.385E-10 9.149E-10 1.182E-09 7.805E-10

52.0625 5.949E-10 1.003E-09 1.213E-09 8.174E-10

52.2813 6.752E-10 1.125E-09 1.285E-09 8.907E-10

52.5000 7.467E-10 1.243E-09 1.384E-09 9.912E-10

52.7188 7.693E-10 1.302E-09 1.469E-09 1.062E-09

52.9375 7.287E-10 1.272E-09 1.504E-09 1.050E-09

53.1563 6.484E-10 1.173E-09 1.480E-09 9.553E-10

53.3750 5.692E-10 1.052E-09 1.415E-09 8.262E-10

53.5938 5.168E-10 9.500E-10 1.341E-09 7.168E-10

53.8125 4.915E-10 8.866E-10 1.279E-09 6.520E-10

54.0313 4.818E-10 8.652E-10 1.238E-09 6.289E-10

54.2500 4.788E-10 8.773E-10 1.212E-09 6.343E-10

54.4688 4.783E-10 9.002E-10 1.196E-09 6.566E-10

54.6875 4.781E-10 9.074E-10 1.187E-09 6.844E-10

54.9063 4.783E-10 8.905E-10 1.183E-09 7.038E-10

55.1250 4.810E-10 8.640E-10 1.175E-09 7.029E-10

55.3438 4.874E-10 8.440E-10 1.159E-09 6.842E-10

55.5625 4.953E-10 8.299E-10 1.146E-09 6.649E-10

55.7813 5.006E-10 8.137E-10 1.156E-09 6.601E-10

56.0000 5.003E-10 8.012E-10 1.194E-09 6.679E-10

56.2188 4.935E-10 8.122E-10 1.241E-09 6.752E-10

56.4375 4.805E-10 8.565E-10 1.270E-09 6.773E-10

56.6563 4.653E-10 9.185E-10 1.265E-09 6.853E-10

56.8750 4.554E-10 9.651E-10 1.230E-09 7.072E-10

57.0938 4.544E-10 9.688E-10 1.180E-09 7.337E-10

57.3125 4.592E-10 9.340E-10 1.127E-09 7.524E-10

57.5313 4.671E-10 8.968E-10 1.079E-09 7.546E-10

57.7500 4.745E-10 8.823E-10 1.036E-09 7.290E-10

57.9688 4.770E-10 8.826E-10 9.974E-10 7.047E-10

58.1875 4.918E-10 9.018E-10 1.002E-09 7.306E-10

58.4063 5.270E-10 9.251E-10 1.059E-09 7.022E-10

58.6250 5.300E-10 8.610E-10 1.068E-09 7.742E-10

58.8438 7.626E-10 1.111E-09 1.309E-09 3.113E-09

59.0625 2.707E-09 3.800E-09 3.638E-09 1.291E-08

59.2813 9.044E-09 1.256E-08 1.145E-08 3.459E-08

59.5000 2.107E-08 2.881E-08 2.640E-08 6.429E-08

59.7188 3.532E-08 4.747E-08 4.416E-08 8.783E-08

59.9375 4.423E-08 5.836E-08 5.523E-08 9.054E-08

60.1563 4.204E-08 5.442E-08 5.244E-08 7.056E-08

60.3750 3.019E-08 3.831E-08 3.763E-08 4.071E-08

60.5938 1.597E-08 1.986E-08 1.995E-08 1.652E-08

60.8125 5.934E-09 7.296E-09 7.558E-09 4.370E-09

61.0313 1.552E-09 2.023E-09 2.252E-09 9.811E-10

61.2500 5.517E-10 8.786E-10 1.119E-09 7.338E-10

61.4688 5.142E-10 8.173E-10 1.111E-09 7.678E-10

61.6875 4.997E-10 7.608E-10 1.089E-09 7.056E-10

61.9063 4.647E-10 7.031E-10 1.049E-09 6.985E-10

62.1250 4.551E-10 6.993E-10 1.052E-09 7.192E-10

62.3438 4.494E-10 7.145E-10 1.044E-09 7.255E-10

62.5625 4.416E-10 7.384E-10 9.995E-10 7.276E-10

62.7813 4.390E-10 7.781E-10 9.477E-10 7.315E-10

63.0000 4.400E-10 8.250E-10 9.165E-10 7.386E-10

63.2188 4.415E-10 8.624E-10 9.103E-10 7.537E-10

63.4375 4.447E-10 8.792E-10 9.123E-10 7.697E-10

63.6563 4.511E-10 8.777E-10 9.040E-10 7.729E-10

63.8750 4.606E-10 8.726E-10 8.876E-10 7.627E-10

64.0938 4.702E-10 8.772E-10 8.806E-10 7.529E-10

64.3125 4.747E-10 8.925E-10 8.926E-10 7.544E-10

64.5313 4.718E-10 9.094E-10 9.169E-10 7.621E-10

64.7500 4.628E-10 9.136E-10 9.451E-10 7.606E-10

64.9688 4.516E-10 8.936E-10 9.775E-10 7.402E-10

65.1875 4.429E-10 8.518E-10 1.014E-09 7.041E-10

65.4063 4.411E-10 8.098E-10 1.043E-09 6.641E-10

65.6250 4.483E-10 7.936E-10 1.050E-09 6.314E-10

65.8438 4.614E-10 8.086E-10 1.039E-09 6.140E-10

66.0625 4.724E-10 8.346E-10 1.028E-09 6.163E-10

66.2813 4.740E-10 8.477E-10 1.033E-09 6.372E-10

66.5000 4.657E-10 8.411E-10 1.051E-09 6.684E-10

66.7188 4.535E-10 8.212E-10 1.068E-09 6.961E-10

66.9375 4.436E-10 7.925E-10 1.068E-09 7.080E-10

67.1563 4.391E-10 7.590E-10 1.039E-09 7.017E-10

67.3750 4.404E-10 7.351E-10 9.805E-10 6.866E-10

67.5938 4.462E-10 7.402E-10 9.123E-10 6.761E-10

67.8125 4.538E-10 7.766E-10 8.690E-10 6.773E-10

68.0313 4.589E-10 8.213E-10 8.741E-10 6.862E-10

68.2500 4.589E-10 8.457E-10 9.192E-10 6.936E-10

68.4688 4.542E-10 8.404E-10 9.691E-10 6.942E-10

68.6875 4.485E-10 8.187E-10 9.912E-10 6.889E-10

68.9063 4.447E-10 7.993E-10 9.788E-10 6.793E-10

69.1250 4.435E-10 7.915E-10 9.499E-10 6.640E-10

69.3438 4.432E-10 7.919E-10 9.254E-10 6.428E-10

69.5625 4.418E-10 7.917E-10 9.122E-10 6.219E-10

69.7813 4.392E-10 7.851E-10 9.052E-10 6.140E-10

70.0000 4.367E-10 7.727E-10 8.996E-10 6.276E-10

70.2188 4.361E-10 7.594E-10 8.939E-10 6.585E-10

70.4375 4.372E-10 7.511E-10 8.840E-10 6.904E-10

70.6563 4.381E-10 7.521E-10 8.663E-10 7.075E-10

70.8750 4.365E-10 7.633E-10 8.487E-10 7.069E-10

71.0938 4.323E-10 7.808E-10 8.492E-10 6.980E-10

71.3125 4.279E-10 7.969E-10 8.768E-10 6.907E-10

71.5313 4.263E-10 8.029E-10 9.164E-10 6.853E-10

71.7500 4.296E-10 7.934E-10 9.396E-10 6.766E-10

71.9688 4.372E-10 7.710E-10 9.283E-10 6.645E-10

72.1875 4.468E-10 7.468E-10 8.861E-10 6.566E-10

72.4063 4.553E-10 7.333E-10 8.321E-10 6.585E-10

72.6250 4.603E-10 7.323E-10 7.880E-10 6.646E-10

72.8438 4.608E-10 7.344E-10 7.694E-10 6.637E-10

73.0625 4.578E-10 7.315E-10 7.809E-10 6.529E-10

73.2813 4.534E-10 7.286E-10 8.139E-10 6.420E-10

73.5000 4.499E-10 7.382E-10 8.490E-10 6.465E-10

73.7188 4.474E-10 7.650E-10 8.678E-10 6.759E-10

73.9375 4.452E-10 8.000E-10 8.714E-10 7.262E-10

74.1563 4.430E-10 8.281E-10 8.888E-10 7.781E-10

74.3750 4.423E-10 8.405E-10 9.567E-10 8.064E-10

74.5938 4.445E-10 8.385E-10 1.079E-09 7.973E-10

74.8125 4.492E-10 8.296E-10 1.206E-09 7.586E-10

75.0313 4.537E-10 8.204E-10 1.262E-09 7.119E-10

75.2500 4.552E-10 8.132E-10 1.210E-09 6.757E-10

75.4688 4.530E-10 8.096E-10 1.087E-09 6.557E-10

75.6875 4.484E-10 8.118E-10 9.693E-10 6.478E-10

75.9063 4.419E-10 8.201E-10 9.123E-10 6.474E-10

76.1250 4.337E-10 8.311E-10 9.131E-10 6.537E-10

76.3438 4.247E-10 8.385E-10 9.355E-10 6.677E-10

76.5625 4.182E-10 8.369E-10 9.496E-10 6.853E-10

76.7813 4.168E-10 8.245E-10 9.465E-10 6.974E-10

77.0000 4.195E-10 8.053E-10 9.306E-10 6.969E-10

77.2188 4.219E-10 7.875E-10 9.099E-10 6.860E-10

77.4375 4.208E-10 7.762E-10 8.923E-10 6.753E-10

77.6563 4.173E-10 7.674E-10 8.805E-10 6.743E-10

77.8750 4.153E-10 7.528E-10 8.699E-10 6.845E-10

78.0938 4.165E-10 7.303E-10 8.534E-10 6.973E-10

78.3125 4.191E-10 7.076E-10 8.283E-10 7.016E-10

78.5313 4.213E-10 6.969E-10 7.981E-10 6.922E-10

78.7500 4.239E-10 7.070E-10 7.698E-10 6.749E-10

78.9688 4.292E-10 7.376E-10 7.521E-10 6.600E-10

79.1875 4.380E-10 7.776E-10 7.535E-10 6.551E-10

79.4063 4.481E-10 8.075E-10 7.768E-10 6.597E-10

79.6250 4.551E-10 8.109E-10 8.141E-10 6.672E-10

79.8438 4.553E-10 7.861E-10 8.494E-10 6.694E-10

80.0625 4.489E-10 7.474E-10 8.707E-10 6.623E-10

80.2813 4.404E-10 7.156E-10 8.782E-10 6.484E-10

80.5000 4.352E-10 7.043E-10 8.808E-10 6.327E-10

80.7188 4.348E-10 7.135E-10 8.841E-10 6.179E-10

80.9375 4.362E-10 7.308E-10 8.853E-10 6.039E-10

81.1563 4.354E-10 7.394E-10 8.815E-10 5.908E-10

81.3750 4.318E-10 7.308E-10 8.772E-10 5.797E-10

81.5938 4.274E-10 7.156E-10 8.783E-10 5.714E-10

81.8125 4.238E-10 7.164E-10 8.817E-10 5.678E-10

82.0313 4.208E-10 7.450E-10 8.785E-10 5.749E-10

82.2500 4.182E-10 7.870E-10 8.662E-10 5.993E-10

82.4688 4.163E-10 8.136E-10 8.527E-10 6.399E-10

82.6875 4.163E-10 8.093E-10 8.478E-10 6.833E-10

82.9063 4.189E-10 7.842E-10 8.558E-10 7.092E-10

83.1250 4.247E-10 7.613E-10 8.756E-10 7.044E-10

83.3438 4.337E-10 7.558E-10 9.020E-10 6.730E-10

83.5625 4.446E-10 7.659E-10 9.253E-10 6.335E-10

83.7813 4.550E-10 7.800E-10 9.363E-10 6.042E-10

84.0000 4.631E-10 7.895E-10 9.363E-10 5.924E-10

84.2188 4.691E-10 7.956E-10 9.375E-10 5.948E-10

84.4375 4.744E-10 8.052E-10 9.491E-10 6.059E-10

84.6563 4.783E-10 8.198E-10 9.631E-10 6.225E-10

84.8750 4.772E-10 8.294E-10 9.584E-10 6.425E-10

85.0938 4.682E-10 8.198E-10 9.234E-10 6.626E-10

85.3125 4.533E-10 7.871E-10 8.727E-10 6.775E-10

85.5313 4.387E-10 7.434E-10 8.400E-10 6.820E-10

85.7500 4.296E-10 7.073E-10 8.506E-10 6.737E-10

85.9688 4.265E-10 6.899E-10 8.983E-10 6.570E-10

86.1875 4.260E-10 6.904E-10 9.508E-10 6.431E-10

86.4063 4.257E-10 7.007E-10 9.797E-10 6.410E-10

86.6250 4.256E-10 7.110E-10 9.808E-10 6.492E-10

86.8438 4.266E-10 7.140E-10 9.660E-10 6.561E-10

87.0625 4.277E-10 7.094E-10 9.413E-10 6.497E-10

87.2813 4.271E-10 7.036E-10 9.048E-10 6.256E-10

87.5000 4.256E-10 7.019E-10 8.617E-10 5.887E-10

87.7188 4.259E-10 7.041E-10 8.289E-10 5.513E-10

87.9375 4.297E-10 7.078E-10 8.186E-10 5.326E-10

88.1563 4.359E-10 7.143E-10 8.255E-10 5.498E-10

88.3750 4.440E-10 7.271E-10 8.382E-10 6.057E-10

88.5938 4.561E-10 7.480E-10 8.541E-10 6.812E-10

88.8125 4.753E-10 7.739E-10 8.765E-10 7.468E-10

89.0313 5.003E-10 7.949E-10 8.999E-10 7.820E-10

89.2500 5.235E-10 7.961E-10 9.105E-10 7.855E-10

89.4688 5.340E-10 7.704E-10 9.023E-10 7.678E-10

89.6875 5.252E-10 7.306E-10 8.856E-10 7.399E-10

89.9063 5.003E-10 7.008E-10 8.756E-10 7.092E-10

90.1250 4.698E-10 6.958E-10 8.756E-10 6.818E-10

90.3438 4.444E-10 7.114E-10 8.767E-10 6.618E-10

90.5625 4.287E-10 7.346E-10 8.697E-10 6.483E-10

90.7813 4.213E-10 7.550E-10 8.537E-10 6.358E-10

91.0000 4.190E-10 7.655E-10 8.354E-10 6.220E-10

91.2188 4.194E-10 7.620E-10 8.240E-10 6.134E-10

91.4375 4.220E-10 7.490E-10 8.276E-10 6.194E-10

91.6563 4.266E-10 7.394E-10 8.497E-10 6.387E-10

91.8750 4.315E-10 7.449E-10 8.839E-10 6.552E-10

92.0938 4.333E-10 7.638E-10 9.137E-10 6.535E-10

92.3125 4.284E-10 7.826E-10 9.220E-10 6.365E-10

92.5313 4.166E-10 7.884E-10 9.041E-10 6.213E-10

92.7500 4.040E-10 7.818E-10 8.715E-10 6.176E-10

92.9688 3.999E-10 7.735E-10 8.421E-10 6.184E-10

93.1875 4.118E-10 7.696E-10 8.272E-10 6.143E-10

93.4063 4.407E-10 7.618E-10 8.281E-10 6.082E-10

93.6250 4.792E-10 7.386E-10 8.395E-10 6.107E-10

93.8438 5.120E-10 7.060E-10 8.541E-10 6.233E-10

94.0625 5.243E-10 6.893E-10 8.654E-10 6.358E-10

94.2813 5.115E-10 7.097E-10 8.694E-10 6.376E-10

94.5000 4.833E-10 7.615E-10 8.667E-10 6.285E-10

94.7188 4.558E-10 8.149E-10 8.636E-10 6.170E-10

94.9375 4.384E-10 8.409E-10 8.704E-10 6.094E-10

95.1563 4.301E-10 8.296E-10 8.948E-10 6.039E-10

95.3750 4.263E-10 7.885E-10 9.303E-10 5.955E-10

95.5938 4.247E-10 7.318E-10 9.530E-10 5.853E-10

95.8125 4.243E-10 6.774E-10 9.354E-10 5.812E-10

96.0313 4.230E-10 6.438E-10 8.723E-10 5.893E-10

96.2500 4.195E-10 6.388E-10 7.900E-10 6.060E-10

96.4688 4.157E-10 6.516E-10 7.282E-10 6.208E-10

96.6875 4.155E-10 6.619E-10 7.093E-10 6.267E-10

96.9063 4.201E-10 6.603E-10 7.265E-10 6.262E-10

97.1250 4.268E-10 6.536E-10 7.580E-10 6.287E-10

97.3438 4.316E-10 6.525E-10 7.884E-10 6.427E-10

97.5625 4.330E-10 6.587E-10 8.144E-10 6.674E-10

97.7813 4.314E-10 6.676E-10 8.359E-10 6.914E-10

98.0000 4.279E-10 6.764E-10 8.486E-10 6.986E-10

98.2188 4.234E-10 6.862E-10 8.477E-10 6.804E-10

98.4375 4.202E-10 6.958E-10 8.352E-10 6.424E-10

98.6563 4.211E-10 7.000E-10 8.243E-10 6.015E-10

98.8750 4.270E-10 6.963E-10 8.363E-10 5.747E-10

99.0938 4.360E-10 6.897E-10 8.945E-10 5.712E-10

99.3125 4.444E-10 6.874E-10 1.012E-09 5.907E-10

99.5313 4.499E-10 6.891E-10 1.172E-09 6.261E-10

99.7500 4.517E-10 6.903E-10 1.322E-09 6.637E-10

99.9688 4.495E-10 6.933E-10 1.392E-09 6.865E-10

100.1875 4.432E-10 7.084E-10 1.349E-09 6.846E-10

100.4063 4.347E-10 7.387E-10 1.221E-09 6.659E-10

100.6250 4.277E-10 7.722E-10 1.073E-09 6.482E-10

100.8438 4.249E-10 7.933E-10 9.569E-10 6.402E-10

101.0625 4.259E-10 7.964E-10 8.859E-10 6.345E-10

101.2813 4.277E-10 7.857E-10 8.468E-10 6.227E-10

101.5000 4.273E-10 7.657E-10 8.245E-10 6.099E-10

101.7188 4.244E-10 7.406E-10 8.145E-10 6.085E-10

101.9375 4.211E-10 7.174E-10 8.194E-10 6.204E-10

102.1563 4.206E-10 7.000E-10 8.380E-10 6.327E-10

102.3750 4.239E-10 6.830E-10 8.566E-10 6.321E-10

102.5938 4.295E-10 6.594E-10 8.551E-10 6.199E-10

102.8125 4.335E-10 6.365E-10 8.264E-10 6.065E-10

103.0313 4.340E-10 6.417E-10 7.883E-10 6.001E-10

103.2500 4.324E-10 6.990E-10 7.677E-10 6.046E-10

103.4688 4.251E-10 7.809E-10 7.606E-10 6.119E-10

103.6875 4.155E-10 8.256E-10 7.332E-10 6.059E-10

103.9063 4.967E-10 9.225E-10 7.660E-10 6.744E-10

104.1250 9.320E-10 1.514E-09 1.259E-09 1.196E-09

104.3438 2.023E-09 3.181E-09 2.829E-09 2.752E-09

104.5625 3.744E-09 5.966E-09 5.709E-09 5.555E-09

104.7813 5.554E-09 8.977E-09 9.200E-09 8.912E-09

105.0000 6.734E-09 1.098E-08 1.196E-08 1.154E-08

105.2188 6.959E-09 1.142E-08 1.301E-08 1.255E-08

105.4375 6.426E-09 1.062E-08 1.231E-08 1.196E-08

105.6563 5.473E-09 9.104E-09 1.046E-08 1.029E-08

105.8750 4.268E-09 7.091E-09 8.020E-09 7.990E-09

106.0938 2.935E-09 4.794E-09 5.456E-09 5.426E-09

106.3125 1.727E-09 2.719E-09 3.249E-09 3.123E-09

106.5313 9.137E-10 1.371E-09 1.792E-09 1.565E-09

106.7500 5.420E-10 8.063E-10 1.122E-09 8.322E-10

106.9688 4.451E-10 6.876E-10 9.476E-10 6.167E-10

107.1875 4.376E-10 6.866E-10 9.498E-10 5.750E-10

107.4063 4.355E-10 6.913E-10 9.671E-10 5.519E-10

107.6250 4.277E-10 7.072E-10 9.628E-10 5.327E-10

107.8438 4.179E-10 7.297E-10 9.386E-10 5.284E-10

108.0625 4.083E-10 7.341E-10 9.024E-10 5.389E-10

108.2813 4.031E-10 7.106E-10 8.634E-10 5.580E-10

108.5000 4.041E-10 6.716E-10 8.307E-10 5.760E-10

108.7188 4.089E-10 6.373E-10 8.167E-10 5.828E-10

108.9375 4.133E-10 6.191E-10 8.320E-10 5.752E-10

109.1563 4.149E-10 6.138E-10 8.682E-10 5.606E-10

109.3750 4.149E-10 6.110E-10 8.969E-10 5.520E-10

109.5938 4.162E-10 6.051E-10 8.974E-10 5.579E-10

109.8125 4.193E-10 5.992E-10 8.787E-10 5.756E-10

110.0313 4.219E-10 6.007E-10 8.692E-10 5.955E-10

110.2500 4.221E-10 6.142E-10 8.844E-10 6.101E-10

110.4688 4.218E-10 6.386E-10 9.118E-10 6.167E-10

110.6875 4.247E-10 6.672E-10 9.264E-10 6.132E-10

110.9063 4.308E-10 6.913E-10 9.159E-10 5.972E-10

111.1250 4.357E-10 7.052E-10 8.871E-10 5.700E-10

111.3438 4.350E-10 7.065E-10 8.542E-10 5.395E-10

111.5625 4.292E-10 6.938E-10 8.261E-10 5.173E-10

111.7813 4.217E-10 6.676E-10 8.055E-10 5.120E-10

112.0000 4.151E-10 6.380E-10 7.949E-10 5.238E-10

112.2188 4.099E-10 6.263E-10 7.976E-10 5.440E-10

112.4375 4.060E-10 6.520E-10 8.112E-10 5.630E-10

112.6563 4.042E-10 7.134E-10 8.248E-10 5.792E-10

112.8750 4.044E-10 7.862E-10 8.291E-10 5.993E-10

113.0938 4.049E-10 8.407E-10 8.292E-10 6.268E-10

113.3125 4.040E-10 8.584E-10 8.415E-10 6.537E-10

113.5313 4.024E-10 8.366E-10 8.757E-10 6.688E-10

113.7500 4.033E-10 7.859E-10 9.222E-10 6.707E-10

113.9688 4.082E-10 7.268E-10 9.601E-10 6.672E-10

114.1875 4.149E-10 6.807E-10 9.743E-10 6.631E-10

114.4063 4.191E-10 6.572E-10 9.629E-10 6.574E-10

114.6250 4.185E-10 6.521E-10 9.305E-10 6.512E-10

114.8438 4.153E-10 6.558E-10 8.819E-10 6.513E-10

115.0625 4.123E-10 6.608E-10 8.250E-10 6.619E-10

115.2813 4.097E-10 6.605E-10 7.739E-10 6.763E-10

115.5000 4.057E-10 6.485E-10 7.424E-10 6.812E-10

115.7188 3.997E-10 6.251E-10 7.339E-10 6.702E-10

115.9375 3.941E-10 6.010E-10 7.419E-10 6.488E-10

116.1563 3.916E-10 5.907E-10 7.584E-10 6.263E-10

116.3750 3.929E-10 5.996E-10 7.779E-10 6.076E-10

116.5938 3.964E-10 6.210E-10 7.947E-10 5.939E-10

116.8125 4.000E-10 6.439E-10 8.017E-10 5.875E-10

117.0313 4.017E-10 6.636E-10 7.953E-10 5.909E-10

117.2500 4.006E-10 6.826E-10 7.784E-10 6.002E-10

117.4688 3.990E-10 7.044E-10 7.582E-10 6.010E-10

117.6875 3.997E-10 7.242E-10 7.392E-10 5.877E-10

117.9063 4.005E-10 7.310E-10 7.241E-10 5.879E-10

118.1250 4.045E-10 7.325E-10 7.303E-10 6.141E-10

118.3438 4.194E-10 7.466E-10 7.714E-10 6.155E-10

118.5625 4.203E-10 7.440E-10 7.913E-10 7.951E-10

118.7813 4.499E-10 7.964E-10 7.890E-10 2.268E-09

119.0000 1.034E-09 1.603E-09 1.340E-09 6.924E-09

119.2188 3.419E-09 4.680E-09 3.889E-09 1.547E-08

119.4375 8.661E-09 1.111E-08 9.683E-09 2.522E-08

119.6563 1.583E-08 1.946E-08 1.770E-08 3.087E-08

119.8750 2.153E-08 2.555E-08 2.411E-08 2.874E-08

120.0938 2.223E-08 2.554E-08 2.496E-08 2.026E-08

120.3125 1.746E-08 1.944E-08 1.973E-08 1.056E-08

120.5313 1.026E-08 1.112E-08 1.182E-08 3.965E-09

120.7500 4.380E-09 4.717E-09 5.344E-09 1.220E-09

120.9688 1.376E-09 1.658E-09 2.003E-09 6.620E-10

121.1875 5.020E-10 8.679E-10 9.898E-10 6.679E-10

121.4063 4.205E-10 8.357E-10 8.627E-10 6.561E-10

121.6250 4.283E-10 8.430E-10 8.563E-10 6.253E-10

121.8438 4.140E-10 8.009E-10 8.339E-10 6.102E-10

122.0625 4.060E-10 7.529E-10 8.203E-10 5.946E-10

122.2813 4.011E-10 7.135E-10 8.198E-10 5.744E-10

122.5000 3.954E-10 6.923E-10 8.279E-10 5.553E-10

122.7188 3.953E-10 6.918E-10 8.349E-10 5.390E-10

122.9375 4.016E-10 6.967E-10 8.274E-10 5.314E-10

123.1563 4.089E-10 6.898E-10 8.053E-10 5.407E-10

123.3750 4.122E-10 6.690E-10 7.805E-10 5.645E-10

123.5938 4.097E-10 6.455E-10 7.623E-10 5.888E-10

123.8125 4.035E-10 6.295E-10 7.576E-10 6.018E-10

124.0313 3.986E-10 6.211E-10 7.807E-10 6.042E-10

124.2500 3.988E-10 6.152E-10 8.490E-10 6.032E-10

124.4688 4.049E-10 6.144E-10 9.614E-10 6.024E-10

124.6875 4.144E-10 6.272E-10 1.081E-09 6.002E-10

124.9063 4.240E-10 6.542E-10 1.149E-09 5.965E-10

125.1250 4.320E-10 6.821E-10 1.132E-09 5.944E-10

125.3438 4.377E-10 6.950E-10 1.046E-09 5.951E-10

125.5625 4.399E-10 6.899E-10 9.481E-10 5.949E-10

125.7813 4.370E-10 6.788E-10 8.853E-10 5.903E-10

126.0000 4.280E-10 6.754E-10 8.631E-10 5.842E-10

126.2188 4.154E-10 6.837E-10 8.566E-10 5.841E-10

126.4375 4.044E-10 6.973E-10 8.420E-10 5.952E-10

126.6563 3.992E-10 7.076E-10 8.146E-10 6.156E-10

126.8750 3.994E-10 7.118E-10 7.828E-10 6.384E-10

127.0938 4.009E-10 7.120E-10 7.551E-10 6.554E-10

127.3125 4.013E-10 7.085E-10 7.352E-10 6.603E-10

127.5313 4.020E-10 6.968E-10 7.263E-10 6.526E-10

127.7500 4.058E-10 6.741E-10 7.332E-10 6.402E-10

127.9688 4.121E-10 6.473E-10 7.564E-10 6.328E-10

128.1875 4.165E-10 6.287E-10 7.869E-10 6.312E-10

128.4063 4.153E-10 6.233E-10 8.103E-10 6.267E-10

128.6250 4.099E-10 6.260E-10 8.170E-10 6.121E-10

128.8438 4.047E-10 6.301E-10 8.048E-10 5.904E-10

129.0625 4.031E-10 6.343E-10 7.750E-10 5.720E-10

129.2813 4.052E-10 6.387E-10 7.342E-10 5.641E-10

129.5000 4.085E-10 6.392E-10 7.011E-10 5.675E-10

129.7188 4.107E-10 6.314E-10 6.994E-10 5.782E-10

129.9375 4.108E-10 6.179E-10 7.337E-10 5.910E-10

130.1563 4.089E-10 6.088E-10 7.778E-10 6.027E-10

130.3750 4.054E-10 6.127E-10 7.976E-10 6.122E-10

130.5938 4.007E-10 6.279E-10 7.862E-10 6.204E-10

130.8125 3.958E-10 6.451E-10 7.680E-10 6.268E-10

131.0313 3.920E-10 6.556E-10 7.670E-10 6.272E-10

131.2500 3.902E-10 6.550E-10 7.789E-10 6.159E-10

131.4688 3.904E-10 6.429E-10 7.798E-10 5.924E-10

131.6875 3.930E-10 6.263E-10 7.581E-10 5.639E-10

131.9063 3.986E-10 6.199E-10 7.298E-10 5.418E-10

132.1250 4.065E-10 6.362E-10 7.194E-10 5.355E-10

132.3438 4.130E-10 6.706E-10 7.320E-10 5.470E-10

132.5625 4.139E-10 7.027E-10 7.499E-10 5.691E-10

132.7813 4.087E-10 7.151E-10 7.538E-10 5.889E-10

133.0000 4.014E-10 7.083E-10 7.402E-10 5.988E-10

133.2188 3.968E-10 6.958E-10 7.203E-10 6.033E-10

133.4375 3.958E-10 6.881E-10 7.083E-10 6.103E-10

133.6563 3.961E-10 6.874E-10 7.110E-10 6.165E-10

133.8750 3.960E-10 6.923E-10 7.252E-10 6.103E-10

134.0938 3.960E-10 7.023E-10 7.410E-10 5.905E-10

134.3125 3.967E-10 7.133E-10 7.504E-10 5.747E-10

134.5313 3.971E-10 7.159E-10 7.518E-10 5.809E-10

134.7500 3.953E-10 7.039E-10 7.474E-10 6.062E-10

134.9688 3.912E-10 6.828E-10 7.403E-10 6.286E-10

135.1875 3.867E-10 6.641E-10 7.354E-10 6.296E-10

135.4063 3.836E-10 6.533E-10 7.400E-10 6.113E-10

135.6250 3.821E-10 6.480E-10 7.583E-10 5.905E-10

135.8438 3.809E-10 6.474E-10 7.852E-10 5.831E-10

136.0625 3.793E-10 6.541E-10 8.104E-10 5.919E-10

136.2813 3.777E-10 6.648E-10 8.283E-10 6.085E-10

136.5000 3.773E-10 6.675E-10 8.401E-10 6.222E-10

136.7188 3.788E-10 6.552E-10 8.474E-10 6.254E-10

136.9375 3.813E-10 6.359E-10 8.457E-10 6.145E-10

137.1563 3.834E-10 6.253E-10 8.283E-10 5.920E-10

137.3750 3.837E-10 6.291E-10 7.988E-10 5.693E-10

137.5938 3.820E-10 6.386E-10 7.735E-10 5.639E-10

137.8125 3.792E-10 6.419E-10 7.666E-10 5.849E-10

138.0313 3.765E-10 6.359E-10 7.730E-10 6.226E-10

138.2500 3.754E-10 6.278E-10 7.739E-10 6.570E-10

138.4688 3.775E-10 6.278E-10 7.595E-10 6.741E-10

138.6875 3.838E-10 6.402E-10 7.407E-10 6.726E-10

138.9063 3.922E-10 6.595E-10 7.355E-10 6.562E-10

139.1250 3.978E-10 6.743E-10 7.488E-10 6.266E-10

139.3438 3.976E-10 6.757E-10 7.693E-10 5.884E-10

139.5625 3.937E-10 6.629E-10 7.819E-10 5.549E-10

139.7813 3.918E-10 6.435E-10 7.787E-10 5.424E-10

140.0000 3.947E-10 6.269E-10 7.626E-10 5.546E-10

140.2188 4.001E-10 6.188E-10 7.420E-10 5.773E-10

140.4375 4.040E-10 6.186E-10 7.230E-10 5.906E-10

140.6563 4.059E-10 6.221E-10 7.062E-10 5.858E-10

140.8750 4.073E-10 6.260E-10 6.925E-10 5.699E-10

141.0938 4.089E-10 6.295E-10 6.881E-10 5.543E-10

141.3125 4.090E-10 6.319E-10 6.984E-10 5.429E-10

141.5313 4.066E-10 6.285E-10 7.173E-10 5.311E-10

141.7500 4.038E-10 6.144E-10 7.275E-10 5.167E-10

141.9688 4.034E-10 5.959E-10 7.172E-10 5.062E-10

142.1875 4.061E-10 5.926E-10 6.925E-10 5.092E-10

142.4063 4.094E-10 6.200E-10 6.721E-10 5.256E-10

142.6250 4.097E-10 6.716E-10 6.711E-10 5.426E-10

142.8438 4.066E-10 7.209E-10 6.911E-10 5.481E-10

143.0625 4.029E-10 7.435E-10 7.191E-10 5.456E-10

143.2813 4.017E-10 7.341E-10 7.372E-10 5.493E-10

143.5000 4.034E-10 7.058E-10 7.344E-10 5.679E-10

143.7188 4.061E-10 6.796E-10 7.139E-10 5.977E-10

143.9375 4.074E-10 6.698E-10 6.890E-10 6.303E-10

144.1563 4.065E-10 6.752E-10 6.711E-10 6.595E-10

144.3750 4.029E-10 6.809E-10 6.615E-10 6.815E-10

144.5938 3.967E-10 6.720E-10 6.534E-10 6.944E-10

144.8125 3.889E-10 6.458E-10 6.397E-10 7.003E-10

145.0313 3.829E-10 6.126E-10 6.184E-10 7.018E-10

145.2500 3.817E-10 5.874E-10 5.959E-10 6.957E-10

145.4688 3.849E-10 5.804E-10 5.844E-10 6.755E-10

145.6875 3.888E-10 5.919E-10 5.950E-10 6.403E-10

145.9063 3.898E-10 6.133E-10 6.292E-10 5.983E-10

146.1250 3.879E-10 6.325E-10 6.777E-10 5.597E-10

146.3438 3.872E-10 6.381E-10 7.275E-10 5.300E-10

146.5625 3.919E-10 6.238E-10 7.682E-10 5.094E-10

146.7813 4.026E-10 5.936E-10 7.928E-10 4.971E-10

147.0000 4.145E-10 5.629E-10 7.978E-10 4.932E-10

147.2188 4.213E-10 5.497E-10 7.862E-10 4.997E-10

147.4375 4.203E-10 5.613E-10 7.674E-10 5.169E-10

147.6563 4.148E-10 5.902E-10 7.500E-10 5.388E-10

147.8750 4.117E-10 6.213E-10 7.354E-10 5.535E-10

148.0938 4.148E-10 6.409E-10 7.227E-10 5.512E-10

148.3125 4.214E-10 6.411E-10 7.173E-10 5.348E-10

148.5313 4.246E-10 6.222E-10 7.295E-10 5.190E-10

148.7500 4.203E-10 5.934E-10 7.622E-10 5.191E-10

148.9688 4.115E-10 5.698E-10 8.054E-10 5.383E-10

149.1875 4.050E-10 5.632E-10 8.448E-10 5.666E-10

149.4063 4.041E-10 5.752E-10 8.737E-10 5.900E-10

149.6250 4.067E-10 5.964E-10 8.923E-10 6.012E-10

149.8438 4.084E-10 6.145E-10 8.969E-10 6.028E-10

150.0625 4.075E-10 6.230E-10 8.768E-10 6.033E-10

150.2813 4.049E-10 6.259E-10 8.281E-10 6.095E-10

150.5000 4.016E-10 6.341E-10 7.663E-10 6.229E-10

150.7188 3.980E-10 6.541E-10 7.193E-10 6.379E-10

150.9375 3.944E-10 6.796E-10 7.051E-10 6.436E-10

151.1563 3.918E-10 6.959E-10 7.179E-10 6.299E-10

151.3750 3.909E-10 6.946E-10 7.338E-10 5.974E-10

151.5938 3.913E-10 6.819E-10 7.293E-10 5.617E-10

151.8125 3.924E-10 6.703E-10 6.988E-10 5.431E-10

152.0313 3.940E-10 6.654E-10 6.588E-10 5.483E-10

152.2500 3.952E-10 6.638E-10 6.346E-10 5.633E-10

152.4688 3.948E-10 6.609E-10 6.385E-10 5.687E-10

152.6875 3.916E-10 6.565E-10 6.607E-10 5.570E-10

152.9063 3.864E-10 6.502E-10 6.823E-10 5.367E-10

153.1250 3.808E-10 6.377E-10 6.923E-10 5.198E-10

153.3438 3.756E-10 6.153E-10 6.909E-10 5.117E-10

153.5625 3.708E-10 5.890E-10 6.811E-10 5.118E-10

153.7813 3.684E-10 5.736E-10 6.637E-10 5.181E-10

154.0000 3.718E-10 5.798E-10 6.414E-10 5.292E-10

154.2188 3.813E-10 6.020E-10 6.216E-10 5.431E-10

154.4375 3.921E-10 6.248E-10 6.118E-10 5.559E-10

154.6563 3.985E-10 6.383E-10 6.130E-10 5.625E-10

154.8750 3.989E-10 6.444E-10 6.206E-10 5.604E-10

155.0938 3.960E-10 6.484E-10 6.316E-10 5.514E-10

155.3125 3.930E-10 6.518E-10 6.483E-10 5.394E-10

155.5313 3.912E-10 6.553E-10 6.721E-10 5.265E-10

155.7500 3.899E-10 6.631E-10 6.959E-10 5.134E-10

155.9688 3.886E-10 6.774E-10 7.093E-10 5.025E-10

156.1875 3.873E-10 6.913E-10 7.141E-10 4.998E-10

156.4063 3.868E-10 6.930E-10 7.265E-10 5.090E-10

156.6250 3.872E-10 6.781E-10 7.566E-10 5.280E-10

156.8438 3.883E-10 6.564E-10 7.899E-10 5.508E-10

157.0625 3.891E-10 6.437E-10 7.952E-10 5.720E-10

157.2813 3.890E-10 6.493E-10 7.550E-10 5.868E-10

157.5000 3.913E-10 6.735E-10 6.904E-10 5.980E-10

157.7188 4.047E-10 7.167E-10 6.585E-10 6.259E-10

157.9375 4.395E-10 7.848E-10 7.171E-10 7.019E-10

158.1563 4.945E-10 8.755E-10 8.750E-10 8.309E-10

158.3750 5.483E-10 9.598E-10 1.065E-09 9.612E-10

158.5938 5.696E-10 9.917E-10 1.179E-09 1.012E-09

158.8125 5.433E-10 9.489E-10 1.146E-09 9.475E-10

159.0313 4.841E-10 8.582E-10 9.878E-10 8.076E-10

159.2500 4.239E-10 7.708E-10 7.982E-10 6.717E-10

159.4688 3.853E-10 7.140E-10 6.658E-10 5.894E-10

159.6875 3.702E-10 6.764E-10 6.218E-10 5.585E-10

159.9063 3.690E-10 6.361E-10 6.463E-10 5.520E-10

160.1250 3.729E-10 5.908E-10 7.020E-10 5.480E-10

160.3438 3.779E-10 5.567E-10 7.566E-10 5.391E-10

160.5625 3.830E-10 5.484E-10 7.879E-10 5.280E-10

160.7813 3.873E-10 5.647E-10 7.861E-10 5.213E-10

161.0000 3.906E-10 5.922E-10 7.551E-10 5.236E-10

161.2188 3.930E-10 6.168E-10 7.064E-10 5.337E-10

161.4375 3.941E-10 6.311E-10 6.518E-10 5.439E-10

161.6563 3.941E-10 6.354E-10 6.026E-10 5.464E-10

161.8750 3.945E-10 6.331E-10 5.707E-10 5.410E-10

162.0938 3.975E-10 6.278E-10 5.638E-10 5.372E-10

162.3125 4.027E-10 6.229E-10 5.785E-10 5.448E-10

162.5313 4.068E-10 6.190E-10 5.999E-10 5.625E-10

162.7500 4.067E-10 6.126E-10 6.139E-10 5.784E-10

162.9688 4.025E-10 5.998E-10 6.195E-10 5.844E-10

163.1875 3.967E-10 5.840E-10 6.316E-10 5.863E-10

163.4063 3.906E-10 5.768E-10 6.664E-10 5.967E-10

163.6250 3.836E-10 5.878E-10 7.210E-10 6.182E-10

163.8438 3.773E-10 6.129E-10 7.704E-10 6.371E-10

164.0625 3.759E-10 6.372E-10 7.891E-10 6.367E-10

164.2813 3.827E-10 6.490E-10 7.747E-10 6.151E-10

164.5000 3.958E-10 6.481E-10 7.472E-10 5.891E-10

164.7188 4.101E-10 6.391E-10 7.280E-10 5.776E-10

164.9375 4.208E-10 6.242E-10 7.218E-10 5.826E-10

165.1563 4.262E-10 6.064E-10 7.179E-10 5.898E-10

165.3750 4.266E-10 5.938E-10 7.042E-10 5.859E-10

165.5938 4.231E-10 5.945E-10 6.809E-10 5.730E-10

165.8125 4.167E-10 6.074E-10 6.608E-10 5.636E-10

166.0313 4.088E-10 6.234E-10 6.572E-10 5.663E-10

166.2500 4.009E-10 6.338E-10 6.701E-10 5.780E-10

166.4688 3.940E-10 6.327E-10 6.847E-10 5.875E-10

166.6875 3.892E-10 6.164E-10 6.829E-10 5.863E-10

166.9063 3.873E-10 5.866E-10 6.578E-10 5.744E-10

167.1250 3.883E-10 5.547E-10 6.178E-10 5.583E-10

167.3438 3.900E-10 5.345E-10 5.807E-10 5.433E-10

167.5625 3.897E-10 5.302E-10 5.636E-10 5.316E-10

167.7813 3.869E-10 5.328E-10 5.742E-10 5.245E-10

168.0000 3.843E-10 5.316E-10 6.096E-10 5.254E-10

168.2188 3.844E-10 5.257E-10 6.588E-10 5.370E-10

168.4375 3.858E-10 5.236E-10 7.057E-10 5.571E-10

168.6563 3.838E-10 5.333E-10 7.319E-10 5.754E-10

168.8750 3.763E-10 5.554E-10 7.231E-10 5.785E-10

169.0938 3.664E-10 5.837E-10 6.798E-10 5.627E-10

169.3125 3.598E-10 6.092E-10 6.210E-10 5.413E-10

169.5313 3.587E-10 6.232E-10 5.747E-10 5.347E-10

169.7500 3.621E-10 6.188E-10 5.572E-10 5.502E-10

169.9688 3.683E-10 5.974E-10 5.624E-10 5.748E-10

170.1875 3.768E-10 5.718E-10 5.729E-10 5.913E-10

170.4063 3.858E-10 5.597E-10 5.803E-10 5.978E-10

170.6250 3.917E-10 5.700E-10 5.903E-10 6.075E-10

170.8438 3.915E-10 5.934E-10 6.106E-10 6.294E-10

171.0625 3.851E-10 6.102E-10 6.370E-10 6.555E-10

171.2813 3.756E-10 6.081E-10 6.571E-10 6.666E-10

171.5000 3.671E-10 5.920E-10 6.618E-10 6.509E-10

171.7188 3.622E-10 5.738E-10 6.527E-10 6.144E-10

171.9375 3.618E-10 5.602E-10 6.396E-10 5.745E-10

172.1563 3.654E-10 5.510E-10 6.328E-10 5.443E-10

172.3750 3.711E-10 5.460E-10 6.365E-10 5.240E-10

172.5938 3.771E-10 5.458E-10 6.473E-10 5.068E-10

172.8125 3.817E-10 5.484E-10 6.588E-10 4.905E-10

173.0313 3.842E-10 5.518E-10 6.674E-10 4.818E-10

173.2500 3.850E-10 5.588E-10 6.734E-10 4.883E-10

173.4688 3.853E-10 5.723E-10 6.755E-10 5.095E-10

173.6875 3.844E-10 5.868E-10 6.690E-10 5.356E-10

173.9063 3.808E-10 5.907E-10 6.548E-10 5.538E-10

174.1250 3.735E-10 5.805E-10 6.494E-10 5.590E-10

174.3438 3.638E-10 5.675E-10 6.770E-10 5.589E-10

174.5625 3.555E-10 5.676E-10 7.454E-10 5.642E-10

174.7813 3.529E-10 5.853E-10 8.298E-10 5.734E-10

175.0000 3.572E-10 6.074E-10 8.846E-10 5.745E-10

175.2188 3.658E-10 6.137E-10 8.796E-10 5.619E-10

175.4375 3.761E-10 5.974E-10 8.263E-10 5.463E-10

175.6563 3.859E-10 5.705E-10 7.649E-10 5.461E-10

175.8750 3.925E-10 5.521E-10 7.288E-10 5.624E-10

176.0938 3.950E-10 5.557E-10 7.254E-10 5.739E-10

176.3125 3.956E-10 5.768E-10 7.405E-10 5.710E-10

176.5313 3.950E-10 5.958E-10 7.522E-10 5.645E-10

176.7500 3.970E-10 6.020E-10 7.508E-10 5.577E-10

176.9688 4.041E-10 5.983E-10 7.341E-10 5.669E-10

177.1875 4.036E-10 5.871E-10 6.885E-10 6.046E-10

177.4063 3.973E-10 5.882E-10 6.324E-10 5.960E-10

177.6250 4.075E-10 6.058E-10 6.256E-10 5.798E-10

177.8438 4.030E-10 5.699E-10 6.451E-10 8.071E-10

178.0625 3.920E-10 5.416E-10 6.497E-10 8.597E-10

178.2813 5.300E-10 7.402E-10 7.725E-10 7.674E-10

178.5000 5.924E-10 7.622E-10 8.263E-10 9.328E-09

178.7188 5.093E-10 8.454E-10 8.174E-10 5.225E-08

178.9375 5.722E-09 9.176E-09 6.821E-09 1.577E-07

179.1563 3.341E-08 4.779E-08 3.818E-08 3.165E-07

179.3750 1.043E-07 1.385E-07 1.186E-07 4.600E-07

179.5938 2.153E-07 2.697E-07 2.454E-07 5.021E-07

179.8125 3.217E-07 3.820E-07 3.674E-07 4.144E-07

180.0313 3.611E-07 4.065E-07 4.130E-07 2.542E-07

180.2500 3.075E-07 3.269E-07 3.518E-07 1.100E-07

180.4688 1.960E-07 1.947E-07 2.237E-07 2.967E-08

180.6875 8.957E-08 8.122E-08 1.015E-07 3.593E-09

180.9063 2.652E-08 2.081E-08 2.953E-08 5.290E-10

181.1250 4.025E-09 2.420E-09 4.318E-09 9.304E-10

181.3438 4.630E-10 6.375E-10 6.644E-10 6.677E-10

181.5625 6.143E-10 9.441E-10 9.780E-10 5.032E-10

181.7813 4.882E-10 7.363E-10 8.111E-10 5.355E-10

182.0000 3.822E-10 6.485E-10 6.331E-10 5.382E-10

182.2188 4.077E-10 6.761E-10 6.170E-10 5.408E-10

182.4375 4.101E-10 6.529E-10 6.014E-10 5.639E-10

182.6563 4.046E-10 6.225E-10 6.013E-10 5.709E-10

182.8750 4.132E-10 6.027E-10 6.180E-10 5.663E-10

183.0938 4.120E-10 5.778E-10 6.092E-10 5.624E-10

183.3125 4.031E-10 5.658E-10 5.896E-10 5.597E-10

183.5313 3.981E-10 5.744E-10 5.872E-10 5.664E-10

183.7500 3.955E-10 5.872E-10 5.983E-10 5.871E-10

183.9688 3.943E-10 5.919E-10 6.108E-10 6.097E-10

184.1875 3.944E-10 5.852E-10 6.179E-10 6.191E-10

184.4063 3.940E-10 5.686E-10 6.170E-10 6.095E-10

184.6250 3.950E-10 5.501E-10 6.126E-10 5.876E-10

184.8438 3.987E-10 5.367E-10 6.102E-10 5.672E-10

185.0625 4.012E-10 5.321E-10 6.101E-10 5.573E-10

185.2813 3.987E-10 5.398E-10 6.113E-10 5.574E-10

185.5000 3.922E-10 5.617E-10 6.153E-10 5.625E-10

185.7188 3.855E-10 5.918E-10 6.255E-10 5.669E-10

185.9375 3.832E-10 6.164E-10 6.424E-10 5.665E-10

186.1563 3.872E-10 6.230E-10 6.590E-10 5.621E-10

186.3750 3.956E-10 6.094E-10 6.648E-10 5.594E-10

186.5938 4.040E-10 5.833E-10 6.563E-10 5.623E-10

186.8125 4.094E-10 5.555E-10 6.400E-10 5.682E-10

187.0313 4.113E-10 5.350E-10 6.261E-10 5.700E-10

187.2500 4.102E-10 5.281E-10 6.198E-10 5.628E-10

187.4688 4.052E-10 5.347E-10 6.195E-10 5.477E-10

187.6875 3.956E-10 5.466E-10 6.200E-10 5.280E-10

187.9063 3.840E-10 5.541E-10 6.180E-10 5.059E-10

188.1250 3.751E-10 5.553E-10 6.127E-10 4.839E-10

188.3438 3.712E-10 5.561E-10 6.034E-10 4.672E-10

188.5625 3.701E-10 5.602E-10 5.897E-10 4.625E-10

188.7813 3.689E-10 5.632E-10 5.743E-10 4.732E-10

189.0000 3.676E-10 5.585E-10 5.647E-10 4.947E-10

189.2188 3.686E-10 5.487E-10 5.687E-10 5.171E-10

189.4375 3.733E-10 5.474E-10 5.878E-10 5.325E-10

189.6563 3.798E-10 5.667E-10 6.141E-10 5.410E-10

189.8750 3.852E-10 6.015E-10 6.349E-10 5.484E-10

190.0938 3.881E-10 6.314E-10 6.416E-10 5.567E-10

190.3125 3.893E-10 6.394E-10 6.380E-10 5.613E-10

190.5313 3.893E-10 6.267E-10 6.351E-10 5.590E-10

190.7500 3.875E-10 6.070E-10 6.375E-10 5.560E-10

190.9688 3.837E-10 5.895E-10 6.370E-10 5.629E-10

191.1875 3.796E-10 5.745E-10 6.236E-10 5.815E-10

191.4063 3.772E-10 5.625E-10 5.992E-10 6.004E-10

191.6250 3.766E-10 5.589E-10 5.768E-10 6.058E-10

191.8438 3.765E-10 5.662E-10 5.679E-10 5.944E-10

192.0625 3.762E-10 5.768E-10 5.745E-10 5.756E-10

192.2813 3.763E-10 5.801E-10 5.912E-10 5.599E-10

192.5000 3.771E-10 5.742E-10 6.108E-10 5.504E-10

192.7188 3.780E-10 5.664E-10 6.267E-10 5.457E-10

192.9375 3.784E-10 5.614E-10 6.360E-10 5.482E-10

193.1563 3.776E-10 5.538E-10 6.391E-10 5.643E-10

193.3750 3.744E-10 5.383E-10 6.385E-10 5.950E-10

193.5938 3.694E-10 5.215E-10 6.355E-10 6.293E-10

193.8125 3.656E-10 5.192E-10 6.290E-10 6.505E-10

194.0313 3.664E-10 5.394E-10 6.159E-10 6.510E-10

194.2500 3.718E-10 5.728E-10 5.944E-10 6.388E-10

194.4688 3.784E-10 6.004E-10 5.684E-10 6.287E-10

194.6875 3.830E-10 6.074E-10 5.495E-10 6.287E-10

194.9063 3.857E-10 5.916E-10 5.506E-10 6.355E-10

195.1250 3.885E-10 5.645E-10 5.755E-10 6.431E-10

195.3438 3.930E-10 5.470E-10 6.130E-10 6.513E-10

195.5625 3.983E-10 5.558E-10 6.442E-10 6.627E-10

195.7813 4.017E-10 5.884E-10 6.573E-10 6.728E-10

196.0000 4.007E-10 6.231E-10 6.553E-10 6.700E-10

196.2188 3.941E-10 6.382E-10 6.502E-10 6.485E-10

196.4375 3.828E-10 6.277E-10 6.522E-10 6.167E-10

196.6563 3.697E-10 6.000E-10 6.631E-10 5.900E-10

196.8750 3.588E-10 5.673E-10 6.762E-10 5.753E-10

197.0938 3.541E-10 5.395E-10 6.812E-10 5.678E-10

197.3125 3.573E-10 5.219E-10 6.719E-10 5.617E-10

197.5313 3.658E-10 5.133E-10 6.530E-10 5.570E-10

197.7500 3.740E-10 5.073E-10 6.354E-10 5.564E-10

197.9688 3.772E-10 4.999E-10 6.250E-10 5.581E-10

198.1875 3.757E-10 4.926E-10 6.176E-10 5.592E-10

198.4063 3.740E-10 4.879E-10 6.072E-10 5.607E-10

198.6250 3.760E-10 4.863E-10 5.940E-10 5.656E-10

198.8438 3.815E-10 4.889E-10 5.853E-10 5.718E-10

199.0625 3.867E-10 4.996E-10 5.904E-10 5.723E-10

199.2813 3.887E-10 5.216E-10 6.186E-10 5.654E-10

199.5000 3.876E-10 5.521E-10 6.740E-10 5.584E-10

199.7188 3.859E-10 5.830E-10 7.476E-10 5.600E-10

199.9375 3.850E-10 6.071E-10 8.142E-10 5.716E-10

200.1563 3.839E-10 6.233E-10 8.435E-10 5.886E-10

200.3750 3.807E-10 6.345E-10 8.198E-10 6.053E-10

200.5938 3.758E-10 6.414E-10 7.535E-10 6.159E-10

200.8125 3.716E-10 6.401E-10 6.756E-10 6.146E-10

201.0313 3.697E-10 6.266E-10 6.178E-10 6.008E-10

201.2500 3.694E-10 6.024E-10 5.942E-10 5.840E-10

201.4688 3.700E-10 5.731E-10 5.981E-10 5.768E-10

201.6875 3.716E-10 5.457E-10 6.132E-10 5.843E-10

201.9063 3.747E-10 5.277E-10 6.264E-10 6.001E-10

202.1250 3.784E-10 5.260E-10 6.315E-10 6.130E-10

202.3438 3.808E-10 5.412E-10 6.285E-10 6.155E-10

202.5625 3.809E-10 5.647E-10 6.223E-10 6.063E-10

202.7813 3.790E-10 5.835E-10 6.167E-10 5.893E-10

203.0000 3.752E-10 5.912E-10 6.096E-10 5.711E-10

203.2188 3.692E-10 5.916E-10 5.964E-10 5.569E-10

203.4375 3.617E-10 5.905E-10 5.797E-10 5.484E-10

203.6563 3.551E-10 5.867E-10 5.705E-10 5.449E-10

203.8750 3.523E-10 5.720E-10 5.768E-10 5.455E-10

204.0938 3.549E-10 5.427E-10 5.958E-10 5.499E-10

204.3125 3.619E-10 5.080E-10 6.181E-10 5.570E-10

204.5313 3.711E-10 4.853E-10 6.361E-10 5.626E-10

204.7500 3.797E-10 4.858E-10 6.455E-10 5.606E-10

204.9688 3.853E-10 5.072E-10 6.437E-10 5.476E-10

205.1875 3.870E-10 5.370E-10 6.326E-10 5.273E-10

205.4063 3.857E-10 5.633E-10 6.207E-10 5.098E-10

205.6250 3.840E-10 5.796E-10 6.171E-10 5.035E-10

205.8438 3.844E-10 5.836E-10 6.238E-10 5.085E-10

206.0625 3.872E-10 5.756E-10 6.335E-10 5.184E-10

206.2813 3.902E-10 5.613E-10 6.355E-10 5.275E-10

206.5000 3.908E-10 5.510E-10 6.244E-10 5.349E-10

206.7188 3.881E-10 5.531E-10 6.047E-10 5.415E-10

206.9375 3.839E-10 5.649E-10 5.868E-10 5.462E-10

207.1563 3.821E-10 5.733E-10 5.783E-10 5.460E-10

207.3750 3.847E-10 5.653E-10 5.784E-10 5.404E-10

207.5938 3.895E-10 5.414E-10 5.806E-10 5.343E-10

207.8125 3.918E-10 5.173E-10 5.802E-10 5.359E-10

208.0313 3.900E-10 5.107E-10 5.775E-10 5.503E-10

208.2500 3.872E-10 5.244E-10 5.757E-10 5.742E-10

208.4688 3.877E-10 5.411E-10 5.783E-10 5.948E-10

208.6875 4.047E-10 5.454E-10 5.890E-10 6.005E-10

208.9063 4.884E-10 5.720E-10 6.273E-10 6.082E-10

209.1250 7.342E-10 7.331E-10 7.596E-10 6.965E-10

209.3438 1.212E-09 1.149E-09 1.097E-09 9.896E-10

209.5625 1.841E-09 1.788E-09 1.693E-09 1.548E-09

209.7813 2.364E-09 2.386E-09 2.403E-09 2.226E-09

210.0000 2.512E-09 2.600E-09 2.889E-09 2.681E-09

210.2188 2.236E-09 2.320E-09 2.892E-09 2.626E-09

210.4375 1.784E-09 1.835E-09 2.530E-09 2.171E-09

210.6563 1.529E-09 1.667E-09 2.295E-09 1.879E-09

210.8750 1.684E-09 2.140E-09 2.646E-09 2.323E-09

211.0938 2.149E-09 3.070E-09 3.538E-09 3.495E-09

211.3125 2.572E-09 3.845E-09 4.376E-09 4.666E-09

211.5313 2.608E-09 3.896E-09 4.499E-09 4.967E-09

211.7500 2.178E-09 3.162E-09 3.739E-09 4.146E-09

211.9688 1.505E-09 2.087E-09 2.524E-09 2.735E-09

212.1875 9.054E-10 1.201E-09 1.451E-09 1.497E-09

212.4063 5.502E-10 7.292E-10 8.303E-10 8.137E-10

212.6250 4.100E-10 5.775E-10 5.968E-10 5.861E-10

212.8438 3.746E-10 5.580E-10 5.470E-10 5.466E-10

213.0625 3.678E-10 5.586E-10 5.461E-10 5.349E-10

213.2813 3.660E-10 5.504E-10 5.543E-10 5.215E-10

213.5000 3.659E-10 5.359E-10 5.669E-10 5.150E-10

213.7188 3.658E-10 5.219E-10 5.831E-10 5.146E-10

213.9375 3.650E-10 5.133E-10 6.045E-10 5.159E-10

214.1563 3.650E-10 5.103E-10 6.296E-10 5.193E-10

214.3750 3.661E-10 5.110E-10 6.472E-10 5.248E-10

214.5938 3.674E-10 5.140E-10 6.437E-10 5.301E-10

214.8125 3.678E-10 5.185E-10 6.172E-10 5.341E-10

215.0313 3.665E-10 5.239E-10 5.829E-10 5.375E-10

215.2500 3.641E-10 5.298E-10 5.636E-10 5.403E-10

215.4688 3.615E-10 5.358E-10 5.722E-10 5.422E-10

215.6875 3.592E-10 5.400E-10 6.027E-10 5.450E-10

215.9063 3.566E-10 5.413E-10 6.363E-10 5.510E-10

216.1250 3.536E-10 5.408E-10 6.555E-10 5.568E-10

216.3438 3.518E-10 5.408E-10 6.545E-10 5.540E-10

216.5625 3.542E-10 5.419E-10 6.398E-10 5.402E-10

216.7813 3.617E-10 5.442E-10 6.234E-10 5.247E-10

217.0000 3.724E-10 5.486E-10 6.144E-10 5.198E-10

217.2188 3.827E-10 5.571E-10 6.141E-10 5.286E-10

217.4375 3.893E-10 5.708E-10 6.160E-10 5.438E-10

217.6563 3.900E-10 5.876E-10 6.114E-10 5.572E-10

217.8750 3.852E-10 6.016E-10 5.963E-10 5.671E-10

218.0938 3.790E-10 6.064E-10 5.759E-10 5.759E-10

218.3125 3.757E-10 6.006E-10 5.603E-10 5.827E-10

218.5313 3.767E-10 5.896E-10 5.573E-10 5.810E-10

218.7500 3.790E-10 5.775E-10 5.663E-10 5.655E-10

218.9688 3.796E-10 5.612E-10 5.793E-10 5.406E-10

219.1875 3.790E-10 5.371E-10 5.863E-10 5.200E-10

219.4063 3.797E-10 5.113E-10 5.824E-10 5.140E-10

219.6250 3.821E-10 4.975E-10 5.740E-10 5.198E-10

219.8438 3.840E-10 5.026E-10 5.758E-10 5.263E-10

220.0625 3.829E-10 5.205E-10 5.967E-10 5.287E-10

220.2813 3.780E-10 5.399E-10 6.255E-10 5.335E-10

220.5000 3.712E-10 5.531E-10 6.372E-10 5.460E-10

220.7188 3.647E-10 5.562E-10 6.179E-10 5.596E-10

220.9375 3.602E-10 5.496E-10 5.800E-10 5.629E-10

221.1563 3.575E-10 5.408E-10 5.491E-10 5.531E-10

221.3750 3.561E-10 5.430E-10 5.387E-10 5.381E-10

221.5938 3.557E-10 5.618E-10 5.434E-10 5.255E-10

221.8125 3.573E-10 5.866E-10 5.521E-10 5.149E-10

222.0313 3.613E-10 5.987E-10 5.592E-10 5.034E-10

222.2500 3.670E-10 5.881E-10 5.642E-10 4.930E-10

222.4688 3.729E-10 5.606E-10 5.706E-10 4.907E-10

222.6875 3.772E-10 5.305E-10 5.853E-10 5.027E-10

222.9063 3.780E-10 5.082E-10 6.134E-10 5.276E-10

223.1250 3.738E-10 4.965E-10 6.468E-10 5.555E-10

223.3438 3.658E-10 4.917E-10 6.665E-10 5.736E-10

223.5625 3.585E-10 4.899E-10 6.610E-10 5.754E-10

223.7813 3.567E-10 4.900E-10 6.394E-10 5.671E-10

224.0000 3.609E-10 4.939E-10 6.270E-10 5.604E-10

224.2188 3.674E-10 5.022E-10 6.497E-10 5.610E-10

224.4375 3.727E-10 5.111E-10 7.168E-10 5.634E-10

224.6563 3.767E-10 5.160E-10 8.087E-10 5.575E-10

224.8750 3.813E-10 5.176E-10 8.799E-10 5.378E-10

225.0938 3.861E-10 5.234E-10 8.862E-10 5.080E-10

225.3125 3.872E-10 5.395E-10 8.206E-10 4.806E-10

225.5313 3.816E-10 5.614E-10 7.213E-10 4.690E-10

225.7500 3.711E-10 5.746E-10 6.389E-10 4.783E-10

225.9688 3.618E-10 5.676E-10 5.948E-10 5.025E-10

226.1875 3.594E-10 5.449E-10 5.751E-10 5.320E-10

226.4063 3.651E-10 5.229E-10 5.582E-10 5.604E-10

226.6250 3.746E-10 5.129E-10 5.398E-10 5.834E-10

226.8438 3.810E-10 5.121E-10 5.298E-10 5.960E-10

227.0625 3.803E-10 5.142E-10 5.347E-10 5.947E-10

227.2813 3.735E-10 5.222E-10 5.506E-10 5.828E-10

227.5000 3.653E-10 5.432E-10 5.693E-10 5.685E-10

227.7188 3.595E-10 5.726E-10 5.834E-10 5.567E-10

227.9375 3.574E-10 5.932E-10 5.872E-10 5.463E-10

228.1563 3.580E-10 5.894E-10 5.789E-10 5.352E-10

228.3750 3.597E-10 5.606E-10 5.634E-10 5.230E-10

228.5938 3.613E-10 5.191E-10 5.492E-10 5.104E-10

228.8125 3.620E-10 4.824E-10 5.415E-10 4.988E-10

229.0313 3.619E-10 4.647E-10 5.415E-10 4.905E-10

229.2500 3.619E-10 4.696E-10 5.500E-10 4.874E-10

229.4688 3.637E-10 4.866E-10 5.678E-10 4.884E-10

229.6875 3.672E-10 4.997E-10 5.920E-10 4.891E-10

229.9063 3.700E-10 5.008E-10 6.142E-10 4.870E-10

230.1250 3.687E-10 4.951E-10 6.256E-10 4.848E-10

230.3438 3.622E-10 4.905E-10 6.217E-10 4.867E-10

230.5625 3.533E-10 4.884E-10 6.050E-10 4.933E-10

230.7813 3.475E-10 4.844E-10 5.844E-10 5.027E-10

231.0000 3.480E-10 4.777E-10 5.710E-10 5.152E-10

231.2188 3.540E-10 4.754E-10 5.699E-10 5.338E-10

231.4375 3.615E-10 4.869E-10 5.780E-10 5.577E-10

231.6563 3.674E-10 5.155E-10 5.902E-10 5.783E-10

231.8750 3.721E-10 5.558E-10 6.091E-10 5.868E-10

232.0938 3.785E-10 5.969E-10 6.390E-10 5.845E-10

232.3125 3.870E-10 6.275E-10 6.742E-10 5.813E-10

232.5313 3.943E-10 6.418E-10 6.981E-10 5.839E-10

232.7500 3.967E-10 6.416E-10 6.991E-10 5.891E-10

232.9688 3.932E-10 6.354E-10 6.808E-10 5.904E-10

233.1875 3.858E-10 6.307E-10 6.547E-10 5.863E-10

233.4063 3.770E-10 6.283E-10 6.285E-10 5.816E-10

233.6250 3.690E-10 6.244E-10 6.041E-10 5.807E-10

233.8438 3.643E-10 6.158E-10 5.816E-10 5.820E-10

234.0625 3.643E-10 6.041E-10 5.607E-10 5.806E-10

234.2813 3.671E-10 5.930E-10 5.408E-10 5.747E-10

234.5000 3.678E-10 5.837E-10 5.241E-10 5.667E-10

234.7188 3.629E-10 5.718E-10 5.149E-10 5.580E-10

234.9375 3.537E-10 5.510E-10 5.136E-10 5.471E-10

235.1563 3.453E-10 5.218E-10 5.161E-10 5.343E-10

235.3750 3.417E-10 4.952E-10 5.194E-10 5.238E-10

235.5938 3.431E-10 4.838E-10 5.250E-10 5.188E-10

235.8125 3.471E-10 4.900E-10 5.352E-10 5.155E-10

236.0313 3.516E-10 5.042E-10 5.468E-10 5.076E-10

236.2500 3.567E-10 5.140E-10 5.531E-10 4.945E-10

236.4688 3.624E-10 5.139E-10 5.530E-10 4.828E-10

236.6875 3.679E-10 5.075E-10 5.530E-10 4.809E-10

236.9063 3.717E-10 5.026E-10 5.598E-10 4.915E-10

237.1250 3.733E-10 5.036E-10 5.705E-10 5.076E-10

237.3438 3.739E-10 5.095E-10 5.760E-10 5.166E-10

237.5625 3.745E-10 5.172E-10 5.745E-10 5.160E-10

237.7813 3.737E-10 5.222E-10 5.731E-10 5.104E-10

238.0000 3.696E-10 5.222E-10 5.788E-10 4.892E-10

238.2188 3.662E-10 5.243E-10 5.936E-10 5.159E-10

238.4375 3.638E-10 5.275E-10 6.026E-10 9.876E-10

238.6563 3.586E-10 5.295E-10 5.757E-10 2.680E-09

238.8750 4.460E-10 7.036E-10 6.044E-10 6.060E-09

239.0938 1.005E-09 1.648E-09 1.151E-09 1.027E-08

239.3125 2.614E-09 4.145E-09 2.971E-09 1.316E-08

239.5313 5.431E-09 8.183E-09 6.335E-09 1.292E-08

239.7500 8.539E-09 1.219E-08 1.017E-08 9.736E-09

239.9688 1.026E-08 1.385E-08 1.240E-08 5.606E-09

240.1875 9.480E-09 1.205E-08 1.158E-08 2.515E-09

240.4063 6.722E-09 7.980E-09 8.261E-09 1.045E-09

240.6250 3.618E-09 3.973E-09 4.465E-09 6.238E-10

240.8438 1.504E-09 1.531E-09 1.879E-09 5.457E-10

241.0625 6.038E-10 6.218E-10 8.118E-10 5.042E-10

241.2813 3.986E-10 4.655E-10 6.114E-10 4.666E-10

241.5000 3.868E-10 4.811E-10 6.273E-10 4.461E-10

241.7188 3.808E-10 4.972E-10 6.191E-10 4.426E-10

241.9375 3.703E-10 5.113E-10 5.909E-10 4.530E-10

242.1563 3.634E-10 5.243E-10 5.718E-10 4.678E-10

242.3750 3.580E-10 5.296E-10 5.649E-10 4.737E-10

242.5938 3.553E-10 5.238E-10 5.687E-10 4.663E-10

242.8125 3.564E-10 5.071E-10 5.802E-10 4.517E-10

243.0313 3.590E-10 4.875E-10 5.921E-10 4.389E-10

243.2500 3.613E-10 4.775E-10 5.994E-10 4.330E-10

243.4688 3.641E-10 4.821E-10 6.039E-10 4.325E-10

243.6875 3.686E-10 4.937E-10 6.082E-10 4.331E-10

243.9063 3.738E-10 4.998E-10 6.094E-10 4.353E-10

244.1250 3.773E-10 4.947E-10 6.011E-10 4.456E-10

244.3438 3.777E-10 4.844E-10 5.823E-10 4.681E-10

244.5625 3.759E-10 4.795E-10 5.599E-10 4.983E-10

244.7813 3.741E-10 4.851E-10 5.422E-10 5.260E-10

245.0000 3.733E-10 4.975E-10 5.338E-10 5.452E-10

245.2188 3.733E-10 5.112E-10 5.376E-10 5.578E-10

245.4375 3.728E-10 5.233E-10 5.553E-10 5.675E-10

245.6563 3.709E-10 5.316E-10 5.807E-10 5.743E-10

245.8750 3.682E-10 5.316E-10 5.974E-10 5.764E-10

246.0938 3.668E-10 5.210E-10 5.910E-10 5.770E-10

246.3125 3.676E-10 5.036E-10 5.676E-10 5.826E-10

246.5313 3.701E-10 4.880E-10 5.536E-10 5.928E-10

246.7500 3.732E-10 4.810E-10 5.695E-10 5.974E-10

246.9688 3.768E-10 4.847E-10 6.072E-10 5.869E-10

247.1875 3.811E-10 4.967E-10 6.383E-10 5.639E-10

247.4063 3.851E-10 5.126E-10 6.439E-10 5.396E-10

247.6250 3.870E-10 5.274E-10 6.317E-10 5.214E-10

247.8438 3.851E-10 5.373E-10 6.240E-10 5.106E-10

248.0625 3.799E-10 5.413E-10 6.351E-10 5.080E-10

248.2813 3.738E-10 5.408E-10 6.614E-10 5.149E-10

248.5000 3.699E-10 5.382E-10 6.895E-10 5.276E-10

248.7188 3.683E-10 5.356E-10 7.067E-10 5.366E-10

248.9375 3.666E-10 5.330E-10 7.092E-10 5.355E-10

249.1563 3.635E-10 5.287E-10 7.066E-10 5.271E-10

249.3750 3.612E-10 5.219E-10 7.176E-10 5.192E-10

249.5938 3.630E-10 5.149E-10 7.542E-10 5.162E-10

249.8125 3.687E-10 5.120E-10 8.045E-10 5.161E-10

250.0313 3.735E-10 5.141E-10 8.386E-10 5.159E-10

250.2500 3.731E-10 5.166E-10 8.345E-10 5.183E-10

250.4688 3.683E-10 5.142E-10 7.980E-10 5.277E-10

250.6875 3.631E-10 5.092E-10 7.536E-10 5.422E-10

250.9063 3.602E-10 5.104E-10 7.207E-10 5.521E-10

251.1250 3.592E-10 5.221E-10 6.996E-10 5.502E-10

251.3438 3.593E-10 5.356E-10 6.772E-10 5.409E-10

251.5625 3.611E-10 5.392E-10 6.446E-10 5.343E-10

251.7813 3.648E-10 5.339E-10 6.076E-10 5.329E-10

252.0000 3.687E-10 5.322E-10 5.824E-10 5.301E-10

252.2188 3.709E-10 5.412E-10 5.778E-10 5.223E-10

252.4375 3.710E-10 5.506E-10 5.853E-10 5.177E-10

252.6563 3.708E-10 5.469E-10 5.884E-10 5.291E-10

252.8750 3.722E-10 5.314E-10 5.804E-10 5.582E-10

253.0938 3.753E-10 5.206E-10 5.688E-10 5.911E-10

253.3125 3.788E-10 5.272E-10 5.636E-10 6.108E-10

253.5313 3.807E-10 5.457E-10 5.651E-10 6.124E-10

253.7500 3.806E-10 5.591E-10 5.661E-10 6.048E-10

253.9688 3.803E-10 5.546E-10 5.634E-10 5.995E-10

254.1875 3.821E-10 5.341E-10 5.632E-10 5.992E-10

254.4063 3.843E-10 5.089E-10 5.731E-10 5.984E-10

254.6250 3.830E-10 4.901E-10 5.914E-10 5.899E-10

254.8438 3.767E-10 4.832E-10 6.075E-10 5.712E-10

255.0625 3.698E-10 4.893E-10 6.112E-10 5.462E-10

255.2813 3.682E-10 5.072E-10 5.988E-10 5.238E-10

255.5000 3.722E-10 5.326E-10 5.739E-10 5.123E-10

255.7188 3.766E-10 5.571E-10 5.452E-10 5.117E-10

255.9375 3.757E-10 5.712E-10 5.231E-10 5.137E-10

256.1563 3.687E-10 5.717E-10 5.147E-10 5.118E-10

256.3750 3.596E-10 5.646E-10 5.204E-10 5.088E-10

256.5938 3.532E-10 5.605E-10 5.358E-10 5.111E-10

256.8125 3.519E-10 5.630E-10 5.551E-10 5.188E-10

257.0313 3.539E-10 5.656E-10 5.726E-10 5.272E-10

257.2500 3.555E-10 5.622E-10 5.837E-10 5.356E-10

257.4688 3.542E-10 5.586E-10 5.878E-10 5.475E-10

257.6875 3.507E-10 5.683E-10 5.883E-10 5.597E-10

257.9063 3.481E-10 5.943E-10 5.871E-10 5.610E-10

258.1250 3.488E-10 6.204E-10 5.822E-10 5.461E-10

258.3438 3.530E-10 6.263E-10 5.700E-10 5.263E-10

258.5625 3.586E-10 6.079E-10 5.503E-10 5.210E-10

258.7813 3.626E-10 5.783E-10 5.269E-10 5.370E-10

259.0000 3.632E-10 5.509E-10 5.055E-10 5.618E-10

259.2188 3.604E-10 5.276E-10 4.922E-10 5.755E-10

259.4375 3.566E-10 5.033E-10 4.930E-10 5.661E-10

259.6563 3.545E-10 4.767E-10 5.106E-10 5.361E-10

259.8750 3.544E-10 4.535E-10 5.394E-10 5.004E-10

260.0938 3.545E-10 4.437E-10 5.664E-10 4.767E-10

260.3125 3.539E-10 4.561E-10 5.809E-10 4.743E-10

260.5313 3.549E-10 4.905E-10 5.832E-10 4.863E-10

260.7500 3.602E-10 5.337E-10 5.823E-10 4.971E-10

260.9688 3.686E-10 5.638E-10 5.859E-10 4.974E-10

261.1875 3.751E-10 5.655E-10 5.927E-10 4.925E-10

261.4063 3.754E-10 5.424E-10 5.953E-10 4.936E-10

261.6250 3.697E-10 5.145E-10 5.874E-10 5.041E-10

261.8438 3.613E-10 5.038E-10 5.703E-10 5.166E-10

262.0625 3.541E-10 5.184E-10 5.519E-10 5.218E-10

262.2813 3.507E-10 5.493E-10 5.405E-10 5.188E-10

262.5000 3.516E-10 5.795E-10 5.411E-10 5.168E-10

262.7188 3.552E-10 5.965E-10 5.548E-10 5.250E-10

262.9375 3.592E-10 5.984E-10 5.781E-10 5.421E-10

263.1563 3.634E-10 5.913E-10 6.042E-10 5.572E-10

263.3750 3.716E-10 5.846E-10 6.264E-10 5.638E-10

263.5938 3.911E-10 5.861E-10 6.433E-10 5.702E-10

263.8125 4.255E-10 5.972E-10 6.586E-10 5.895E-10

264.0313 4.664E-10 6.078E-10 6.730E-10 6.184E-10

264.2500 4.942E-10 6.032E-10 6.788E-10 6.327E-10

264.4688 4.919E-10 5.804E-10 6.674E-10 6.115E-10

264.6875 4.600E-10 5.527E-10 6.406E-10 5.632E-10

264.9063 4.161E-10 5.361E-10 6.107E-10 5.213E-10

265.1250 3.798E-10 5.316E-10 5.899E-10 5.130E-10

265.3438 3.595E-10 5.284E-10 5.828E-10 5.354E-10

265.5625 3.521E-10 5.209E-10 5.888E-10 5.622E-10

265.7813 3.510E-10 5.156E-10 6.058E-10 5.694E-10

266.0000 3.523E-10 5.200E-10 6.283E-10 5.529E-10

266.2188 3.546E-10 5.301E-10 6.460E-10 5.265E-10

266.4375 3.576E-10 5.341E-10 6.482E-10 5.072E-10

266.6563 3.604E-10 5.251E-10 6.309E-10 5.047E-10

266.8750 3.613E-10 5.087E-10 5.988E-10 5.162E-10

267.0938 3.593E-10 4.979E-10 5.640E-10 5.306E-10

267.3125 3.548E-10 5.023E-10 5.413E-10 5.375E-10

267.5313 3.492E-10 5.211E-10 5.418E-10 5.358E-10

267.7500 3.447E-10 5.426E-10 5.647E-10 5.320E-10

267.9688 3.436E-10 5.544E-10 5.943E-10 5.317E-10

268.1875 3.473E-10 5.549E-10 6.068E-10 5.340E-10

268.4063 3.554E-10 5.526E-10 5.870E-10 5.371E-10

268.6250 3.651E-10 5.533E-10 5.401E-10 5.414E-10

268.8438 3.723E-10 5.510E-10 4.902E-10 5.456E-10

269.0625 3.747E-10 5.373E-10 4.627E-10 5.441E-10

269.2813 3.733E-10 5.155E-10 4.673E-10 5.327E-10

269.5000 3.702E-10 4.996E-10 4.926E-10 5.151E-10

269.7188 3.672E-10 4.985E-10 5.183E-10 4.987E-10

269.9375 3.643E-10 5.054E-10 5.319E-10 4.868E-10

270.1563 3.615E-10 5.082E-10 5.360E-10 4.774E-10

270.3750 3.600E-10 5.044E-10 5.403E-10 4.683E-10

270.5938 3.614E-10 5.024E-10 5.493E-10 4.620E-10

270.8125 3.656E-10 5.089E-10 5.595E-10 4.630E-10

271.0313 3.710E-10 5.188E-10 5.674E-10 4.728E-10

271.2500 3.749E-10 5.217E-10 5.742E-10 4.868E-10

271.4688 3.755E-10 5.145E-10 5.815E-10 4.980E-10

271.6875 3.725E-10 5.033E-10 5.839E-10 5.022E-10

271.9063 3.676E-10 4.952E-10 5.741E-10 4.993E-10

272.1250 3.630E-10 4.904E-10 5.531E-10 4.891E-10

272.3438 3.604E-10 4.867E-10 5.313E-10 4.725E-10

272.5625 3.596E-10 4.856E-10 5.179E-10 4.555E-10

272.7813 3.593E-10 4.897E-10 5.132E-10 4.495E-10

273.0000 3.586E-10 4.956E-10 5.140E-10 4.610E-10

273.2188 3.580E-10 4.948E-10 5.204E-10 4.845E-10

273.4375 3.584E-10 4.829E-10 5.319E-10 5.072E-10

273.6563 3.597E-10 4.657E-10 5.422E-10 5.225E-10

273.8750 3.609E-10 4.539E-10 5.449E-10 5.336E-10

274.0938 3.609E-10 4.559E-10 5.491E-10 5.465E-10

274.3125 3.601E-10 4.740E-10 5.797E-10 5.623E-10

274.5313 3.598E-10 5.034E-10 6.495E-10 5.772E-10

274.7500 3.615E-10 5.325E-10 7.324E-10 5.862E-10

274.9688 3.655E-10 5.499E-10 7.767E-10 5.842E-10

275.1875 3.706E-10 5.521E-10 7.525E-10 5.684E-10

275.4063 3.744E-10 5.456E-10 6.812E-10 5.435E-10

275.6250 3.750E-10 5.387E-10 6.136E-10 5.221E-10

275.8438 3.723E-10 5.348E-10 5.824E-10 5.148E-10

276.0625 3.685E-10 5.335E-10 5.807E-10 5.205E-10

276.2813 3.656E-10 5.340E-10 5.816E-10 5.293E-10

276.5000 3.644E-10 5.359E-10 5.698E-10 5.335E-10

276.7188 3.642E-10 5.371E-10 5.538E-10 5.320E-10

276.9375 3.641E-10 5.354E-10 5.508E-10 5.285E-10

277.1563 3.640E-10 5.326E-10 5.644E-10 5.283E-10

277.3750 3.649E-10 5.341E-10 5.794E-10 5.357E-10

277.5938 3.675E-10 5.441E-10 5.775E-10 5.469E-10

277.8125 3.707E-10 5.623E-10 5.555E-10 5.497E-10

278.0313 3.718E-10 5.841E-10 5.263E-10 5.333E-10

278.2500 3.687E-10 6.022E-10 5.062E-10 5.032E-10

278.4688 3.625E-10 6.092E-10 5.023E-10 4.790E-10

278.6875 3.571E-10 6.004E-10 5.102E-10 4.769E-10

278.9063 3.554E-10 5.765E-10 5.200E-10 4.941E-10

279.1250 3.577E-10 5.448E-10 5.236E-10 5.132E-10

279.3438 3.623E-10 5.173E-10 5.197E-10 5.189E-10

279.5625 3.675E-10 5.055E-10 5.133E-10 5.090E-10

279.7813 3.720E-10 5.117E-10 5.103E-10 4.925E-10

280.0000 3.747E-10 5.269E-10 5.141E-10 4.812E-10

280.2188 3.762E-10 5.376E-10 5.261E-10 4.823E-10

280.4375 3.778E-10 5.380E-10 5.472E-10 4.959E-10

280.6563 3.798E-10 5.334E-10 5.768E-10 5.156E-10

280.8750 3.809E-10 5.313E-10 6.088E-10 5.318E-10

281.0938 3.792E-10 5.334E-10 6.314E-10 5.371E-10

281.3125 3.743E-10 5.361E-10 6.342E-10 5.305E-10

281.5313 3.676E-10 5.358E-10 6.168E-10 5.185E-10

281.7500 3.623E-10 5.312E-10 5.896E-10 5.122E-10

281.9688 3.617E-10 5.221E-10 5.625E-10 5.196E-10

282.1875 3.665E-10 5.105E-10 5.368E-10 5.378E-10

282.4063 3.731E-10 5.028E-10 5.091E-10 5.538E-10

282.6250 3.758E-10 5.065E-10 4.817E-10 5.550E-10

282.8438 3.723E-10 5.231E-10 4.653E-10 5.395E-10

283.0625 3.661E-10 5.436E-10 4.695E-10 5.171E-10

283.2813 3.651E-10 5.554E-10 4.940E-10 5.019E-10

283.5000 3.787E-10 5.547E-10 5.314E-10 5.014E-10

283.7188 4.155E-10 5.525E-10 5.754E-10 5.135E-10

283.9375 4.804E-10 5.683E-10 6.222E-10 5.329E-10

284.1563 5.688E-10 6.151E-10 6.655E-10 5.610E-10

284.3750 6.631E-10 6.876E-10 6.977E-10 6.050E-10

284.5938 7.369E-10 7.616E-10 7.199E-10 6.673E-10

284.8125 7.665E-10 8.049E-10 7.424E-10 7.344E-10

285.0313 7.420E-10 7.961E-10 7.684E-10 7.788E-10

285.2500 6.717E-10 7.384E-10 7.819E-10 7.762E-10

285.4688 5.788E-10 6.572E-10 7.603E-10 7.249E-10

285.6875 4.918E-10 5.822E-10 6.996E-10 6.473E-10

285.9063 4.310E-10 5.293E-10 6.211E-10 5.718E-10

286.1250 3.987E-10 4.975E-10 5.515E-10 5.152E-10

286.3438 3.829E-10 4.797E-10 5.038E-10 4.819E-10

286.5625 3.714E-10 4.723E-10 4.766E-10 4.725E-10

286.7813 3.618E-10 4.752E-10 4.653E-10 4.839E-10

287.0000 3.577E-10 4.863E-10 4.664E-10 5.048E-10

287.2188 3.602E-10 4.980E-10 4.755E-10 5.185E-10

287.4375 3.645E-10 5.017E-10 4.881E-10 5.167E-10

287.6563 3.650E-10 4.954E-10 5.065E-10 5.071E-10

287.8750 3.610E-10 4.846E-10 5.384E-10 5.038E-10

288.0938 3.569E-10 4.776E-10 5.852E-10 5.119E-10

288.3125 3.580E-10 4.799E-10 6.333E-10 5.249E-10

288.5313 3.650E-10 4.938E-10 6.607E-10 5.359E-10

288.7500 3.735E-10 5.159E-10 6.550E-10 5.445E-10

288.9688 3.772E-10 5.366E-10 6.226E-10 5.503E-10

289.1875 3.740E-10 5.464E-10 5.802E-10 5.472E-10

289.4063 3.673E-10 5.462E-10 5.406E-10 5.292E-10

289.6250 3.623E-10 5.473E-10 5.091E-10 4.999E-10

289.8438 3.612E-10 5.577E-10 4.894E-10 4.732E-10

290.0625 3.622E-10 5.730E-10 4.885E-10 4.636E-10

290.2813 3.630E-10 5.820E-10 5.098E-10 4.770E-10

290.5000 3.633E-10 5.766E-10 5.439E-10 5.074E-10

290.7188 3.644E-10 5.573E-10 5.705E-10 5.404E-10

290.9375 3.663E-10 5.311E-10 5.738E-10 5.609E-10

291.1563 3.670E-10 5.089E-10 5.580E-10 5.619E-10

291.3750 3.647E-10 5.016E-10 5.443E-10 5.460E-10

291.5938 3.597E-10 5.134E-10 5.507E-10 5.217E-10

291.8125 3.545E-10 5.383E-10 5.731E-10 4.983E-10

292.0313 3.511E-10 5.633E-10 5.878E-10 4.836E-10

292.2500 3.500E-10 5.737E-10 5.745E-10 4.808E-10

292.4688 3.513E-10 5.623E-10 5.364E-10 4.878E-10

292.6875 3.550E-10 5.341E-10 4.991E-10 4.993E-10

292.9063 3.601E-10 5.044E-10 4.887E-10 5.105E-10

293.1250 3.635E-10 4.867E-10 5.103E-10 5.208E-10

293.3438 3.624E-10 4.821E-10 5.435E-10 5.320E-10

293.5625 3.577E-10 4.817E-10 5.589E-10 5.452E-10

293.7813 3.525E-10 4.794E-10 5.417E-10 5.570E-10

294.0000 3.485E-10 4.798E-10 5.029E-10 5.620E-10

294.2188 3.450E-10 4.906E-10 4.696E-10 5.591E-10

294.4375 3.415E-10 5.096E-10 4.614E-10 5.523E-10

294.6563 3.402E-10 5.240E-10 4.766E-10 5.467E-10

294.8750 3.431E-10 5.219E-10 4.979E-10 5.430E-10

295.0938 3.498E-10 5.036E-10 5.097E-10 5.411E-10

295.3125 3.567E-10 4.806E-10 5.090E-10 5.440E-10

295.5313 3.600E-10 4.653E-10 5.031E-10 5.546E-10

295.7500 3.590E-10 4.618E-10 4.997E-10 5.696E-10

295.9688 3.558E-10 4.674E-10 5.019E-10 5.814E-10

296.1875 3.534E-10 4.786E-10 5.115E-10 5.825E-10

296.4063 3.533E-10 4.940E-10 5.305E-10 5.661E-10

296.6250 3.541E-10 5.114E-10 5.550E-10 5.352E-10

296.8438 3.545E-10 5.266E-10 5.738E-10 5.083E-10

297.0625 3.530E-10 5.321E-10 5.759E-10 4.940E-10

297.2813 3.488E-10 5.249E-10 5.606E-10 4.919E-10

297.5000 3.473E-10 5.145E-10 5.379E-10 5.242E-10

297.7188 3.527E-10 5.059E-10 5.141E-10 5.561E-10

297.9375 3.597E-10 4.894E-10 4.886E-10 5.441E-10

298.1563 3.792E-10 4.750E-10 4.838E-10 1.421E-09

298.3750 4.196E-10 4.666E-10 5.177E-10 6.507E-09

298.5938 4.074E-10 4.339E-10 5.147E-10 2.028E-08

298.8125 5.535E-10 8.626E-10 6.288E-10 4.295E-08

299.0313 2.513E-09 3.876E-09 2.290E-09 6.598E-08

299.2500 9.823E-09 1.291E-08 8.599E-09 7.619E-08

299.4688 2.513E-08 2.911E-08 2.185E-08 6.685E-08

299.6875 4.536E-08 4.724E-08 3.929E-08 4.413E-08

299.9063 6.089E-08 5.738E-08 5.241E-08 2.119E-08

300.1250 6.223E-08 5.287E-08 5.305E-08 6.968E-09

300.3438 4.852E-08 3.671E-08 4.076E-08 1.590E-09

300.5625 2.834E-08 1.862E-08 2.328E-08 6.077E-10

300.7813 1.188E-08 6.489E-09 9.439E-09 5.929E-10

301.0000 3.349E-09 1.501E-09 2.592E-09 5.553E-10

301.2188 7.349E-10 4.896E-10 7.035E-10 5.324E-10

301.4375 4.090E-10 5.065E-10 5.621E-10 5.412E-10

301.6563 4.091E-10 5.200E-10 5.704E-10 5.377E-10

301.8750 3.749E-10 5.119E-10 5.217E-10 5.310E-10

302.0938 3.649E-10 5.274E-10 5.068E-10 5.388E-10

302.3125 3.694E-10 5.381E-10 5.210E-10 5.545E-10

302.5313 3.676E-10 5.334E-10 5.396E-10 5.644E-10

302.7500 3.662E-10 5.145E-10 5.523E-10 5.582E-10

302.9688 3.695E-10 4.832E-10 5.457E-10 5.327E-10

303.1875 3.742E-10 4.533E-10 5.195E-10 4.975E-10

303.4063 3.761E-10 4.397E-10 4.932E-10 4.687E-10

303.6250 3.721E-10 4.441E-10 4.832E-10 4.582E-10

303.8438 3.632E-10 4.603E-10 4.875E-10 4.667E-10

304.0625 3.546E-10 4.815E-10 4.942E-10 4.859E-10

304.2813 3.502E-10 5.021E-10 4.985E-10 5.048E-10

304.5000 3.504E-10 5.166E-10 5.067E-10 5.166E-10

304.7188 3.546E-10 5.219E-10 5.267E-10 5.202E-10

304.9375 3.610E-10 5.189E-10 5.559E-10 5.174E-10

305.1563 3.670E-10 5.142E-10 5.827E-10 5.111E-10

305.3750 3.711E-10 5.130E-10 5.947E-10 5.023E-10

305.5938 3.728E-10 5.133E-10 5.869E-10 4.895E-10

305.8125 3.726E-10 5.105E-10 5.660E-10 4.739E-10

306.0313 3.713E-10 5.052E-10 5.453E-10 4.636E-10

306.2500 3.694E-10 5.034E-10 5.338E-10 4.675E-10

306.4688 3.668E-10 5.101E-10 5.302E-10 4.829E-10

306.6875 3.638E-10 5.247E-10 5.288E-10 4.948E-10

306.9063 3.613E-10 5.416E-10 5.266E-10 4.914E-10

307.1250 3.606E-10 5.530E-10 5.245E-10 4.770E-10

307.3438 3.630E-10 5.534E-10 5.242E-10 4.659E-10

307.5625 3.689E-10 5.456E-10 5.282E-10 4.651E-10

307.7813 3.764E-10 5.381E-10 5.390E-10 4.704E-10

308.0000 3.811E-10 5.377E-10 5.556E-10 4.778E-10

308.2188 3.799E-10 5.433E-10 5.710E-10 4.917E-10

308.4375 3.737E-10 5.486E-10 5.746E-10 5.176E-10

308.6563 3.667E-10 5.496E-10 5.598E-10 5.529E-10

308.8750 3.623E-10 5.467E-10 5.278E-10 5.849E-10

309.0938 3.596E-10 5.432E-10 4.872E-10 5.985E-10

309.3125 3.556E-10 5.437E-10 4.533E-10 5.863E-10

309.5313 3.483E-10 5.498E-10 4.415E-10 5.548E-10

309.7500 3.387E-10 5.588E-10 4.582E-10 5.213E-10

309.9688 3.310E-10 5.645E-10 4.942E-10 5.024E-10

310.1875 3.295E-10 5.609E-10 5.314E-10 5.029E-10

310.4063 3.358E-10 5.449E-10 5.551E-10 5.144E-10

310.6250 3.463E-10 5.184E-10 5.624E-10 5.242E-10

310.8438 3.552E-10 4.896E-10 5.597E-10 5.248E-10

311.0625 3.583E-10 4.684E-10 5.540E-10 5.185E-10

311.2813 3.557E-10 4.602E-10 5.477E-10 5.152E-10

311.5000 3.510E-10 4.611E-10 5.397E-10 5.227E-10

311.7188 3.474E-10 4.613E-10 5.297E-10 5.373E-10

311.9375 3.461E-10 4.552E-10 5.196E-10 5.452E-10

312.1563 3.480E-10 4.492E-10 5.111E-10 5.378E-10

312.3750 3.524E-10 4.541E-10 5.021E-10 5.208E-10

312.5938 3.573E-10 4.743E-10 4.877E-10 5.089E-10

312.8125 3.597E-10 5.029E-10 4.680E-10 5.102E-10

313.0313 3.577E-10 5.275E-10 4.517E-10 5.188E-10

313.2500 3.526E-10 5.390E-10 4.509E-10 5.229E-10

313.4688 3.494E-10 5.393E-10 4.714E-10 5.173E-10

313.6875 3.529E-10 5.408E-10 5.087E-10 5.052E-10

313.9063 3.644E-10 5.574E-10 5.539E-10 4.915E-10

314.1250 3.823E-10 5.913E-10 6.008E-10 4.783E-10

314.3438 4.025E-10 6.277E-10 6.444E-10 4.670E-10

314.5625 4.193E-10 6.463E-10 6.780E-10 4.645E-10

314.7813 4.268E-10 6.369E-10 6.928E-10 4.811E-10

315.0000 4.200E-10 6.020E-10 6.816E-10 5.172E-10

315.2188 3.999E-10 5.527E-10 6.444E-10 5.564E-10

315.4375 3.754E-10 5.055E-10 5.917E-10 5.782E-10

315.6563 3.584E-10 4.779E-10 5.404E-10 5.770E-10

315.8750 3.567E-10 4.806E-10 5.057E-10 5.653E-10

316.0938 3.718E-10 5.137E-10 4.979E-10 5.617E-10

316.3125 3.998E-10 5.708E-10 5.238E-10 5.788E-10

316.5313 4.337E-10 6.421E-10 5.842E-10 6.217E-10

316.7500 4.655E-10 7.113E-10 6.665E-10 6.841E-10

316.9688 4.838E-10 7.533E-10 7.401E-10 7.406E-10

317.1875 4.794E-10 7.466E-10 7.705E-10 7.568E-10

317.4063 4.539E-10 6.922E-10 7.450E-10 7.180E-10

317.6250 4.189E-10 6.147E-10 6.793E-10 6.463E-10

317.8438 3.869E-10 5.437E-10 6.030E-10 5.820E-10

318.0625 3.653E-10 4.952E-10 5.430E-10 5.501E-10

318.2813 3.549E-10 4.669E-10 5.131E-10 5.465E-10

318.5000 3.524E-10 4.516E-10 5.125E-10 5.544E-10

318.7188 3.550E-10 4.516E-10 5.300E-10 5.651E-10

318.9375 3.583E-10 4.695E-10 5.501E-10 5.762E-10

319.1563 3.575E-10 4.945E-10 5.633E-10 5.837E-10

319.3750 3.547E-10 5.093E-10 5.704E-10 5.868E-10

319.5938 3.549E-10 5.048E-10 5.717E-10 5.920E-10

319.8125 3.580E-10 4.858E-10 5.605E-10 6.049E-10

320.0313 3.618E-10 4.655E-10 5.362E-10 6.207E-10

320.2500 3.644E-10 4.531E-10 5.088E-10 6.267E-10

320.4688 3.630E-10 4.500E-10 4.872E-10 6.187E-10

320.6875 3.594E-10 4.574E-10 4.731E-10 6.097E-10

320.9063 3.579E-10 4.773E-10 4.654E-10 6.128E-10

321.1250 3.587E-10 5.061E-10 4.637E-10 6.217E-10

321.3438 3.615E-10 5.373E-10 4.681E-10 6.212E-10

321.5625 3.671E-10 5.624E-10 4.741E-10 6.060E-10

321.7813 3.711E-10 5.699E-10 4.759E-10 5.813E-10

322.0000 3.711E-10 5.577E-10 4.779E-10 5.572E-10

322.2188 3.692E-10 5.370E-10 4.879E-10 5.426E-10

322.4375 3.645E-10 5.169E-10 5.016E-10 5.377E-10

322.6563 3.599E-10 5.001E-10 5.132E-10 5.389E-10

322.8750 3.602E-10 4.844E-10 5.244E-10 5.411E-10

323.0938 3.621E-10 4.639E-10 5.350E-10 5.374E-10

323.3125 3.644E-10 4.425E-10 5.439E-10 5.300E-10

323.5313 3.701E-10 4.354E-10 5.474E-10 5.301E-10

323.7500 3.734E-10 4.495E-10 5.328E-10 5.407E-10

323.9688 3.708E-10 4.799E-10 5.012E-10 5.537E-10

324.1875 3.683E-10 5.147E-10 4.805E-10 5.577E-10

324.4063 3.632E-10 5.371E-10 4.946E-10 5.434E-10

324.6250 3.549E-10 5.460E-10 5.458E-10 5.199E-10

324.8438 3.537E-10 5.533E-10 6.164E-10 5.089E-10

325.0625 3.562E-10 5.518E-10 6.705E-10 5.106E-10

325.2813 3.566E-10 5.330E-10 6.862E-10 5.128E-10

325.5000 3.621E-10 5.118E-10 6.750E-10 5.186E-10

325.7188 3.654E-10 4.981E-10 6.483E-10 5.313E-10

325.9375 3.595E-10 4.929E-10 6.102E-10 5.436E-10

326.1563 3.601E-10 5.000E-10 5.755E-10 5.481E-10

326.3750 3.638E-10 5.012E-10 5.509E-10 5.346E-10

326.5938 3.603E-10 4.809E-10 5.416E-10 5.064E-10

326.8125 3.671E-10 4.650E-10 5.596E-10 4.856E-10

327.0313 3.778E-10 4.678E-10 5.822E-10 4.756E-10

327.2500 3.728E-10 4.782E-10 5.842E-10 4.727E-10

327.4688 3.733E-10 4.979E-10 5.880E-10 4.924E-10

327.6875 3.777E-10 5.104E-10 5.948E-10 5.244E-10

327.9063 3.654E-10 4.970E-10 5.802E-10 5.427E-10

328.1250 3.664E-10 4.861E-10 5.640E-10 5.594E-10

328.3438 3.751E-10 4.801E-10 5.506E-10 5.746E-10

328.5625 3.564E-10 4.619E-10 5.158E-10 5.680E-10

328.7813 3.566E-10 4.748E-10 4.920E-10 5.630E-10

329.0000 3.801E-10 5.107E-10 4.881E-10 5.665E-10

329.2188 3.665E-10 5.034E-10 4.715E-10 5.547E-10

329.4375 3.662E-10 4.938E-10 4.844E-10 5.583E-10

329.6563 3.966E-10 5.117E-10 5.273E-10 5.730E-10

329.8750 3.794E-10 4.987E-10 5.219E-10 5.368E-10

330.0938 3.819E-10 4.975E-10 5.151E-10 4.908E-10

330.3125 4.283E-10 5.255E-10 5.484E-10 4.805E-10

330.5313 3.938E-10 4.974E-10 5.578E-10 4.711E-10

330.7500 3.786E-10 5.018E-10 6.063E-10 5.164E-10

330.9688 4.467E-10 5.804E-10 6.775E-10 6.059E-10

331.1875 4.019E-10 5.567E-10 5.924E-10 5.896E-10

331.4063 3.695E-10 5.199E-10 5.061E-10 5.487E-10

331.6250 4.736E-10 5.852E-10 5.674E-10 5.849E-10

331.8438 4.127E-10 5.442E-10 5.257E-10 5.421E-10

332.0625 3.618E-10 5.361E-10 4.908E-10 5.162E-10

332.2813 5.549E-10 7.032E-10 6.521E-10 6.264E-10

332.5000 4.835E-10 6.104E-10 6.201E-10 5.780E-10

332.7188 3.616E-10 4.570E-10 5.494E-10 5.144E-10

332.9375 6.595E-10 6.681E-10 7.913E-10 7.152E-10

333.1563 5.726E-10 6.260E-10 7.076E-10 6.871E-10

333.3750 3.896E-10 5.478E-10 5.291E-10 5.671E-10

333.5938 9.913E-10 1.084E-09 9.757E-10 8.880E-10

333.8125 8.836E-10 9.915E-10 9.090E-10 8.278E-10

334.0313 3.765E-10 5.385E-10 5.572E-10 5.431E-10

334.2500 1.417E-09 1.334E-09 1.397E-09 1.121E-09

334.4688 1.373E-09 1.284E-09 1.341E-09 1.079E-09

334.6875 3.721E-10 4.693E-10 4.941E-10 5.221E-10

334.9063 2.557E-09 2.193E-09 2.140E-09 1.798E-09

335.1250 2.791E-09 2.369E-09 2.331E-09 1.923E-09

335.3438 3.685E-10 4.885E-10 5.250E-10 5.014E-10

335.5625 5.150E-09 4.438E-09 4.356E-09 3.216E-09

335.7813 6.542E-09 5.597E-09 5.464E-09 4.023E-09

336.0000 3.732E-10 4.896E-10 5.647E-10 5.260E-10

336.2188 1.257E-08 9.992E-09 9.948E-09 7.521E-09

336.4375 1.895E-08 1.508E-08 1.486E-08 1.113E-08

336.6563 4.579E-10 5.960E-10 6.056E-10 5.689E-10

336.8750 3.745E-08 3.062E-08 2.995E-08 2.204E-08

337.0938 6.925E-08 5.621E-08 5.501E-08 4.045E-08

337.3125 1.678E-09 1.698E-09 1.612E-09 1.328E-09

337.5313 1.518E-07 1.210E-07 1.175E-07 8.647E-08

337.7500 3.619E-07 2.890E-07 2.807E-07 2.066E-07

337.9688 1.803E-08 1.454E-08 1.367E-08 1.040E-08

338.1875 1.033E-06 8.268E-07 8.125E-07 5.936E-07

338.4063 3.646E-06 2.916E-06 2.860E-06 2.092E-06

338.6250 4.420E-07 3.534E-07 3.494E-07 2.542E-07

338.8438 2.371E-05 1.897E-05 1.850E-05 1.359E-05

339.0625 2.541E-04 2.033E-04 1.984E-04 1.456E-04

339.2813 1.011E-03 8.090E-04 7.900E-04 5.795E-04

339.5000 2.440E-03 1.952E-03 1.906E-03 1.398E-03

339.7188 4.117E-03 3.293E-03 3.216E-03 2.359E-03

339.9375 5.142E-03 4.113E-03 4.017E-03 2.946E-03

340.1563 4.851E-03 3.880E-03 3.790E-03 2.780E-03

340.3750 3.438E-03 2.750E-03 2.686E-03 1.970E-03

340.5938 1.771E-03 1.417E-03 1.383E-03 1.015E-03

340.8125 6.096E-04 4.877E-04 4.762E-04 3.493E-04

341.0313 1.119E-04 8.950E-05 8.737E-05 6.410E-05

341.2500 3.498E-06 2.803E-06 2.729E-06 2.006E-06

341.4688 2.619E-06 2.091E-06 2.049E-06 1.499E-06

341.6875 2.816E-06 2.249E-06 2.203E-06 1.612E-06

341.9063 2.318E-07 1.844E-07 1.818E-07 1.325E-07

342.1250 1.937E-07 1.562E-07 1.516E-07 1.121E-07

342.3438 3.348E-07 2.694E-07 2.622E-07 1.933E-07

342.5625 4.281E-08 3.486E-08 3.392E-08 2.529E-08

342.7813 3.055E-08 2.417E-08 2.394E-08 1.748E-08

343.0000 7.175E-08 5.706E-08 5.608E-08 4.098E-08

343.2188 1.256E-08 1.007E-08 1.002E-08 7.353E-09

343.4375 7.276E-09 6.027E-09 5.941E-09 4.460E-09

343.6563 2.160E-08 1.742E-08 1.706E-08 1.261E-08

343.8750 4.999E-09 4.129E-09 4.106E-09 3.088E-09

344.0938 2.271E-09 2.017E-09 2.064E-09 1.627E-09

344.3125 7.968E-09 6.598E-09 6.545E-09 4.997E-09

344.5313 2.360E-09 2.056E-09 2.139E-09 1.754E-09

344.7500 1.025E-09 9.263E-10 1.111E-09 8.007E-10

344.9688 3.651E-09 3.015E-09 3.202E-09 2.149E-09

345.1875 1.427E-09 1.271E-09 1.433E-09 9.489E-10

345.4063 5.748E-10 6.389E-10 6.802E-10 6.764E-10

345.6250 1.819E-09 1.666E-09 1.611E-09 1.490E-09

345.8438 8.950E-10 9.176E-10 9.050E-10 8.909E-10

346.0625 4.541E-10 5.221E-10 5.684E-10 5.548E-10

346.2813 1.143E-09 1.032E-09 1.097E-09 9.536E-10

346.5000 7.063E-10 6.761E-10 7.738E-10 7.553E-10

346.7188 3.912E-10 4.582E-10 5.649E-10 6.133E-10

346.9375 7.556E-10 7.980E-10 8.533E-10 8.319E-10

347.1563 5.620E-10 6.605E-10 6.600E-10 7.151E-10

347.3750 3.849E-10 4.818E-10 4.756E-10 6.137E-10

347.5938 6.059E-10 6.059E-10 6.364E-10 7.503E-10

347.8125 5.067E-10 5.230E-10 5.833E-10 6.939E-10

348.0313 3.816E-10 4.700E-10 5.225E-10 6.101E-10

348.2500 5.151E-10 6.107E-10 6.499E-10 6.832E-10

348.4688 4.710E-10 5.569E-10 6.080E-10 6.586E-10

348.6875 3.842E-10 4.425E-10 5.046E-10 5.810E-10

348.9063 4.568E-10 4.725E-10 5.307E-10 5.688E-10

349.1250 4.253E-10 4.509E-10 5.279E-10 5.179E-10

349.3438 3.585E-10 4.212E-10 5.586E-10 4.888E-10

349.5625 4.030E-10 4.820E-10 6.885E-10 5.368E-10

349.7813 3.895E-10 4.869E-10 7.353E-10 5.341E-10

350.0000 3.486E-10 4.614E-10 7.200E-10 4.974E-10

**Data plotted in FIG 2(b)**

Frequency PBS without cells Cells in LB, -41.4 min Cells in LB, -5.3 min Cells in LB, 7.2 min

0.2188 2.931E-06 8.260E-06 3.587E-06 6.770E-06

0.4375 2.123E-06 5.591E-06 2.511E-06 4.547E-06

0.6563 1.233E-06 2.851E-06 1.372E-06 2.283E-06

0.8750 5.729E-07 1.063E-06 5.837E-07 8.302E-07

1.0938 2.174E-07 2.956E-07 2.051E-07 2.283E-07

1.3125 7.351E-08 8.603E-08 7.608E-08 7.786E-08

1.5313 2.651E-08 4.274E-08 4.026E-08 5.155E-08

1.7500 1.203E-08 2.471E-08 2.837E-08 3.874E-08

1.9688 7.571E-09 1.593E-08 2.413E-08 3.129E-08

2.1875 6.287E-09 1.346E-08 2.298E-08 2.837E-08

2.4063 5.628E-09 1.195E-08 2.196E-08 2.580E-08

2.6250 5.043E-09 1.070E-08 2.058E-08 2.337E-08

2.8438 4.635E-09 1.014E-08 1.925E-08 2.180E-08

3.0625 4.354E-09 9.692E-09 1.802E-08 2.082E-08

3.2813 4.089E-09 9.127E-09 1.678E-08 2.015E-08

3.5000 3.807E-09 8.618E-09 1.544E-08 1.948E-08

3.7188 3.527E-09 8.304E-09 1.406E-08 1.838E-08

3.9375 3.299E-09 8.310E-09 1.285E-08 1.682E-08

4.1563 3.158E-09 8.557E-09 1.201E-08 1.526E-08

4.3750 3.087E-09 8.696E-09 1.161E-08 1.410E-08

4.5938 3.031E-09 8.446E-09 1.150E-08 1.343E-08

4.8125 2.950E-09 7.829E-09 1.148E-08 1.307E-08

5.0313 2.845E-09 7.087E-09 1.138E-08 1.289E-08

5.2500 2.746E-09 6.458E-09 1.114E-08 1.284E-08

5.4688 2.659E-09 6.008E-09 1.078E-08 1.279E-08

5.6875 2.564E-09 5.677E-09 1.032E-08 1.259E-08

5.9063 2.447E-09 5.440E-09 9.835E-09 1.215E-08

6.1250 2.325E-09 5.328E-09 9.438E-09 1.153E-08

6.3438 2.227E-09 5.337E-09 9.199E-09 1.078E-08

6.5625 2.168E-09 5.397E-09 9.041E-09 9.937E-09

6.7813 2.135E-09 5.421E-09 8.826E-09 9.099E-09

7.0000 2.103E-09 5.355E-09 8.486E-09 8.474E-09

7.2188 2.056E-09 5.203E-09 8.061E-09 8.231E-09

7.4375 1.999E-09 5.003E-09 7.649E-09 8.303E-09

7.6563 1.946E-09 4.787E-09 7.339E-09 8.405E-09

7.8750 1.910E-09 4.570E-09 7.160E-09 8.316E-09

8.0938 1.887E-09 4.386E-09 7.057E-09 8.102E-09

8.3125 1.867E-09 4.270E-09 6.945E-09 7.978E-09

8.5313 1.836E-09 4.211E-09 6.786E-09 8.019E-09

8.7500 1.789E-09 4.141E-09 6.599E-09 8.092E-09

8.9688 1.734E-09 4.016E-09 6.412E-09 8.068E-09

9.1875 1.686E-09 3.874E-09 6.227E-09 7.966E-09

9.4063 1.658E-09 3.790E-09 6.037E-09 7.877E-09

9.6250 1.652E-09 3.774E-09 5.839E-09 7.805E-09

9.8438 1.654E-09 3.755E-09 5.638E-09 7.654E-09

10.0625 1.644E-09 3.662E-09 5.445E-09 7.350E-09

10.2813 1.610E-09 3.507E-09 5.260E-09 6.925E-09

10.5000 1.559E-09 3.368E-09 5.071E-09 6.493E-09

10.7188 1.505E-09 3.318E-09 4.879E-09 6.170E-09

10.9375 1.464E-09 3.356E-09 4.725E-09 5.998E-09

11.1563 1.444E-09 3.418E-09 4.667E-09 5.919E-09

11.3750 1.441E-09 3.427E-09 4.710E-09 5.815E-09

11.5938 1.445E-09 3.358E-09 4.778E-09 5.609E-09

11.8125 1.444E-09 3.239E-09 4.778E-09 5.347E-09

12.0313 1.435E-09 3.111E-09 4.702E-09 5.163E-09

12.2500 1.426E-09 2.999E-09 4.657E-09 5.143E-09

12.4688 1.416E-09 2.916E-09 4.758E-09 5.229E-09

12.6875 1.399E-09 2.865E-09 4.971E-09 5.276E-09

12.9063 1.370E-09 2.825E-09 5.117E-09 5.205E-09

13.1250 1.338E-09 2.760E-09 5.050E-09 5.068E-09

13.3438 1.319E-09 2.674E-09 4.800E-09 4.946E-09

13.5625 1.320E-09 2.639E-09 4.509E-09 4.844E-09

13.7813 1.333E-09 2.720E-09 4.281E-09 4.723E-09

14.0000 1.341E-09 2.887E-09 4.133E-09 4.616E-09

14.2188 1.334E-09 3.027E-09 4.055E-09 4.616E-09

14.4375 1.312E-09 3.052E-09 4.033E-09 4.733E-09

14.6563 1.282E-09 2.968E-09 4.021E-09 4.846E-09

14.8750 1.253E-09 2.838E-09 3.978E-09 4.825E-09

15.0938 1.231E-09 2.708E-09 3.931E-09 4.661E-09

15.3125 1.219E-09 2.579E-09 3.936E-09 4.449E-09

15.5313 1.215E-09 2.445E-09 3.984E-09 4.280E-09

15.7500 1.214E-09 2.320E-09 3.988E-09 4.187E-09

15.9688 1.211E-09 2.238E-09 3.891E-09 4.169E-09

16.1875 1.199E-09 2.227E-09 3.752E-09 4.226E-09

16.4063 1.181E-09 2.275E-09 3.683E-09 4.363E-09

16.6250 1.163E-09 2.329E-09 3.732E-09 4.562E-09

16.8438 1.148E-09 2.339E-09 3.841E-09 4.760E-09

17.0625 1.136E-09 2.303E-09 3.913E-09 4.863E-09

17.2813 1.131E-09 2.265E-09 3.894E-09 4.800E-09

17.5000 1.135E-09 2.266E-09 3.792E-09 4.575E-09

17.7188 1.143E-09 2.308E-09 3.644E-09 4.277E-09

17.9375 1.141E-09 2.351E-09 3.494E-09 4.026E-09

18.1563 1.118E-09 2.351E-09 3.385E-09 3.895E-09

18.3750 1.082E-09 2.290E-09 3.347E-09 3.855E-09

18.5938 1.054E-09 2.203E-09 3.372E-09 3.813E-09

18.8125 1.051E-09 2.151E-09 3.414E-09 3.707E-09

19.0313 1.073E-09 2.161E-09 3.427E-09 3.563E-09

19.2500 1.106E-09 2.199E-09 3.405E-09 3.447E-09

19.4688 1.131E-09 2.211E-09 3.372E-09 3.388E-09

19.6875 1.136E-09 2.192E-09 3.344E-09 3.361E-09

19.9063 1.118E-09 2.180E-09 3.318E-09 3.346E-09

20.1250 1.087E-09 2.197E-09 3.285E-09 3.362E-09

20.3438 1.053E-09 2.214E-09 3.239E-09 3.420E-09

20.5625 1.025E-09 2.188E-09 3.194E-09 3.486E-09

20.7813 1.002E-09 2.117E-09 3.173E-09 3.510E-09

21.0000 9.847E-10 2.034E-09 3.178E-09 3.485E-09

21.2188 9.730E-10 1.960E-09 3.177E-09 3.463E-09

21.4375 9.721E-10 1.887E-09 3.124E-09 3.491E-09

21.6563 9.834E-10 1.801E-09 3.020E-09 3.568E-09

21.8750 9.993E-10 1.720E-09 2.913E-09 3.641E-09

22.0938 1.008E-09 1.685E-09 2.851E-09 3.648E-09

22.3125 1.006E-09 1.725E-09 2.830E-09 3.574E-09

22.5313 9.974E-10 1.830E-09 2.824E-09 3.477E-09

22.7500 9.891E-10 1.958E-09 2.818E-09 3.454E-09

22.9688 9.816E-10 2.062E-09 2.810E-09 3.554E-09

23.1875 9.732E-10 2.110E-09 2.800E-09 3.713E-09

23.4063 9.652E-10 2.105E-09 2.790E-09 3.808E-09

23.6250 9.613E-10 2.077E-09 2.810E-09 3.759E-09

23.8438 9.618E-10 2.061E-09 2.891E-09 3.597E-09

24.0625 9.631E-10 2.072E-09 3.022E-09 3.412E-09

24.2813 9.631E-10 2.091E-09 3.133E-09 3.279E-09

24.5000 9.627E-10 2.092E-09 3.150E-09 3.224E-09

24.7188 9.622E-10 2.067E-09 3.063E-09 3.235E-09

24.9375 9.582E-10 2.036E-09 2.939E-09 3.263E-09

25.1563 9.479E-10 2.022E-09 2.852E-09 3.237E-09

25.3750 9.337E-10 2.026E-09 2.820E-09 3.121E-09

25.5938 9.220E-10 2.022E-09 2.806E-09 2.951E-09

25.8125 9.156E-10 1.977E-09 2.765E-09 2.806E-09

26.0313 9.106E-10 1.884E-09 2.681E-09 2.734E-09

26.2500 9.008E-10 1.767E-09 2.553E-09 2.718E-09

26.4688 8.851E-10 1.666E-09 2.398E-09 2.725E-09

26.6875 8.702E-10 1.613E-09 2.251E-09 2.740E-09

26.9063 8.642E-10 1.615E-09 2.154E-09 2.758E-09

27.1250 8.703E-10 1.664E-09 2.119E-09 2.763E-09

27.3438 8.843E-10 1.741E-09 2.109E-09 2.753E-09

27.5625 8.997E-10 1.820E-09 2.075E-09 2.753E-09

27.7813 9.119E-10 1.871E-09 2.003E-09 2.791E-09

28.0000 9.194E-10 1.869E-09 1.927E-09 2.865E-09

28.2188 9.220E-10 1.806E-09 1.896E-09 2.933E-09

28.4375 9.183E-10 1.708E-09 1.924E-09 2.942E-09

28.6563 9.072E-10 1.608E-09 1.989E-09 2.860E-09

28.8750 8.914E-10 1.531E-09 2.055E-09 2.702E-09

29.0938 8.770E-10 1.476E-09 2.091E-09 2.532E-09

29.3125 8.692E-10 1.437E-09 2.093E-09 2.420E-09

29.5313 8.667E-10 1.418E-09 2.076E-09 2.385E-09

29.7500 8.636E-10 1.424E-09 2.071E-09 2.383E-09

29.9688 8.554E-10 1.442E-09 2.092E-09 2.364E-09

30.1875 8.443E-10 1.455E-09 2.137E-09 2.329E-09

30.4063 8.360E-10 1.457E-09 2.193E-09 2.319E-09

30.6250 8.336E-10 1.463E-09 2.247E-09 2.344E-09

30.8438 8.351E-10 1.491E-09 2.282E-09 2.358E-09

31.0625 8.371E-10 1.538E-09 2.289E-09 2.319E-09

31.2813 8.376E-10 1.584E-09 2.285E-09 2.253E-09

31.5000 8.366E-10 1.598E-09 2.301E-09 2.232E-09

31.7188 8.342E-10 1.563E-09 2.341E-09 2.289E-09

31.9375 8.321E-10 1.496E-09 2.363E-09 2.387E-09

32.1563 8.334E-10 1.434E-09 2.329E-09 2.471E-09

32.3750 8.386E-10 1.400E-09 2.245E-09 2.512E-09

32.5938 8.439E-10 1.388E-09 2.156E-09 2.512E-09

32.8125 8.433E-10 1.383E-09 2.092E-09 2.481E-09

33.0313 8.358E-10 1.383E-09 2.049E-09 2.435E-09

33.2500 8.271E-10 1.397E-09 2.016E-09 2.397E-09

33.4688 8.245E-10 1.429E-09 2.003E-09 2.400E-09

33.6875 8.297E-10 1.483E-09 2.027E-09 2.452E-09

33.9063 8.380E-10 1.558E-09 2.084E-09 2.522E-09

34.1250 8.421E-10 1.644E-09 2.144E-09 2.555E-09

34.3438 8.374E-10 1.710E-09 2.178E-09 2.525E-09

34.5625 8.250E-10 1.722E-09 2.167E-09 2.456E-09

34.7813 8.109E-10 1.672E-09 2.115E-09 2.391E-09

35.0000 8.022E-10 1.586E-09 2.045E-09 2.348E-09

35.2188 8.032E-10 1.493E-09 2.000E-09 2.311E-09

35.4375 8.122E-10 1.416E-09 2.009E-09 2.268E-09

35.6563 8.237E-10 1.367E-09 2.058E-09 2.226E-09

35.8750 8.322E-10 1.357E-09 2.105E-09 2.203E-09

36.0938 8.349E-10 1.387E-09 2.122E-09 2.201E-09

36.3125 8.296E-10 1.442E-09 2.123E-09 2.207E-09

36.5313 8.157E-10 1.499E-09 2.134E-09 2.200E-09

36.7500 7.966E-10 1.536E-09 2.158E-09 2.167E-09

36.9688 7.801E-10 1.543E-09 2.179E-09 2.106E-09

37.1875 7.722E-10 1.517E-09 2.187E-09 2.031E-09

37.4063 7.729E-10 1.467E-09 2.190E-09 1.970E-09

37.6250 7.778E-10 1.413E-09 2.194E-09 1.953E-09

37.8438 7.830E-10 1.375E-09 2.189E-09 1.995E-09

38.0625 7.876E-10 1.364E-09 2.160E-09 2.075E-09

38.2813 7.916E-10 1.374E-09 2.106E-09 2.147E-09

38.5000 7.938E-10 1.391E-09 2.044E-09 2.184E-09

38.7188 7.916E-10 1.392E-09 1.985E-09 2.189E-09

38.9375 7.827E-10 1.360E-09 1.930E-09 2.178E-09

39.1563 7.679E-10 1.297E-09 1.874E-09 2.161E-09

39.3750 7.513E-10 1.226E-09 1.823E-09 2.132E-09

39.5938 7.393E-10 1.182E-09 1.802E-09 2.091E-09

39.8125 7.354E-10 1.183E-09 1.823E-09 2.053E-09

40.0313 7.387E-10 1.217E-09 1.865E-09 2.035E-09

40.2500 7.447E-10 1.246E-09 1.879E-09 2.047E-09

40.4688 7.490E-10 1.239E-09 1.834E-09 2.081E-09

40.6875 7.504E-10 1.203E-09 1.759E-09 2.112E-09

40.9063 7.495E-10 1.181E-09 1.717E-09 2.116E-09

41.1250 7.469E-10 1.203E-09 1.747E-09 2.088E-09

41.3438 7.433E-10 1.263E-09 1.830E-09 2.051E-09

41.5625 7.402E-10 1.319E-09 1.906E-09 2.034E-09

41.7813 7.396E-10 1.341E-09 1.929E-09 2.044E-09

42.0000 7.420E-10 1.326E-09 1.884E-09 2.066E-09

42.2188 7.457E-10 1.294E-09 1.793E-09 2.085E-09

42.4375 7.486E-10 1.268E-09 1.698E-09 2.101E-09

42.6563 7.499E-10 1.259E-09 1.642E-09 2.117E-09

42.8750 7.491E-10 1.269E-09 1.636E-09 2.118E-09

43.0938 7.446E-10 1.287E-09 1.662E-09 2.098E-09

43.3125 7.375E-10 1.295E-09 1.692E-09 2.083E-09

43.5313 7.335E-10 1.280E-09 1.719E-09 2.106E-09

43.7500 7.285E-10 1.250E-09 1.748E-09 2.155E-09

43.9688 7.256E-10 1.232E-09 1.771E-09 2.171E-09

44.1875 8.513E-10 1.250E-09 1.770E-09 2.111E-09

44.4063 1.456E-09 1.306E-09 1.754E-09 2.011E-09

44.6250 2.899E-09 1.447E-09 1.792E-09 2.001E-09

44.8438 5.017E-09 1.823E-09 1.998E-09 2.236E-09

45.0625 6.872E-09 2.582E-09 2.452E-09 2.774E-09

45.2813 7.345E-09 3.603E-09 3.062E-09 3.445E-09

45.5000 6.127E-09 4.403E-09 3.536E-09 3.899E-09

45.7188 4.011E-09 4.474E-09 3.576E-09 3.855E-09

45.9375 2.141E-09 3.749E-09 3.146E-09 3.359E-09

46.1563 1.103E-09 2.678E-09 2.522E-09 2.742E-09

46.3750 7.510E-10 1.812E-09 2.045E-09 2.317E-09

46.5938 6.911E-10 1.383E-09 1.847E-09 2.147E-09

46.8125 6.866E-10 1.274E-09 1.837E-09 2.098E-09

47.0313 6.876E-10 1.275E-09 1.878E-09 2.052E-09

47.2500 6.982E-10 1.275E-09 1.909E-09 2.008E-09

47.4688 7.140E-10 1.260E-09 1.926E-09 2.014E-09

47.6875 7.278E-10 1.245E-09 1.915E-09 2.070E-09

47.9063 7.349E-10 1.231E-09 1.845E-09 2.122E-09

48.1250 7.321E-10 1.207E-09 1.719E-09 2.121E-09

48.3438 7.202E-10 1.166E-09 1.596E-09 2.055E-09

48.5625 7.051E-10 1.107E-09 1.548E-09 1.948E-09

48.7813 6.938E-10 1.045E-09 1.592E-09 1.835E-09

49.0000 6.914E-10 9.981E-10 1.676E-09 1.745E-09

49.2188 6.989E-10 9.821E-10 1.727E-09 1.696E-09

49.4375 7.133E-10 9.921E-10 1.723E-09 1.689E-09

49.6563 7.280E-10 1.008E-09 1.691E-09 1.713E-09

49.8750 7.367E-10 1.008E-09 1.673E-09 1.750E-09

50.0938 7.366E-10 9.837E-10 1.679E-09 1.787E-09

50.3125 7.302E-10 9.463E-10 1.695E-09 1.815E-09

50.5313 7.232E-10 9.258E-10 1.699E-09 1.833E-09

50.7500 7.202E-10 9.539E-10 1.682E-09 1.843E-09

50.9688 7.224E-10 1.035E-09 1.651E-09 1.845E-09

51.1875 7.280E-10 1.132E-09 1.625E-09 1.849E-09

51.4063 7.346E-10 1.195E-09 1.621E-09 1.869E-09

51.6250 7.416E-10 1.206E-09 1.641E-09 1.913E-09

51.8438 7.524E-10 1.190E-09 1.678E-09 1.969E-09

52.0625 7.731E-10 1.196E-09 1.724E-09 2.011E-09

52.2813 8.056E-10 1.249E-09 1.773E-09 2.026E-09

52.5000 8.397E-10 1.330E-09 1.818E-09 2.019E-09

52.7188 8.557E-10 1.387E-09 1.851E-09 1.997E-09

52.9375 8.406E-10 1.376E-09 1.863E-09 1.959E-09

53.1563 8.006E-10 1.295E-09 1.852E-09 1.893E-09

53.3750 7.566E-10 1.185E-09 1.819E-09 1.792E-09

53.5938 7.264E-10 1.096E-09 1.760E-09 1.666E-09

53.8125 7.141E-10 1.053E-09 1.671E-09 1.550E-09

54.0313 7.127E-10 1.055E-09 1.564E-09 1.490E-09

54.2500 7.141E-10 1.086E-09 1.484E-09 1.510E-09

54.4688 7.143E-10 1.125E-09 1.483E-09 1.584E-09

54.6875 7.133E-10 1.148E-09 1.573E-09 1.659E-09

54.9063 7.124E-10 1.133E-09 1.703E-09 1.705E-09

55.1250 7.119E-10 1.087E-09 1.794E-09 1.732E-09

55.3438 7.109E-10 1.047E-09 1.799E-09 1.765E-09

55.5625 7.100E-10 1.044E-09 1.733E-09 1.814E-09

55.7813 7.107E-10 1.081E-09 1.645E-09 1.862E-09

56.0000 7.128E-10 1.132E-09 1.579E-09 1.884E-09

56.2188 7.124E-10 1.170E-09 1.559E-09 1.866E-09

56.4375 7.050E-10 1.186E-09 1.586E-09 1.829E-09

56.6563 6.908E-10 1.178E-09 1.648E-09 1.813E-09

56.8750 6.756E-10 1.147E-09 1.724E-09 1.826E-09

57.0938 6.653E-10 1.095E-09 1.781E-09 1.830E-09

57.3125 6.628E-10 1.041E-09 1.791E-09 1.798E-09

57.5313 6.717E-10 1.014E-09 1.739E-09 1.755E-09

57.7500 6.886E-10 1.025E-09 1.627E-09 1.746E-09

57.9688 7.059E-10 1.064E-09 1.485E-09 1.810E-09

58.1875 7.423E-10 1.150E-09 1.400E-09 1.939E-09

58.4063 8.056E-10 1.272E-09 1.409E-09 1.939E-09

58.6250 8.313E-10 1.307E-09 1.360E-09 1.904E-09

58.8438 1.091E-09 1.766E-09 1.637E-09 3.977E-09

59.0625 3.165E-09 5.379E-09 4.851E-09 1.333E-08

59.2813 9.885E-09 1.679E-08 1.578E-08 3.437E-08

59.5000 2.256E-08 3.780E-08 3.672E-08 6.330E-08

59.7188 3.745E-08 6.183E-08 6.154E-08 8.616E-08

59.9375 4.659E-08 7.578E-08 7.696E-08 8.855E-08

60.1563 4.406E-08 7.052E-08 7.307E-08 6.874E-08

60.3750 3.155E-08 4.955E-08 5.256E-08 3.945E-08

60.5938 1.672E-08 2.562E-08 2.810E-08 1.600E-08

60.8125 6.327E-09 9.363E-09 1.090E-08 4.545E-09

61.0313 1.805E-09 2.588E-09 3.377E-09 1.678E-09

61.2500 7.575E-10 1.175E-09 1.615E-09 1.724E-09

61.4688 6.981E-10 1.150E-09 1.521E-09 1.854E-09

61.6875 6.758E-10 1.102E-09 1.517E-09 1.776E-09

61.9063 6.459E-10 1.028E-09 1.499E-09 1.716E-09

62.1250 6.484E-10 1.018E-09 1.513E-09 1.684E-09

62.3438 6.568E-10 1.040E-09 1.513E-09 1.654E-09

62.5625 6.620E-10 1.068E-09 1.496E-09 1.639E-09

62.7813 6.686E-10 1.088E-09 1.489E-09 1.632E-09

63.0000 6.734E-10 1.078E-09 1.504E-09 1.617E-09

63.2188 6.737E-10 1.038E-09 1.547E-09 1.601E-09

63.4375 6.724E-10 9.873E-10 1.608E-09 1.592E-09

63.6563 6.708E-10 9.482E-10 1.670E-09 1.587E-09

63.8750 6.672E-10 9.400E-10 1.716E-09 1.582E-09

64.0938 6.597E-10 9.687E-10 1.731E-09 1.585E-09

64.3125 6.514E-10 1.017E-09 1.706E-09 1.619E-09

64.5313 6.494E-10 1.052E-09 1.656E-09 1.693E-09

64.7500 6.568E-10 1.049E-09 1.609E-09 1.785E-09

64.9688 6.678E-10 1.015E-09 1.576E-09 1.843E-09

65.1875 6.737E-10 9.845E-10 1.530E-09 1.823E-09

65.4063 6.704E-10 9.904E-10 1.443E-09 1.726E-09

65.6250 6.607E-10 1.032E-09 1.326E-09 1.608E-09

65.8438 6.496E-10 1.074E-09 1.232E-09 1.543E-09

66.0625 6.409E-10 1.082E-09 1.214E-09 1.563E-09

66.2813 6.360E-10 1.048E-09 1.265E-09 1.645E-09

66.5000 6.345E-10 9.964E-10 1.326E-09 1.726E-09

66.7188 6.348E-10 9.524E-10 1.336E-09 1.757E-09

66.9375 6.356E-10 9.236E-10 1.299E-09 1.720E-09

67.1563 6.373E-10 9.076E-10 1.274E-09 1.630E-09

67.3750 6.410E-10 9.094E-10 1.317E-09 1.523E-09

67.5938 6.461E-10 9.389E-10 1.419E-09 1.443E-09

67.8125 6.492E-10 9.924E-10 1.519E-09 1.422E-09

68.0313 6.472E-10 1.042E-09 1.566E-09 1.456E-09

68.2500 6.404E-10 1.058E-09 1.562E-09 1.506E-09

68.4688 6.324E-10 1.040E-09 1.533E-09 1.536E-09

68.6875 6.270E-10 1.025E-09 1.494E-09 1.535E-09

68.9063 6.244E-10 1.051E-09 1.447E-09 1.517E-09

69.1250 6.231E-10 1.114E-09 1.398E-09 1.501E-09

69.3438 6.224E-10 1.169E-09 1.361E-09 1.488E-09

69.5625 6.232E-10 1.170E-09 1.341E-09 1.465E-09

69.7813 6.259E-10 1.112E-09 1.336E-09 1.428E-09

70.0000 6.283E-10 1.026E-09 1.351E-09 1.390E-09

70.2188 6.282E-10 9.554E-10 1.398E-09 1.372E-09

70.4375 6.266E-10 9.259E-10 1.476E-09 1.381E-09

70.6563 6.260E-10 9.403E-10 1.554E-09 1.397E-09

70.8750 6.271E-10 9.796E-10 1.588E-09 1.404E-09

71.0938 6.275E-10 1.015E-09 1.561E-09 1.408E-09

71.3125 6.247E-10 1.022E-09 1.489E-09 1.428E-09

71.5313 6.195E-10 9.947E-10 1.408E-09 1.474E-09

71.7500 6.159E-10 9.463E-10 1.336E-09 1.527E-09

71.9688 6.174E-10 8.998E-10 1.274E-09 1.567E-09

72.1875 6.230E-10 8.756E-10 1.222E-09 1.587E-09

72.4063 6.284E-10 8.799E-10 1.190E-09 1.601E-09

72.6250 6.305E-10 9.034E-10 1.190E-09 1.615E-09

72.8438 6.299E-10 9.310E-10 1.223E-09 1.606E-09

73.0625 6.288E-10 9.562E-10 1.276E-09 1.542E-09

73.2813 6.267E-10 9.817E-10 1.331E-09 1.426E-09

73.5000 6.231E-10 1.010E-09 1.381E-09 1.312E-09

73.7188 6.203E-10 1.036E-09 1.427E-09 1.262E-09

73.9375 6.222E-10 1.053E-09 1.471E-09 1.291E-09

74.1563 6.298E-10 1.060E-09 1.499E-09 1.358E-09

74.3750 6.395E-10 1.063E-09 1.500E-09 1.409E-09

74.5938 6.471E-10 1.060E-09 1.475E-09 1.423E-09

74.8125 6.502E-10 1.046E-09 1.441E-09 1.407E-09

75.0313 6.485E-10 1.019E-09 1.407E-09 1.384E-09

75.2500 6.434E-10 9.890E-10 1.359E-09 1.375E-09

75.4688 6.379E-10 9.716E-10 1.289E-09 1.391E-09

75.6875 6.343E-10 9.723E-10 1.217E-09 1.423E-09

75.9063 6.327E-10 9.811E-10 1.184E-09 1.445E-09

76.1250 6.311E-10 9.861E-10 1.209E-09 1.443E-09

76.3438 6.281E-10 9.876E-10 1.268E-09 1.437E-09

76.5625 6.229E-10 9.931E-10 1.315E-09 1.453E-09

76.7813 6.150E-10 1.001E-09 1.329E-09 1.488E-09

77.0000 6.049E-10 1.001E-09 1.324E-09 1.511E-09

77.2188 5.951E-10 9.865E-10 1.329E-09 1.500E-09

77.4375 5.891E-10 9.739E-10 1.351E-09 1.458E-09

77.6563 5.890E-10 9.882E-10 1.376E-09 1.401E-09

77.8750 5.948E-10 1.040E-09 1.380E-09 1.338E-09

78.0938 6.035E-10 1.110E-09 1.348E-09 1.276E-09

78.3125 6.108E-10 1.161E-09 1.285E-09 1.229E-09

78.5313 6.123E-10 1.160E-09 1.217E-09 1.213E-09

78.7500 6.073E-10 1.103E-09 1.180E-09 1.229E-09

78.9688 5.998E-10 1.015E-09 1.192E-09 1.269E-09

79.1875 5.951E-10 9.347E-10 1.235E-09 1.325E-09

79.4063 5.950E-10 8.799E-10 1.272E-09 1.384E-09

79.6250 5.975E-10 8.471E-10 1.272E-09 1.430E-09

79.8438 6.002E-10 8.284E-10 1.234E-09 1.451E-09

80.0625 6.007E-10 8.255E-10 1.175E-09 1.453E-09

80.2813 5.965E-10 8.420E-10 1.115E-09 1.442E-09

80.5000 5.869E-10 8.714E-10 1.071E-09 1.418E-09

80.7188 5.764E-10 8.999E-10 1.056E-09 1.375E-09

80.9375 5.724E-10 9.193E-10 1.076E-09 1.322E-09

81.1563 5.773E-10 9.322E-10 1.120E-09 1.288E-09

81.3750 5.856E-10 9.469E-10 1.162E-09 1.294E-09

81.5938 5.902E-10 9.682E-10 1.183E-09 1.338E-09

81.8125 5.901E-10 9.907E-10 1.192E-09 1.392E-09

82.0313 5.884E-10 1.004E-09 1.213E-09 1.435E-09

82.2500 5.860E-10 1.003E-09 1.263E-09 1.462E-09

82.4688 5.798E-10 9.903E-10 1.329E-09 1.477E-09

82.6875 5.679E-10 9.730E-10 1.377E-09 1.480E-09

82.9063 5.541E-10 9.532E-10 1.378E-09 1.460E-09

83.1250 5.451E-10 9.333E-10 1.327E-09 1.410E-09

83.3438 5.454E-10 9.179E-10 1.251E-09 1.341E-09

83.5625 5.541E-10 9.090E-10 1.192E-09 1.281E-09

83.7813 5.668E-10 9.040E-10 1.173E-09 1.258E-09

84.0000 5.803E-10 9.036E-10 1.189E-09 1.282E-09

84.2188 5.930E-10 9.137E-10 1.222E-09 1.335E-09

84.4375 6.038E-10 9.345E-10 1.254E-09 1.383E-09

84.6563 6.090E-10 9.502E-10 1.275E-09 1.399E-09

84.8750 6.059E-10 9.399E-10 1.269E-09 1.378E-09

85.0938 5.957E-10 8.982E-10 1.225E-09 1.340E-09

85.3125 5.840E-10 8.414E-10 1.154E-09 1.316E-09

85.5313 5.760E-10 7.960E-10 1.088E-09 1.318E-09

85.7500 5.729E-10 7.804E-10 1.062E-09 1.337E-09

85.9688 5.728E-10 7.947E-10 1.086E-09 1.344E-09

86.1875 5.739E-10 8.223E-10 1.146E-09 1.321E-09

86.4063 5.760E-10 8.433E-10 1.210E-09 1.279E-09

86.6250 5.791E-10 8.476E-10 1.243E-09 1.252E-09

86.8438 5.818E-10 8.398E-10 1.232E-09 1.266E-09

87.0625 5.827E-10 8.351E-10 1.193E-09 1.307E-09

87.2813 5.817E-10 8.515E-10 1.160E-09 1.341E-09

87.5000 5.817E-10 8.957E-10 1.147E-09 1.347E-09

87.7188 5.861E-10 9.522E-10 1.142E-09 1.335E-09

87.9375 5.948E-10 9.899E-10 1.119E-09 1.319E-09

88.1563 6.008E-10 9.895E-10 1.078E-09 1.295E-09

88.3750 5.964E-10 9.617E-10 1.044E-09 1.259E-09

88.5938 5.869E-10 9.353E-10 1.044E-09 1.233E-09

88.8125 5.874E-10 9.291E-10 1.081E-09 1.250E-09

89.0313 5.999E-10 9.401E-10 1.138E-09 1.314E-09

89.2500 6.357E-10 9.538E-10 1.187E-09 1.383E-09

89.4688 8.305E-10 9.584E-10 1.212E-09 1.416E-09

89.6875 1.498E-09 9.452E-10 1.209E-09 1.403E-09

89.9063 2.876E-09 9.078E-10 1.192E-09 1.369E-09

90.1250 4.676E-09 8.713E-10 1.188E-09 1.363E-09

90.3438 6.017E-09 9.447E-10 1.262E-09 1.447E-09

90.5625 6.061E-09 1.298E-09 1.492E-09 1.672E-09

90.7813 4.778E-09 1.994E-09 1.891E-09 1.999E-09

91.0000 2.974E-09 2.808E-09 2.321E-09 2.274E-09

91.2188 1.549E-09 3.300E-09 2.544E-09 2.325E-09

91.4375 8.377E-10 3.161E-09 2.422E-09 2.119E-09

91.6563 6.292E-10 2.491E-09 2.053E-09 1.797E-09

91.8750 6.011E-10 1.698E-09 1.680E-09 1.533E-09

92.0938 5.917E-10 1.142E-09 1.476E-09 1.382E-09

92.3125 5.770E-10 9.060E-10 1.437E-09 1.292E-09

92.5313 5.658E-10 8.660E-10 1.453E-09 1.214E-09

92.7500 5.604E-10 8.737E-10 1.434E-09 1.158E-09

92.9688 5.596E-10 8.596E-10 1.366E-09 1.155E-09

93.1875 5.611E-10 8.290E-10 1.285E-09 1.192E-09

93.4063 5.644E-10 8.147E-10 1.227E-09 1.227E-09

93.6250 5.703E-10 8.314E-10 1.201E-09 1.233E-09

93.8438 5.780E-10 8.613E-10 1.187E-09 1.222E-09

94.0625 5.831E-10 8.758E-10 1.162E-09 1.215E-09

94.2813 5.828E-10 8.673E-10 1.124E-09 1.214E-09

94.5000 5.790E-10 8.501E-10 1.091E-09 1.214E-09

94.7188 5.742E-10 8.381E-10 1.076E-09 1.215E-09

94.9375 5.681E-10 8.328E-10 1.079E-09 1.223E-09

95.1563 5.594E-10 8.333E-10 1.088E-09 1.235E-09

95.3750 5.486E-10 8.432E-10 1.094E-09 1.237E-09

95.5938 5.380E-10 8.610E-10 1.098E-09 1.227E-09

95.8125 5.292E-10 8.721E-10 1.106E-09 1.218E-09

96.0313 5.219E-10 8.603E-10 1.120E-09 1.224E-09

96.2500 5.167E-10 8.267E-10 1.138E-09 1.246E-09

96.4688 5.148E-10 7.880E-10 1.152E-09 1.272E-09

96.6875 5.173E-10 7.591E-10 1.154E-09 1.287E-09

96.9063 5.238E-10 7.436E-10 1.135E-09 1.282E-09

97.1250 5.329E-10 7.394E-10 1.091E-09 1.259E-09

97.3438 5.429E-10 7.452E-10 1.027E-09 1.234E-09

97.5625 5.522E-10 7.584E-10 9.637E-10 1.231E-09

97.7813 5.598E-10 7.745E-10 9.243E-10 1.261E-09

98.0000 5.642E-10 7.909E-10 9.163E-10 1.308E-09

98.2188 5.636E-10 8.078E-10 9.331E-10 1.342E-09

98.4375 5.571E-10 8.235E-10 9.671E-10 1.347E-09

98.6563 5.472E-10 8.309E-10 1.010E-09 1.323E-09

98.8750 5.394E-10 8.240E-10 1.048E-09 1.273E-09

99.0938 5.390E-10 8.050E-10 1.069E-09 1.199E-09

99.3125 5.471E-10 7.839E-10 1.080E-09 1.116E-09

99.5313 5.611E-10 7.720E-10 1.099E-09 1.052E-09

99.7500 5.764E-10 7.766E-10 1.128E-09 1.035E-09

99.9688 5.882E-10 7.974E-10 1.150E-09 1.071E-09

100.1875 5.935E-10 8.243E-10 1.152E-09 1.139E-09

100.4063 5.916E-10 8.388E-10 1.150E-09 1.203E-09

100.6250 5.847E-10 8.237E-10 1.168E-09 1.226E-09

100.8438 5.752E-10 7.789E-10 1.204E-09 1.202E-09

101.0625 5.648E-10 7.279E-10 1.228E-09 1.153E-09

101.2813 5.552E-10 7.022E-10 1.209E-09 1.110E-09

101.5000 5.495E-10 7.134E-10 1.151E-09 1.087E-09

101.7188 5.496E-10 7.433E-10 1.089E-09 1.072E-09

101.9375 5.543E-10 7.656E-10 1.055E-09 1.057E-09

102.1563 5.588E-10 7.722E-10 1.058E-09 1.047E-09

102.3750 5.594E-10 7.751E-10 1.077E-09 1.060E-09

102.5938 5.565E-10 7.881E-10 1.085E-09 1.103E-09

102.8125 5.535E-10 8.121E-10 1.065E-09 1.166E-09

103.0313 5.530E-10 8.377E-10 1.022E-09 1.229E-09

103.2500 5.550E-10 8.548E-10 9.713E-10 1.274E-09

103.4688 5.555E-10 8.566E-10 9.300E-10 1.293E-09

103.6875 5.516E-10 8.426E-10 9.100E-10 1.292E-09

103.9063 5.603E-10 8.355E-10 9.304E-10 1.300E-09

104.1250 6.355E-10 8.992E-10 1.039E-09 1.389E-09

104.3438 8.409E-10 1.110E-09 1.300E-09 1.637E-09

104.5625 1.174E-09 1.472E-09 1.731E-09 2.052E-09

104.7813 1.521E-09 1.869E-09 2.236E-09 2.513E-09

105.0000 1.728E-09 2.136E-09 2.635E-09 2.831E-09

105.2188 1.724E-09 2.192E-09 2.785E-09 2.882E-09

105.4375 1.562E-09 2.079E-09 2.668E-09 2.689E-09

105.6563 1.342E-09 1.875E-09 2.371E-09 2.368E-09

105.8750 1.124E-09 1.625E-09 2.003E-09 2.026E-09

106.0938 9.367E-10 1.359E-09 1.646E-09 1.716E-09

106.3125 8.059E-10 1.135E-09 1.358E-09 1.461E-09

106.5313 7.503E-10 9.968E-10 1.166E-09 1.274E-09

106.7500 7.488E-10 9.304E-10 1.060E-09 1.156E-09

106.9688 7.504E-10 8.892E-10 1.010E-09 1.087E-09

107.1875 7.190E-10 8.479E-10 9.916E-10 1.043E-09

107.4063 6.589E-10 8.116E-10 9.920E-10 1.016E-09

107.6250 5.967E-10 7.843E-10 1.006E-09 1.010E-09

107.8438 5.531E-10 7.583E-10 1.019E-09 1.024E-09

108.0625 5.328E-10 7.322E-10 1.012E-09 1.047E-09

108.2813 5.306E-10 7.175E-10 9.758E-10 1.068E-09

108.5000 5.395E-10 7.217E-10 9.302E-10 1.087E-09

108.7188 5.518E-10 7.334E-10 9.117E-10 1.104E-09

108.9375 5.599E-10 7.311E-10 9.410E-10 1.119E-09

109.1563 5.591E-10 7.020E-10 1.000E-09 1.131E-09

109.3750 5.504E-10 6.519E-10 1.051E-09 1.142E-09

109.5938 5.387E-10 5.994E-10 1.070E-09 1.153E-09

109.8125 5.285E-10 5.637E-10 1.072E-09 1.159E-09

110.0313 5.217E-10 5.547E-10 1.084E-09 1.154E-09

110.2500 5.194E-10 5.709E-10 1.115E-09 1.141E-09

110.4688 5.220E-10 6.023E-10 1.140E-09 1.135E-09

110.6875 5.284E-10 6.357E-10 1.129E-09 1.139E-09

110.9063 5.344E-10 6.588E-10 1.084E-09 1.137E-09

111.1250 5.361E-10 6.651E-10 1.037E-09 1.110E-09

111.3438 5.325E-10 6.574E-10 1.015E-09 1.063E-09

111.5625 5.265E-10 6.472E-10 1.013E-09 1.019E-09

111.7813 5.225E-10 6.486E-10 1.013E-09 9.993E-10

112.0000 5.237E-10 6.675E-10 1.012E-09 1.005E-09

112.2188 5.300E-10 6.961E-10 1.018E-09 1.022E-09

112.4375 5.385E-10 7.192E-10 1.034E-09 1.037E-09

112.6563 5.439E-10 7.273E-10 1.047E-09 1.043E-09

112.8750 5.424E-10 7.220E-10 1.046E-09 1.038E-09

113.0938 5.340E-10 7.113E-10 1.033E-09 1.024E-09

113.3125 5.235E-10 7.046E-10 1.013E-09 1.011E-09

113.5313 5.169E-10 7.132E-10 9.898E-10 1.017E-09

113.7500 5.170E-10 7.463E-10 9.686E-10 1.051E-09

113.9688 5.220E-10 8.006E-10 9.551E-10 1.107E-09

114.1875 5.280E-10 8.549E-10 9.527E-10 1.161E-09

114.4063 5.317E-10 8.829E-10 9.593E-10 1.198E-09

114.6250 5.325E-10 8.729E-10 9.706E-10 1.206E-09

114.8438 5.321E-10 8.362E-10 9.843E-10 1.185E-09

115.0625 5.330E-10 7.940E-10 9.994E-10 1.137E-09

115.2813 5.362E-10 7.596E-10 1.017E-09 1.072E-09

115.5000 5.394E-10 7.338E-10 1.040E-09 9.983E-10

115.7188 5.397E-10 7.147E-10 1.072E-09 9.274E-10

115.9375 5.379E-10 7.043E-10 1.109E-09 8.715E-10

116.1563 5.389E-10 7.048E-10 1.138E-09 8.430E-10

116.3750 5.452E-10 7.119E-10 1.141E-09 8.495E-10

116.5938 5.535E-10 7.187E-10 1.118E-09 8.898E-10

116.8125 5.595E-10 7.217E-10 1.083E-09 9.517E-10

117.0313 5.623E-10 7.173E-10 1.053E-09 1.018E-09

117.2500 5.644E-10 7.005E-10 1.030E-09 1.076E-09

117.4688 5.709E-10 6.760E-10 1.013E-09 1.110E-09

117.6875 5.825E-10 6.568E-10 1.008E-09 1.113E-09

117.9063 5.886E-10 6.475E-10 1.019E-09 1.108E-09

118.1250 5.888E-10 6.545E-10 1.050E-09 1.104E-09

118.3438 5.957E-10 6.813E-10 1.098E-09 1.045E-09

118.5625 5.749E-10 6.797E-10 1.082E-09 1.282E-09

118.7813 6.180E-10 7.986E-10 1.038E-09 3.522E-09

119.0000 1.652E-09 2.176E-09 1.944E-09 1.058E-08

119.2188 5.833E-09 7.192E-09 6.207E-09 2.343E-08

119.4375 1.496E-08 1.748E-08 1.591E-08 3.793E-08

119.6563 2.737E-08 3.070E-08 2.937E-08 4.607E-08

119.8750 3.717E-08 4.024E-08 4.015E-08 4.250E-08

120.0938 3.826E-08 4.009E-08 4.153E-08 2.956E-08

120.3125 2.990E-08 3.039E-08 3.264E-08 1.512E-08

120.5313 1.743E-08 1.726E-08 1.922E-08 5.538E-09

120.7500 7.285E-09 7.183E-09 8.280E-09 1.725E-09

120.9688 2.138E-09 2.302E-09 2.720E-09 1.060E-09

121.1875 6.626E-10 9.241E-10 1.115E-09 1.122E-09

121.4063 5.388E-10 7.443E-10 9.737E-10 1.106E-09

121.6250 5.603E-10 7.051E-10 1.007E-09 1.060E-09

121.8438 5.442E-10 6.721E-10 1.016E-09 1.050E-09

122.0625 5.435E-10 6.731E-10 1.039E-09 1.050E-09

122.2813 5.495E-10 6.916E-10 1.044E-09 1.049E-09

122.5000 5.465E-10 7.219E-10 1.004E-09 1.051E-09

122.7188 5.395E-10 7.618E-10 9.361E-10 1.071E-09

122.9375 5.333E-10 7.955E-10 8.753E-10 1.113E-09

123.1563 5.311E-10 8.119E-10 8.465E-10 1.167E-09

123.3750 5.361E-10 8.152E-10 8.503E-10 1.205E-09

123.5938 5.462E-10 8.169E-10 8.629E-10 1.211E-09

123.8125 5.540E-10 8.255E-10 8.609E-10 1.194E-09

124.0313 5.536E-10 8.383E-10 8.422E-10 1.169E-09

124.2500 5.460E-10 8.461E-10 8.242E-10 1.141E-09

124.4688 5.378E-10 8.493E-10 8.268E-10 1.103E-09

124.6875 5.344E-10 8.614E-10 8.569E-10 1.057E-09

124.9063 5.335E-10 8.902E-10 9.041E-10 1.021E-09

125.1250 5.290E-10 9.221E-10 9.492E-10 1.014E-09

125.3438 5.187E-10 9.321E-10 9.746E-10 1.040E-09

125.5625 5.064E-10 9.046E-10 9.730E-10 1.077E-09

125.7813 4.969E-10 8.455E-10 9.489E-10 1.100E-09

126.0000 4.910E-10 7.788E-10 9.147E-10 1.103E-09

126.2188 4.871E-10 7.346E-10 8.831E-10 1.101E-09

126.4375 4.844E-10 7.301E-10 8.625E-10 1.104E-09

126.6563 4.847E-10 7.563E-10 8.576E-10 1.105E-09

126.8750 4.902E-10 7.864E-10 8.688E-10 1.089E-09

127.0938 5.006E-10 7.993E-10 8.913E-10 1.051E-09

127.3125 5.116E-10 7.945E-10 9.185E-10 1.007E-09

127.5313 5.177E-10 7.828E-10 9.475E-10 9.757E-10

127.7500 5.165E-10 7.698E-10 9.791E-10 9.712E-10

127.9688 5.113E-10 7.518E-10 1.010E-09 9.967E-10

128.1875 5.070E-10 7.256E-10 1.032E-09 1.040E-09

128.4063 5.047E-10 6.972E-10 1.037E-09 1.074E-09

128.6250 5.015E-10 6.785E-10 1.025E-09 1.076E-09

128.8438 4.952E-10 6.757E-10 1.004E-09 1.047E-09

129.0625 4.883E-10 6.833E-10 9.761E-10 1.015E-09

129.2813 4.853E-10 6.902E-10 9.440E-10 1.008E-09

129.5000 4.878E-10 6.943E-10 9.103E-10 1.019E-09

129.7188 4.922E-10 7.056E-10 8.830E-10 1.021E-09

129.9375 4.946E-10 7.313E-10 8.720E-10 9.953E-10

130.1563 4.952E-10 7.605E-10 8.795E-10 9.522E-10

130.3750 4.984E-10 7.692E-10 8.987E-10 9.197E-10

130.5938 5.066E-10 7.433E-10 9.214E-10 9.140E-10

130.8125 5.164E-10 6.952E-10 9.453E-10 9.273E-10

131.0313 5.215E-10 6.536E-10 9.684E-10 9.405E-10

131.2500 5.189E-10 6.407E-10 9.828E-10 9.423E-10

131.4688 5.119E-10 6.574E-10 9.825E-10 9.357E-10

131.6875 5.051E-10 6.874E-10 9.752E-10 9.322E-10

131.9063 5.001E-10 7.141E-10 9.771E-10 9.431E-10

132.1250 4.953E-10 7.328E-10 9.932E-10 9.718E-10

132.3438 4.900E-10 7.497E-10 1.010E-09 1.009E-09

132.5625 4.867E-10 7.710E-10 1.008E-09 1.038E-09

132.7813 4.881E-10 7.948E-10 9.851E-10 1.049E-09

133.0000 4.932E-10 8.126E-10 9.508E-10 1.047E-09

133.2188 4.984E-10 8.164E-10 9.168E-10 1.047E-09

133.4375 5.010E-10 8.035E-10 8.876E-10 1.055E-09

133.6563 5.010E-10 7.782E-10 8.641E-10 1.066E-09

133.8750 4.998E-10 7.526E-10 8.492E-10 1.071E-09

134.0938 4.971E-10 7.411E-10 8.470E-10 1.071E-09

134.3125 4.916E-10 7.475E-10 8.562E-10 1.068E-09

134.5313 5.051E-10 7.592E-10 8.678E-10 1.062E-09

134.7500 6.222E-10 7.581E-10 8.734E-10 1.049E-09

134.9688 9.724E-10 7.384E-10 8.749E-10 1.029E-09

135.1875 1.597E-09 7.093E-10 8.804E-10 1.007E-09

135.4063 2.303E-09 6.816E-10 8.903E-10 9.907E-10

135.6250 2.721E-09 6.631E-10 8.991E-10 9.902E-10

135.8438 2.590E-09 6.780E-10 9.194E-10 1.032E-09

136.0625 2.000E-09 7.834E-10 9.970E-10 1.147E-09

136.2813 1.302E-09 1.034E-09 1.175E-09 1.330E-09

136.5000 8.048E-10 1.404E-09 1.427E-09 1.507E-09

136.7188 5.772E-10 1.740E-09 1.641E-09 1.575E-09

136.9375 5.147E-10 1.850E-09 1.696E-09 1.498E-09

137.1563 5.047E-10 1.655E-09 1.559E-09 1.340E-09

137.3750 5.021E-10 1.267E-09 1.315E-09 1.204E-09

137.5938 5.031E-10 8.909E-10 1.085E-09 1.136E-09

137.8125 5.076E-10 6.698E-10 9.425E-10 1.112E-09

138.0313 5.102E-10 6.098E-10 8.865E-10 1.086E-09

138.2500 5.076E-10 6.374E-10 8.806E-10 1.035E-09

138.4688 5.012E-10 6.831E-10 8.899E-10 9.628E-10

138.6875 4.958E-10 7.140E-10 8.977E-10 8.889E-10

138.9063 4.943E-10 7.230E-10 9.009E-10 8.419E-10

139.1250 4.964E-10 7.152E-10 9.019E-10 8.423E-10

139.3438 4.991E-10 7.001E-10 9.021E-10 8.852E-10

139.5625 5.000E-10 6.840E-10 8.987E-10 9.387E-10

139.7813 4.991E-10 6.661E-10 8.870E-10 9.649E-10

140.0000 4.984E-10 6.445E-10 8.668E-10 9.497E-10

140.2188 4.994E-10 6.249E-10 8.455E-10 9.114E-10

140.4375 5.012E-10 6.182E-10 8.333E-10 8.806E-10

140.6563 5.005E-10 6.302E-10 8.336E-10 8.701E-10

140.8750 4.951E-10 6.558E-10 8.410E-10 8.691E-10

141.0938 4.863E-10 6.836E-10 8.485E-10 8.636E-10

141.3125 4.783E-10 7.051E-10 8.527E-10 8.559E-10

141.5313 4.742E-10 7.181E-10 8.534E-10 8.591E-10

141.7500 4.742E-10 7.240E-10 8.490E-10 8.762E-10

141.9688 4.767E-10 7.257E-10 8.363E-10 8.937E-10

142.1875 4.803E-10 7.258E-10 8.152E-10 8.957E-10

142.4063 4.846E-10 7.245E-10 7.938E-10 8.823E-10

142.6250 4.892E-10 7.199E-10 7.849E-10 8.706E-10

142.8438 4.932E-10 7.121E-10 7.954E-10 8.799E-10

143.0625 4.955E-10 7.054E-10 8.187E-10 9.166E-10

143.2813 4.952E-10 7.026E-10 8.391E-10 9.700E-10

143.5000 4.925E-10 7.000E-10 8.449E-10 1.019E-09

143.7188 4.879E-10 6.915E-10 8.367E-10 1.040E-09

143.9375 4.823E-10 6.779E-10 8.246E-10 1.021E-09

144.1563 4.763E-10 6.676E-10 8.196E-10 9.653E-10

144.3750 4.710E-10 6.688E-10 8.245E-10 8.973E-10

144.5938 4.673E-10 6.834E-10 8.324E-10 8.450E-10

144.8125 4.649E-10 7.079E-10 8.353E-10 8.239E-10

145.0313 4.630E-10 7.355E-10 8.338E-10 8.316E-10

145.2500 4.621E-10 7.568E-10 8.355E-10 8.576E-10

145.4688 4.634E-10 7.619E-10 8.438E-10 8.927E-10

145.6875 4.669E-10 7.457E-10 8.514E-10 9.303E-10

145.9063 4.699E-10 7.127E-10 8.493E-10 9.644E-10

146.1250 4.708E-10 6.732E-10 8.413E-10 9.916E-10

146.3438 4.702E-10 6.388E-10 8.435E-10 1.011E-09

146.5625 4.698E-10 6.210E-10 8.675E-10 1.019E-09

146.7813 4.697E-10 6.295E-10 9.044E-10 1.008E-09

147.0000 4.696E-10 6.653E-10 9.305E-10 9.786E-10

147.2188 4.716E-10 7.138E-10 9.281E-10 9.407E-10

147.4375 4.794E-10 7.490E-10 9.014E-10 9.087E-10

147.6563 4.932E-10 7.508E-10 8.710E-10 8.874E-10

147.8750 5.075E-10 7.192E-10 8.556E-10 8.747E-10

148.0938 5.152E-10 6.714E-10 8.600E-10 8.739E-10

148.3125 5.136E-10 6.289E-10 8.797E-10 8.919E-10

148.5313 5.068E-10 6.061E-10 9.061E-10 9.237E-10

148.7500 5.011E-10 6.091E-10 9.277E-10 9.479E-10

148.9688 4.997E-10 6.363E-10 9.334E-10 9.463E-10

149.1875 5.017E-10 6.779E-10 9.262E-10 9.230E-10

149.4063 5.045E-10 7.188E-10 9.281E-10 8.980E-10

149.6250 5.075E-10 7.470E-10 9.599E-10 8.840E-10

149.8438 5.111E-10 7.593E-10 1.013E-09 8.751E-10

150.0625 5.138E-10 7.588E-10 1.049E-09 8.620E-10

150.2813 5.114E-10 7.497E-10 1.037E-09 8.495E-10

150.5000 5.013E-10 7.365E-10 9.797E-10 8.535E-10

150.7188 4.860E-10 7.251E-10 9.087E-10 8.783E-10

150.9375 4.714E-10 7.195E-10 8.530E-10 9.052E-10

151.1563 4.612E-10 7.171E-10 8.223E-10 9.072E-10

151.3750 4.548E-10 7.101E-10 8.100E-10 8.758E-10

151.5938 4.501E-10 6.955E-10 8.060E-10 8.311E-10

151.8125 4.454E-10 6.821E-10 8.032E-10 8.044E-10

152.0313 4.404E-10 6.823E-10 7.973E-10 8.111E-10

152.2500 4.361E-10 6.948E-10 7.854E-10 8.389E-10

152.4688 4.349E-10 7.022E-10 7.664E-10 8.620E-10

152.6875 4.401E-10 6.892E-10 7.412E-10 8.648E-10

152.9063 4.519E-10 6.618E-10 7.147E-10 8.514E-10

153.1250 4.650E-10 6.412E-10 6.970E-10 8.351E-10

153.3438 4.733E-10 6.409E-10 7.007E-10 8.243E-10

153.5625 4.760E-10 6.548E-10 7.305E-10 8.200E-10

153.7813 4.772E-10 6.681E-10 7.758E-10 8.211E-10

154.0000 4.800E-10 6.752E-10 8.155E-10 8.287E-10

154.2188 4.820E-10 6.812E-10 8.331E-10 8.461E-10

154.4375 4.794E-10 6.923E-10 8.251E-10 8.759E-10

154.6563 4.719E-10 7.062E-10 7.987E-10 9.176E-10

154.8750 4.632E-10 7.124E-10 7.651E-10 9.642E-10

155.0938 4.568E-10 7.024E-10 7.367E-10 1.002E-09

155.3125 4.550E-10 6.784E-10 7.253E-10 1.015E-09

155.5313 4.585E-10 6.535E-10 7.368E-10 9.876E-10

155.7500 4.668E-10 6.387E-10 7.649E-10 9.261E-10

155.9688 4.763E-10 6.320E-10 7.934E-10 8.589E-10

156.1875 4.823E-10 6.241E-10 8.062E-10 8.221E-10

156.4063 4.828E-10 6.110E-10 7.986E-10 8.304E-10

156.6250 4.795E-10 5.972E-10 7.780E-10 8.668E-10

156.8438 4.761E-10 5.860E-10 7.565E-10 9.013E-10

157.0625 4.740E-10 5.748E-10 7.426E-10 9.165E-10

157.2813 4.731E-10 5.631E-10 7.419E-10 9.129E-10

157.5000 4.770E-10 5.647E-10 7.611E-10 9.008E-10

157.7188 4.954E-10 6.055E-10 8.085E-10 8.959E-10

157.9375 5.364E-10 7.007E-10 8.853E-10 9.155E-10

158.1563 5.930E-10 8.281E-10 9.756E-10 9.657E-10

158.3750 6.401E-10 9.314E-10 1.045E-09 1.027E-09

158.5938 6.508E-10 9.605E-10 1.058E-09 1.065E-09

158.8125 6.177E-10 9.121E-10 1.001E-09 1.059E-09

159.0313 5.594E-10 8.265E-10 9.009E-10 1.018E-09

159.2500 5.052E-10 7.469E-10 8.056E-10 9.629E-10

159.4688 4.738E-10 6.910E-10 7.532E-10 9.046E-10

159.6875 4.647E-10 6.584E-10 7.503E-10 8.473E-10

159.9063 4.661E-10 6.467E-10 7.791E-10 7.996E-10

160.1250 4.673E-10 6.492E-10 8.178E-10 7.759E-10

160.3438 4.654E-10 6.480E-10 8.548E-10 7.820E-10

160.5625 4.631E-10 6.253E-10 8.869E-10 8.072E-10

160.7813 4.628E-10 5.839E-10 9.101E-10 8.325E-10

161.0000 4.629E-10 5.473E-10 9.143E-10 8.435E-10

161.2188 4.608E-10 5.372E-10 8.911E-10 8.376E-10

161.4375 4.565E-10 5.547E-10 8.468E-10 8.211E-10

161.6563 4.523E-10 5.835E-10 8.020E-10 8.041E-10

161.8750 4.504E-10 6.077E-10 7.757E-10 7.963E-10

162.0938 4.516E-10 6.241E-10 7.707E-10 8.054E-10

162.3125 4.557E-10 6.400E-10 7.772E-10 8.335E-10

162.5313 4.614E-10 6.640E-10 7.845E-10 8.727E-10

162.7500 4.658E-10 6.967E-10 7.893E-10 9.077E-10

162.9688 4.661E-10 7.289E-10 7.990E-10 9.279E-10

163.1875 4.628E-10 7.455E-10 8.279E-10 9.368E-10

163.4063 4.594E-10 7.348E-10 8.812E-10 9.443E-10

163.6250 4.582E-10 6.989E-10 9.422E-10 9.526E-10

163.8438 4.574E-10 6.582E-10 9.813E-10 9.545E-10

164.0625 4.537E-10 6.387E-10 9.816E-10 9.442E-10

164.2813 4.474E-10 6.514E-10 9.499E-10 9.254E-10

164.5000 4.425E-10 6.828E-10 9.035E-10 9.064E-10

164.7188 4.417E-10 7.083E-10 8.538E-10 8.923E-10

164.9375 4.440E-10 7.137E-10 8.053E-10 8.823E-10

165.1563 4.471E-10 7.027E-10 7.641E-10 8.730E-10

165.3750 4.499E-10 6.872E-10 7.413E-10 8.641E-10

165.5938 4.529E-10 6.778E-10 7.464E-10 8.589E-10

165.8125 4.566E-10 6.806E-10 7.774E-10 8.596E-10

166.0313 4.610E-10 6.962E-10 8.203E-10 8.630E-10

166.2500 4.659E-10 7.169E-10 8.590E-10 8.636E-10

166.4688 4.704E-10 7.285E-10 8.863E-10 8.587E-10

166.6875 4.730E-10 7.205E-10 9.042E-10 8.480E-10

166.9063 4.730E-10 6.970E-10 9.154E-10 8.308E-10

167.1250 4.707E-10 6.742E-10 9.163E-10 8.086E-10

167.3438 4.666E-10 6.671E-10 8.995E-10 7.912E-10

167.5625 4.604E-10 6.766E-10 8.632E-10 7.918E-10

167.7813 4.535E-10 6.912E-10 8.175E-10 8.106E-10

168.0000 4.492E-10 6.981E-10 7.780E-10 8.290E-10

168.2188 4.511E-10 6.920E-10 7.527E-10 8.283E-10

168.4375 4.581E-10 6.751E-10 7.359E-10 8.124E-10

168.6563 4.643E-10 6.522E-10 7.195E-10 8.031E-10

168.8750 4.640E-10 6.313E-10 7.046E-10 8.137E-10

169.0938 4.559E-10 6.222E-10 7.013E-10 8.337E-10

169.3125 4.433E-10 6.276E-10 7.163E-10 8.434E-10

169.5313 4.323E-10 6.353E-10 7.462E-10 8.341E-10

169.7500 4.286E-10 6.273E-10 7.814E-10 8.151E-10

169.9688 4.345E-10 5.999E-10 8.130E-10 8.054E-10

170.1875 4.458E-10 5.692E-10 8.354E-10 8.215E-10

170.4063 4.543E-10 5.540E-10 8.452E-10 8.651E-10

170.6250 4.549E-10 5.573E-10 8.432E-10 9.179E-10

170.8438 4.496E-10 5.665E-10 8.334E-10 9.518E-10

171.0625 4.450E-10 5.697E-10 8.194E-10 9.502E-10

171.2813 4.464E-10 5.682E-10 8.026E-10 9.202E-10

171.5000 4.540E-10 5.724E-10 7.857E-10 8.848E-10

171.7188 4.635E-10 5.882E-10 7.766E-10 8.611E-10

171.9375 4.689E-10 6.068E-10 7.854E-10 8.478E-10

172.1563 4.671E-10 6.117E-10 8.152E-10 8.338E-10

172.3750 4.607E-10 5.974E-10 8.555E-10 8.156E-10

172.5938 4.550E-10 5.774E-10 8.871E-10 8.028E-10

172.8125 4.537E-10 5.700E-10 8.952E-10 8.048E-10

173.0313 4.557E-10 5.778E-10 8.805E-10 8.165E-10

173.2500 4.580E-10 5.864E-10 8.567E-10 8.226E-10

173.4688 4.588E-10 5.851E-10 8.399E-10 8.179E-10

173.6875 4.581E-10 5.797E-10 8.378E-10 8.179E-10

173.9063 4.573E-10 5.847E-10 8.487E-10 8.425E-10

174.1250 4.590E-10 6.065E-10 8.659E-10 8.908E-10

174.3438 4.656E-10 6.361E-10 8.814E-10 9.384E-10

174.5625 4.761E-10 6.581E-10 8.885E-10 9.577E-10

174.7813 4.854E-10 6.661E-10 8.842E-10 9.391E-10

175.0000 4.880E-10 6.651E-10 8.671E-10 8.945E-10

175.2188 4.822E-10 6.625E-10 8.383E-10 8.420E-10

175.4375 4.732E-10 6.595E-10 8.034E-10 7.927E-10

175.6563 4.666E-10 6.504E-10 7.675E-10 7.568E-10

175.8750 4.625E-10 6.316E-10 7.317E-10 7.447E-10

176.0938 4.586E-10 6.141E-10 7.017E-10 7.583E-10

176.3125 4.542E-10 6.141E-10 6.892E-10 7.942E-10

176.5313 4.488E-10 6.354E-10 6.999E-10 8.419E-10

176.7500 4.481E-10 6.703E-10 7.337E-10 8.769E-10

176.9688 4.553E-10 7.009E-10 7.804E-10 8.977E-10

177.1875 4.564E-10 7.030E-10 8.081E-10 9.156E-10

177.4063 4.549E-10 6.881E-10 8.094E-10 8.746E-10

177.6250 4.742E-10 6.774E-10 8.212E-10 8.256E-10

177.8438 4.783E-10 6.287E-10 8.214E-10 1.011E-09

178.0625 4.754E-10 6.192E-10 8.208E-10 1.074E-09

178.2813 6.191E-10 8.548E-10 1.011E-09 1.069E-09

178.5000 6.619E-10 8.794E-10 1.116E-09 8.962E-09

178.7188 6.359E-10 9.011E-10 9.352E-10 4.796E-08

178.9375 6.493E-09 9.291E-09 6.816E-09 1.435E-07

179.1563 3.612E-08 4.860E-08 3.955E-08 2.871E-07

179.3750 1.101E-07 1.412E-07 1.248E-07 4.169E-07

179.5938 2.236E-07 2.750E-07 2.596E-07 4.549E-07

179.8125 3.294E-07 3.896E-07 3.897E-07 3.755E-07

180.0313 3.652E-07 4.147E-07 4.384E-07 2.304E-07

180.2500 3.073E-07 3.336E-07 3.733E-07 9.984E-08

180.4688 1.936E-07 1.989E-07 2.371E-07 2.711E-08

180.6875 8.750E-08 8.320E-08 1.073E-07 3.576E-09

180.9063 2.595E-08 2.153E-08 3.105E-08 8.642E-10

181.1250 4.431E-09 2.651E-09 4.566E-09 1.239E-09

181.3438 1.007E-09 7.233E-10 8.652E-10 1.005E-09

181.5625 9.494E-10 9.905E-10 1.239E-09 9.201E-10

181.7813 6.609E-10 8.114E-10 1.101E-09 1.075E-09

182.0000 4.890E-10 8.397E-10 1.067E-09 1.202E-09

182.2188 4.839E-10 1.046E-09 1.259E-09 1.269E-09

182.4375 4.664E-10 1.178E-09 1.343E-09 1.270E-09

182.6563 4.469E-10 1.188E-09 1.265E-09 1.177E-09

182.8750 4.443E-10 1.074E-09 1.106E-09 1.028E-09

183.0938 4.367E-10 8.793E-10 9.372E-10 8.827E-10

183.3125 4.291E-10 7.050E-10 8.200E-10 7.825E-10

183.5313 4.308E-10 6.075E-10 7.641E-10 7.514E-10

183.7500 4.357E-10 5.755E-10 7.372E-10 7.827E-10

183.9688 4.408E-10 5.779E-10 7.218E-10 8.401E-10

184.1875 4.453E-10 5.870E-10 7.197E-10 8.799E-10

184.4063 4.471E-10 5.869E-10 7.307E-10 8.738E-10

184.6250 4.480E-10 5.787E-10 7.454E-10 8.228E-10

184.8438 4.508E-10 5.696E-10 7.504E-10 7.603E-10

185.0625 4.544E-10 5.644E-10 7.414E-10 7.288E-10

185.2813 4.548E-10 5.665E-10 7.319E-10 7.478E-10

185.5000 4.498E-10 5.794E-10 7.389E-10 7.994E-10

185.7188 4.411E-10 6.020E-10 7.612E-10 8.459E-10

185.9375 4.341E-10 6.256E-10 7.779E-10 8.628E-10

186.1563 4.318E-10 6.369E-10 7.687E-10 8.507E-10

186.3750 4.327E-10 6.299E-10 7.335E-10 8.193E-10

186.5938 4.335E-10 6.138E-10 6.943E-10 7.748E-10

186.8125 4.332E-10 6.027E-10 6.796E-10 7.257E-10

187.0313 4.337E-10 5.999E-10 7.012E-10 6.908E-10

187.2500 4.373E-10 5.936E-10 7.429E-10 6.891E-10

187.4688 4.436E-10 5.708E-10 7.719E-10 7.238E-10

187.6875 4.484E-10 5.318E-10 7.691E-10 7.778E-10

187.9063 4.475E-10 4.924E-10 7.456E-10 8.250E-10

188.1250 4.417E-10 4.721E-10 7.284E-10 8.503E-10

188.3438 4.368E-10 4.795E-10 7.335E-10 8.620E-10

188.5625 4.386E-10 5.074E-10 7.575E-10 8.798E-10

188.7813 4.469E-10 5.378E-10 7.905E-10 9.093E-10

189.0000 4.560E-10 5.542E-10 8.235E-10 9.319E-10

189.2188 4.604E-10 5.514E-10 8.437E-10 9.249E-10

189.4375 4.588E-10 5.371E-10 8.337E-10 8.887E-10

189.6563 4.534E-10 5.259E-10 7.878E-10 8.469E-10

189.8750 4.468E-10 5.302E-10 7.254E-10 8.231E-10

190.0938 4.402E-10 5.512E-10 6.795E-10 8.215E-10

190.3125 4.341E-10 5.765E-10 6.678E-10 8.316E-10

190.5313 4.292E-10 5.884E-10 6.813E-10 8.438E-10

190.7500 4.264E-10 5.769E-10 6.995E-10 8.542E-10

190.9688 4.252E-10 5.489E-10 7.134E-10 8.582E-10

191.1875 4.244E-10 5.224E-10 7.287E-10 8.493E-10

191.4063 4.227E-10 5.128E-10 7.506E-10 8.274E-10

191.6250 4.203E-10 5.210E-10 7.724E-10 8.059E-10

191.8438 4.189E-10 5.342E-10 7.806E-10 8.022E-10

192.0625 4.200E-10 5.383E-10 7.717E-10 8.200E-10

192.2813 4.231E-10 5.305E-10 7.589E-10 8.434E-10

192.5000 4.256E-10 5.211E-10 7.594E-10 8.507E-10

192.7188 4.254E-10 5.233E-10 7.763E-10 8.330E-10

192.9375 4.235E-10 5.386E-10 7.938E-10 8.006E-10

193.1563 4.221E-10 5.555E-10 7.935E-10 7.767E-10

193.3750 4.227E-10 5.623E-10 7.725E-10 7.838E-10

193.5938 4.252E-10 5.608E-10 7.456E-10 8.273E-10

193.8125 4.287E-10 5.613E-10 7.321E-10 8.856E-10

194.0313 4.324E-10 5.659E-10 7.419E-10 9.209E-10

194.2500 4.350E-10 5.619E-10 7.718E-10 9.098E-10

194.4688 4.355E-10 5.399E-10 8.088E-10 8.631E-10

194.6875 4.339E-10 5.118E-10 8.380E-10 8.136E-10

194.9063 4.313E-10 5.029E-10 8.475E-10 7.848E-10

195.1250 4.288E-10 5.245E-10 8.326E-10 7.774E-10

195.3438 4.281E-10 5.642E-10 7.984E-10 7.827E-10

195.5625 4.301E-10 6.029E-10 7.603E-10 7.980E-10

195.7813 4.337E-10 6.336E-10 7.368E-10 8.256E-10

196.0000 4.365E-10 6.569E-10 7.375E-10 8.612E-10

196.2188 4.378E-10 6.670E-10 7.563E-10 8.924E-10

196.4375 4.399E-10 6.535E-10 7.784E-10 9.072E-10

196.6563 4.448E-10 6.170E-10 7.952E-10 9.013E-10

196.8750 4.519E-10 5.745E-10 8.109E-10 8.796E-10

197.0938 4.580E-10 5.481E-10 8.342E-10 8.522E-10

197.3125 4.600E-10 5.483E-10 8.649E-10 8.290E-10

197.5313 4.570E-10 5.688E-10 8.909E-10 8.143E-10

197.7500 4.496E-10 5.917E-10 8.985E-10 8.060E-10

197.9688 4.397E-10 5.985E-10 8.839E-10 8.008E-10

198.1875 4.298E-10 5.842E-10 8.539E-10 7.976E-10

198.4063 4.216E-10 5.627E-10 8.163E-10 7.954E-10

198.6250 4.155E-10 5.565E-10 7.756E-10 7.906E-10

198.8438 4.115E-10 5.778E-10 7.382E-10 7.775E-10

199.0625 4.112E-10 6.206E-10 7.172E-10 7.520E-10

199.2813 4.167E-10 6.677E-10 7.250E-10 7.161E-10

199.5000 4.281E-10 7.018E-10 7.585E-10 6.782E-10

199.7188 4.404E-10 7.103E-10 7.982E-10 6.504E-10

199.9375 4.461E-10 6.885E-10 8.235E-10 6.418E-10

200.1563 4.414E-10 6.462E-10 8.272E-10 6.531E-10

200.3750 4.299E-10 6.062E-10 8.122E-10 6.772E-10

200.5938 4.190E-10 5.880E-10 7.827E-10 7.045E-10

200.8125 4.136E-10 5.912E-10 7.426E-10 7.284E-10

201.0313 4.133E-10 5.994E-10 7.027E-10 7.470E-10

201.2500 4.158E-10 5.992E-10 6.806E-10 7.595E-10

201.4688 4.196E-10 5.900E-10 6.892E-10 7.640E-10

201.6875 4.239E-10 5.798E-10 7.238E-10 7.595E-10

201.9063 4.282E-10 5.770E-10 7.639E-10 7.495E-10

202.1250 4.322E-10 5.874E-10 7.883E-10 7.407E-10

202.3438 4.350E-10 6.091E-10 7.894E-10 7.386E-10

202.5625 4.343E-10 6.287E-10 7.737E-10 7.446E-10

202.7813 4.280E-10 6.279E-10 7.518E-10 7.583E-10

203.0000 4.180E-10 6.017E-10 7.299E-10 7.767E-10

203.2188 4.094E-10 5.669E-10 7.101E-10 7.917E-10

203.4375 4.069E-10 5.489E-10 6.944E-10 7.941E-10

203.6563 4.106E-10 5.613E-10 6.847E-10 7.830E-10

203.8750 4.164E-10 5.971E-10 6.818E-10 7.693E-10

204.0938 4.197E-10 6.364E-10 6.869E-10 7.649E-10

204.3125 4.188E-10 6.579E-10 7.027E-10 7.710E-10

204.5313 4.154E-10 6.487E-10 7.286E-10 7.785E-10

204.7500 4.136E-10 6.110E-10 7.570E-10 7.785E-10

204.9688 4.159E-10 5.624E-10 7.760E-10 7.709E-10

205.1875 4.217E-10 5.237E-10 7.771E-10 7.618E-10

205.4063 4.269E-10 5.057E-10 7.597E-10 7.548E-10

205.6250 4.281E-10 5.090E-10 7.304E-10 7.469E-10

205.8438 4.255E-10 5.329E-10 7.014E-10 7.361E-10

206.0625 4.223E-10 5.750E-10 6.861E-10 7.277E-10

206.2813 4.220E-10 6.222E-10 6.909E-10 7.285E-10

206.5000 4.243E-10 6.520E-10 7.084E-10 7.359E-10

206.7188 4.264E-10 6.505E-10 7.208E-10 7.388E-10

206.9375 4.262E-10 6.248E-10 7.133E-10 7.314E-10

207.1563 4.243E-10 5.932E-10 6.862E-10 7.212E-10

207.3750 4.226E-10 5.691E-10 6.546E-10 7.187E-10

207.5938 4.223E-10 5.551E-10 6.381E-10 7.242E-10

207.8125 4.227E-10 5.506E-10 6.480E-10 7.285E-10

208.0313 4.235E-10 5.572E-10 6.803E-10 7.248E-10

208.2500 4.243E-10 5.762E-10 7.192E-10 7.160E-10

208.4688 4.266E-10 6.034E-10 7.462E-10 7.095E-10

208.6875 4.387E-10 6.320E-10 7.510E-10 7.108E-10

208.9063 4.881E-10 6.794E-10 7.480E-10 7.334E-10

209.1250 6.213E-10 8.134E-10 7.954E-10 8.202E-10

209.3438 8.703E-10 1.117E-09 9.851E-10 1.038E-09

209.5625 1.196E-09 1.574E-09 1.365E-09 1.411E-09

209.7813 1.473E-09 1.992E-09 1.840E-09 1.840E-09

210.0000 1.562E-09 2.108E-09 2.175E-09 2.121E-09

210.2188 1.428E-09 1.833E-09 2.181E-09 2.098E-09

210.4375 1.195E-09 1.404E-09 1.921E-09 1.851E-09

210.6563 1.072E-09 1.241E-09 1.716E-09 1.707E-09

210.8750 1.196E-09 1.593E-09 1.877E-09 1.977E-09

211.0938 1.519E-09 2.297E-09 2.390E-09 2.629E-09

211.3125 1.825E-09 2.872E-09 2.888E-09 3.237E-09

211.5313 1.883E-09 2.908E-09 2.956E-09 3.316E-09

211.7500 1.626E-09 2.379E-09 2.492E-09 2.754E-09

211.9688 1.188E-09 1.617E-09 1.769E-09 1.887E-09

212.1875 7.838E-10 9.940E-10 1.160E-09 1.172E-09

212.4063 5.369E-10 6.657E-10 8.420E-10 8.231E-10

212.6250 4.381E-10 5.640E-10 7.508E-10 7.518E-10

212.8438 4.147E-10 5.586E-10 7.451E-10 7.683E-10

213.0625 4.117E-10 5.722E-10 7.510E-10 7.623E-10

213.2813 4.120E-10 5.828E-10 7.646E-10 7.284E-10

213.5000 4.146E-10 5.879E-10 7.871E-10 7.021E-10

213.7188 4.180E-10 5.878E-10 7.982E-10 7.069E-10

213.9375 4.202E-10 5.853E-10 7.789E-10 7.406E-10

214.1563 4.219E-10 5.813E-10 7.320E-10 7.839E-10

214.3750 4.253E-10 5.756E-10 6.782E-10 8.144E-10

214.5938 4.304E-10 5.747E-10 6.380E-10 8.184E-10

214.8125 4.342E-10 5.899E-10 6.196E-10 8.002E-10

215.0313 4.334E-10 6.212E-10 6.194E-10 7.798E-10

215.2500 4.282E-10 6.480E-10 6.295E-10 7.776E-10

215.4688 4.224E-10 6.474E-10 6.436E-10 7.946E-10

215.6875 4.196E-10 6.200E-10 6.558E-10 8.118E-10

215.9063 4.191E-10 5.901E-10 6.579E-10 8.107E-10

216.1250 4.173E-10 5.785E-10 6.450E-10 7.912E-10

216.3438 4.131E-10 5.826E-10 6.243E-10 7.673E-10

216.5625 4.092E-10 5.857E-10 6.128E-10 7.510E-10

216.7813 4.091E-10 5.793E-10 6.213E-10 7.472E-10

217.0000 4.138E-10 5.693E-10 6.442E-10 7.577E-10

217.2188 4.215E-10 5.651E-10 6.673E-10 7.788E-10

217.4375 4.298E-10 5.696E-10 6.817E-10 7.977E-10

217.6563 4.360E-10 5.790E-10 6.858E-10 7.989E-10

217.8750 4.377E-10 5.859E-10 6.812E-10 7.796E-10

218.0938 4.349E-10 5.829E-10 6.742E-10 7.536E-10

218.3125 4.303E-10 5.674E-10 6.775E-10 7.387E-10

218.5313 4.268E-10 5.455E-10 6.998E-10 7.387E-10

218.7500 4.248E-10 5.292E-10 7.309E-10 7.423E-10

218.9688 4.233E-10 5.276E-10 7.446E-10 7.386E-10

219.1875 4.217E-10 5.397E-10 7.245E-10 7.296E-10

219.4063 4.206E-10 5.577E-10 6.823E-10 7.234E-10

219.6250 4.203E-10 5.738E-10 6.453E-10 7.202E-10

219.8438 4.203E-10 5.836E-10 6.288E-10 7.115E-10

220.0625 4.205E-10 5.855E-10 6.278E-10 6.972E-10

220.2813 4.217E-10 5.802E-10 6.323E-10 6.930E-10

220.5000 4.240E-10 5.707E-10 6.408E-10 7.165E-10

220.7188 4.258E-10 5.614E-10 6.546E-10 7.663E-10

220.9375 4.251E-10 5.584E-10 6.676E-10 8.179E-10

221.1563 4.209E-10 5.682E-10 6.687E-10 8.422E-10

221.3750 4.139E-10 5.908E-10 6.555E-10 8.268E-10

221.5938 4.063E-10 6.137E-10 6.406E-10 7.827E-10

221.8125 4.010E-10 6.185E-10 6.411E-10 7.313E-10

222.0313 4.010E-10 5.998E-10 6.605E-10 6.872E-10

222.2500 4.065E-10 5.719E-10 6.855E-10 6.547E-10

222.4688 4.137E-10 5.526E-10 7.017E-10 6.384E-10

222.6875 4.181E-10 5.440E-10 7.107E-10 6.476E-10

222.9063 4.181E-10 5.360E-10 7.254E-10 6.879E-10

223.1250 4.164E-10 5.233E-10 7.489E-10 7.486E-10

223.3438 4.168E-10 5.109E-10 7.653E-10 8.041E-10

223.5625 4.197E-10 5.060E-10 7.560E-10 8.300E-10

223.7813 4.227E-10 5.122E-10 7.211E-10 8.194E-10

224.0000 4.260E-10 5.312E-10 6.831E-10 7.863E-10

224.2188 4.332E-10 5.602E-10 6.702E-10 7.508E-10

224.4375 4.467E-10 5.876E-10 6.977E-10 7.228E-10

224.6563 4.617E-10 5.972E-10 7.569E-10 6.999E-10

224.8750 4.711E-10 5.853E-10 8.200E-10 6.794E-10

225.0938 4.779E-10 5.661E-10 8.570E-10 6.692E-10

225.3125 5.015E-10 5.554E-10 8.541E-10 6.800E-10

225.5313 5.623E-10 5.553E-10 8.205E-10 7.088E-10

225.7500 6.534E-10 5.581E-10 7.764E-10 7.377E-10

225.9688 7.326E-10 5.613E-10 7.356E-10 7.523E-10

226.1875 7.506E-10 5.693E-10 7.005E-10 7.558E-10

226.4063 6.941E-10 5.815E-10 6.726E-10 7.590E-10

226.6250 5.967E-10 5.891E-10 6.608E-10 7.640E-10

226.8438 5.080E-10 5.871E-10 6.741E-10 7.687E-10

227.0625 4.549E-10 5.861E-10 7.088E-10 7.849E-10

227.2813 4.334E-10 6.076E-10 7.526E-10 8.337E-10

227.5000 4.268E-10 6.676E-10 8.010E-10 9.161E-10

227.7188 4.242E-10 7.597E-10 8.581E-10 9.940E-10

227.9375 4.227E-10 8.536E-10 9.159E-10 1.017E-09

228.1563 4.219E-10 9.101E-10 9.434E-10 9.704E-10

228.3750 4.207E-10 9.057E-10 9.122E-10 8.863E-10

228.5938 4.185E-10 8.458E-10 8.302E-10 8.139E-10

228.8125 4.167E-10 7.571E-10 7.394E-10 7.772E-10

229.0313 4.170E-10 6.677E-10 6.804E-10 7.680E-10

229.2500 4.192E-10 5.939E-10 6.649E-10 7.663E-10

229.4688 4.214E-10 5.404E-10 6.794E-10 7.602E-10

229.6875 4.225E-10 5.050E-10 7.020E-10 7.503E-10

229.9063 4.228E-10 4.819E-10 7.139E-10 7.402E-10

230.1250 4.228E-10 4.664E-10 7.058E-10 7.283E-10

230.3438 4.221E-10 4.587E-10 6.838E-10 7.104E-10

230.5625 4.190E-10 4.632E-10 6.651E-10 6.888E-10

230.7813 4.136E-10 4.804E-10 6.644E-10 6.752E-10

231.0000 4.079E-10 5.013E-10 6.804E-10 6.785E-10

231.2188 4.045E-10 5.141E-10 6.989E-10 6.928E-10

231.4375 4.045E-10 5.163E-10 7.063E-10 7.041E-10

231.6563 4.076E-10 5.177E-10 7.026E-10 7.073E-10

231.8750 4.133E-10 5.302E-10 6.983E-10 7.083E-10

232.0938 4.199E-10 5.548E-10 7.006E-10 7.114E-10

232.3125 4.251E-10 5.805E-10 7.066E-10 7.136E-10

232.5313 4.269E-10 5.962E-10 7.084E-10 7.135E-10

232.7500 4.265E-10 5.994E-10 7.041E-10 7.186E-10

232.9688 4.268E-10 5.949E-10 6.975E-10 7.367E-10

233.1875 4.293E-10 5.876E-10 6.921E-10 7.621E-10

233.4063 4.323E-10 5.808E-10 6.868E-10 7.759E-10

233.6250 4.332E-10 5.774E-10 6.805E-10 7.603E-10

233.8438 4.305E-10 5.791E-10 6.752E-10 7.153E-10

234.0625 4.243E-10 5.829E-10 6.754E-10 6.614E-10

234.2813 4.158E-10 5.829E-10 6.834E-10 6.260E-10

234.5000 4.066E-10 5.769E-10 6.942E-10 6.245E-10

234.7188 3.991E-10 5.686E-10 6.953E-10 6.515E-10

234.9375 3.953E-10 5.640E-10 6.753E-10 6.871E-10

235.1563 3.963E-10 5.650E-10 6.359E-10 7.104E-10

235.3750 4.016E-10 5.703E-10 5.934E-10 7.111E-10

235.5938 4.094E-10 5.773E-10 5.661E-10 6.968E-10

235.8125 4.167E-10 5.839E-10 5.616E-10 6.874E-10

236.0313 4.202E-10 5.870E-10 5.776E-10 6.974E-10

236.2500 4.186E-10 5.823E-10 6.081E-10 7.206E-10

236.4688 4.132E-10 5.684E-10 6.448E-10 7.365E-10

236.6875 4.077E-10 5.496E-10 6.758E-10 7.328E-10

236.9063 4.046E-10 5.356E-10 6.919E-10 7.182E-10

237.1250 4.037E-10 5.339E-10 6.953E-10 7.107E-10

237.3438 4.033E-10 5.432E-10 6.976E-10 7.184E-10

237.5625 4.020E-10 5.549E-10 7.083E-10 7.345E-10

237.7813 4.004E-10 5.614E-10 7.280E-10 7.484E-10

238.0000 3.998E-10 5.621E-10 7.505E-10 7.549E-10

238.2188 4.013E-10 5.618E-10 7.654E-10 7.594E-10

238.4375 4.037E-10 5.630E-10 7.589E-10 7.836E-10

238.6563 4.063E-10 5.670E-10 7.208E-10 8.659E-10

238.8750 4.173E-10 5.898E-10 6.646E-10 1.033E-09

239.0938 4.657E-10 6.777E-10 6.447E-10 1.250E-09

239.3125 5.892E-10 8.828E-10 7.349E-10 1.414E-09

239.5313 7.898E-10 1.189E-09 9.597E-10 1.421E-09

239.7500 9.963E-10 1.467E-09 1.235E-09 1.261E-09

239.9688 1.095E-09 1.548E-09 1.403E-09 1.028E-09

240.1875 1.024E-09 1.371E-09 1.352E-09 8.374E-10

240.4063 8.270E-10 1.041E-09 1.122E-09 7.445E-10

240.6250 6.170E-10 7.387E-10 8.547E-10 7.319E-10

240.8438 4.783E-10 5.691E-10 6.757E-10 7.570E-10

241.0625 4.207E-10 5.230E-10 6.077E-10 7.867E-10

241.2813 4.084E-10 5.410E-10 6.012E-10 7.985E-10

241.5000 4.082E-10 5.804E-10 6.080E-10 7.783E-10

241.7188 4.077E-10 6.236E-10 6.147E-10 7.265E-10

241.9375 4.069E-10 6.575E-10 6.276E-10 6.610E-10

242.1563 4.074E-10 6.697E-10 6.485E-10 6.054E-10

242.3750 4.089E-10 6.584E-10 6.687E-10 5.755E-10

242.5938 4.101E-10 6.345E-10 6.778E-10 5.753E-10

242.8125 4.112E-10 6.107E-10 6.725E-10 6.002E-10

243.0313 4.140E-10 5.919E-10 6.579E-10 6.395E-10

243.2500 4.191E-10 5.786E-10 6.417E-10 6.790E-10

243.4688 4.248E-10 5.740E-10 6.281E-10 7.073E-10

243.6875 4.276E-10 5.850E-10 6.174E-10 7.203E-10

243.9063 4.258E-10 6.136E-10 6.101E-10 7.213E-10

244.1250 4.207E-10 6.498E-10 6.097E-10 7.154E-10

244.3438 4.151E-10 6.750E-10 6.203E-10 7.051E-10

244.5625 4.120E-10 6.759E-10 6.414E-10 6.922E-10

244.7813 4.124E-10 6.544E-10 6.632E-10 6.807E-10

245.0000 4.156E-10 6.240E-10 6.730E-10 6.759E-10

245.2188 4.188E-10 5.969E-10 6.675E-10 6.774E-10

245.4375 4.191E-10 5.757E-10 6.583E-10 6.773E-10

245.6563 4.152E-10 5.571E-10 6.590E-10 6.680E-10

245.8750 4.081E-10 5.403E-10 6.681E-10 6.516E-10

246.0938 4.009E-10 5.299E-10 6.720E-10 6.372E-10

246.3125 3.965E-10 5.312E-10 6.621E-10 6.292E-10

246.5313 3.956E-10 5.429E-10 6.455E-10 6.241E-10

246.7500 3.969E-10 5.554E-10 6.380E-10 6.187E-10

246.9688 3.985E-10 5.577E-10 6.499E-10 6.173E-10

247.1875 3.996E-10 5.457E-10 6.804E-10 6.260E-10

247.4063 4.003E-10 5.244E-10 7.175E-10 6.432E-10

247.6250 4.016E-10 5.028E-10 7.469E-10 6.591E-10

247.8438 4.045E-10 4.910E-10 7.619E-10 6.671E-10

248.0625 4.086E-10 4.973E-10 7.680E-10 6.677E-10

248.2813 4.121E-10 5.240E-10 7.727E-10 6.630E-10

248.5000 4.128E-10 5.633E-10 7.737E-10 6.511E-10

248.7188 4.113E-10 5.986E-10 7.622E-10 6.302E-10

248.9375 4.112E-10 6.137E-10 7.377E-10 6.048E-10

249.1563 4.179E-10 6.058E-10 7.169E-10 5.848E-10

249.3750 4.334E-10 5.894E-10 7.206E-10 5.786E-10

249.5938 4.542E-10 5.872E-10 7.518E-10 5.865E-10

249.8125 4.714E-10 6.110E-10 7.884E-10 6.021E-10

250.0313 4.766E-10 6.495E-10 8.021E-10 6.193E-10

250.2500 4.687E-10 6.765E-10 7.856E-10 6.361E-10

250.4688 4.539E-10 6.735E-10 7.561E-10 6.530E-10

250.6875 4.405E-10 6.443E-10 7.330E-10 6.693E-10

250.9063 4.318E-10 6.071E-10 7.168E-10 6.830E-10

251.1250 4.258E-10 5.771E-10 6.950E-10 6.926E-10

251.3438 4.197E-10 5.573E-10 6.638E-10 6.967E-10

251.5625 4.135E-10 5.437E-10 6.356E-10 6.931E-10

251.7813 4.087E-10 5.324E-10 6.238E-10 6.812E-10

252.0000 4.060E-10 5.209E-10 6.259E-10 6.650E-10

252.2188 4.049E-10 5.075E-10 6.250E-10 6.522E-10

252.4375 4.037E-10 4.923E-10 6.061E-10 6.484E-10

252.6563 4.011E-10 4.780E-10 5.719E-10 6.538E-10

252.8750 3.964E-10 4.688E-10 5.411E-10 6.618E-10

253.0938 3.913E-10 4.691E-10 5.333E-10 6.638E-10

253.3125 3.891E-10 4.822E-10 5.527E-10 6.571E-10

253.5313 3.935E-10 5.085E-10 5.873E-10 6.484E-10

253.7500 4.049E-10 5.420E-10 6.197E-10 6.456E-10

253.9688 4.193E-10 5.690E-10 6.378E-10 6.475E-10

254.1875 4.298E-10 5.759E-10 6.371E-10 6.457E-10

254.4063 4.325E-10 5.602E-10 6.212E-10 6.376E-10

254.6250 4.278E-10 5.336E-10 6.028E-10 6.303E-10

254.8438 4.194E-10 5.119E-10 5.974E-10 6.307E-10

255.0625 4.101E-10 5.025E-10 6.114E-10 6.363E-10

255.2813 4.017E-10 5.020E-10 6.368E-10 6.409E-10

255.5000 3.954E-10 5.046E-10 6.605E-10 6.446E-10

255.7188 3.919E-10 5.094E-10 6.762E-10 6.528E-10

255.9375 3.908E-10 5.188E-10 6.849E-10 6.663E-10

256.1563 3.914E-10 5.323E-10 6.875E-10 6.771E-10

256.3750 3.931E-10 5.435E-10 6.834E-10 6.753E-10

256.5938 3.955E-10 5.466E-10 6.750E-10 6.578E-10

256.8125 3.985E-10 5.435E-10 6.699E-10 6.312E-10

257.0313 4.021E-10 5.435E-10 6.737E-10 6.081E-10

257.2500 4.062E-10 5.528E-10 6.860E-10 5.996E-10

257.4688 4.083E-10 5.663E-10 7.030E-10 6.078E-10

257.6875 4.060E-10 5.729E-10 7.210E-10 6.236E-10

257.9063 4.002E-10 5.700E-10 7.349E-10 6.336E-10

258.1250 3.965E-10 5.675E-10 7.372E-10 6.321E-10

258.3438 4.010E-10 5.751E-10 7.244E-10 6.270E-10

258.5625 4.133E-10 5.900E-10 7.045E-10 6.324E-10

258.7813 4.253E-10 5.991E-10 6.933E-10 6.537E-10

259.0000 4.290E-10 5.927E-10 6.995E-10 6.813E-10

259.2188 4.236E-10 5.709E-10 7.160E-10 6.994E-10

259.4375 4.151E-10 5.409E-10 7.251E-10 6.998E-10

259.6563 4.095E-10 5.126E-10 7.143E-10 6.870E-10

259.8750 4.074E-10 4.954E-10 6.850E-10 6.713E-10

260.0938 4.058E-10 4.942E-10 6.475E-10 6.596E-10

260.3125 4.029E-10 5.055E-10 6.116E-10 6.521E-10

260.5313 3.994E-10 5.217E-10 5.844E-10 6.477E-10

260.7500 3.976E-10 5.383E-10 5.739E-10 6.490E-10

260.9688 3.991E-10 5.531E-10 5.849E-10 6.572E-10

261.1875 4.034E-10 5.612E-10 6.114E-10 6.657E-10

261.4063 4.087E-10 5.569E-10 6.364E-10 6.650E-10

261.6250 4.121E-10 5.438E-10 6.453E-10 6.540E-10

261.8438 4.130E-10 5.338E-10 6.390E-10 6.419E-10

262.0625 4.132E-10 5.344E-10 6.318E-10 6.382E-10

262.2813 4.151E-10 5.404E-10 6.359E-10 6.454E-10

262.5000 4.202E-10 5.425E-10 6.484E-10 6.630E-10

262.7188 4.297E-10 5.398E-10 6.546E-10 6.894E-10

262.9375 4.465E-10 5.405E-10 6.434E-10 7.146E-10

263.1563 4.762E-10 5.580E-10 6.239E-10 7.227E-10

263.3750 5.268E-10 6.139E-10 6.311E-10 7.137E-10

263.5938 6.098E-10 7.354E-10 7.094E-10 7.193E-10

263.8125 7.306E-10 9.284E-10 8.746E-10 7.781E-10

264.0313 8.671E-10 1.140E-09 1.080E-09 8.861E-10

264.2500 9.636E-10 1.267E-09 1.229E-09 9.837E-10

264.4688 9.626E-10 1.230E-09 1.236E-09 1.004E-09

264.6875 8.548E-10 1.046E-09 1.099E-09 9.321E-10

264.9063 6.919E-10 8.188E-10 8.944E-10 8.129E-10

265.1250 5.454E-10 6.467E-10 7.167E-10 7.029E-10

265.3438 4.539E-10 5.563E-10 6.169E-10 6.274E-10

265.5625 4.122E-10 5.121E-10 5.881E-10 5.805E-10

265.7813 3.968E-10 4.759E-10 5.967E-10 5.520E-10

266.0000 3.912E-10 4.427E-10 6.160E-10 5.425E-10

266.2188 3.885E-10 4.313E-10 6.380E-10 5.540E-10

266.4375 3.859E-10 4.563E-10 6.636E-10 5.789E-10

266.6563 3.828E-10 5.110E-10 6.881E-10 6.050E-10

266.8750 3.814E-10 5.712E-10 6.989E-10 6.282E-10

267.0938 3.845E-10 6.112E-10 6.856E-10 6.508E-10

267.3125 3.912E-10 6.201E-10 6.498E-10 6.702E-10

267.5313 3.972E-10 6.062E-10 6.048E-10 6.761E-10

267.7500 3.985E-10 5.872E-10 5.684E-10 6.639E-10

267.9688 3.948E-10 5.754E-10 5.555E-10 6.450E-10

268.1875 3.887E-10 5.696E-10 5.698E-10 6.374E-10

268.4063 3.827E-10 5.600E-10 6.008E-10 6.503E-10

268.6250 3.785E-10 5.402E-10 6.308E-10 6.821E-10

268.8438 3.773E-10 5.151E-10 6.472E-10 7.272E-10

269.0625 3.807E-10 4.954E-10 6.496E-10 7.779E-10

269.2813 3.887E-10 4.867E-10 6.465E-10 8.197E-10

269.5000 3.984E-10 4.867E-10 6.467E-10 8.337E-10

269.7188 4.060E-10 4.925E-10 6.529E-10 8.096E-10

269.9375 4.093E-10 5.064E-10 6.600E-10 7.554E-10

270.1563 4.108E-10 5.323E-10 6.606E-10 6.936E-10

270.3750 4.160E-10 5.679E-10 6.500E-10 6.454E-10

270.5938 4.302E-10 6.011E-10 6.305E-10 6.184E-10

270.8125 4.541E-10 6.165E-10 6.102E-10 6.076E-10

271.0313 4.807E-10 6.075E-10 5.969E-10 6.052E-10

271.2500 4.978E-10 5.837E-10 5.917E-10 6.086E-10

271.4688 4.964E-10 5.632E-10 5.878E-10 6.192E-10

271.6875 4.778E-10 5.560E-10 5.769E-10 6.376E-10

271.9063 4.526E-10 5.570E-10 5.566E-10 6.604E-10

272.1250 4.319E-10 5.557E-10 5.338E-10 6.803E-10

272.3438 4.190E-10 5.484E-10 5.214E-10 6.888E-10

272.5625 4.116E-10 5.396E-10 5.299E-10 6.843E-10

272.7813 4.059E-10 5.367E-10 5.614E-10 6.805E-10

273.0000 4.001E-10 5.479E-10 6.110E-10 6.979E-10

273.2188 3.934E-10 5.772E-10 6.726E-10 7.403E-10

273.4375 3.856E-10 6.167E-10 7.358E-10 7.836E-10

273.6563 3.777E-10 6.470E-10 7.822E-10 7.971E-10

273.8750 3.727E-10 6.526E-10 7.941E-10 7.732E-10

274.0938 3.743E-10 6.350E-10 7.749E-10 7.317E-10

274.3125 3.851E-10 6.072E-10 7.517E-10 6.955E-10

274.5313 4.034E-10 5.806E-10 7.496E-10 6.716E-10

274.7500 4.222E-10 5.611E-10 7.670E-10 6.541E-10

274.9688 4.324E-10 5.535E-10 7.799E-10 6.352E-10

275.1875 4.297E-10 5.602E-10 7.687E-10 6.093E-10

275.4063 4.185E-10 5.742E-10 7.336E-10 5.761E-10

275.6250 4.076E-10 5.804E-10 6.902E-10 5.444E-10

275.8438 4.030E-10 5.666E-10 6.574E-10 5.301E-10

276.0625 4.035E-10 5.330E-10 6.461E-10 5.420E-10

276.2813 4.045E-10 4.916E-10 6.513E-10 5.683E-10

276.5000 4.032E-10 4.573E-10 6.565E-10 5.841E-10

276.7188 4.007E-10 4.397E-10 6.495E-10 5.749E-10

276.9375 3.985E-10 4.392E-10 6.341E-10 5.527E-10

277.1563 3.966E-10 4.491E-10 6.233E-10 5.482E-10

277.3750 3.936E-10 4.612E-10 6.222E-10 5.815E-10

277.5938 3.897E-10 4.715E-10 6.236E-10 6.410E-10

277.8125 3.869E-10 4.829E-10 6.191E-10 6.924E-10

278.0313 3.873E-10 4.999E-10 6.114E-10 7.099E-10

278.2500 3.902E-10 5.224E-10 6.127E-10 6.974E-10

278.4688 3.923E-10 5.459E-10 6.303E-10 6.764E-10

278.6875 3.898E-10 5.652E-10 6.568E-10 6.617E-10

278.9063 3.824E-10 5.764E-10 6.750E-10 6.538E-10

279.1250 3.730E-10 5.753E-10 6.751E-10 6.501E-10

279.3438 3.666E-10 5.585E-10 6.625E-10 6.508E-10

279.5625 3.666E-10 5.299E-10 6.502E-10 6.547E-10

279.7813 3.735E-10 5.023E-10 6.454E-10 6.599E-10

280.0000 3.847E-10 4.886E-10 6.447E-10 6.704E-10

280.2188 3.955E-10 4.896E-10 6.397E-10 6.899E-10

280.4375 4.010E-10 4.925E-10 6.239E-10 7.079E-10

280.6563 3.990E-10 4.842E-10 5.991E-10 7.043E-10

280.8750 3.903E-10 4.640E-10 5.760E-10 6.736E-10

281.0938 3.790E-10 4.432E-10 5.661E-10 6.358E-10

281.3125 3.696E-10 4.323E-10 5.719E-10 6.132E-10

281.5313 3.656E-10 4.322E-10 5.873E-10 6.059E-10

281.7500 3.684E-10 4.360E-10 6.062E-10 5.977E-10

281.9688 3.766E-10 4.389E-10 6.262E-10 5.808E-10

282.1875 3.862E-10 4.434E-10 6.454E-10 5.622E-10

282.4063 3.923E-10 4.567E-10 6.593E-10 5.508E-10

282.6250 3.921E-10 4.806E-10 6.642E-10 5.459E-10

282.8438 3.874E-10 5.074E-10 6.611E-10 5.412E-10

283.0625 3.826E-10 5.253E-10 6.524E-10 5.329E-10

283.2813 3.806E-10 5.290E-10 6.379E-10 5.245E-10

283.5000 3.812E-10 5.234E-10 6.163E-10 5.243E-10

283.7188 3.823E-10 5.195E-10 5.921E-10 5.386E-10

283.9375 3.829E-10 5.264E-10 5.772E-10 5.647E-10

284.1563 3.834E-10 5.451E-10 5.823E-10 5.930E-10

284.3750 3.840E-10 5.653E-10 6.061E-10 6.158E-10

284.5938 3.834E-10 5.717E-10 6.354E-10 6.322E-10

284.8125 3.804E-10 5.574E-10 6.564E-10 6.432E-10

285.0313 3.765E-10 5.308E-10 6.657E-10 6.474E-10

285.2500 3.745E-10 5.071E-10 6.694E-10 6.446E-10

285.4688 3.768E-10 4.953E-10 6.736E-10 6.403E-10

285.6875 3.826E-10 4.931E-10 6.758E-10 6.414E-10

285.9063 3.893E-10 4.932E-10 6.657E-10 6.479E-10

286.1250 3.932E-10 4.927E-10 6.374E-10 6.565E-10

286.3438 3.925E-10 4.941E-10 5.987E-10 6.688E-10

286.5625 3.880E-10 4.995E-10 5.674E-10 6.923E-10

286.7813 3.826E-10 5.057E-10 5.559E-10 7.277E-10

287.0000 3.783E-10 5.065E-10 5.617E-10 7.599E-10

287.2188 3.751E-10 4.997E-10 5.718E-10 7.686E-10

287.4375 3.724E-10 4.910E-10 5.776E-10 7.477E-10

287.6563 3.703E-10 4.885E-10 5.826E-10 7.109E-10

287.8750 3.695E-10 4.946E-10 5.942E-10 6.785E-10

288.0938 3.713E-10 5.023E-10 6.110E-10 6.604E-10

288.3125 3.762E-10 5.042E-10 6.202E-10 6.548E-10

288.5313 3.839E-10 5.024E-10 6.124E-10 6.580E-10

288.7500 3.926E-10 5.079E-10 5.940E-10 6.700E-10

288.9688 4.001E-10 5.266E-10 5.817E-10 6.889E-10

289.1875 4.045E-10 5.484E-10 5.856E-10 7.050E-10

289.4063 4.048E-10 5.566E-10 6.002E-10 7.052E-10

289.6250 4.015E-10 5.470E-10 6.144E-10 6.833E-10

289.8438 3.966E-10 5.328E-10 6.227E-10 6.455E-10

290.0625 3.919E-10 5.285E-10 6.266E-10 6.051E-10

290.2813 3.877E-10 5.343E-10 6.278E-10 5.758E-10

290.5000 3.833E-10 5.394E-10 6.241E-10 5.674E-10

290.7188 3.801E-10 5.366E-10 6.142E-10 5.821E-10

290.9375 3.804E-10 5.277E-10 5.994E-10 6.123E-10

291.1563 3.842E-10 5.160E-10 5.813E-10 6.448E-10

291.3750 3.873E-10 4.998E-10 5.581E-10 6.697E-10

291.5938 3.859E-10 4.746E-10 5.296E-10 6.836E-10

291.8125 3.806E-10 4.415E-10 5.044E-10 6.860E-10

292.0313 3.756E-10 4.096E-10 4.969E-10 6.754E-10

292.2500 3.743E-10 3.910E-10 5.126E-10 6.527E-10

292.4688 3.762E-10 3.933E-10 5.396E-10 6.260E-10

292.6875 3.788E-10 4.155E-10 5.599E-10 6.076E-10

292.9063 3.807E-10 4.499E-10 5.664E-10 6.064E-10

293.1250 3.823E-10 4.871E-10 5.658E-10 6.205E-10

293.3438 3.853E-10 5.199E-10 5.650E-10 6.377E-10

293.5625 3.897E-10 5.450E-10 5.630E-10 6.463E-10

293.7813 3.929E-10 5.614E-10 5.578E-10 6.451E-10

294.0000 3.921E-10 5.698E-10 5.562E-10 6.425E-10

294.2188 3.871E-10 5.710E-10 5.664E-10 6.440E-10

294.4375 3.804E-10 5.658E-10 5.848E-10 6.452E-10

294.6563 3.753E-10 5.571E-10 5.988E-10 6.397E-10

294.8750 3.734E-10 5.490E-10 6.013E-10 6.277E-10

295.0938 3.742E-10 5.433E-10 5.969E-10 6.144E-10

295.3125 3.761E-10 5.354E-10 5.924E-10 6.042E-10

295.5313 3.769E-10 5.184E-10 5.887E-10 5.999E-10

295.7500 3.750E-10 4.923E-10 5.841E-10 6.029E-10

295.9688 3.712E-10 4.677E-10 5.817E-10 6.141E-10

296.1875 3.688E-10 4.568E-10 5.894E-10 6.350E-10

296.4063 3.689E-10 4.604E-10 6.096E-10 6.634E-10

296.6250 3.699E-10 4.678E-10 6.326E-10 6.960E-10

296.8438 3.718E-10 4.675E-10 6.448E-10 7.264E-10

297.0625 3.749E-10 4.539E-10 6.389E-10 7.285E-10

297.2813 3.785E-10 4.349E-10 6.173E-10 6.931E-10

297.5000 3.856E-10 4.311E-10 5.964E-10 6.833E-10

297.7188 3.932E-10 4.410E-10 5.878E-10 6.863E-10

297.9375 3.922E-10 4.430E-10 5.815E-10 6.653E-10

298.1563 4.131E-10 4.711E-10 5.956E-10 2.015E-09

298.3750 4.650E-10 5.211E-10 6.537E-10 9.638E-09

298.5938 4.265E-10 4.614E-10 6.444E-10 3.012E-08

298.8125 7.881E-10 1.148E-09 9.347E-10 6.371E-08

299.0313 4.557E-09 6.675E-09 4.395E-09 9.776E-08

299.2500 1.749E-08 2.374E-08 1.719E-08 1.128E-07

299.4688 4.301E-08 5.461E-08 4.387E-08 9.883E-08

299.6875 7.482E-08 8.927E-08 7.888E-08 6.506E-08

299.9063 9.701E-08 1.087E-07 1.052E-07 3.101E-08

300.1250 9.581E-08 1.001E-07 1.064E-07 9.938E-09

300.3438 7.206E-08 6.933E-08 8.162E-08 2.045E-09

300.5625 4.041E-08 3.488E-08 4.647E-08 6.845E-10

300.7813 1.607E-08 1.183E-08 1.864E-08 7.111E-10

301.0000 4.161E-09 2.390E-09 4.832E-09 6.470E-10

301.2188 7.830E-10 5.018E-10 9.697E-10 5.889E-10

301.4375 4.292E-10 5.165E-10 6.534E-10 6.002E-10

301.6563 4.330E-10 4.895E-10 6.940E-10 6.115E-10

301.8750 3.927E-10 4.243E-10 6.391E-10 6.061E-10

302.0938 3.888E-10 4.223E-10 6.207E-10 5.970E-10

302.3125 3.947E-10 4.266E-10 6.129E-10 5.947E-10

302.5313 3.881E-10 4.258E-10 5.907E-10 6.086E-10

302.7500 3.823E-10 4.352E-10 5.758E-10 6.347E-10

302.9688 3.795E-10 4.480E-10 5.726E-10 6.564E-10

303.1875 3.770E-10 4.555E-10 5.736E-10 6.613E-10

303.4063 3.763E-10 4.570E-10 5.780E-10 6.476E-10

303.6250 3.780E-10 4.546E-10 5.848E-10 6.225E-10

303.8438 3.808E-10 4.545E-10 5.925E-10 6.011E-10

304.0625 3.838E-10 4.634E-10 5.998E-10 6.006E-10

304.2813 3.855E-10 4.835E-10 6.017E-10 6.288E-10

304.5000 3.858E-10 5.119E-10 5.915E-10 6.754E-10

304.7188 3.866E-10 5.405E-10 5.698E-10 7.135E-10

304.9375 3.888E-10 5.587E-10 5.451E-10 7.183E-10

305.1563 3.907E-10 5.612E-10 5.286E-10 6.901E-10

305.3750 3.907E-10 5.502E-10 5.265E-10 6.539E-10

305.5938 3.884E-10 5.300E-10 5.365E-10 6.315E-10

305.8125 3.853E-10 5.038E-10 5.511E-10 6.216E-10

306.0313 3.829E-10 4.746E-10 5.621E-10 6.096E-10

306.2500 3.808E-10 4.453E-10 5.632E-10 5.889E-10

306.4688 3.776E-10 4.197E-10 5.555E-10 5.650E-10

306.6875 3.726E-10 4.033E-10 5.482E-10 5.436E-10

306.9063 3.673E-10 4.015E-10 5.495E-10 5.250E-10

307.1250 3.636E-10 4.145E-10 5.554E-10 5.108E-10

307.3438 3.623E-10 4.371E-10 5.549E-10 5.083E-10

307.5625 3.623E-10 4.603E-10 5.448E-10 5.231E-10

307.7813 3.617E-10 4.766E-10 5.354E-10 5.501E-10

308.0000 3.601E-10 4.837E-10 5.389E-10 5.775E-10

308.2188 3.589E-10 4.867E-10 5.586E-10 5.974E-10

308.4375 3.595E-10 4.910E-10 5.873E-10 6.096E-10

308.6563 3.619E-10 4.965E-10 6.151E-10 6.161E-10

308.8750 3.652E-10 4.978E-10 6.363E-10 6.151E-10

309.0938 3.679E-10 4.923E-10 6.518E-10 6.036E-10

309.3125 3.699E-10 4.859E-10 6.656E-10 5.845E-10

309.5313 3.723E-10 4.875E-10 6.757E-10 5.688E-10

309.7500 3.762E-10 4.987E-10 6.717E-10 5.693E-10

309.9688 3.825E-10 5.126E-10 6.461E-10 5.918E-10

310.1875 3.910E-10 5.208E-10 6.064E-10 6.295E-10

310.4063 3.994E-10 5.208E-10 5.712E-10 6.628E-10

310.6250 4.040E-10 5.167E-10 5.541E-10 6.723E-10

310.8438 4.028E-10 5.134E-10 5.527E-10 6.557E-10

311.0625 3.964E-10 5.113E-10 5.544E-10 6.290E-10

311.2813 3.890E-10 5.072E-10 5.514E-10 6.099E-10

311.5000 3.856E-10 4.970E-10 5.475E-10 6.011E-10

311.7188 3.880E-10 4.766E-10 5.513E-10 5.934E-10

311.9375 3.934E-10 4.455E-10 5.650E-10 5.812E-10

312.1563 3.979E-10 4.127E-10 5.818E-10 5.682E-10

312.3750 3.980E-10 3.955E-10 5.919E-10 5.609E-10

312.5938 3.936E-10 4.072E-10 5.930E-10 5.606E-10

312.8125 3.877E-10 4.433E-10 5.932E-10 5.633E-10

313.0313 3.835E-10 4.823E-10 6.012E-10 5.631E-10

313.2500 3.825E-10 5.043E-10 6.159E-10 5.580E-10

313.4688 3.844E-10 5.076E-10 6.276E-10 5.521E-10

313.6875 3.878E-10 5.010E-10 6.303E-10 5.502E-10

313.9063 3.918E-10 4.894E-10 6.306E-10 5.532E-10

314.1250 3.983E-10 4.737E-10 6.393E-10 5.586E-10

314.3438 4.085E-10 4.584E-10 6.560E-10 5.638E-10

314.5625 4.208E-10 4.518E-10 6.692E-10 5.717E-10

314.7813 4.310E-10 4.580E-10 6.722E-10 5.886E-10

315.0000 4.340E-10 4.730E-10 6.678E-10 6.117E-10

315.2188 4.279E-10 4.912E-10 6.604E-10 6.262E-10

315.4375 4.185E-10 5.109E-10 6.503E-10 6.199E-10

315.6563 4.135E-10 5.295E-10 6.390E-10 5.975E-10

315.8750 4.165E-10 5.423E-10 6.347E-10 5.768E-10

316.0938 4.277E-10 5.486E-10 6.494E-10 5.759E-10

316.3125 4.469E-10 5.584E-10 6.870E-10 6.049E-10

316.5313 4.764E-10 5.861E-10 7.390E-10 6.660E-10

316.7500 5.180E-10 6.332E-10 7.910E-10 7.485E-10

316.9688 5.612E-10 6.735E-10 8.281E-10 8.217E-10

317.1875 5.827E-10 6.712E-10 8.350E-10 8.480E-10

317.4063 5.666E-10 6.162E-10 8.003E-10 8.130E-10

317.6250 5.187E-10 5.323E-10 7.271E-10 7.369E-10

317.8438 4.616E-10 4.537E-10 6.401E-10 6.551E-10

318.0625 4.184E-10 4.049E-10 5.739E-10 5.935E-10

318.2813 3.958E-10 3.934E-10 5.460E-10 5.597E-10

318.5000 3.849E-10 4.134E-10 5.485E-10 5.527E-10

318.7188 3.784E-10 4.534E-10 5.665E-10 5.721E-10

318.9375 3.743E-10 4.977E-10 5.876E-10 6.115E-10

319.1563 3.699E-10 5.296E-10 5.997E-10 6.530E-10

319.3750 3.648E-10 5.413E-10 5.955E-10 6.785E-10

319.5938 3.610E-10 5.349E-10 5.779E-10 6.801E-10

319.8125 3.598E-10 5.190E-10 5.589E-10 6.626E-10

320.0313 3.644E-10 5.071E-10 5.530E-10 6.398E-10

320.2500 3.744E-10 5.063E-10 5.624E-10 6.228E-10

320.4688 3.821E-10 5.084E-10 5.752E-10 6.130E-10

320.6875 3.853E-10 5.020E-10 5.821E-10 6.095E-10

320.9063 3.864E-10 4.834E-10 5.825E-10 6.067E-10

321.1250 3.833E-10 4.571E-10 5.765E-10 5.916E-10

321.3438 3.775E-10 4.371E-10 5.677E-10 5.615E-10

321.5625 3.732E-10 4.336E-10 5.619E-10 5.324E-10

321.7813 3.666E-10 4.383E-10 5.565E-10 5.191E-10

322.0000 3.582E-10 4.402E-10 5.500E-10 5.263E-10

322.2188 3.550E-10 4.386E-10 5.474E-10 5.503E-10

322.4375 3.545E-10 4.363E-10 5.493E-10 5.781E-10

322.6563 3.548E-10 4.408E-10 5.532E-10 5.971E-10

322.8750 3.594E-10 4.561E-10 5.541E-10 6.007E-10

323.0938 3.621E-10 4.703E-10 5.457E-10 5.873E-10

323.3125 3.613E-10 4.758E-10 5.383E-10 5.691E-10

323.5313 3.644E-10 4.792E-10 5.503E-10 5.610E-10

323.7500 3.668E-10 4.796E-10 5.762E-10 5.611E-10

323.9688 3.676E-10 4.758E-10 6.037E-10 5.675E-10

324.1875 3.779E-10 4.769E-10 6.388E-10 5.862E-10

324.4063 3.921E-10 4.853E-10 6.845E-10 6.112E-10

324.6250 4.054E-10 5.051E-10 7.313E-10 6.381E-10

324.8438 4.254E-10 5.417E-10 7.678E-10 6.706E-10

325.0625 4.340E-10 5.711E-10 7.754E-10 6.881E-10

325.2813 4.172E-10 5.707E-10 7.519E-10 6.694E-10

325.5000 3.981E-10 5.514E-10 7.162E-10 6.283E-10

325.7188 3.819E-10 5.209E-10 6.729E-10 5.799E-10

325.9375 3.634E-10 4.908E-10 6.320E-10 5.343E-10

326.1563 3.625E-10 4.913E-10 6.226E-10 5.114E-10

326.3750 3.672E-10 5.101E-10 6.308E-10 5.012E-10

326.5938 3.585E-10 5.181E-10 6.258E-10 4.855E-10

326.8125 3.638E-10 5.292E-10 6.227E-10 4.844E-10

327.0313 3.741E-10 5.381E-10 6.215E-10 5.000E-10

327.2500 3.639E-10 5.232E-10 6.029E-10 5.131E-10

327.4688 3.704E-10 5.133E-10 5.876E-10 5.334E-10

327.6875 3.838E-10 5.147E-10 5.736E-10 5.463E-10

327.9063 3.614E-10 5.073E-10 5.415E-10 5.325E-10

328.1250 3.549E-10 5.184E-10 5.286E-10 5.351E-10

328.3438 3.695E-10 5.249E-10 5.308E-10 5.550E-10

328.5625 3.503E-10 4.818E-10 5.094E-10 5.552E-10

328.7813 3.578E-10 4.641E-10 5.182E-10 5.792E-10

329.0000 3.926E-10 4.954E-10 5.675E-10 6.156E-10

329.2188 3.678E-10 5.058E-10 5.931E-10 5.896E-10

329.4375 3.702E-10 5.383E-10 6.363E-10 5.656E-10

329.6563 4.193E-10 5.776E-10 6.882E-10 5.823E-10

329.8750 3.834E-10 5.106E-10 6.563E-10 5.612E-10

330.0938 3.772E-10 4.504E-10 6.427E-10 5.652E-10

330.3125 4.491E-10 4.677E-10 6.883E-10 6.129E-10

330.5313 4.008E-10 4.222E-10 6.366E-10 5.696E-10

330.7500 3.838E-10 4.289E-10 5.946E-10 5.480E-10

330.9688 4.921E-10 5.460E-10 6.578E-10 6.276E-10

331.1875 4.176E-10 5.205E-10 6.223E-10 5.960E-10

331.4063 3.779E-10 5.240E-10 6.141E-10 5.657E-10

331.6250 5.675E-10 6.982E-10 7.385E-10 6.644E-10

331.8438 4.780E-10 6.113E-10 6.321E-10 6.005E-10

332.0625 3.792E-10 4.686E-10 5.097E-10 5.734E-10

332.2813 6.680E-10 6.296E-10 6.922E-10 7.951E-10

332.5000 5.596E-10 5.338E-10 6.394E-10 7.012E-10

332.7188 4.077E-10 4.686E-10 5.541E-10 5.628E-10

332.9375 9.331E-10 9.516E-10 9.136E-10 9.063E-10

333.1563 7.816E-10 8.388E-10 8.231E-10 8.206E-10

333.3750 4.010E-10 4.635E-10 6.364E-10 5.803E-10

333.5938 1.288E-09 1.098E-09 1.310E-09 1.181E-09

333.8125 1.130E-09 1.011E-09 1.148E-09 1.082E-09

334.0313 3.915E-10 5.065E-10 6.180E-10 5.902E-10

334.2500 2.105E-09 1.866E-09 1.955E-09 1.727E-09

334.4688 2.027E-09 1.781E-09 1.944E-09 1.674E-09

334.6875 3.964E-10 5.205E-10 5.465E-10 5.980E-10

334.9063 3.870E-09 3.303E-09 2.726E-09 2.899E-09

335.1250 4.234E-09 3.578E-09 3.046E-09 3.073E-09

335.3438 4.008E-10 4.288E-10 4.952E-10 5.667E-10

335.5625 8.165E-09 6.329E-09 5.789E-09 6.025E-09

335.7813 1.042E-08 8.054E-09 7.193E-09 7.658E-09

336.0000 3.902E-10 4.455E-10 4.866E-10 5.510E-10

336.2188 1.977E-08 1.606E-08 1.466E-08 1.287E-08

336.4375 3.002E-08 2.415E-08 2.180E-08 1.960E-08

336.6563 5.136E-10 5.955E-10 6.304E-10 5.654E-10

336.8750 5.988E-08 4.690E-08 4.266E-08 4.131E-08

337.0938 1.108E-07 8.688E-08 7.902E-08 7.575E-08

337.3125 2.486E-09 2.037E-09 2.167E-09 2.192E-09

337.5313 2.431E-07 1.919E-07 1.702E-07 1.612E-07

337.7500 5.799E-07 4.571E-07 4.071E-07 3.859E-07

337.9688 2.854E-08 2.291E-08 2.022E-08 1.913E-08

338.1875 1.658E-06 1.301E-06 1.168E-06 1.110E-06

338.4063 5.850E-06 4.593E-06 4.118E-06 3.912E-06

338.6250 7.100E-07 5.561E-07 4.994E-07 4.747E-07

338.8438 3.801E-05 2.990E-05 2.677E-05 2.542E-05

339.0625 4.074E-04 3.203E-04 2.869E-04 2.724E-04

339.2813 1.621E-03 1.275E-03 1.142E-03 1.084E-03

339.5000 3.911E-03 3.075E-03 2.754E-03 2.615E-03

339.7188 6.600E-03 5.189E-03 4.647E-03 4.413E-03

339.9375 8.244E-03 6.481E-03 5.805E-03 5.512E-03

340.1563 7.777E-03 6.114E-03 5.476E-03 5.200E-03

340.3750 5.512E-03 4.334E-03 3.882E-03 3.686E-03

340.5938 2.839E-03 2.232E-03 1.999E-03 1.898E-03

340.8125 9.773E-04 7.684E-04 6.882E-04 6.534E-04

341.0313 1.793E-04 1.410E-04 1.263E-04 1.199E-04

341.2500 5.607E-06 4.418E-06 3.954E-06 3.746E-06

341.4688 4.200E-06 3.294E-06 2.953E-06 2.812E-06

341.6875 4.516E-06 3.544E-06 3.177E-06 3.023E-06

341.9063 3.715E-07 2.911E-07 2.616E-07 2.493E-07

342.1250 3.106E-07 2.447E-07 2.187E-07 2.077E-07

342.3438 5.370E-07 4.219E-07 3.773E-07 3.593E-07

342.5625 6.862E-08 5.378E-08 4.804E-08 4.623E-08

342.7813 4.859E-08 3.871E-08 3.507E-08 3.278E-08

343.0000 1.146E-07 9.070E-08 8.176E-08 7.700E-08

343.2188 1.980E-08 1.589E-08 1.456E-08 1.368E-08

343.4375 1.151E-08 9.169E-09 8.366E-09 7.832E-09

343.6563 3.452E-08 2.727E-08 2.456E-08 2.297E-08

343.8750 7.841E-09 6.327E-09 5.865E-09 5.347E-09

344.0938 3.379E-09 2.832E-09 2.790E-09 2.760E-09

344.3125 1.252E-08 1.003E-08 9.259E-09 8.928E-09

344.5313 3.570E-09 3.007E-09 2.914E-09 2.753E-09

344.7500 1.384E-09 1.324E-09 1.252E-09 1.250E-09

344.9688 5.523E-09 4.604E-09 4.062E-09 4.112E-09

345.1875 1.987E-09 1.812E-09 1.585E-09 1.701E-09

345.4063 7.301E-10 7.803E-10 7.922E-10 6.747E-10

345.6250 2.787E-09 2.359E-09 2.303E-09 1.953E-09

345.8438 1.268E-09 1.168E-09 1.229E-09 1.009E-09

346.0625 4.779E-10 5.889E-10 6.476E-10 6.109E-10

346.2813 1.531E-09 1.446E-09 1.379E-09 1.396E-09

346.5000 8.519E-10 8.804E-10 8.990E-10 9.376E-10

346.7188 4.056E-10 4.630E-10 5.793E-10 5.873E-10

346.9375 1.016E-09 9.150E-10 1.001E-09 9.628E-10

347.1563 6.838E-10 6.844E-10 7.538E-10 7.445E-10

347.3750 3.753E-10 4.808E-10 5.180E-10 5.409E-10

347.5938 7.280E-10 7.642E-10 7.591E-10 7.633E-10

347.8125 5.691E-10 6.253E-10 6.672E-10 6.541E-10

348.0313 3.541E-10 4.461E-10 5.553E-10 5.280E-10

348.2500 5.516E-10 5.974E-10 7.300E-10 6.767E-10

348.4688 4.770E-10 5.404E-10 6.934E-10 6.215E-10

348.6875 3.499E-10 4.549E-10 6.000E-10 5.047E-10

348.9063 4.897E-10 5.856E-10 6.866E-10 5.567E-10

349.1250 4.647E-10 5.747E-10 6.688E-10 5.210E-10

349.3438 3.807E-10 5.105E-10 6.374E-10 4.769E-10

349.5625 4.784E-10 6.115E-10 7.572E-10 5.550E-10

349.7813 4.839E-10 6.718E-10 8.084E-10 5.527E-10

350.0000 4.269E-10 6.861E-10 7.837E-10 5.114E-10

**Data plotted in FIG 3(a)**

Frequency Cells in LB, -41.4 min Cells in LB, -5.3 min Cells in PMB, 7.2 min

0.2188 8.807E-06 4.994E-06 1.688E-05

0.4375 5.363E-06 3.011E-06 1.045E-05

0.6563 2.112E-06 1.144E-06 4.278E-06

0.8750 3.623E-07 1.520E-07 7.876E-07

1.0938 -6.210E-08 -6.410E-08 -2.311E-07

1.3125 1.740E-08 1.511E-08 -1.930E-07

1.5313 8.369E-08 8.064E-08 -5.176E-08

1.7500 7.140E-08 8.943E-08 -5.972E-09

1.9688 5.067E-08 8.363E-08 6.079E-09

2.1875 4.183E-08 7.900E-08 1.031E-08

2.4063 3.551E-08 7.311E-08 7.236E-09

2.6250 2.993E-08 6.664E-08 4.461E-09

2.8438 2.667E-08 6.128E-08 4.302E-09

3.0625 2.499E-08 5.710E-08 3.708E-09

3.2813 2.423E-08 5.456E-08 2.307E-09

3.5000 2.373E-08 5.401E-08 9.496E-10

3.7188 2.241E-08 5.423E-08 -2.045E-10

3.9375 2.029E-08 5.325E-08 -6.964E-10

4.1563 1.852E-08 5.013E-08 -3.748E-10

4.3750 1.791E-08 4.555E-08 1.133E-10

4.5938 1.833E-08 4.120E-08 3.388E-10

4.8125 1.894E-08 3.853E-08 4.348E-10

5.0313 1.901E-08 3.776E-08 6.047E-10

5.2500 1.847E-08 3.798E-08 9.032E-10

5.4688 1.777E-08 3.784E-08 1.228E-09

5.6875 1.722E-08 3.667E-08 1.427E-09

5.9063 1.684E-08 3.481E-08 1.481E-09

6.1250 1.664E-08 3.308E-08 1.453E-09

6.3438 1.670E-08 3.189E-08 1.341E-09

6.5625 1.691E-08 3.109E-08 1.134E-09

6.7813 1.686E-08 3.034E-08 8.752E-10

7.0000 1.627E-08 2.949E-08 6.033E-10

7.2188 1.526E-08 2.841E-08 3.171E-10

7.4375 1.414E-08 2.688E-08 9.940E-12

7.6563 1.302E-08 2.473E-08 -3.000E-10

7.8750 1.177E-08 2.216E-08 -5.596E-10

8.0938 1.041E-08 1.976E-08 -7.105E-10

8.3125 9.236E-09 1.824E-08 -7.354E-10

8.5313 8.616E-09 1.781E-08 -6.779E-10

8.7500 8.575E-09 1.805E-08 -6.485E-10

8.9688 8.783E-09 1.824E-08 -7.855E-10

9.1875 8.862E-09 1.795E-08 -1.123E-09

9.4063 8.696E-09 1.738E-08 -1.502E-09

9.6250 8.435E-09 1.694E-08 -1.683E-09

9.8438 8.250E-09 1.689E-08 -1.535E-09

10.0625 8.118E-09 1.723E-08 -1.106E-09

10.2813 7.873E-09 1.778E-08 -5.539E-10

10.5000 7.420E-09 1.830E-08 -5.959E-11

10.7188 6.819E-09 1.850E-08 2.669E-10

10.9375 6.185E-09 1.829E-08 4.454E-10

11.1563 5.597E-09 1.788E-08 5.641E-10

11.3750 5.117E-09 1.753E-08 6.406E-10

11.5938 4.827E-09 1.716E-08 5.895E-10

11.8125 4.799E-09 1.633E-08 3.449E-10

12.0313 5.004E-09 1.481E-08 -3.498E-11

12.2500 5.284E-09 1.305E-08 -4.120E-10

12.4688 5.464E-09 1.185E-08 -6.794E-10

12.6875 5.496E-09 1.159E-08 -8.052E-10

12.9063 5.476E-09 1.187E-08 -8.081E-10

13.1250 5.508E-09 1.194E-08 -7.242E-10

13.3438 5.615E-09 1.150E-08 -5.737E-10

13.5625 5.773E-09 1.082E-08 -3.444E-10

13.7813 5.954E-09 1.039E-08 -4.500E-11

14.0000 6.095E-09 1.049E-08 2.147E-10

14.2188 6.126E-09 1.098E-08 2.558E-10

14.4375 6.076E-09 1.152E-08 -6.743E-12

14.6563 6.100E-09 1.179E-08 -4.546E-10

14.8750 6.314E-09 1.168E-08 -8.521E-10

15.0938 6.642E-09 1.129E-08 -1.032E-09

15.3125 6.861E-09 1.089E-08 -9.950E-10

15.5313 6.791E-09 1.075E-08 -8.536E-10

15.7500 6.430E-09 1.097E-08 -7.189E-10

15.9688 5.926E-09 1.141E-08 -6.253E-10

16.1875 5.451E-09 1.172E-08 -5.462E-10

16.4063 5.089E-09 1.162E-08 -4.666E-10

16.6250 4.823E-09 1.108E-08 -4.271E-10

16.8438 4.619E-09 1.036E-08 -4.877E-10

17.0625 4.478E-09 9.772E-09 -6.456E-10

17.2813 4.406E-09 9.578E-09 -8.045E-10

17.5000 4.390E-09 9.798E-09 -8.414E-10

17.7188 4.418E-09 1.016E-08 -7.125E-10

17.9375 4.482E-09 1.021E-08 -5.001E-10

18.1563 4.549E-09 9.653E-09 -3.674E-10

18.3750 4.588E-09 8.666E-09 -4.557E-10

18.5938 4.619E-09 7.798E-09 -7.789E-10

18.8125 4.662E-09 7.477E-09 -1.181E-09

19.0313 4.635E-09 7.645E-09 -1.418E-09

19.2500 4.395E-09 7.911E-09 -1.348E-09

19.4688 3.926E-09 7.969E-09 -1.055E-09

19.6875 3.413E-09 7.805E-09 -7.753E-10

19.9063 3.089E-09 7.549E-09 -6.644E-10

20.1250 3.056E-09 7.290E-09 -6.785E-10

20.3438 3.277E-09 7.081E-09 -6.742E-10

20.5625 3.640E-09 6.993E-09 -5.808E-10

20.7813 4.007E-09 7.064E-09 -4.382E-10

21.0000 4.234E-09 7.249E-09 -3.124E-10

21.2188 4.228E-09 7.464E-09 -2.391E-10

21.4375 4.017E-09 7.679E-09 -2.362E-10

21.6563 3.726E-09 7.903E-09 -3.105E-10

21.8750 3.483E-09 8.124E-09 -4.335E-10

22.0938 3.335E-09 8.300E-09 -5.432E-10

22.3125 3.250E-09 8.401E-09 -5.879E-10

22.5313 3.185E-09 8.432E-09 -5.599E-10

22.7500 3.150E-09 8.447E-09 -4.842E-10

22.9688 3.194E-09 8.563E-09 -3.839E-10

23.1875 3.337E-09 8.849E-09 -2.611E-10

23.4063 3.499E-09 9.139E-09 -1.183E-10

23.6250 3.538E-09 9.042E-09 1.834E-12

23.8438 3.395E-09 8.321E-09 1.923E-11

24.0625 3.186E-09 7.278E-09 -1.157E-10

24.2813 3.107E-09 6.621E-09 -3.594E-10

24.5000 3.249E-09 6.835E-09 -6.006E-10

24.7188 3.537E-09 7.723E-09 -7.499E-10

24.9375 3.811E-09 8.614E-09 -7.911E-10

25.1563 3.935E-09 8.973E-09 -7.468E-10

25.3750 3.883E-09 8.758E-09 -6.194E-10

25.5938 3.778E-09 8.261E-09 -3.903E-10

25.8125 3.799E-09 7.736E-09 -8.304E-11

26.0313 3.985E-09 7.237E-09 1.911E-10

26.2500 4.170E-09 6.742E-09 2.841E-10

26.4688 4.166E-09 6.304E-09 1.386E-10

26.6875 3.964E-09 6.026E-09 -1.291E-10

26.9063 3.688E-09 5.918E-09 -2.954E-10

27.1250 3.405E-09 5.863E-09 -2.167E-10

27.3438 3.103E-09 5.754E-09 3.749E-11

27.5625 2.822E-09 5.636E-09 2.495E-10

27.7813 2.695E-09 5.630E-09 2.542E-10

28.0000 2.777E-09 5.744E-09 5.445E-11

28.2188 2.924E-09 5.832E-09 -2.556E-10

28.4375 2.910E-09 5.753E-09 -6.190E-10

28.6563 2.665E-09 5.519E-09 -1.025E-09

28.8750 2.355E-09 5.269E-09 -1.427E-09

29.0938 2.202E-09 5.154E-09 -1.714E-09

29.3125 2.260E-09 5.275E-09 -1.763E-09

29.5313 2.403E-09 5.647E-09 -1.501E-09

29.7500 2.509E-09 6.155E-09 -9.603E-10

29.9688 2.587E-09 6.546E-09 -3.065E-10

30.1875 2.687E-09 6.573E-09 2.076E-10

30.4063 2.760E-09 6.191E-09 3.879E-10

30.6250 2.672E-09 5.615E-09 2.372E-10

30.8438 2.387E-09 5.168E-09 -6.032E-11

31.0625 2.049E-09 5.034E-09 -2.955E-10

31.2813 1.864E-09 5.145E-09 -3.786E-10

31.5000 1.910E-09 5.298E-09 -3.562E-10

31.7188 2.113E-09 5.364E-09 -3.216E-10

31.9375 2.363E-09 5.380E-09 -3.224E-10

32.1563 2.585E-09 5.423E-09 -3.478E-10

32.3750 2.730E-09 5.455E-09 -3.844E-10

32.5938 2.770E-09 5.331E-09 -4.599E-10

32.8125 2.734E-09 5.016E-09 -6.097E-10

33.0313 2.703E-09 4.695E-09 -8.114E-10

33.2500 2.743E-09 4.609E-09 -9.811E-10

33.4688 2.866E-09 4.795E-09 -1.045E-09

33.6875 3.014E-09 5.059E-09 -9.962E-10

33.9063 3.074E-09 5.201E-09 -8.729E-10

34.1250 2.934E-09 5.190E-09 -7.046E-10

34.3438 2.594E-09 5.106E-09 -5.153E-10

34.5625 2.214E-09 5.002E-09 -3.743E-10

34.7813 2.002E-09 4.876E-09 -3.913E-10

35.0000 2.037E-09 4.746E-09 -6.110E-10

35.2188 2.209E-09 4.681E-09 -9.251E-10

35.4375 2.344E-09 4.757E-09 -1.142E-09

35.6563 2.374E-09 4.995E-09 -1.161E-09

35.8750 2.385E-09 5.325E-09 -1.049E-09

36.0938 2.491E-09 5.594E-09 -9.311E-10

36.3125 2.666E-09 5.646E-09 -8.493E-10

36.5313 2.740E-09 5.437E-09 -7.675E-10

36.7500 2.585E-09 5.103E-09 -6.634E-10

36.9688 2.283E-09 4.865E-09 -5.700E-10

37.1875 2.051E-09 4.848E-09 -5.324E-10

37.4063 2.020E-09 4.983E-09 -5.603E-10

37.6250 2.126E-09 5.093E-09 -6.217E-10

37.8438 2.210E-09 5.046E-09 -6.652E-10

38.0625 2.185E-09 4.827E-09 -6.513E-10

38.2813 2.105E-09 4.484E-09 -5.788E-10

38.5000 2.086E-09 4.089E-09 -4.826E-10

38.7188 2.183E-09 3.723E-09 -4.051E-10

38.9375 2.351E-09 3.470E-09 -3.721E-10

39.1563 2.513E-09 3.393E-09 -3.939E-10

39.3750 2.639E-09 3.511E-09 -4.715E-10

39.5938 2.741E-09 3.807E-09 -5.797E-10

39.8125 2.806E-09 4.241E-09 -6.391E-10

40.0313 2.766E-09 4.726E-09 -5.298E-10

40.2500 2.567E-09 5.118E-09 -1.732E-10

40.4688 2.264E-09 5.267E-09 3.838E-10

40.6875 2.027E-09 5.162E-09 9.780E-10

40.9063 2.022E-09 4.973E-09 1.425E-09

41.1250 2.257E-09 4.882E-09 1.599E-09

41.3438 2.547E-09 4.873E-09 1.456E-09

41.5625 2.655E-09 4.772E-09 1.055E-09

41.7813 2.499E-09 4.489E-09 5.520E-10

42.0000 2.214E-09 4.129E-09 1.192E-10

42.2188 1.997E-09 3.845E-09 -1.832E-10

42.4375 1.908E-09 3.691E-09 -4.169E-10

42.6563 1.857E-09 3.656E-09 -6.398E-10

42.8750 1.768E-09 3.749E-09 -8.160E-10

43.0938 1.678E-09 3.948E-09 -8.776E-10

43.3125 1.667E-09 4.126E-09 -8.341E-10

43.5313 1.731E-09 4.119E-09 -7.678E-10

43.7500 1.786E-09 3.894E-09 -7.298E-10

43.9688 1.767E-09 3.585E-09 -6.860E-10

44.1875 1.680E-09 3.350E-09 -5.781E-10

44.4063 1.551E-09 3.227E-09 -4.147E-10

44.6250 1.394E-09 3.162E-09 -2.775E-10

44.8438 1.235E-09 3.145E-09 -2.354E-10

45.0625 1.140E-09 3.259E-09 -2.657E-10

45.2813 1.179E-09 3.579E-09 -2.675E-10

45.5000 1.357E-09 4.058E-09 -1.563E-10

45.7188 1.600E-09 4.510E-09 6.200E-11

45.9375 1.813E-09 4.724E-09 3.071E-10

46.1563 1.933E-09 4.608E-09 4.982E-10

46.3750 1.955E-09 4.260E-09 5.988E-10

46.5938 1.921E-09 3.892E-09 6.045E-10

46.8125 1.901E-09 3.647E-09 5.182E-10

47.0313 1.943E-09 3.502E-09 3.426E-10

47.2500 2.045E-09 3.391E-09 8.240E-11

47.4688 2.161E-09 3.367E-09 -2.444E-10

47.6875 2.249E-09 3.554E-09 -5.818E-10

47.9063 2.290E-09 3.925E-09 -8.365E-10

48.1250 2.273E-09 4.231E-09 -9.268E-10

48.3438 2.202E-09 4.221E-09 -8.294E-10

48.5625 2.127E-09 3.897E-09 -5.779E-10

48.7813 2.145E-09 3.526E-09 -2.486E-10

49.0000 2.305E-09 3.458E-09 4.142E-11

49.2188 2.523E-09 3.910E-09 1.783E-10

49.4375 2.622E-09 4.823E-09 1.357E-10

49.6563 2.491E-09 5.835E-09 -1.555E-11

49.8750 2.196E-09 6.453E-09 -2.084E-10

50.0938 1.912E-09 6.367E-09 -4.476E-10

50.3125 1.749E-09 5.664E-09 -7.529E-10

50.5313 1.671E-09 4.730E-09 -1.074E-09

50.7500 1.590E-09 3.944E-09 -1.292E-09

50.9688 1.486E-09 3.475E-09 -1.282E-09

51.1875 1.410E-09 3.287E-09 -9.972E-10

51.4063 1.408E-09 3.247E-09 -5.566E-10

51.6250 1.479E-09 3.212E-09 -2.169E-10

51.8438 1.602E-09 3.103E-09 -1.679E-10

52.0625 1.777E-09 2.958E-09 -3.193E-10

52.2813 2.011E-09 2.908E-09 -3.736E-10

52.5000 2.270E-09 3.065E-09 -1.475E-10

52.7188 2.470E-09 3.423E-09 2.283E-10

52.9375 2.530E-09 3.853E-09 4.511E-10

53.1563 2.424E-09 4.171E-09 3.364E-10

53.3750 2.192E-09 4.255E-09 -5.099E-11

53.5938 1.918E-09 4.129E-09 -4.938E-10

53.8125 1.706E-09 3.922E-09 -8.006E-10

54.0313 1.641E-09 3.744E-09 -9.048E-10

54.2500 1.725E-09 3.615E-09 -8.392E-10

54.4688 1.855E-09 3.524E-09 -6.626E-10

54.6875 1.896E-09 3.474E-09 -4.448E-10

54.9063 1.801E-09 3.450E-09 -2.958E-10

55.1250 1.639E-09 3.390E-09 -3.237E-10

55.3438 1.492E-09 3.261E-09 -5.186E-10

55.5625 1.369E-09 3.142E-09 -7.298E-10

55.7813 1.250E-09 3.169E-09 -8.081E-10

56.0000 1.182E-09 3.386E-09 -7.456E-10

56.2188 1.281E-09 3.695E-09 -6.362E-10

56.4375 1.600E-09 3.935E-09 -5.186E-10

56.6563 2.029E-09 3.993E-09 -3.385E-10

56.8750 2.344E-09 3.850E-09 -9.110E-11

57.0938 2.370E-09 3.568E-09 1.221E-10

57.3125 2.149E-09 3.239E-09 2.298E-10

57.5313 1.899E-09 2.924E-09 1.858E-10

57.7500 1.777E-09 2.634E-09 -7.051E-11

57.9688 1.764E-09 2.400E-09 -2.793E-10

58.1875 1.789E-09 2.340E-09 -1.923E-10

58.4063 1.723E-09 2.468E-09 -6.864E-10

58.6250 1.350E-09 2.498E-09 -1.507E-10

58.8438 1.445E-09 2.551E-09 1.621E-08

59.0625 5.592E-09 4.742E-09 7.720E-08

59.2813 1.905E-08 1.315E-08 1.964E-07

59.5000 4.256E-08 2.982E-08 3.336E-07

59.7188 6.706E-08 4.976E-08 4.058E-07

59.9375 7.811E-08 6.210E-08 3.576E-07

60.1563 6.838E-08 5.869E-08 2.195E-07

60.3750 4.466E-08 4.183E-08 7.965E-08

60.5938 2.114E-08 2.208E-08 2.210E-09

60.8125 7.085E-09 8.686E-09 -1.419E-08

61.0313 2.129E-09 3.427E-09 -6.477E-09

61.2500 1.327E-09 2.669E-09 -6.323E-10

61.4688 1.194E-09 2.833E-09 -7.739E-11

61.6875 9.603E-10 2.794E-09 -4.478E-10

61.9063 8.342E-10 2.764E-09 -2.319E-10

62.1250 8.666E-10 2.839E-09 4.189E-12

62.3438 9.825E-10 2.822E-09 9.714E-11

62.5625 1.159E-09 2.614E-09 1.744E-10

62.7813 1.394E-09 2.334E-09 2.242E-10

63.0000 1.650E-09 2.150E-09 2.720E-10

63.2188 1.850E-09 2.106E-09 3.776E-10

63.4375 1.926E-09 2.099E-09 4.772E-10

63.6563 1.881E-09 2.016E-09 4.518E-10

63.8750 1.800E-09 1.868E-09 2.985E-10

64.0938 1.772E-09 1.774E-09 1.486E-10

64.3125 1.832E-09 1.817E-09 1.246E-10

64.5313 1.943E-09 1.972E-09 2.076E-10

64.7500 2.016E-09 2.183E-09 2.662E-10

64.9688 1.967E-09 2.432E-09 1.940E-10

65.1875 1.783E-09 2.689E-09 -1.821E-11

65.4063 1.559E-09 2.864E-09 -3.156E-10

65.6250 1.429E-09 2.865E-09 -6.256E-10

65.8438 1.440E-09 2.726E-09 -8.617E-10

66.0625 1.524E-09 2.602E-09 -9.295E-10

66.2813 1.587E-09 2.619E-09 -7.798E-10

66.5000 1.596E-09 2.769E-09 -4.729E-10

66.7188 1.554E-09 2.937E-09 -1.626E-10

66.9375 1.449E-09 2.994E-09 6.130E-12

67.1563 1.288E-09 2.854E-09 -7.784E-12

67.3750 1.147E-09 2.513E-09 -1.348E-10

67.5938 1.143E-09 2.091E-09 -2.616E-10

67.8125 1.304E-09 1.801E-09 -3.114E-10

68.0313 1.524E-09 1.801E-09 -2.821E-10

68.2500 1.660E-09 2.058E-09 -2.237E-10

68.4688 1.657E-09 2.369E-09 -1.831E-10

68.6875 1.567E-09 2.528E-09 -1.801E-10

68.9063 1.481E-09 2.478E-09 -2.257E-10

69.1250 1.444E-09 2.320E-09 -3.346E-10

69.3438 1.448E-09 2.183E-09 -4.971E-10

69.5625 1.455E-09 2.116E-09 -6.482E-10

69.7813 1.433E-09 2.091E-09 -6.895E-10

70.0000 1.377E-09 2.072E-09 -5.648E-10

70.2188 1.306E-09 2.043E-09 -3.201E-10

70.4375 1.254E-09 1.981E-09 -8.100E-11

70.6563 1.255E-09 1.876E-09 4.543E-11

70.8750 1.326E-09 1.784E-09 5.281E-11

71.0938 1.447E-09 1.811E-09 1.614E-11

71.3125 1.561E-09 1.993E-09 -6.113E-12

71.5313 1.603E-09 2.228E-09 -3.554E-11

71.7500 1.532E-09 2.342E-09 -1.285E-10

71.9688 1.365E-09 2.234E-09 -2.820E-10

72.1875 1.177E-09 1.939E-09 -4.173E-10

72.4063 1.055E-09 1.582E-09 -4.687E-10

72.6250 1.021E-09 1.303E-09 -4.607E-10

72.8438 1.030E-09 1.194E-09 -4.716E-10

73.0625 1.031E-09 1.277E-09 -5.319E-10

73.2813 1.039E-09 1.489E-09 -5.825E-10

73.5000 1.112E-09 1.710E-09 -5.199E-10

73.7188 1.275E-09 1.831E-09 -2.721E-10

73.9375 1.482E-09 1.864E-09 1.359E-10

74.1563 1.650E-09 1.975E-09 5.555E-10

74.3750 1.723E-09 2.366E-09 7.807E-10

74.5938 1.700E-09 3.052E-09 6.925E-10

74.8125 1.624E-09 3.747E-09 3.553E-10

75.0313 1.548E-09 4.040E-09 -4.147E-11

75.2500 1.500E-09 3.736E-09 -3.340E-10

75.4688 1.492E-09 3.045E-09 -4.734E-10

75.6875 1.530E-09 2.404E-09 -4.983E-10

75.9063 1.612E-09 2.115E-09 -4.515E-10

76.1250 1.719E-09 2.167E-09 -3.382E-10

76.3438 1.810E-09 2.346E-09 -1.605E-10

76.5625 1.837E-09 2.463E-09 2.750E-11

76.7813 1.777E-09 2.453E-09 1.324E-10

77.0000 1.655E-09 2.347E-09 1.071E-10

77.2188 1.542E-09 2.216E-09 4.115E-12

77.4375 1.485E-09 2.122E-09 -7.049E-11

77.6563 1.455E-09 2.074E-09 -5.098E-11

77.8750 1.385E-09 2.026E-09 4.316E-11

78.0938 1.254E-09 1.925E-09 1.341E-10

78.3125 1.113E-09 1.767E-09 1.465E-10

78.5313 1.041E-09 1.582E-09 5.706E-11

78.7500 1.083E-09 1.407E-09 -9.787E-11

78.9688 1.224E-09 1.275E-09 -2.542E-10

79.1875 1.397E-09 1.233E-09 -3.615E-10

79.4063 1.507E-09 1.309E-09 -4.040E-10

79.6250 1.488E-09 1.481E-09 -3.995E-10

79.8438 1.348E-09 1.681E-09 -3.840E-10

80.0625 1.168E-09 1.839E-09 -3.899E-10

80.2813 1.039E-09 1.930E-09 -4.321E-10

80.5000 1.005E-09 1.975E-09 -5.129E-10

80.7188 1.058E-09 1.995E-09 -6.251E-10

80.9375 1.147E-09 1.994E-09 -7.450E-10

81.1563 1.199E-09 1.977E-09 -8.406E-10

81.3750 1.172E-09 1.973E-09 -8.986E-10

81.5938 1.111E-09 2.004E-09 -9.290E-10

81.8125 1.136E-09 2.044E-09 -9.283E-10

82.0313 1.311E-09 2.043E-09 -8.503E-10

82.2500 1.560E-09 1.988E-09 -6.406E-10

82.4688 1.718E-09 1.922E-09 -3.108E-10

82.6875 1.695E-09 1.894E-09 2.651E-11

82.9063 1.541E-09 1.925E-09 2.076E-10

83.1250 1.381E-09 2.005E-09 1.253E-10

83.3438 1.300E-09 2.104E-09 -1.882E-10

83.5625 1.295E-09 2.174E-09 -5.803E-10

83.7813 1.316E-09 2.178E-09 -8.885E-10

84.0000 1.324E-09 2.132E-09 -1.043E-09

84.2188 1.324E-09 2.104E-09 -1.070E-09

84.4375 1.349E-09 2.140E-09 -1.026E-09

84.6563 1.408E-09 2.197E-09 -9.275E-10

84.8750 1.467E-09 2.177E-09 -7.632E-10

85.0938 1.464E-09 2.029E-09 -5.374E-10

85.3125 1.365E-09 1.825E-09 -3.053E-10

85.5313 1.203E-09 1.722E-09 -1.572E-10

85.7500 1.053E-09 1.834E-09 -1.521E-10

85.9688 9.735E-10 2.124E-09 -2.569E-10

86.1875 9.789E-10 2.426E-09 -3.612E-10

86.4063 1.038E-09 2.592E-09 -3.747E-10

86.6250 1.096E-09 2.598E-09 -3.107E-10

86.8438 1.107E-09 2.508E-09 -2.651E-10

87.0625 1.076E-09 2.362E-09 -3.229E-10

87.2813 1.046E-09 2.157E-09 -5.051E-10

87.5000 1.046E-09 1.920E-09 -7.805E-10

87.7188 1.056E-09 1.732E-09 -1.073E-09

87.9375 1.055E-09 1.651E-09 -1.248E-09

88.1563 1.057E-09 1.655E-09 -1.163E-09

88.3750 1.083E-09 1.681E-09 -7.916E-10

88.5938 1.132E-09 1.703E-09 -2.989E-10

88.8125 1.170E-09 1.721E-09 6.171E-11

89.0313 1.147E-09 1.713E-09 1.407E-10

89.2500 1.025E-09 1.641E-09 -1.256E-11

89.4688 8.233E-10 1.534E-09 -2.314E-10

89.6875 6.502E-10 1.489E-09 -3.800E-10

89.9063 6.235E-10 1.574E-09 -4.245E-10

90.1250 7.655E-10 1.748E-09 -4.006E-10

90.3438 9.933E-10 1.899E-09 -3.585E-10

90.5625 1.210E-09 1.948E-09 -3.418E-10

90.7813 1.364E-09 1.899E-09 -3.818E-10

91.0000 1.436E-09 1.808E-09 -4.706E-10

91.2188 1.414E-09 1.741E-09 -5.410E-10

91.4375 1.327E-09 1.746E-09 -5.147E-10

91.6563 1.248E-09 1.846E-09 -4.000E-10

91.8750 1.252E-09 2.013E-09 -3.099E-10

92.0938 1.347E-09 2.173E-09 -3.369E-10

92.3125 1.479E-09 2.248E-09 -4.302E-10

92.5313 1.576E-09 2.213E-09 -4.574E-10

92.7500 1.609E-09 2.099E-09 -3.884E-10

92.9688 1.586E-09 1.955E-09 -3.501E-10

93.1875 1.499E-09 1.802E-09 -4.746E-10

93.4063 1.294E-09 1.642E-09 -7.464E-10

93.6250 9.515E-10 1.488E-09 -1.026E-09

93.8438 5.873E-10 1.384E-09 -1.183E-09

94.0625 4.255E-10 1.379E-09 -1.181E-09

94.2813 6.107E-10 1.475E-09 -1.068E-09

94.5000 1.055E-09 1.620E-09 -9.196E-10

94.7188 1.505E-09 1.759E-09 -7.956E-10

94.9375 1.747E-09 1.897E-09 -7.192E-10

95.1563 1.731E-09 2.083E-09 -6.975E-10

95.3750 1.523E-09 2.307E-09 -7.332E-10

95.5938 1.217E-09 2.445E-09 -8.000E-10

95.8125 9.165E-10 2.348E-09 -8.284E-10

96.0313 7.361E-10 1.995E-09 -7.555E-10

96.2500 7.279E-10 1.546E-09 -5.986E-10

96.4688 8.202E-10 1.216E-09 -4.538E-10

96.6875 8.793E-10 1.110E-09 -4.065E-10

96.9063 8.444E-10 1.181E-09 -4.469E-10

97.1250 7.699E-10 1.322E-09 -4.788E-10

97.3438 7.366E-10 1.468E-09 -4.079E-10

97.5625 7.640E-10 1.609E-09 -2.264E-10

97.7813 8.221E-10 1.740E-09 -2.804E-11

98.0000 8.907E-10 1.833E-09 5.588E-11

98.2188 9.698E-10 1.853E-09 -5.135E-11

98.4375 1.041E-09 1.799E-09 -3.218E-10

98.6563 1.060E-09 1.733E-09 -6.462E-10

98.8750 1.006E-09 1.768E-09 -8.999E-10

99.0938 9.196E-10 2.048E-09 -9.970E-10

99.3125 8.601E-10 2.667E-09 -9.109E-10

99.5313 8.390E-10 3.550E-09 -6.787E-10

99.7500 8.350E-10 4.392E-09 -4.008E-10

99.9688 8.645E-10 4.805E-09 -2.068E-10

100.1875 9.836E-10 4.595E-09 -1.718E-10

100.4063 1.199E-09 3.913E-09 -2.514E-10

100.6250 1.425E-09 3.110E-09 -3.349E-10

100.8438 1.557E-09 2.466E-09 -3.752E-10

101.0625 1.569E-09 2.057E-09 -4.267E-10

101.2813 1.500E-09 1.823E-09 -5.326E-10

101.5000 1.390E-09 1.699E-09 -6.295E-10

101.7188 1.267E-09 1.658E-09 -6.172E-10

101.9375 1.156E-09 1.704E-09 -4.993E-10

102.1563 1.062E-09 1.814E-09 -4.001E-10

102.3750 9.495E-10 1.900E-09 -4.301E-10

102.5938 7.871E-10 1.860E-09 -5.686E-10

102.8125 6.370E-10 1.674E-09 -7.041E-10

103.0313 6.635E-10 1.454E-09 -7.571E-10

103.2500 9.910E-10 1.346E-09 -7.096E-10

103.4688 1.487E-09 1.347E-09 -5.964E-10

103.6875 1.789E-09 1.246E-09 -5.688E-10

103.9063 1.877E-09 9.699E-10 -6.672E-10

104.1250 2.744E-09 1.298E-09 1.397E-12

104.3438 5.953E-09 4.028E-09 3.621E-09

104.5625 1.187E-08 1.063E-08 1.202E-08

104.7813 1.855E-08 2.020E-08 2.403E-08

105.0000 2.314E-08 2.921E-08 3.528E-08

105.2188 2.434E-08 3.389E-08 4.137E-08

105.4375 2.286E-08 3.297E-08 4.094E-08

105.6563 1.971E-08 2.783E-08 3.539E-08

105.8750 1.521E-08 2.081E-08 2.686E-08

106.0938 9.853E-09 1.380E-08 1.730E-08

106.3125 5.025E-09 8.102E-09 8.796E-09

106.5313 2.051E-09 4.438E-09 3.014E-09

106.7500 9.787E-10 2.738E-09 2.071E-10

106.9688 8.567E-10 2.298E-09 -7.149E-10

107.1875 8.928E-10 2.354E-09 -9.803E-10

107.4063 9.308E-10 2.464E-09 -1.143E-09

107.6250 1.062E-09 2.484E-09 -1.232E-09

107.8438 1.243E-09 2.403E-09 -1.189E-09

108.0625 1.320E-09 2.251E-09 -1.033E-09

108.2813 1.219E-09 2.058E-09 -8.440E-10

108.5000 9.964E-10 1.866E-09 -7.117E-10

108.7188 7.786E-10 1.759E-09 -6.966E-10

108.9375 6.530E-10 1.821E-09 -7.898E-10

109.1563 6.145E-10 2.018E-09 -9.157E-10

109.3750 5.987E-10 2.182E-09 -9.828E-10

109.5938 5.588E-10 2.177E-09 -9.470E-10

109.8125 5.089E-10 2.053E-09 -8.338E-10

110.0313 5.026E-10 1.984E-09 -6.991E-10

110.2500 5.769E-10 2.070E-09 -5.868E-10

110.4688 7.143E-10 2.227E-09 -5.337E-10

110.6875 8.572E-10 2.294E-09 -5.831E-10

110.9063 9.575E-10 2.199E-09 -7.548E-10

111.1250 1.008E-09 2.007E-09 -1.004E-09

111.3438 1.018E-09 1.824E-09 -1.236E-09

111.5625 9.800E-10 1.697E-09 -1.363E-09

111.7813 8.761E-10 1.622E-09 -1.346E-09

112.0000 7.476E-10 1.599E-09 -1.204E-09

112.2188 7.121E-10 1.645E-09 -1.005E-09

112.4375 8.764E-10 1.744E-09 -8.280E-10

112.6563 1.228E-09 1.832E-09 -6.883E-10

112.8750 1.632E-09 1.855E-09 -5.336E-10

113.0938 1.932E-09 1.853E-09 -3.236E-10

113.3125 2.036E-09 1.928E-09 -1.074E-10

113.5313 1.923E-09 2.132E-09 2.169E-11

113.7500 1.637E-09 2.392E-09 3.013E-11

113.9688 1.281E-09 2.580E-09 -3.561E-11

114.1875 9.866E-10 2.622E-09 -1.195E-10

114.4063 8.331E-10 2.534E-09 -1.957E-10

114.6250 8.076E-10 2.352E-09 -2.400E-10

114.8438 8.459E-10 2.094E-09 -2.143E-10

115.0625 8.904E-10 1.787E-09 -1.085E-10

115.2813 9.031E-10 1.511E-09 2.298E-11

115.5000 8.590E-10 1.354E-09 9.260E-11

115.7188 7.617E-10 1.339E-09 5.349E-11

115.9375 6.590E-10 1.417E-09 -6.924E-11

116.1563 6.156E-10 1.525E-09 -2.239E-10

116.3750 6.584E-10 1.629E-09 -3.790E-10

116.5938 7.573E-10 1.705E-09 -5.129E-10

116.8125 8.648E-10 1.724E-09 -5.907E-10

117.0313 9.650E-10 1.678E-09 -5.777E-10

117.2500 1.077E-09 1.588E-09 -4.969E-10

117.4688 1.207E-09 1.482E-09 -4.780E-10

117.6875 1.313E-09 1.370E-09 -5.868E-10

117.9063 1.347E-09 1.279E-09 -5.911E-10

118.1250 1.333E-09 1.292E-09 -4.191E-10

118.3438 1.328E-09 1.441E-09 -5.237E-10

118.5625 1.309E-09 1.549E-09 8.638E-10

118.7813 1.436E-09 1.368E-09 1.208E-08

119.0000 2.671E-09 1.178E-09 4.370E-08

119.2188 6.524E-09 2.112E-09 9.158E-08

119.4375 1.315E-08 5.253E-09 1.266E-07

119.6563 1.970E-08 1.006E-08 1.147E-07

119.8750 2.187E-08 1.410E-08 5.392E-08

120.0938 1.792E-08 1.495E-08 -1.739E-08

120.3125 1.056E-08 1.238E-08 -5.561E-08

120.5313 4.278E-09 8.325E-09 -5.096E-08

120.7500 1.385E-09 4.925E-09 -2.659E-08

120.9688 1.077E-09 3.006E-09 -7.592E-09

121.1875 1.544E-09 2.215E-09 -7.587E-10

121.4063 1.818E-09 1.955E-09 -2.175E-10

121.6250 1.815E-09 1.874E-09 -5.171E-10

121.8438 1.661E-09 1.828E-09 -5.235E-10

122.0625 1.438E-09 1.796E-09 -5.827E-10

122.2813 1.246E-09 1.820E-09 -7.017E-10

122.5000 1.160E-09 1.900E-09 -8.053E-10

122.7188 1.158E-09 1.940E-09 -9.313E-10

122.9375 1.150E-09 1.862E-09 -1.039E-09

123.1563 1.071E-09 1.694E-09 -1.023E-09

123.3750 9.365E-10 1.534E-09 -8.644E-10

123.5938 8.195E-10 1.445E-09 -6.568E-10

123.8125 7.654E-10 1.453E-09 -5.069E-10

124.0313 7.459E-10 1.613E-09 -4.499E-10

124.2500 7.116E-10 2.000E-09 -4.598E-10

124.4688 6.732E-10 2.606E-09 -5.132E-10

124.6875 6.918E-10 3.232E-09 -6.036E-10

124.9063 7.886E-10 3.568E-09 -7.077E-10

125.1250 8.995E-10 3.421E-09 -7.862E-10

125.3438 9.396E-10 2.900E-09 -8.247E-10

125.5625 8.988E-10 2.330E-09 -8.437E-10

125.7813 8.532E-10 1.990E-09 -8.561E-10

126.0000 8.846E-10 1.915E-09 -8.339E-10

126.2188 1.001E-09 1.949E-09 -7.366E-10

126.4375 1.137E-09 1.929E-09 -5.652E-10

126.6563 1.223E-09 1.802E-09 -3.667E-10

126.8750 1.246E-09 1.620E-09 -1.909E-10

127.0938 1.239E-09 1.454E-09 -7.053E-11

127.3125 1.217E-09 1.338E-09 -3.547E-11

127.5313 1.148E-09 1.283E-09 -1.008E-10

127.7500 1.000E-09 1.301E-09 -2.268E-10

127.9688 8.163E-10 1.397E-09 -3.335E-10

128.1875 6.884E-10 1.546E-09 -3.796E-10

128.4063 6.649E-10 1.686E-09 -4.057E-10

128.6250 7.101E-10 1.755E-09 -4.772E-10

128.8438 7.621E-10 1.715E-09 -6.045E-10

129.0625 7.941E-10 1.554E-09 -7.360E-10

129.2813 8.069E-10 1.310E-09 -8.128E-10

129.5000 7.917E-10 1.103E-09 -8.119E-10

129.7188 7.360E-10 1.081E-09 -7.466E-10

129.9375 6.598E-10 1.275E-09 -6.475E-10

130.1563 6.198E-10 1.537E-09 -5.421E-10

130.3750 6.611E-10 1.670E-09 -4.409E-10

130.5938 7.719E-10 1.632E-09 -3.410E-10

130.8125 8.950E-10 1.556E-09 -2.532E-10

131.0313 9.745E-10 1.572E-09 -2.210E-10

131.2500 9.811E-10 1.650E-09 -2.941E-10

131.4688 9.129E-10 1.654E-09 -4.784E-10

131.6875 8.057E-10 1.516E-09 -7.199E-10

131.9063 7.388E-10 1.322E-09 -9.349E-10

132.1250 7.858E-10 1.218E-09 -1.045E-09

132.3438 9.410E-10 1.253E-09 -1.007E-09

132.5625 1.114E-09 1.350E-09 -8.425E-10

132.7813 1.212E-09 1.402E-09 -6.480E-10

133.0000 1.215E-09 1.365E-09 -5.143E-10

133.2188 1.171E-09 1.279E-09 -4.433E-10

133.4375 1.135E-09 1.216E-09 -3.814E-10

133.6563 1.129E-09 1.230E-09 -3.354E-10

133.8750 1.157E-09 1.311E-09 -3.829E-10

134.0938 1.212E-09 1.401E-09 -5.364E-10

134.3125 1.269E-09 1.451E-09 -6.652E-10

134.5313 1.281E-09 1.456E-09 -6.194E-10

134.7500 1.225E-09 1.442E-09 -4.089E-10

134.9688 1.130E-09 1.425E-09 -2.038E-10

135.1875 1.051E-09 1.422E-09 -1.605E-10

135.4063 1.008E-09 1.466E-09 -2.794E-10

135.6250 9.871E-10 1.579E-09 -4.285E-10

135.8438 9.906E-10 1.739E-09 -4.767E-10

136.0625 1.037E-09 1.892E-09 -3.956E-10

136.2813 1.105E-09 2.003E-09 -2.541E-10

136.5000 1.123E-09 2.072E-09 -1.454E-10

136.7188 1.046E-09 2.106E-09 -1.319E-10

136.9375 9.245E-10 2.081E-09 -2.356E-10

137.1563 8.539E-10 1.971E-09 -4.272E-10

137.3750 8.732E-10 1.801E-09 -6.057E-10

137.5938 9.353E-10 1.666E-09 -6.342E-10

137.8125 9.693E-10 1.643E-09 -4.499E-10

138.0313 9.508E-10 1.695E-09 -1.358E-10

138.2500 9.122E-10 1.706E-09 1.401E-10

138.4688 9.004E-10 1.612E-09 2.562E-10

138.6875 9.342E-10 1.469E-09 1.958E-10

138.9063 9.951E-10 1.392E-09 3.616E-12

139.1250 1.046E-09 1.435E-09 -2.700E-10

139.3438 1.055E-09 1.554E-09 -5.654E-10

139.5625 1.006E-09 1.647E-09 -7.950E-10

139.7813 9.081E-10 1.640E-09 -8.771E-10

140.0000 7.998E-10 1.532E-09 -8.051E-10

140.2188 7.249E-10 1.384E-09 -6.705E-10

140.4375 7.015E-10 1.253E-09 -5.984E-10

140.6563 7.110E-10 1.147E-09 -6.499E-10

140.8750 7.244E-10 1.061E-09 -7.846E-10

141.0938 7.352E-10 1.026E-09 -9.177E-10

141.3125 7.483E-10 1.085E-09 -1.007E-09

141.5313 7.423E-10 1.206E-09 -1.080E-09

141.7500 6.798E-10 1.280E-09 -1.170E-09

141.9688 5.791E-10 1.224E-09 -1.249E-09

142.1875 5.451E-10 1.067E-09 -1.247E-09

142.4063 6.800E-10 9.323E-10 -1.144E-09

142.6250 9.649E-10 9.252E-10 -1.015E-09

142.8438 1.256E-09 1.056E-09 -9.482E-10

143.0625 1.403E-09 1.237E-09 -9.387E-10

143.2813 1.357E-09 1.347E-09 -9.006E-10

143.5000 1.190E-09 1.321E-09 -7.698E-10

143.7188 1.029E-09 1.189E-09 -5.588E-10

143.9375 9.676E-10 1.040E-09 -3.162E-10

144.1563 1.003E-09 9.436E-10 -8.200E-11

144.3750 1.055E-09 9.087E-10 1.162E-10

144.5938 1.040E-09 8.983E-10 2.652E-10

144.8125 9.369E-10 8.642E-10 3.712E-10

145.0313 7.854E-10 7.774E-10 4.291E-10

145.2500 6.521E-10 6.560E-10 3.914E-10

145.4688 5.956E-10 5.722E-10 2.095E-10

145.6875 6.379E-10 6.105E-10 -9.379E-11

145.9063 7.518E-10 7.999E-10 -4.275E-10

146.1250 8.688E-10 1.087E-09 -7.127E-10

146.3438 9.040E-10 1.375E-09 -9.382E-10

146.5625 7.980E-10 1.579E-09 -1.135E-09

146.7813 5.706E-10 1.658E-09 -1.314E-09

147.0000 3.335E-10 1.619E-09 -1.436E-09

147.2188 2.222E-10 1.514E-09 -1.438E-09

147.4375 2.928E-10 1.414E-09 -1.296E-09

147.6563 4.839E-10 1.345E-09 -1.084E-09

147.8750 6.741E-10 1.280E-09 -9.462E-10

148.0938 7.656E-10 1.189E-09 -9.879E-10

148.3125 7.301E-10 1.121E-09 -1.167E-09

148.5313 6.070E-10 1.172E-09 -1.314E-09

148.7500 4.710E-10 1.383E-09 -1.280E-09

148.9688 3.885E-10 1.680E-09 -1.062E-09

149.1875 3.886E-10 1.942E-09 -7.916E-10

149.4063 4.594E-10 2.111E-09 -6.038E-10

149.6250 5.631E-10 2.202E-09 -5.372E-10

149.8438 6.547E-10 2.218E-09 -5.372E-10

150.0625 7.072E-10 2.109E-09 -5.264E-10

150.2813 7.379E-10 1.847E-09 -4.578E-10

150.5000 8.012E-10 1.513E-09 -3.287E-10

150.7188 9.327E-10 1.266E-09 -1.840E-10

150.9375 1.095E-09 1.206E-09 -1.115E-10

151.1563 1.200E-09 1.294E-09 -1.977E-10

151.3750 1.198E-09 1.389E-09 -4.432E-10

151.5938 1.125E-09 1.361E-09 -7.238E-10

151.8125 1.054E-09 1.181E-09 -8.767E-10

152.0313 1.018E-09 9.446E-10 -8.488E-10

152.2500 1.002E-09 7.994E-10 -7.416E-10

152.4688 9.884E-10 8.239E-10 -6.971E-10

152.6875 9.816E-10 9.690E-10 -7.625E-10

152.9063 9.757E-10 1.122E-09 -8.793E-10

153.1250 9.370E-10 1.210E-09 -9.679E-10

153.3438 8.414E-10 1.232E-09 -9.903E-10

153.5625 7.215E-10 1.203E-09 -9.522E-10

153.7813 6.496E-10 1.118E-09 -8.848E-10

154.0000 6.650E-10 9.716E-10 -8.247E-10

154.2188 7.359E-10 8.050E-10 -7.901E-10

154.4375 8.024E-10 6.872E-10 -7.754E-10

154.6563 8.418E-10 6.576E-10 -7.736E-10

154.8750 8.737E-10 6.989E-10 -7.925E-10

155.0938 9.123E-10 7.779E-10 -8.400E-10

155.3125 9.476E-10 8.897E-10 -9.107E-10

155.5313 9.773E-10 1.036E-09 -9.962E-10

155.7500 1.028E-09 1.179E-09 -1.089E-09

155.9688 1.115E-09 1.263E-09 -1.162E-09

156.1875 1.199E-09 1.298E-09 -1.174E-09

156.4063 1.211E-09 1.371E-09 -1.098E-09

156.6250 1.126E-09 1.540E-09 -9.541E-10

156.8438 9.995E-10 1.724E-09 -7.848E-10

157.0625 9.243E-10 1.750E-09 -6.266E-10

157.2813 9.557E-10 1.521E-09 -5.110E-10

157.5000 1.078E-09 1.140E-09 -4.416E-10

157.7188 1.244E-09 8.815E-10 -3.289E-10

157.9375 1.429E-09 1.017E-09 -9.473E-12

158.1563 1.627E-09 1.603E-09 5.648E-10

158.3750 1.797E-09 2.381E-09 1.159E-09

158.5938 1.856E-09 2.908E-09 1.392E-09

158.8125 1.765E-09 2.868E-09 1.093E-09

159.0313 1.589E-09 2.305E-09 4.657E-10

159.2500 1.438E-09 1.568E-09 -1.222E-10

159.4688 1.337E-09 1.034E-09 -4.620E-10

159.6875 1.211E-09 8.691E-10 -5.848E-10

159.9063 9.937E-10 1.015E-09 -6.258E-10

160.1250 7.203E-10 1.311E-09 -6.868E-10

160.3438 5.027E-10 1.593E-09 -7.955E-10

160.5625 4.282E-10 1.742E-09 -9.209E-10

160.7813 4.949E-10 1.708E-09 -1.007E-09

161.0000 6.296E-10 1.512E-09 -1.014E-09

161.2188 7.529E-10 1.221E-09 -9.542E-10

161.4375 8.265E-10 9.040E-10 -8.834E-10

161.6563 8.508E-10 6.238E-10 -8.639E-10

161.8750 8.352E-10 4.392E-10 -9.094E-10

162.0938 7.893E-10 3.833E-10 -9.625E-10

162.3125 7.327E-10 4.369E-10 -9.439E-10

162.5313 6.881E-10 5.359E-10 -8.381E-10

162.7500 6.536E-10 6.165E-10 -7.132E-10

162.9688 6.058E-10 6.721E-10 -6.345E-10

163.1875 5.497E-10 7.737E-10 -5.752E-10

163.4063 5.442E-10 1.007E-09 -4.459E-10

163.6250 6.441E-10 1.358E-09 -2.249E-10

163.8438 8.188E-10 1.675E-09 -2.903E-11

164.0625 9.615E-10 1.789E-09 -2.203E-11

164.2813 9.899E-10 1.669E-09 -2.420E-10

164.5000 9.115E-10 1.438E-09 -5.458E-10

164.7188 7.820E-10 1.247E-09 -7.461E-10

164.9375 6.399E-10 1.151E-09 -7.902E-10

165.1563 5.106E-10 1.097E-09 -7.767E-10

165.3750 4.378E-10 1.017E-09 -8.102E-10

165.5938 4.613E-10 9.045E-10 -8.830E-10

165.8125 5.688E-10 8.264E-10 -9.065E-10

166.0313 7.019E-10 8.509E-10 -8.240E-10

166.2500 8.037E-10 9.699E-10 -6.716E-10

166.4688 8.361E-10 1.092E-09 -5.439E-10

166.6875 7.722E-10 1.109E-09 -5.162E-10

166.9063 6.167E-10 9.767E-10 -5.941E-10

167.1250 4.334E-10 7.431E-10 -7.271E-10

167.3438 3.121E-10 5.224E-10 -8.565E-10

167.5625 2.898E-10 4.264E-10 -9.455E-10

167.7813 3.196E-10 5.026E-10 -9.787E-10

168.0000 3.273E-10 7.191E-10 -9.516E-10

168.2188 2.940E-10 9.988E-10 -8.620E-10

168.4375 2.746E-10 1.258E-09 -7.164E-10

168.6563 3.396E-10 1.419E-09 -5.590E-10

168.8750 5.047E-10 1.412E-09 -4.760E-10

169.0938 7.165E-10 1.221E-09 -5.228E-10

169.3125 8.958E-10 9.239E-10 -6.377E-10

169.5313 9.792E-10 6.664E-10 -6.803E-10

169.7500 9.364E-10 5.473E-10 -5.857E-10

169.9688 7.826E-10 5.411E-10 -4.434E-10

170.1875 5.925E-10 5.528E-10 -3.815E-10

170.4063 4.753E-10 5.435E-10 -4.005E-10

170.6250 4.999E-10 5.672E-10 -3.716E-10

170.8438 6.316E-10 6.840E-10 -1.991E-10

171.0625 7.602E-10 8.712E-10 5.324E-11

171.2813 8.015E-10 1.040E-09 2.129E-10

171.5000 7.591E-10 1.115E-09 1.571E-10

171.7188 6.855E-10 1.091E-09 -8.849E-11

171.9375 6.117E-10 1.018E-09 -3.956E-10

172.1563 5.410E-10 9.595E-10 -6.574E-10

172.3750 4.812E-10 9.478E-10 -8.595E-10

172.5938 4.466E-10 9.753E-10 -1.040E-09

172.8125 4.355E-10 1.014E-09 -1.202E-09

173.0313 4.408E-10 1.049E-09 -1.288E-09

173.2500 4.746E-10 1.079E-09 -1.245E-09

173.4688 5.488E-10 1.089E-09 -1.082E-09

173.6875 6.339E-10 1.057E-09 -8.734E-10

173.9063 6.752E-10 9.965E-10 -7.040E-10

174.1250 6.595E-10 1.007E-09 -6.067E-10

174.3438 6.412E-10 1.220E-09 -5.319E-10

174.5625 6.878E-10 1.657E-09 -4.258E-10

174.7813 8.011E-10 2.153E-09 -3.343E-10

175.0000 8.998E-10 2.440E-09 -3.594E-10

175.2188 8.871E-10 2.363E-09 -5.248E-10

175.4375 7.396E-10 2.001E-09 -7.253E-10

175.6563 5.345E-10 1.594E-09 -8.032E-10

175.8750 3.959E-10 1.351E-09 -7.279E-10

176.0938 4.017E-10 1.318E-09 -6.584E-10

176.3125 5.158E-10 1.401E-09 -6.851E-10

176.5313 6.252E-10 1.471E-09 -7.305E-10

176.7500 6.484E-10 1.451E-09 -7.995E-10

176.9688 5.884E-10 1.315E-09 -7.832E-10

177.1875 5.292E-10 1.059E-09 -4.863E-10

177.4063 5.700E-10 7.749E-10 -5.042E-10

177.6250 6.110E-10 6.781E-10 -7.087E-10

177.8438 4.364E-10 8.146E-10 1.091E-09

178.0625 3.407E-10 9.039E-10 1.585E-09

178.2813 6.776E-10 8.170E-10 -2.035E-10

178.5000 4.531E-10 7.686E-10 6.580E-08

178.7188 1.378E-09 1.191E-09 3.998E-07

178.9375 1.872E-08 5.691E-09 1.178E-06

179.1563 7.953E-08 2.665E-08 2.196E-06

179.3750 1.901E-07 8.138E-08 2.761E-06

179.5938 3.022E-07 1.707E-07 2.225E-06

179.8125 3.350E-07 2.598E-07 7.183E-07

180.0313 2.526E-07 2.953E-07 -8.318E-07

180.2500 1.074E-07 2.516E-07 -1.536E-06

180.4688 -7.991E-09 1.571E-07 -1.294E-06

180.6875 -4.692E-08 6.735E-08 -6.698E-07

180.9063 -3.229E-08 1.654E-08 -2.039E-07

181.1250 -9.424E-09 1.102E-09 -2.608E-08

181.3438 4.784E-10 5.832E-10 -4.573E-10

181.5625 1.343E-09 1.508E-09 -2.910E-09

181.7813 8.887E-10 1.276E-09 -1.680E-09

182.0000 9.895E-10 8.648E-10 -8.363E-10

182.2188 1.002E-09 6.285E-10 -1.013E-09

182.4375 8.588E-10 5.257E-10 -8.532E-10

182.6563 7.198E-10 5.562E-10 -7.558E-10

182.8750 5.623E-10 6.021E-10 -8.587E-10

183.0938 4.305E-10 5.596E-10 -8.784E-10

183.3125 4.126E-10 4.981E-10 -8.316E-10

183.5313 4.887E-10 5.128E-10 -7.405E-10

183.7500 5.744E-10 5.911E-10 -5.592E-10

183.9688 6.073E-10 6.692E-10 -3.743E-10

184.1875 5.694E-10 7.092E-10 -3.019E-10

184.4063 4.796E-10 7.065E-10 -3.733E-10

184.6250 3.705E-10 6.752E-10 -5.513E-10

184.8438 2.757E-10 6.402E-10 -7.387E-10

185.0625 2.363E-10 6.262E-10 -8.349E-10

185.2813 2.929E-10 6.471E-10 -8.149E-10

185.5000 4.512E-10 7.071E-10 -7.246E-10

185.7188 6.558E-10 8.032E-10 -6.387E-10

185.9375 8.056E-10 9.127E-10 -6.237E-10

186.1563 8.199E-10 9.838E-10 -6.888E-10

186.3750 6.974E-10 9.694E-10 -7.748E-10

186.5938 5.056E-10 8.733E-10 -8.174E-10

186.8125 3.206E-10 7.498E-10 -8.136E-10

187.0313 1.957E-10 6.590E-10 -8.153E-10

187.2500 1.638E-10 6.296E-10 -8.621E-10

187.4688 2.289E-10 6.566E-10 -9.404E-10

187.6875 3.484E-10 7.146E-10 -1.019E-09

187.9063 4.543E-10 7.691E-10 -1.100E-09

188.1250 5.103E-10 7.889E-10 -1.202E-09

188.3438 5.367E-10 7.586E-10 -1.302E-09

188.5625 5.658E-10 6.866E-10 -1.329E-09

188.7813 5.891E-10 6.059E-10 -1.237E-09

189.0000 5.701E-10 5.590E-10 -1.060E-09

189.2188 5.099E-10 5.761E-10 -8.938E-10

189.4375 4.770E-10 6.579E-10 -8.108E-10

189.6563 5.477E-10 7.706E-10 -7.952E-10

189.8750 7.114E-10 8.581E-10 -7.794E-10

190.0938 8.618E-10 8.801E-10 -7.376E-10

190.3125 8.996E-10 8.528E-10 -7.112E-10

190.5313 8.290E-10 8.362E-10 -7.292E-10

190.7500 7.293E-10 8.600E-10 -7.386E-10

190.9688 6.528E-10 8.787E-10 -6.558E-10

191.1875 5.920E-10 8.255E-10 -4.794E-10

191.4063 5.388E-10 7.004E-10 -3.134E-10

191.6250 5.224E-10 5.766E-10 -2.671E-10

191.8438 5.634E-10 5.265E-10 -3.543E-10

192.0625 6.237E-10 5.652E-10 -4.988E-10

192.2813 6.413E-10 6.601E-10 -6.219E-10

192.5000 6.045E-10 7.672E-10 -7.012E-10

192.7188 5.565E-10 8.528E-10 -7.444E-10

192.9375 5.257E-10 9.029E-10 -7.288E-10

193.1563 4.886E-10 9.257E-10 -5.973E-10

193.3750 4.199E-10 9.403E-10 -3.338E-10

193.5938 3.543E-10 9.516E-10 -2.846E-11

193.8125 3.623E-10 9.362E-10 1.656E-10

194.0313 4.703E-10 8.574E-10 1.635E-10

194.2500 6.262E-10 7.041E-10 2.673E-11

194.4688 7.429E-10 5.184E-10 -1.027E-10

194.6875 7.561E-10 3.840E-10 -1.390E-10

194.9063 6.533E-10 3.752E-10 -1.070E-10

195.1250 4.869E-10 5.009E-10 -6.998E-11

195.3438 3.648E-10 6.891E-10 -4.093E-11

195.5625 3.845E-10 8.370E-10 6.205E-12

195.7813 5.462E-10 8.918E-10 5.762E-11

196.0000 7.451E-10 8.859E-10 4.441E-11

196.2188 8.660E-10 8.949E-10 -7.139E-11

196.4375 8.704E-10 9.706E-10 -2.307E-10

196.6563 7.893E-10 1.107E-09 -3.363E-10

196.8750 6.679E-10 1.244E-09 -3.660E-10

197.0938 5.393E-10 1.300E-09 -3.872E-10

197.3125 4.241E-10 1.228E-09 -4.598E-10

197.5313 3.284E-10 1.072E-09 -5.620E-10

197.7500 2.495E-10 9.249E-10 -6.307E-10

197.9688 1.910E-10 8.474E-10 -6.419E-10

198.1875 1.586E-10 8.141E-10 -6.220E-10

198.4063 1.419E-10 7.643E-10 -5.972E-10

198.6250 1.216E-10 6.779E-10 -5.743E-10

198.8438 1.055E-10 5.969E-10 -5.689E-10

199.0625 1.357E-10 5.962E-10 -6.057E-10

199.2813 2.472E-10 7.458E-10 -6.748E-10

199.5000 4.233E-10 1.068E-09 -7.206E-10

199.7188 6.048E-10 1.497E-09 -6.948E-10

199.9375 7.434E-10 1.880E-09 -5.980E-10

200.1563 8.394E-10 2.054E-09 -4.575E-10

200.3750 9.196E-10 1.937E-09 -3.030E-10

200.5938 9.854E-10 1.587E-09 -1.825E-10

200.8125 1.001E-09 1.167E-09 -1.603E-10

201.0313 9.376E-10 8.493E-10 -2.518E-10

201.2500 8.043E-10 7.165E-10 -3.808E-10

201.4688 6.383E-10 7.354E-10 -4.407E-10

201.6875 4.767E-10 8.123E-10 -3.951E-10

201.9063 3.591E-10 8.694E-10 -2.972E-10

202.1250 3.288E-10 8.773E-10 -2.253E-10

202.3438 4.005E-10 8.472E-10 -2.242E-10

202.5625 5.304E-10 8.109E-10 -2.967E-10

202.7813 6.457E-10 7.899E-10 -4.137E-10

203.0000 7.098E-10 7.714E-10 -5.257E-10

203.2188 7.449E-10 7.303E-10 -5.899E-10

203.4375 7.806E-10 6.775E-10 -5.976E-10

203.6563 7.962E-10 6.624E-10 -5.735E-10

203.8750 7.300E-10 7.146E-10 -5.473E-10

204.0938 5.529E-10 8.083E-10 -5.325E-10

204.3125 3.208E-10 8.950E-10 -5.323E-10

204.5313 1.428E-10 9.452E-10 -5.603E-10

204.7500 9.838E-11 9.500E-10 -6.422E-10

204.9688 1.859E-10 9.077E-10 -7.871E-10

205.1875 3.425E-10 8.353E-10 -9.575E-10

205.4063 4.961E-10 7.746E-10 -1.083E-09

205.6250 5.963E-10 7.637E-10 -1.119E-09

205.8438 6.160E-10 7.997E-10 -1.083E-09

206.0625 5.563E-10 8.392E-10 -1.028E-09

206.2813 4.599E-10 8.332E-10 -9.805E-10

206.5000 3.994E-10 7.667E-10 -9.282E-10

206.7188 4.260E-10 6.698E-10 -8.554E-10

206.9375 5.147E-10 5.912E-10 -7.869E-10

207.1563 5.716E-10 5.535E-10 -7.745E-10

207.3750 5.129E-10 5.393E-10 -8.380E-10

207.5938 3.533E-10 5.245E-10 -9.223E-10

207.8125 2.058E-10 5.093E-10 -9.282E-10

208.0313 1.791E-10 5.040E-10 -8.021E-10

208.2500 2.712E-10 5.098E-10 -5.945E-10

208.4688 3.614E-10 5.216E-10 -4.381E-10

208.6875 2.906E-10 4.859E-10 -5.267E-10

208.9063 -2.674E-11 2.273E-10 -1.116E-09

209.1250 -4.984E-10 -4.195E-10 -2.340E-09

209.3438 -8.437E-10 -1.217E-09 -3.772E-09

209.5625 -7.890E-10 -1.404E-09 -4.325E-09

209.7813 -3.733E-10 -3.470E-10 -3.121E-09

210.0000 -7.671E-13 1.586E-09 -7.336E-10

210.2188 -2.531E-11 3.176E-09 9.811E-10

210.4375 -2.081E-10 3.682E-09 9.583E-10

210.6563 2.757E-10 3.802E-09 6.723E-10

210.8750 2.044E-09 4.915E-09 2.915E-09

211.0938 4.633E-09 7.344E-09 8.406E-09

211.3125 6.588E-09 9.712E-09 1.422E-08

211.5313 6.676E-09 1.021E-08 1.628E-08

211.7500 4.986E-09 8.332E-09 1.324E-08

211.9688 2.749E-09 5.242E-09 7.508E-09

212.1875 1.151E-09 2.546E-09 2.544E-09

212.4063 5.039E-10 1.032E-09 -6.930E-13

212.6250 4.397E-10 5.000E-10 -6.797E-10

212.8438 5.279E-10 4.177E-10 -7.119E-10

213.0625 5.697E-10 4.517E-10 -7.495E-10

213.2813 5.342E-10 5.090E-10 -8.393E-10

213.5000 4.535E-10 5.808E-10 -8.892E-10

213.7188 3.766E-10 6.739E-10 -8.915E-10

213.9375 3.326E-10 8.001E-10 -8.751E-10

214.1563 3.164E-10 9.432E-10 -8.486E-10

214.3750 3.143E-10 1.037E-09 -8.147E-10

214.5938 3.236E-10 1.010E-09 -7.836E-10

214.8125 3.467E-10 8.566E-10 -7.554E-10

215.0313 3.831E-10 6.684E-10 -7.197E-10

215.2500 4.298E-10 5.725E-10 -6.787E-10

215.4688 4.777E-10 6.362E-10 -6.440E-10

215.6875 5.138E-10 8.229E-10 -6.044E-10

215.9063 5.351E-10 1.029E-09 -5.375E-10

216.1250 5.497E-10 1.156E-09 -4.689E-10

216.3438 5.591E-10 1.160E-09 -4.769E-10

216.5625 5.521E-10 1.063E-09 -6.023E-10

216.7813 5.231E-10 9.264E-10 -7.814E-10

217.0000 4.882E-10 8.148E-10 -9.024E-10

217.2188 4.780E-10 7.541E-10 -9.141E-10

217.4375 5.174E-10 7.271E-10 -8.472E-10

217.6563 6.073E-10 6.968E-10 -7.488E-10

217.8750 7.119E-10 6.383E-10 -6.352E-10

218.0938 7.733E-10 5.575E-10 -5.176E-10

218.3125 7.592E-10 4.873E-10 -4.395E-10

218.5313 6.924E-10 4.647E-10 -4.604E-10

218.7500 6.121E-10 5.030E-10 -5.990E-10

218.9688 5.182E-10 5.732E-10 -7.971E-10

219.1875 3.871E-10 6.163E-10 -9.528E-10

219.4063 2.401E-10 5.910E-10 -1.004E-09

219.6250 1.500E-10 5.292E-10 -9.771E-10

219.8438 1.678E-10 5.283E-10 -9.423E-10

220.0625 2.740E-10 6.538E-10 -9.145E-10

220.2813 4.086E-10 8.454E-10 -8.398E-10

220.5000 5.198E-10 9.510E-10 -6.901E-10

220.7188 5.733E-10 8.777E-10 -5.337E-10

220.9375 5.615E-10 6.880E-10 -4.728E-10

221.1563 5.275E-10 5.270E-10 -5.287E-10

221.3750 5.477E-10 4.759E-10 -6.336E-10

221.5938 6.543E-10 5.048E-10 -7.289E-10

221.8125 7.837E-10 5.454E-10 -8.231E-10

222.0313 8.287E-10 5.629E-10 -9.436E-10

222.2500 7.378E-10 5.594E-10 -1.069E-09

222.4688 5.526E-10 5.620E-10 -1.132E-09

222.6875 3.607E-10 6.216E-10 -1.073E-09

222.9063 2.326E-10 7.772E-10 -8.854E-10

223.1250 1.904E-10 9.910E-10 -6.356E-10

223.3438 2.087E-10 1.149E-09 -4.333E-10

223.5625 2.392E-10 1.159E-09 -3.623E-10

223.7813 2.498E-10 1.046E-09 -4.128E-10

224.0000 2.478E-10 9.518E-10 -4.974E-10

224.2188 2.576E-10 1.044E-09 -5.438E-10

224.4375 2.782E-10 1.396E-09 -5.657E-10

224.6563 2.830E-10 1.897E-09 -6.424E-10

224.8750 2.658E-10 2.276E-09 -8.322E-10

225.0938 2.716E-10 2.285E-09 -1.100E-09

225.3125 3.555E-10 1.905E-09 -1.321E-09

225.5313 5.087E-10 1.372E-09 -1.368E-09

225.7500 6.400E-10 9.616E-10 -1.215E-09

225.9688 6.529E-10 7.635E-10 -9.541E-10

226.1875 5.400E-10 6.648E-10 -7.065E-10

226.4063 3.858E-10 5.358E-10 -5.310E-10

226.6250 2.776E-10 3.771E-10 -4.252E-10

226.8438 2.372E-10 2.834E-10 -3.773E-10

227.0625 2.529E-10 3.153E-10 -3.821E-10

227.2813 3.353E-10 4.444E-10 -4.215E-10

227.5000 4.978E-10 5.979E-10 -4.686E-10

227.7188 6.936E-10 7.113E-10 -5.161E-10

227.9375 8.195E-10 7.451E-10 -5.800E-10

228.1563 7.958E-10 6.946E-10 -6.705E-10

228.3750 6.261E-10 5.966E-10 -7.789E-10

228.5938 3.863E-10 5.064E-10 -8.890E-10

228.8125 1.777E-10 4.585E-10 -9.849E-10

229.0313 8.010E-11 4.592E-10 -1.048E-09

229.2500 1.071E-10 5.071E-10 -1.072E-09

229.4688 1.922E-10 5.990E-10 -1.078E-09

229.6875 2.450E-10 7.166E-10 -1.100E-09

229.9063 2.356E-10 8.270E-10 -1.138E-09

230.1250 2.108E-10 8.990E-10 -1.145E-09

230.3438 2.221E-10 9.142E-10 -1.080E-09

230.5625 2.593E-10 8.694E-10 -9.599E-10

230.7813 2.696E-10 7.858E-10 -8.413E-10

231.0000 2.297E-10 7.061E-10 -7.486E-10

231.2188 1.835E-10 6.661E-10 -6.507E-10

231.4375 2.053E-10 6.689E-10 -5.233E-10

231.6563 3.319E-10 7.053E-10 -4.090E-10

231.8750 5.300E-10 7.854E-10 -3.804E-10

232.0938 7.229E-10 9.198E-10 -4.476E-10

232.3125 8.462E-10 1.072E-09 -5.381E-10

232.5313 8.845E-10 1.166E-09 -5.751E-10

232.7500 8.707E-10 1.158E-09 -5.526E-10

232.9688 8.556E-10 1.074E-09 -5.158E-10

233.1875 8.701E-10 9.675E-10 -4.906E-10

233.4063 9.059E-10 8.683E-10 -4.585E-10

233.6250 9.284E-10 7.748E-10 -4.033E-10

233.8438 9.073E-10 6.740E-10 -3.564E-10

234.0625 8.423E-10 5.547E-10 -3.673E-10

234.2813 7.651E-10 4.253E-10 -4.347E-10

234.5000 7.093E-10 3.265E-10 -5.024E-10

234.7188 6.703E-10 3.018E-10 -5.318E-10

234.9375 6.055E-10 3.468E-10 -5.451E-10

235.1563 4.901E-10 4.092E-10 -5.794E-10

235.3750 3.620E-10 4.481E-10 -6.324E-10

235.5938 2.904E-10 4.720E-10 -6.826E-10

235.8125 3.027E-10 5.075E-10 -7.392E-10

236.0313 3.565E-10 5.473E-10 -8.359E-10

236.2500 3.834E-10 5.551E-10 -9.768E-10

236.4688 3.509E-10 5.214E-10 -1.112E-09

236.6875 2.847E-10 4.901E-10 -1.170E-09

236.9063 2.365E-10 5.078E-10 -1.117E-09

237.1250 2.328E-10 5.590E-10 -1.004E-09

237.3438 2.622E-10 5.869E-10 -9.391E-10

237.5625 3.018E-10 5.752E-10 -9.485E-10

237.7813 3.343E-10 5.722E-10 -9.854E-10

238.0000 3.571E-10 6.274E-10 -1.118E-09

238.2188 3.878E-10 7.315E-10 -8.844E-10

238.4375 4.189E-10 7.963E-10 2.798E-09

238.6563 4.588E-10 6.728E-10 1.598E-08

238.8750 9.409E-10 3.382E-10 4.155E-08

239.0938 3.084E-09 2.685E-10 6.991E-08

239.3125 8.025E-09 1.467E-09 7.988E-08

239.5313 1.482E-08 4.586E-09 5.613E-08

239.7500 1.983E-08 8.747E-09 7.252E-09

239.9688 1.948E-08 1.164E-08 -3.816E-08

240.1875 1.379E-08 1.137E-08 -5.614E-08

240.4063 6.503E-09 8.199E-09 -4.614E-08

240.6250 1.479E-09 4.257E-09 -2.530E-08

240.8438 -3.456E-10 1.571E-09 -9.494E-09

241.0625 -3.916E-10 6.211E-10 -2.821E-09

241.2813 -1.199E-10 6.479E-10 -1.519E-09

241.5000 3.229E-11 8.061E-10 -1.587E-09

241.7188 1.554E-10 7.932E-10 -1.567E-09

241.9375 2.921E-10 6.925E-10 -1.405E-09

242.1563 4.033E-10 6.228E-10 -1.236E-09

242.3750 4.624E-10 6.144E-10 -1.149E-09

242.5938 4.451E-10 6.516E-10 -1.185E-09

242.8125 3.463E-10 7.105E-10 -1.307E-09

243.0313 2.225E-10 7.635E-10 -1.426E-09

243.2500 1.542E-10 7.923E-10 -1.490E-09

243.4688 1.645E-10 8.015E-10 -1.516E-09

243.6875 2.039E-10 8.006E-10 -1.546E-09

243.9063 2.086E-10 7.779E-10 -1.569E-09

244.1250 1.611E-10 7.107E-10 -1.517E-09

244.3438 1.017E-10 6.013E-10 -1.345E-09

244.5625 8.428E-11 4.838E-10 -1.097E-09

244.7813 1.253E-10 3.936E-10 -8.676E-10

245.0000 1.986E-10 3.502E-10 -7.126E-10

245.2188 2.748E-10 3.719E-10 -6.148E-10

245.4375 3.454E-10 4.754E-10 -5.347E-10

245.6563 4.021E-10 6.314E-10 -4.671E-10

245.8750 4.170E-10 7.414E-10 -4.307E-10

246.0938 3.658E-10 7.129E-10 -4.147E-10

246.3125 2.647E-10 5.756E-10 -3.774E-10

246.5313 1.640E-10 4.817E-10 -3.169E-10

246.7500 1.079E-10 5.539E-10 -3.058E-10

246.9688 1.081E-10 7.482E-10 -4.159E-10

247.1875 1.512E-10 9.015E-10 -6.269E-10

247.4063 2.174E-10 9.106E-10 -8.469E-10

247.6250 2.892E-10 8.299E-10 -1.003E-09

247.8438 3.549E-10 7.968E-10 -1.072E-09

248.0625 4.062E-10 8.897E-10 -1.052E-09

248.2813 4.367E-10 1.074E-09 -9.512E-10

248.5000 4.445E-10 1.257E-09 -8.222E-10

248.7188 4.389E-10 1.364E-09 -7.395E-10

248.9375 4.334E-10 1.388E-09 -7.353E-10

249.1563 4.269E-10 1.390E-09 -7.770E-10

249.3750 4.021E-10 1.466E-09 -8.200E-10

249.5938 3.529E-10 1.664E-09 -8.573E-10

249.8125 3.049E-10 1.918E-09 -9.027E-10

250.0313 2.904E-10 2.085E-09 -9.408E-10

250.2500 3.065E-10 2.064E-09 -9.198E-10

250.4688 3.199E-10 1.884E-09 -8.092E-10

250.6875 3.206E-10 1.660E-09 -6.560E-10

250.9063 3.440E-10 1.490E-09 -5.569E-10

251.1250 4.144E-10 1.375E-09 -5.638E-10

251.3438 4.888E-10 1.247E-09 -6.364E-10

251.5625 4.991E-10 1.051E-09 -7.017E-10

251.7813 4.487E-10 8.193E-10 -7.415E-10

252.0000 4.177E-10 6.530E-10 -7.941E-10

252.2188 4.554E-10 6.144E-10 -8.714E-10

252.4375 5.076E-10 6.566E-10 -9.076E-10

252.6563 4.877E-10 6.756E-10 -8.177E-10

252.8750 3.937E-10 6.222E-10 -6.025E-10

253.0938 3.163E-10 5.383E-10 -3.714E-10

253.3125 3.333E-10 4.888E-10 -2.455E-10

253.5313 4.261E-10 4.864E-10 -2.479E-10

253.7500 5.010E-10 4.926E-10 -3.056E-10

253.9688 4.776E-10 4.786E-10 -3.455E-10

254.1875 3.537E-10 4.678E-10 -3.609E-10

254.4063 2.006E-10 5.111E-10 -3.849E-10

254.6250 1.035E-10 6.228E-10 -4.406E-10

254.8438 1.002E-10 7.508E-10 -5.370E-10

255.0625 1.723E-10 8.106E-10 -6.777E-10

255.2813 2.813E-10 7.493E-10 -8.383E-10

255.5000 4.003E-10 5.845E-10 -9.592E-10

255.7188 5.121E-10 3.961E-10 -9.984E-10

255.9375 5.957E-10 2.753E-10 -9.758E-10

256.1563 6.369E-10 2.673E-10 -9.358E-10

256.3750 6.487E-10 3.519E-10 -8.880E-10

256.5938 6.611E-10 4.759E-10 -8.211E-10

256.8125 6.821E-10 5.936E-10 -7.513E-10

257.0313 6.856E-10 6.816E-10 -7.018E-10

257.2500 6.579E-10 7.357E-10 -6.482E-10

257.4688 6.451E-10 7.670E-10 -5.459E-10

257.6875 7.185E-10 7.890E-10 -4.244E-10

257.9063 8.778E-10 7.972E-10 -3.937E-10

258.1250 1.019E-09 7.654E-10 -5.151E-10

258.3438 1.028E-09 6.723E-10 -7.009E-10

258.5625 8.950E-10 5.281E-10 -7.858E-10

258.7813 7.079E-10 3.717E-10 -6.932E-10

259.0000 5.525E-10 2.468E-10 -5.043E-10

259.2188 4.382E-10 1.868E-10 -3.762E-10

259.4375 3.242E-10 2.126E-10 -4.203E-10

259.6563 1.878E-10 3.250E-10 -6.362E-10

259.8750 5.925E-11 4.897E-10 -9.132E-10

260.0938 4.508E-12 6.431E-10 -1.098E-09

260.3125 7.640E-11 7.292E-10 -1.112E-09

260.5313 2.624E-10 7.365E-10 -1.026E-09

260.7500 4.729E-10 7.011E-10 -9.840E-10

260.9688 5.935E-10 6.733E-10 -1.047E-09

261.1875 5.672E-10 6.756E-10 -1.135E-09

261.4063 4.367E-10 6.883E-10 -1.129E-09

261.6250 3.136E-10 6.759E-10 -1.003E-09

261.8438 3.004E-10 6.266E-10 -8.411E-10

262.0625 4.215E-10 5.624E-10 -7.455E-10

262.2813 6.127E-10 5.168E-10 -7.419E-10

262.5000 7.757E-10 5.156E-10 -7.640E-10

262.7188 8.502E-10 5.727E-10 -7.281E-10

262.9375 8.383E-10 6.827E-10 -6.269E-10

263.1563 7.761E-10 8.079E-10 -5.419E-10

263.3750 6.927E-10 8.871E-10 -5.542E-10

263.5938 5.930E-10 8.723E-10 -6.561E-10

263.8125 4.634E-10 7.640E-10 -7.733E-10

264.0313 2.944E-10 6.127E-10 -8.669E-10

264.2500 1.146E-10 4.873E-10 -9.718E-10

264.4688 2.747E-13 4.357E-10 -1.118E-09

264.6875 2.378E-11 4.649E-10 -1.246E-09

264.9063 1.753E-10 5.446E-10 -1.230E-09

265.1250 3.522E-10 6.325E-10 -1.013E-09

265.3438 4.476E-10 7.076E-10 -6.809E-10

265.5625 4.471E-10 7.843E-10 -4.153E-10

265.7813 4.235E-10 8.873E-10 -3.515E-10

266.0000 4.410E-10 1.008E-09 -4.887E-10

266.2188 4.846E-10 1.096E-09 -7.124E-10

266.4375 4.898E-10 1.091E-09 -8.853E-10

266.6563 4.246E-10 9.767E-10 -9.262E-10

266.8750 3.284E-10 7.888E-10 -8.437E-10

267.0938 2.786E-10 6.014E-10 -7.167E-10

267.3125 3.285E-10 4.978E-10 -6.281E-10

267.5313 4.641E-10 5.326E-10 -5.986E-10

267.7500 6.086E-10 6.891E-10 -5.926E-10

267.9688 6.808E-10 8.639E-10 -5.863E-10

268.1875 6.629E-10 9.140E-10 -5.969E-10

268.4063 6.051E-10 7.547E-10 -6.361E-10

268.6250 5.552E-10 4.332E-10 -6.777E-10

268.8438 5.026E-10 1.076E-10 -7.010E-10

269.0625 4.125E-10 -6.292E-11 -7.321E-10

269.2813 2.993E-10 -2.839E-11 -8.087E-10

269.5000 2.280E-10 1.332E-10 -9.220E-10

269.7188 2.383E-10 2.969E-10 -1.026E-09

269.9375 2.932E-10 3.912E-10 -1.095E-09

270.1563 3.244E-10 4.303E-10 -1.147E-09

270.3750 3.110E-10 4.628E-10 -1.206E-09

270.5938 2.926E-10 5.062E-10 -1.266E-09

270.8125 3.048E-10 5.404E-10 -1.291E-09

271.0313 3.300E-10 5.544E-10 -1.256E-09

271.2500 3.246E-10 5.710E-10 -1.178E-09

271.4688 2.809E-10 6.092E-10 -1.096E-09

271.6875 2.355E-10 6.401E-10 -1.040E-09

271.9063 2.179E-10 6.124E-10 -1.024E-09

272.1250 2.167E-10 5.188E-10 -1.067E-09

272.3438 2.106E-10 4.097E-10 -1.177E-09

272.5625 2.088E-10 3.377E-10 -1.302E-09

272.7813 2.335E-10 3.127E-10 -1.346E-09

273.0000 2.704E-10 3.214E-10 -1.252E-09

273.2188 2.688E-10 3.610E-10 -1.065E-09

273.4375 2.006E-10 4.247E-10 -8.909E-10

273.6563 9.737E-11 4.756E-10 -7.827E-10

273.8750 2.547E-11 4.840E-10 -7.058E-10

274.0938 3.663E-11 5.079E-10 -6.055E-10

274.3125 1.421E-10 6.869E-10 -4.763E-10

274.5313 3.069E-10 1.086E-09 -3.583E-10

274.7500 4.597E-10 1.549E-09 -3.015E-10

274.9688 5.338E-10 1.778E-09 -3.483E-10

275.1875 5.177E-10 1.611E-09 -5.111E-10

275.4063 4.601E-10 1.184E-09 -7.342E-10

275.6250 4.188E-10 7.956E-10 -9.043E-10

275.8438 4.123E-10 6.326E-10 -9.402E-10

276.0625 4.260E-10 6.448E-10 -8.664E-10

276.2813 4.448E-10 6.662E-10 -7.756E-10

276.5000 4.620E-10 6.058E-10 -7.342E-10

276.7188 4.697E-10 5.160E-10 -7.441E-10

276.9375 4.612E-10 4.998E-10 -7.705E-10

277.1563 4.461E-10 5.775E-10 -7.709E-10

277.3750 4.491E-10 6.574E-10 -7.212E-10

277.5938 4.901E-10 6.320E-10 -6.536E-10

277.8125 5.736E-10 4.883E-10 -6.573E-10

278.0313 6.889E-10 3.161E-10 -7.929E-10

278.2500 8.072E-10 2.194E-10 -1.003E-09

278.4688 8.807E-10 2.322E-10 -1.142E-09

278.6875 8.618E-10 3.083E-10 -1.117E-09

278.9063 7.379E-10 3.734E-10 -9.702E-10

279.1250 5.486E-10 3.806E-10 -8.393E-10

279.3438 3.703E-10 3.325E-10 -8.310E-10

279.5625 2.752E-10 2.661E-10 -9.488E-10

279.7813 2.855E-10 2.237E-10 -1.111E-09

280.0000 3.549E-10 2.299E-10 -1.220E-09

280.2188 4.061E-10 2.897E-10 -1.223E-09

280.4375 3.996E-10 4.010E-10 -1.129E-09

280.6563 3.621E-10 5.581E-10 -9.929E-10

280.8750 3.444E-10 7.342E-10 -8.758E-10

281.0938 3.659E-10 8.726E-10 -8.207E-10

281.3125 4.084E-10 9.163E-10 -8.335E-10

281.5313 4.438E-10 8.556E-10 -8.753E-10

281.7500 4.474E-10 7.304E-10 -8.832E-10

281.9688 3.999E-10 5.793E-10 -8.210E-10

282.1875 3.090E-10 4.061E-10 -7.172E-10

282.4063 2.295E-10 2.109E-10 -6.437E-10

282.6250 2.351E-10 3.888E-11 -6.552E-10

282.8438 3.467E-10 -3.481E-11 -7.488E-10

283.0625 4.952E-10 2.453E-11 -8.744E-10

283.2813 5.665E-10 1.698E-10 -9.849E-10

283.5000 4.870E-10 3.053E-10 -1.094E-09

283.7188 2.701E-10 3.466E-10 -1.286E-09

283.9375 -2.988E-12 2.441E-10 -1.639E-09

284.1563 -2.346E-10 -1.340E-11 -2.108E-09

284.3750 -3.559E-10 -3.674E-10 -2.499E-09

284.5938 -3.549E-10 -6.610E-10 -2.588E-09

284.8125 -2.784E-10 -7.013E-10 -2.296E-09

285.0313 -1.910E-10 -4.138E-10 -1.762E-09

285.2500 -1.209E-10 6.378E-11 -1.236E-09

285.4688 -5.560E-11 4.699E-10 -9.124E-10

285.6875 1.104E-11 6.196E-10 -8.394E-10

285.9063 5.466E-11 5.185E-10 -9.536E-10

286.1250 5.769E-11 3.066E-10 -1.142E-09

286.3438 4.680E-11 1.249E-10 -1.278E-09

286.5625 6.912E-11 3.492E-11 -1.262E-09

286.7813 1.391E-10 2.536E-11 -1.098E-09

287.0000 2.236E-10 5.530E-11 -9.045E-10

287.2188 2.747E-10 9.264E-11 -8.177E-10

287.4375 2.716E-10 1.402E-10 -8.650E-10

287.6563 2.334E-10 2.421E-10 -9.434E-10

287.8750 1.959E-10 4.464E-10 -9.375E-10

288.0938 1.793E-10 7.366E-10 -8.433E-10

288.3125 1.866E-10 1.005E-09 -7.510E-10

288.5313 2.245E-10 1.120E-09 -7.202E-10

288.7500 3.004E-10 1.039E-09 -7.192E-10

288.9688 3.946E-10 8.335E-10 -7.029E-10

289.1875 4.667E-10 6.102E-10 -7.019E-10

289.4063 5.034E-10 4.234E-10 -7.898E-10

289.6250 5.374E-10 2.723E-10 -9.782E-10

289.8438 6.012E-10 1.665E-10 -1.177E-09

290.0625 6.808E-10 1.552E-10 -1.260E-09

290.2813 7.261E-10 2.721E-10 -1.162E-09

290.5000 6.947E-10 4.647E-10 -9.275E-10

290.7188 5.813E-10 6.097E-10 -6.801E-10

290.9375 4.245E-10 6.177E-10 -5.357E-10

291.1563 2.974E-10 5.235E-10 -5.338E-10

291.3750 2.698E-10 4.591E-10 -6.389E-10

291.5938 3.625E-10 5.238E-10 -7.896E-10

291.8125 5.307E-10 6.809E-10 -9.306E-10

292.0313 6.884E-10 7.843E-10 -1.018E-09

292.2500 7.526E-10 7.145E-10 -1.031E-09

292.4688 6.819E-10 4.905E-10 -9.863E-10

292.6875 5.045E-10 2.567E-10 -9.267E-10

292.9063 3.109E-10 1.687E-10 -8.788E-10

293.1250 1.937E-10 2.724E-10 -8.250E-10

293.3438 1.742E-10 4.676E-10 -7.294E-10

293.5625 1.979E-10 5.823E-10 -5.910E-10

293.7813 2.140E-10 5.133E-10 -4.593E-10

294.0000 2.386E-10 3.152E-10 -3.887E-10

294.2188 3.183E-10 1.458E-10 -3.841E-10

294.4375 4.430E-10 1.189E-10 -4.100E-10

294.6563 5.306E-10 2.131E-10 -4.434E-10

294.8750 5.026E-10 3.175E-10 -4.947E-10

295.0938 3.635E-10 3.464E-10 -5.614E-10

295.3125 1.975E-10 3.036E-10 -5.923E-10

295.5313 9.342E-11 2.511E-10 -5.363E-10

295.7500 7.987E-11 2.373E-10 -4.118E-10

295.9688 1.289E-10 2.682E-10 -2.946E-10

296.1875 2.044E-10 3.363E-10 -2.679E-10

296.4063 2.906E-10 4.450E-10 -3.945E-10

296.6250 3.833E-10 5.800E-10 -6.404E-10

296.8438 4.655E-10 6.849E-10 -8.530E-10

297.0625 5.045E-10 7.057E-10 -9.516E-10

297.2813 4.878E-10 6.424E-10 -9.362E-10

297.5000 4.380E-10 5.215E-10 -6.731E-10

297.7188 3.603E-10 3.552E-10 -4.673E-10

297.9375 2.297E-10 1.704E-10 -6.149E-10

298.1563 4.074E-11 3.164E-11 6.045E-09

298.3750 -2.307E-10 -5.007E-12 4.523E-08

298.5938 -3.441E-10 4.753E-11 1.523E-07

298.8125 1.227E-09 -1.354E-10 3.272E-07

299.0313 7.090E-09 -1.834E-09 4.909E-07

299.2500 1.670E-08 -7.533E-09 5.134E-07

299.4688 2.166E-08 -1.922E-08 3.220E-07

299.6875 9.977E-09 -3.518E-08 -1.163E-08

299.9063 -2.006E-08 -4.887E-08 -3.104E-07

300.1250 -5.255E-08 -5.284E-08 -4.312E-07

300.3438 -6.620E-08 -4.474E-08 -3.665E-07

300.5625 -5.459E-08 -2.937E-08 -2.174E-07

300.7813 -3.048E-08 -1.446E-08 -8.970E-08

301.0000 -1.077E-08 -4.873E-09 -2.374E-08

301.2188 -1.857E-09 -7.432E-10 -3.620E-09

301.4375 4.998E-11 3.080E-10 -1.020E-09

301.6563 1.247E-10 3.546E-10 -1.048E-09

301.8750 2.701E-10 2.721E-10 -8.344E-10

302.0938 4.124E-10 2.445E-10 -6.960E-10

302.3125 4.461E-10 2.992E-10 -6.099E-10

302.5313 4.304E-10 4.154E-10 -5.186E-10

302.7500 3.329E-10 4.956E-10 -5.562E-10

302.9688 1.404E-10 4.395E-10 -7.793E-10

303.1875 -5.156E-11 2.637E-10 -1.089E-09

303.4063 -1.385E-10 1.024E-10 -1.328E-09

303.6250 -9.152E-11 6.877E-11 -1.379E-09

303.8438 4.802E-11 1.441E-10 -1.243E-09

304.0625 2.138E-10 2.310E-10 -1.028E-09

304.2813 3.528E-10 2.805E-10 -8.464E-10

304.5000 4.328E-10 3.264E-10 -7.563E-10

304.7188 4.384E-10 4.160E-10 -7.610E-10

304.9375 3.867E-10 5.462E-10 -8.322E-10

305.1563 3.269E-10 6.646E-10 -9.284E-10

305.3750 2.971E-10 7.095E-10 -1.029E-09

305.5938 2.894E-10 6.552E-10 -1.141E-09

305.8125 2.750E-10 5.371E-10 -1.261E-09

306.0313 2.527E-10 4.267E-10 -1.331E-09

306.2500 2.536E-10 3.722E-10 -1.285E-09

306.4688 3.051E-10 3.670E-10 -1.145E-09

306.6875 4.029E-10 3.759E-10 -1.029E-09

306.9063 5.109E-10 3.774E-10 -1.037E-09

307.1250 5.781E-10 3.693E-10 -1.143E-09

307.3438 5.675E-10 3.545E-10 -1.248E-09

307.5625 4.906E-10 3.434E-10 -1.300E-09

307.7813 4.078E-10 3.621E-10 -1.317E-09

308.0000 3.796E-10 4.300E-10 -1.296E-09

308.2188 4.169E-10 5.243E-10 -1.179E-09

308.4375 4.812E-10 5.802E-10 -9.292E-10

308.6563 5.257E-10 5.358E-10 -6.008E-10

308.8750 5.338E-10 3.786E-10 -3.179E-10

309.0938 5.295E-10 1.629E-10 -1.915E-10

309.3125 5.543E-10 -7.900E-12 -2.552E-10

309.5313 6.292E-10 -3.303E-11 -4.436E-10

309.7500 7.322E-10 1.164E-10 -6.297E-10

309.9688 8.071E-10 3.660E-10 -7.156E-10

310.1875 7.954E-10 5.861E-10 -7.007E-10

310.4063 6.713E-10 6.855E-10 -6.598E-10

310.6250 4.656E-10 6.670E-10 -6.654E-10

310.8438 2.556E-10 6.007E-10 -7.302E-10

311.0625 1.207E-10 5.508E-10 -8.030E-10

311.2813 8.941E-11 5.294E-10 -8.087E-10

311.5000 1.206E-10 5.107E-10 -7.137E-10

311.7188 1.416E-10 4.747E-10 -5.722E-10

311.9375 1.149E-10 4.239E-10 -5.006E-10

312.1563 7.088E-11 3.654E-10 -5.729E-10

312.3750 7.390E-11 2.887E-10 -7.394E-10

312.5938 1.588E-10 1.788E-10 -8.696E-10

312.8125 3.043E-10 5.243E-11 -8.784E-10

313.0313 4.523E-10 -2.902E-11 -7.964E-10

313.2500 5.452E-10 -4.330E-12 -7.249E-10

313.4688 5.645E-10 1.307E-10 -7.435E-10

313.6875 5.532E-10 3.232E-10 -8.645E-10

313.9063 5.817E-10 5.154E-10 -1.060E-09

314.1250 6.706E-10 6.806E-10 -1.302E-09

314.3438 7.607E-10 8.141E-10 -1.546E-09

314.5625 7.710E-10 9.093E-10 -1.696E-09

314.7813 6.773E-10 9.512E-10 -1.625E-09

315.0000 5.204E-10 9.260E-10 -1.292E-09

315.2188 3.577E-10 8.286E-10 -8.317E-10

315.4375 2.315E-10 6.680E-10 -4.723E-10

315.6563 1.729E-10 4.727E-10 -3.499E-10

315.8750 1.970E-10 2.842E-10 -4.269E-10

316.0938 2.972E-10 1.539E-10 -5.724E-10

316.3125 4.593E-10 1.422E-10 -6.564E-10

316.5313 6.669E-10 2.929E-10 -5.870E-10

316.7500 8.756E-10 5.812E-10 -3.495E-10

316.9688 1.007E-09 8.955E-10 -5.307E-11

317.1875 9.944E-10 1.094E-09 1.070E-10

317.4063 8.339E-10 1.094E-09 4.385E-12

317.6250 5.975E-10 9.192E-10 -2.810E-10

317.8438 3.808E-10 6.674E-10 -5.319E-10

318.0625 2.311E-10 4.483E-10 -6.116E-10

318.2813 1.311E-10 3.370E-10 -5.587E-10

318.5000 5.972E-11 3.478E-10 -4.782E-10

318.7188 4.525E-11 4.327E-10 -4.153E-10

318.9375 1.267E-10 5.285E-10 -3.544E-10

319.1563 2.704E-10 6.080E-10 -2.902E-10

319.3750 3.681E-10 6.647E-10 -2.447E-10

319.5938 3.420E-10 6.708E-10 -2.058E-10

319.8125 2.193E-10 5.895E-10 -1.292E-10

320.0313 8.466E-11 4.291E-10 -3.664E-11

320.2500 1.264E-12 2.584E-10 -9.955E-12

320.4688 -8.448E-12 1.432E-10 -6.132E-11

320.6875 5.308E-11 8.342E-11 -1.029E-10

320.9063 1.720E-10 4.796E-11 -6.762E-11

321.1250 3.279E-10 3.416E-11 -4.418E-12

321.3438 4.855E-10 4.264E-11 -3.011E-11

321.5625 5.945E-10 4.534E-11 -1.915E-10

321.7813 6.140E-10 3.290E-11 -4.150E-10

322.0000 5.459E-10 4.415E-11 -6.021E-10

322.2188 4.416E-10 1.124E-10 -7.002E-10

322.4375 3.556E-10 2.168E-10 -7.022E-10

322.6563 2.878E-10 3.096E-10 -6.570E-10

322.8750 1.991E-10 3.718E-10 -6.420E-10

323.0938 7.420E-11 4.209E-10 -6.856E-10

323.3125 -5.735E-11 4.587E-10 -7.606E-10

323.5313 -1.288E-10 4.457E-10 -8.045E-10

323.7500 -6.860E-11 3.438E-10 -7.479E-10

323.9688 1.152E-10 1.787E-10 -6.263E-10

324.1875 3.227E-10 7.489E-11 -5.762E-10

324.4063 4.754E-10 1.844E-10 -6.480E-10

324.6250 5.712E-10 5.232E-10 -7.653E-10

324.8438 6.182E-10 9.323E-10 -8.421E-10

325.0625 5.963E-10 1.226E-09 -8.482E-10

325.2813 4.898E-10 1.313E-09 -8.333E-10

325.5000 3.410E-10 1.219E-09 -8.315E-10

325.7188 2.467E-10 1.048E-09 -7.585E-10

325.9375 2.502E-10 8.639E-10 -6.169E-10

326.1563 2.863E-10 6.627E-10 -5.876E-10

326.3750 2.721E-10 5.012E-10 -7.213E-10

326.5938 1.792E-10 4.685E-10 -9.123E-10

326.8125 5.247E-11 5.324E-10 -1.127E-09

327.0313 8.859E-12 6.005E-10 -1.287E-09

327.2500 9.451E-11 6.403E-10 -1.271E-09

327.4688 2.010E-10 6.585E-10 -1.123E-09

327.6875 2.463E-10 6.729E-10 -9.074E-10

327.9063 2.403E-10 6.593E-10 -6.699E-10

328.1250 1.740E-10 5.618E-10 -5.481E-10

328.3438 9.165E-11 4.351E-10 -4.979E-10

328.5625 9.481E-11 3.438E-10 -4.034E-10

328.7813 1.655E-10 2.068E-10 -4.442E-10

329.0000 2.344E-10 5.092E-11 -5.998E-10

329.2188 2.698E-10 3.405E-11 -5.853E-10

329.4375 2.177E-10 1.095E-10 -5.554E-10

329.6563 1.482E-10 1.806E-10 -6.774E-10

329.8750 1.719E-10 2.476E-10 -8.244E-10

330.0938 1.511E-10 1.949E-10 -1.201E-09

330.3125 4.900E-11 1.204E-10 -1.641E-09

330.5313 8.397E-11 3.699E-10 -1.447E-09

330.7500 1.930E-10 7.330E-10 -9.771E-10

330.9688 2.515E-10 7.503E-10 -8.113E-10

331.1875 3.695E-10 5.213E-10 -5.891E-10

331.4063 3.445E-10 2.141E-10 -6.556E-10

331.6250 1.290E-10 -2.990E-11 -1.183E-09

331.8438 2.394E-10 7.945E-11 -1.042E-09

332.0625 4.778E-10 1.710E-10 -8.481E-10

332.2813 3.333E-10 -1.005E-11 -1.491E-09

332.5000 2.141E-10 2.141E-10 -1.313E-09

332.7188 3.871E-11 5.054E-10 -8.609E-10

332.9375 -4.443E-10 1.870E-10 -1.614E-09

333.1563 -1.946E-10 2.049E-10 -1.157E-09

333.3750 3.881E-10 2.305E-10 -6.681E-10

333.5938 2.132E-11 -6.533E-10 -2.850E-09

333.8125 1.082E-10 -4.194E-10 -2.481E-09

334.0313 4.090E-10 4.650E-10 -7.537E-10

334.2500 -9.537E-10 -6.772E-10 -4.344E-09

334.4688 -9.851E-10 -7.450E-10 -4.325E-09

334.6875 4.852E-11 1.310E-10 -8.824E-10

334.9063 -2.519E-09 -2.939E-09 -7.948E-09

335.1250 -2.839E-09 -3.188E-09 -8.788E-09

335.3438 1.752E-10 3.274E-10 -1.015E-09

335.5625 -4.451E-09 -5.089E-09 -1.707E-08

335.7813 -5.750E-09 -6.709E-09 -2.162E-08

336.0000 1.552E-10 5.267E-10 -8.609E-10

336.2188 -1.481E-08 -1.548E-08 -4.123E-08

336.4375 -2.204E-08 -2.387E-08 -6.281E-08

336.6563 2.764E-10 2.776E-10 -1.185E-09

336.8750 -3.850E-08 -4.329E-08 -1.218E-07

337.0938 -7.307E-08 -8.169E-08 -2.257E-07

337.3125 -3.761E-10 -9.392E-10 -4.763E-09

337.5313 -1.716E-07 -1.959E-07 -5.090E-07

337.7500 -4.064E-07 -4.634E-07 -1.209E-06

337.9688 -1.991E-08 -2.540E-08 -6.129E-08

338.1875 -1.148E-06 -1.257E-06 -3.416E-06

338.4063 -4.061E-06 -4.481E-06 -1.208E-05

338.6250 -4.937E-07 -5.280E-07 -1.461E-06

338.8438 -2.637E-05 -2.970E-05 -7.866E-05

339.0625 -2.827E-04 -3.171E-04 -8.427E-04

339.2813 -1.126E-03 -1.261E-03 -3.354E-03

339.5000 -2.715E-03 -3.041E-03 -8.090E-03

339.7188 -4.582E-03 -5.130E-03 -1.365E-02

339.9375 -5.723E-03 -6.408E-03 -1.705E-02

340.1563 -5.399E-03 -6.045E-03 -1.609E-02

340.3750 -3.827E-03 -4.285E-03 -1.140E-02

340.5938 -1.971E-03 -2.207E-03 -5.872E-03

340.8125 -6.782E-04 -7.598E-04 -2.021E-03

341.0313 -1.244E-04 -1.395E-04 -3.709E-04

341.2500 -3.866E-06 -4.380E-06 -1.159E-05

341.4688 -2.939E-06 -3.246E-06 -8.698E-06

341.6875 -3.159E-06 -3.495E-06 -9.355E-06

341.9063 -2.646E-07 -2.859E-07 -7.737E-07

342.1250 -2.090E-07 -2.403E-07 -6.361E-07

342.3438 -3.643E-07 -4.141E-07 -1.101E-06

342.5625 -4.472E-08 -5.117E-08 -1.381E-07

342.7813 -3.600E-08 -3.823E-08 -1.036E-07

343.0000 -8.223E-08 -8.984E-08 -2.410E-07

343.2188 -1.430E-08 -1.502E-08 -4.245E-08

343.4375 -7.441E-09 -8.164E-09 -2.391E-08

343.6563 -2.371E-08 -2.641E-08 -7.185E-08

343.8750 -5.334E-09 -5.651E-09 -1.689E-08

344.0938 -1.910E-09 -1.746E-09 -7.050E-09

344.3125 -8.117E-09 -8.672E-09 -2.512E-08

344.5313 -2.186E-09 -1.825E-09 -6.755E-09

344.7500 -1.039E-09 -7.293E-11 -3.787E-09

344.9688 -4.026E-09 -3.123E-09 -1.371E-08

345.1875 -1.361E-09 -5.307E-10 -5.759E-09

345.4063 -1.354E-10 3.660E-11 -1.258E-09

345.6250 -1.345E-09 -1.752E-09 -4.600E-09

345.8438 -3.661E-10 -5.072E-10 -2.079E-09

346.0625 -1.138E-10 8.707E-11 -1.265E-09

346.2813 -1.111E-09 -8.248E-10 -3.518E-09

346.5000 -6.600E-10 -1.798E-10 -1.667E-09

346.7188 -1.190E-10 4.257E-10 -3.217E-10

346.9375 -2.561E-10 -7.648E-12 -1.455E-09

347.1563 5.613E-11 -5.930E-12 -8.583E-10

347.3750 4.680E-11 -4.788E-11 -2.702E-10

347.5938 -4.920E-10 -3.904E-10 -9.256E-10

347.8125 -4.018E-10 -1.281E-10 -5.934E-10

348.0313 -5.097E-13 2.384E-10 -2.724E-10

348.2500 3.980E-11 2.039E-10 -7.418E-10

348.4688 -1.430E-11 2.164E-10 -5.899E-10

348.6875 -1.676E-10 1.216E-10 -5.190E-10

348.9063 -4.048E-10 -1.434E-10 -1.177E-09

349.1250 -3.498E-10 2.019E-11 -1.328E-09

349.3438 -1.435E-10 5.756E-10 -1.035E-09

349.5625 -5.278E-11 1.062E-09 -1.008E-09

349.7813 5.001E-11 1.406E-09 -9.242E-10

350.0000 1.357E-10 1.552E-09 -8.911E-10

**Data plotted in FIG 3(b)**

Frequency Cells in LB, -41.4 min Cells in LB, -5.3 min Cells in LB, 7.2 min

0.2188 2.710E-05 3.720E-06 2.295E-05

0.4375 1.763E-05 2.200E-06 1.449E-05

0.6563 8.227E-06 7.872E-07 6.274E-06

0.8750 2.493E-06 6.082E-08 1.537E-06

1.0938 3.972E-07 -7.049E-08 6.419E-08

1.3125 6.319E-08 1.376E-08 2.490E-08

1.5313 8.205E-08 7.721E-08 1.486E-07

1.7500 6.399E-08 9.189E-08 1.585E-07

1.9688 4.201E-08 9.318E-08 1.407E-07

2.1875 3.600E-08 9.389E-08 1.309E-07

2.4063 3.168E-08 9.189E-08 1.195E-07

2.6250 2.826E-08 8.738E-08 1.084E-07

2.8438 2.748E-08 8.213E-08 1.015E-07

3.0625 2.666E-08 7.676E-08 9.733E-08

3.2813 2.513E-08 7.120E-08 9.487E-08

3.5000 2.398E-08 6.520E-08 9.255E-08

3.7188 2.381E-08 5.894E-08 8.766E-08

3.9375 2.499E-08 5.335E-08 7.974E-08

4.1563 2.697E-08 4.944E-08 7.121E-08

4.3750 2.804E-08 4.754E-08 6.471E-08

4.5938 2.705E-08 4.726E-08 6.104E-08

4.8125 2.433E-08 4.762E-08 5.942E-08

5.0313 2.108E-08 4.761E-08 5.896E-08

5.2500 1.839E-08 4.681E-08 5.921E-08

5.4688 1.654E-08 4.527E-08 5.948E-08

5.6875 1.535E-08 4.321E-08 5.884E-08

5.9063 1.473E-08 4.110E-08 5.692E-08

6.1250 1.479E-08 3.954E-08 5.393E-08

6.3438 1.533E-08 3.874E-08 5.003E-08

6.5625 1.593E-08 3.818E-08 4.533E-08

6.7813 1.622E-08 3.715E-08 4.052E-08

7.0000 1.605E-08 3.540E-08 3.698E-08

7.2188 1.552E-08 3.325E-08 3.580E-08

7.4375 1.479E-08 3.124E-08 3.658E-08

7.6563 1.396E-08 2.978E-08 3.750E-08

7.8750 1.304E-08 2.897E-08 3.718E-08

8.0938 1.222E-08 2.851E-08 3.604E-08

8.3125 1.174E-08 2.799E-08 3.542E-08

8.5313 1.159E-08 2.726E-08 3.585E-08

8.7500 1.147E-08 2.647E-08 3.657E-08

8.9688 1.112E-08 2.572E-08 3.675E-08

9.1875 1.064E-08 2.495E-08 3.643E-08

9.4063 1.036E-08 2.403E-08 3.607E-08

9.6250 1.031E-08 2.293E-08 3.567E-08

9.8438 1.020E-08 2.178E-08 3.475E-08

10.0625 9.778E-09 2.074E-08 3.300E-08

10.2813 9.160E-09 1.988E-08 3.066E-08

10.5000 8.716E-09 1.910E-08 2.838E-08

10.7188 8.732E-09 1.832E-08 2.677E-08

10.9375 9.137E-09 1.768E-08 2.599E-08

11.1563 9.554E-09 1.747E-08 2.564E-08

11.3750 9.612E-09 1.772E-08 2.503E-08

11.5938 9.240E-09 1.809E-08 2.378E-08

11.8125 8.642E-09 1.809E-08 2.222E-08

12.0313 8.037E-09 1.771E-08 2.118E-08

12.2500 7.519E-09 1.751E-08 2.111E-08

12.4688 7.144E-09 1.814E-08 2.169E-08

12.6875 6.970E-09 1.944E-08 2.207E-08

12.9063 6.913E-09 2.044E-08 2.182E-08

13.1250 6.745E-09 2.024E-08 2.119E-08

13.3438 6.407E-09 1.892E-08 2.057E-08

13.5625 6.224E-09 1.727E-08 1.996E-08

13.7813 6.570E-09 1.590E-08 1.915E-08

14.0000 7.376E-09 1.501E-08 1.847E-08

14.2188 8.124E-09 1.461E-08 1.851E-08

14.4375 8.364E-09 1.461E-08 1.934E-08

14.6563 8.090E-09 1.471E-08 2.020E-08

14.8750 7.578E-09 1.464E-08 2.025E-08

15.0938 7.025E-09 1.449E-08 1.939E-08

15.3125 6.433E-09 1.459E-08 1.820E-08

15.5313 5.773E-09 1.489E-08 1.721E-08

15.7500 5.138E-09 1.491E-08 1.666E-08

15.9688 4.743E-09 1.438E-08 1.658E-08

16.1875 4.746E-09 1.366E-08 1.699E-08

16.4063 5.079E-09 1.337E-08 1.791E-08

16.6250 5.447E-09 1.375E-08 1.921E-08

16.8438 5.576E-09 1.446E-08 2.048E-08

17.0625 5.452E-09 1.493E-08 2.117E-08

17.2813 5.284E-09 1.485E-08 2.083E-08

17.5000 5.271E-09 1.425E-08 1.946E-08

17.7188 5.440E-09 1.336E-08 1.762E-08

17.9375 5.673E-09 1.252E-08 1.614E-08

18.1563 5.785E-09 1.204E-08 1.549E-08

18.3750 5.659E-09 1.203E-08 1.547E-08

18.5938 5.360E-09 1.233E-08 1.538E-08

18.8125 5.111E-09 1.258E-08 1.477E-08

19.0313 5.049E-09 1.253E-08 1.378E-08

19.2500 5.072E-09 1.222E-08 1.289E-08

19.4688 5.008E-09 1.189E-08 1.238E-08

19.6875 4.889E-09 1.170E-08 1.219E-08

19.9063 4.918E-09 1.165E-08 1.221E-08

20.1250 5.162E-09 1.164E-08 1.249E-08

20.3438 5.417E-09 1.157E-08 1.304E-08

20.5625 5.431E-09 1.148E-08 1.361E-08

20.7813 5.184E-09 1.149E-08 1.388E-08

21.0000 4.852E-09 1.162E-08 1.384E-08

21.2188 4.538E-09 1.168E-08 1.377E-08

21.4375 4.170E-09 1.138E-08 1.395E-08

21.6563 3.677E-09 1.073E-08 1.434E-08

21.8750 3.184E-09 1.003E-08 1.468E-08

22.0938 2.959E-09 9.626E-09 1.467E-08

22.3125 3.172E-09 9.523E-09 1.424E-08

22.5313 3.750E-09 9.536E-09 1.371E-08

22.7500 4.446E-09 9.546E-09 1.363E-08

22.9688 5.009E-09 9.547E-09 1.427E-08

23.1875 5.297E-09 9.536E-09 1.527E-08

23.4063 5.312E-09 9.526E-09 1.588E-08

23.6250 5.190E-09 9.660E-09 1.562E-08

23.8438 5.108E-09 1.012E-08 1.464E-08

24.0625 5.153E-09 1.086E-08 1.353E-08

24.2813 5.250E-09 1.149E-08 1.273E-08

24.5000 5.258E-09 1.158E-08 1.241E-08

24.7188 5.136E-09 1.109E-08 1.248E-08

24.9375 4.997E-09 1.041E-08 1.267E-08

25.1563 4.976E-09 9.976E-09 1.258E-08

25.3750 5.069E-09 9.875E-09 1.197E-08

25.5938 5.109E-09 9.860E-09 1.102E-08

25.8125 4.915E-09 9.668E-09 1.019E-08

26.0313 4.466E-09 9.214E-09 9.792E-09

26.2500 3.920E-09 8.544E-09 9.755E-09

26.4688 3.489E-09 7.753E-09 9.889E-09

26.6875 3.292E-09 7.006E-09 1.007E-08

26.9063 3.333E-09 6.490E-09 1.021E-08

27.1250 3.552E-09 6.256E-09 1.021E-08

27.3438 3.874E-09 6.121E-09 1.006E-08

27.5625 4.199E-09 5.839E-09 9.968E-09

27.7813 4.397E-09 5.359E-09 1.012E-08

28.0000 4.344E-09 4.889E-09 1.052E-08

28.2188 4.015E-09 4.695E-09 1.092E-08

28.4375 3.530E-09 4.874E-09 1.099E-08

28.6563 3.081E-09 5.308E-09 1.057E-08

28.8750 2.770E-09 5.770E-09 9.717E-09

29.0938 2.564E-09 6.060E-09 8.787E-09

29.3125 2.406E-09 6.112E-09 8.163E-09

29.5313 2.322E-09 6.034E-09 7.969E-09

29.7500 2.365E-09 6.020E-09 7.976E-09

29.9688 2.500E-09 6.188E-09 7.908E-09

30.1875 2.620E-09 6.506E-09 7.766E-09

30.4063 2.674E-09 6.872E-09 7.757E-09

30.6250 2.720E-09 7.190E-09 7.923E-09

30.8438 2.851E-09 7.378E-09 7.997E-09

31.0625 3.082E-09 7.407E-09 7.750E-09

31.2813 3.312E-09 7.381E-09 7.354E-09

31.5000 3.387E-09 7.480E-09 7.234E-09

31.7188 3.223E-09 7.718E-09 7.585E-09

31.9375 2.895E-09 7.858E-09 8.188E-09

32.1563 2.574E-09 7.656E-09 8.682E-09

32.3750 2.373E-09 7.149E-09 8.893E-09

32.5938 2.283E-09 6.616E-09 8.860E-09

32.8125 2.260E-09 6.258E-09 8.683E-09

33.0313 2.299E-09 6.054E-09 8.449E-09

33.2500 2.413E-09 5.913E-09 8.276E-09

33.4688 2.593E-09 5.855E-09 8.307E-09

33.6875 2.838E-09 5.965E-09 8.588E-09

33.9063 3.179E-09 6.238E-09 8.955E-09

34.1250 3.596E-09 6.557E-09 9.130E-09

34.3438 3.953E-09 6.774E-09 8.978E-09

34.5625 4.076E-09 6.787E-09 8.638E-09

34.7813 3.898E-09 6.570E-09 8.338E-09

35.0000 3.503E-09 6.222E-09 8.132E-09

35.2188 3.027E-09 5.960E-09 7.907E-09

35.4375 2.587E-09 5.958E-09 7.594E-09

35.6563 2.279E-09 6.173E-09 7.276E-09

35.8750 2.183E-09 6.390E-09 7.084E-09

36.0938 2.323E-09 6.473E-09 7.058E-09

36.3125 2.633E-09 6.509E-09 7.127E-09

36.5313 2.992E-09 6.649E-09 7.170E-09

36.7500 3.278E-09 6.894E-09 7.080E-09

36.9688 3.396E-09 7.105E-09 6.815E-09

37.1875 3.304E-09 7.197E-09 6.418E-09

37.4063 3.048E-09 7.212E-09 6.048E-09

37.6250 2.749E-09 7.207E-09 5.918E-09

37.8438 2.530E-09 7.148E-09 6.138E-09

38.0625 2.447E-09 6.957E-09 6.586E-09

38.2813 2.479E-09 6.631E-09 6.997E-09

38.5000 2.553E-09 6.261E-09 7.203E-09

38.7188 2.568E-09 5.939E-09 7.243E-09

38.9375 2.451E-09 5.678E-09 7.235E-09

39.1563 2.207E-09 5.444E-09 7.218E-09

39.3750 1.933E-09 5.252E-09 7.142E-09

39.5938 1.769E-09 5.197E-09 6.973E-09

39.8125 1.793E-09 5.340E-09 6.767E-09

40.0313 1.950E-09 5.561E-09 6.639E-09

40.2500 2.067E-09 5.604E-09 6.677E-09

40.4688 2.008E-09 5.327E-09 6.853E-09

40.6875 1.820E-09 4.893E-09 7.031E-09

40.9063 1.710E-09 4.659E-09 7.061E-09

41.1250 1.838E-09 4.846E-09 6.911E-09

41.3438 2.158E-09 5.334E-09 6.711E-09

41.5625 2.463E-09 5.787E-09 6.625E-09

41.7813 2.577E-09 5.917E-09 6.691E-09

42.0000 2.486E-09 5.647E-09 6.808E-09

42.2188 2.304E-09 5.109E-09 6.897E-09

42.4375 2.156E-09 4.557E-09 6.978E-09

42.6563 2.108E-09 4.229E-09 7.064E-09

42.8750 2.163E-09 4.204E-09 7.074E-09

43.0938 2.276E-09 4.375E-09 6.981E-09

43.3125 2.354E-09 4.584E-09 6.934E-09

43.5313 2.298E-09 4.762E-09 7.097E-09

43.7500 2.167E-09 4.957E-09 7.421E-09

43.9688 2.094E-09 5.102E-09 7.533E-09

44.1875 1.546E-09 4.381E-09 6.420E-09

44.4063 -1.242E-09 8.599E-10 2.212E-09

44.6250 -7.867E-09 -7.119E-09 -6.479E-09

44.8438 -1.672E-08 -1.797E-08 -1.773E-08

45.0625 -2.229E-08 -2.592E-08 -2.561E-08

45.2813 -1.951E-08 -2.515E-08 -2.442E-08

45.5000 -9.250E-09 -1.554E-08 -1.443E-08

45.7188 1.873E-09 -3.301E-09 -2.039E-09

45.9375 7.693E-09 4.870E-09 6.173E-09

46.1563 7.528E-09 7.226E-09 8.692E-09

46.3750 4.914E-09 6.513E-09 8.255E-09

46.5938 3.036E-09 5.727E-09 7.595E-09

46.8125 2.503E-09 5.696E-09 7.330E-09

47.0313 2.503E-09 5.922E-09 7.048E-09

47.2500 2.447E-09 6.038E-09 6.719E-09

47.4688 2.292E-09 6.047E-09 6.660E-09

47.6875 2.146E-09 5.907E-09 6.914E-09

47.9063 2.038E-09 5.471E-09 7.185E-09

48.1250 1.934E-09 4.772E-09 7.191E-09

48.3438 1.785E-09 4.139E-09 6.870E-09

48.5625 1.564E-09 3.951E-09 6.323E-09

48.7813 1.301E-09 4.267E-09 5.711E-09

49.0000 1.077E-09 4.754E-09 5.188E-09

49.2188 9.572E-10 5.004E-09 4.850E-09

49.4375 9.355E-10 4.898E-09 4.724E-09

49.6563 9.411E-10 4.636E-09 4.777E-09

49.8750 8.972E-10 4.480E-09 4.951E-09

50.0938 7.741E-10 4.516E-09 5.172E-09

50.3125 6.165E-10 4.643E-09 5.376E-09

50.5313 5.478E-10 4.706E-09 5.526E-09

50.7500 7.057E-10 4.624E-09 5.603E-09

50.9688 1.106E-09 4.436E-09 5.605E-09

51.1875 1.572E-09 4.258E-09 5.591E-09

51.4063 1.861E-09 4.198E-09 5.671E-09

51.6250 1.877E-09 4.274E-09 5.896E-09

51.8438 1.742E-09 4.424E-09 6.165E-09

52.0625 1.667E-09 4.565E-09 6.292E-09

52.2813 1.772E-09 4.656E-09 6.188E-09

52.5000 2.011E-09 4.722E-09 5.940E-09

52.7188 2.220E-09 4.817E-09 5.714E-09

52.9375 2.239E-09 4.971E-09 5.576E-09

53.1563 2.030E-09 5.136E-09 5.423E-09

53.3750 1.694E-09 5.197E-09 5.084E-09

53.5938 1.394E-09 5.034E-09 4.509E-09

53.8125 1.240E-09 4.598E-09 3.886E-09

54.0313 1.257E-09 4.000E-09 3.538E-09

54.2500 1.407E-09 3.537E-09 3.651E-09

54.4688 1.608E-09 3.529E-09 4.093E-09

54.6875 1.726E-09 4.046E-09 4.547E-09

54.9063 1.654E-09 4.790E-09 4.825E-09

55.1250 1.426E-09 5.309E-09 4.989E-09

55.3438 1.224E-09 5.344E-09 5.195E-09

55.5625 1.216E-09 4.973E-09 5.492E-09

55.7813 1.401E-09 4.468E-09 5.776E-09

56.0000 1.647E-09 4.086E-09 5.891E-09

56.2188 1.844E-09 3.975E-09 5.785E-09

56.4375 1.963E-09 4.170E-09 5.609E-09

56.6563 1.997E-09 4.603E-09 5.599E-09

56.8750 1.914E-09 5.116E-09 5.769E-09

57.0938 1.702E-09 5.501E-09 5.855E-09

57.3125 1.439E-09 5.570E-09 5.676E-09

57.5313 1.257E-09 5.225E-09 5.368E-09

57.7500 1.228E-09 4.497E-09 5.211E-09

57.9688 1.336E-09 3.591E-09 5.493E-09

58.1875 1.593E-09 2.904E-09 6.046E-09

58.4063 1.891E-09 2.593E-09 5.667E-09

58.6250 1.936E-09 2.171E-09 5.302E-09

58.8438 2.950E-09 2.266E-09 1.614E-08

59.0625 1.078E-08 8.737E-09 5.966E-08

59.2813 3.462E-08 3.265E-08 1.452E-07

59.5000 7.702E-08 7.958E-08 2.424E-07

59.7188 1.235E-07 1.359E-07 2.901E-07

59.9375 1.479E-07 1.716E-07 2.497E-07

60.1563 1.340E-07 1.638E-07 1.464E-07

60.3750 9.106E-08 1.185E-07 4.613E-08

60.5938 4.478E-08 6.377E-08 -5.433E-09

60.8125 1.495E-08 2.512E-08 -1.176E-08

61.0313 3.500E-09 8.092E-09 -1.864E-09

61.2500 1.642E-09 4.033E-09 4.666E-09

61.4688 1.815E-09 3.841E-09 5.800E-09

61.6875 1.682E-09 3.943E-09 5.468E-09

61.9063 1.459E-09 4.011E-09 5.286E-09

62.1250 1.398E-09 4.073E-09 5.081E-09

62.3438 1.464E-09 4.026E-09 4.853E-09

62.5625 1.582E-09 3.903E-09 4.732E-09

62.7813 1.647E-09 3.822E-09 4.648E-09

63.0000 1.573E-09 3.884E-09 4.533E-09

63.2188 1.370E-09 4.124E-09 4.434E-09

63.4375 1.118E-09 4.478E-09 4.388E-09

63.6563 9.278E-10 4.837E-09 4.368E-09

63.8750 9.048E-10 5.118E-09 4.358E-09

64.0938 1.089E-09 5.246E-09 4.424E-09

64.3125 1.376E-09 5.155E-09 4.675E-09

64.5313 1.563E-09 4.882E-09 5.132E-09

64.7500 1.511E-09 4.574E-09 5.638E-09

64.9688 1.282E-09 4.321E-09 5.918E-09

65.1875 1.098E-09 4.030E-09 5.761E-09

65.4063 1.145E-09 3.555E-09 5.200E-09

65.6250 1.405E-09 2.942E-09 4.557E-09

65.8438 1.678E-09 2.473E-09 4.230E-09

66.0625 1.759E-09 2.419E-09 4.406E-09

66.2813 1.611E-09 2.740E-09 4.920E-09

66.5000 1.358E-09 3.092E-09 5.416E-09

66.7188 1.133E-09 3.150E-09 5.599E-09

66.9375 9.817E-10 2.932E-09 5.373E-09

67.1563 8.921E-10 2.781E-09 4.826E-09

67.3750 8.821E-10 3.004E-09 4.164E-09

67.5938 1.006E-09 3.555E-09 3.658E-09

67.8125 1.262E-09 4.103E-09 3.514E-09

68.0313 1.525E-09 4.384E-09 3.725E-09

68.2500 1.639E-09 4.398E-09 4.069E-09

68.4688 1.587E-09 4.278E-09 4.295E-09

68.6875 1.541E-09 4.091E-09 4.318E-09

68.9063 1.687E-09 3.838E-09 4.227E-09

69.1250 2.015E-09 3.566E-09 4.139E-09

69.3438 2.297E-09 3.358E-09 4.064E-09

69.5625 2.299E-09 3.240E-09 3.924E-09

69.7813 1.989E-09 3.199E-09 3.685E-09

70.0000 1.542E-09 3.271E-09 3.443E-09

70.2188 1.181E-09 3.539E-09 3.340E-09

70.4375 1.040E-09 3.989E-09 3.400E-09

70.6563 1.116E-09 4.433E-09 3.501E-09

70.8750 1.310E-09 4.624E-09 3.537E-09

71.0938 1.486E-09 4.465E-09 3.555E-09

71.3125 1.537E-09 4.075E-09 3.695E-09

71.5313 1.426E-09 3.642E-09 3.998E-09

71.7500 1.198E-09 3.254E-09 4.341E-09

71.9688 9.537E-10 2.895E-09 4.567E-09

72.1875 8.017E-10 2.566E-09 4.653E-09

72.4063 7.964E-10 2.352E-09 4.704E-09

72.6250 9.051E-10 2.342E-09 4.774E-09

72.8438 1.048E-09 2.533E-09 4.725E-09

73.0625 1.182E-09 2.839E-09 4.352E-09

73.2813 1.322E-09 3.164E-09 3.672E-09

73.5000 1.484E-09 3.468E-09 3.010E-09

73.7188 1.630E-09 3.749E-09 2.727E-09

73.9375 1.706E-09 3.983E-09 2.888E-09

74.1563 1.706E-09 4.103E-09 3.242E-09

74.3750 1.669E-09 4.053E-09 3.494E-09

74.5938 1.617E-09 3.869E-09 3.530E-09

74.8125 1.532E-09 3.659E-09 3.416E-09

75.0313 1.403E-09 3.470E-09 3.286E-09

75.2500 1.275E-09 3.229E-09 3.264E-09

75.4688 1.214E-09 2.864E-09 3.396E-09

75.6875 1.236E-09 2.477E-09 3.606E-09

75.9063 1.289E-09 2.298E-09 3.745E-09

76.1250 1.322E-09 2.450E-09 3.747E-09

76.3438 1.346E-09 2.801E-09 3.730E-09

76.5625 1.400E-09 3.099E-09 3.853E-09

76.7813 1.482E-09 3.221E-09 4.108E-09

77.0000 1.530E-09 3.252E-09 4.308E-09

77.2188 1.508E-09 3.332E-09 4.299E-09

77.4375 1.474E-09 3.492E-09 4.085E-09

77.6563 1.547E-09 3.634E-09 3.747E-09

77.8750 1.780E-09 3.625E-09 3.336E-09

78.0938 2.092E-09 3.393E-09 2.911E-09

78.3125 2.316E-09 2.992E-09 2.588E-09

78.5313 2.304E-09 2.599E-09 2.482E-09

78.7500 2.036E-09 2.420E-09 2.606E-09

78.9688 1.630E-09 2.528E-09 2.891E-09

79.1875 1.244E-09 2.800E-09 3.253E-09

79.4063 9.662E-10 3.009E-09 3.607E-09

79.6250 7.863E-10 2.998E-09 3.865E-09

79.8438 6.779E-10 2.766E-09 3.979E-09

80.0625 6.606E-10 2.425E-09 3.985E-09

80.2813 7.658E-10 2.109E-09 3.947E-09

80.5000 9.640E-10 1.913E-09 3.861E-09

80.7188 1.162E-09 1.888E-09 3.664E-09

80.9375 1.281E-09 2.024E-09 3.373E-09

81.1563 1.322E-09 2.246E-09 3.138E-09

81.3750 1.355E-09 2.438E-09 3.128E-09

81.5938 1.439E-09 2.535E-09 3.359E-09

81.8125 1.554E-09 2.584E-09 3.685E-09

82.0313 1.631E-09 2.714E-09 3.952E-09

82.2500 1.636E-09 3.010E-09 4.127E-09

82.4688 1.605E-09 3.419E-09 4.256E-09

82.6875 1.577E-09 3.761E-09 4.345E-09

82.9063 1.547E-09 3.844E-09 4.309E-09

83.1250 1.491E-09 3.603E-09 4.064E-09

83.3438 1.411E-09 3.172E-09 3.647E-09

83.5625 1.322E-09 2.787E-09 3.234E-09

83.7813 1.232E-09 2.608E-09 3.022E-09

84.0000 1.161E-09 2.625E-09 3.086E-09

84.2188 1.148E-09 2.736E-09 3.327E-09

84.4375 1.199E-09 2.861E-09 3.552E-09

84.6563 1.252E-09 2.950E-09 3.614E-09

84.8750 1.216E-09 2.934E-09 3.506E-09

85.0938 1.056E-09 2.742E-09 3.343E-09

85.3125 8.265E-10 2.403E-09 3.266E-09

85.5313 6.360E-10 2.075E-09 3.330E-09

85.7500 5.727E-10 1.943E-09 3.460E-09

85.9688 6.460E-10 2.083E-09 3.504E-09

86.1875 7.807E-10 2.417E-09 3.358E-09

86.4063 8.764E-10 2.763E-09 3.092E-09

86.6250 8.828E-10 2.934E-09 2.915E-09

86.8438 8.291E-10 2.858E-09 2.980E-09

87.0625 8.013E-10 2.634E-09 3.223E-09

87.2813 8.894E-10 2.449E-09 3.430E-09

87.5000 1.114E-09 2.379E-09 3.467E-09

87.7188 1.379E-09 2.321E-09 3.370E-09

87.9375 1.526E-09 2.142E-09 3.222E-09

88.1563 1.494E-09 1.876E-09 3.042E-09

88.3750 1.375E-09 1.710E-09 2.853E-09

88.5938 1.289E-09 1.761E-09 2.755E-09

88.8125 1.255E-09 1.971E-09 2.855E-09

89.0313 1.248E-09 2.221E-09 3.158E-09

89.2500 1.135E-09 2.299E-09 3.360E-09

89.4688 1.679E-10 1.333E-09 2.391E-09

89.6875 -3.294E-09 -2.472E-09 -1.680E-09

89.9063 -1.049E-08 -1.040E-08 -1.012E-08

90.1250 -1.983E-08 -2.063E-08 -2.092E-08

90.3438 -2.627E-08 -2.783E-08 -2.843E-08

90.5625 -2.470E-08 -2.677E-08 -2.735E-08

90.7813 -1.464E-08 -1.722E-08 -1.772E-08

91.0000 -1.326E-09 -4.540E-09 -5.295E-09

91.2188 8.420E-09 4.816E-09 3.526E-09

91.4375 1.133E-08 8.163E-09 6.551E-09

91.6563 8.981E-09 7.249E-09 5.875E-09

91.8750 5.096E-09 5.291E-09 4.462E-09

92.0938 2.316E-09 4.189E-09 3.617E-09

92.3125 1.190E-09 4.051E-09 3.169E-09

92.5313 1.044E-09 4.202E-09 2.764E-09

92.7500 1.110E-09 4.126E-09 2.463E-09

92.9688 1.043E-09 3.748E-09 2.450E-09

93.1875 8.798E-10 3.277E-09 2.666E-09

93.4063 7.904E-10 2.930E-09 2.852E-09

93.6250 8.453E-10 2.748E-09 2.853E-09

93.8438 9.578E-10 2.623E-09 2.743E-09

94.0625 1.006E-09 2.451E-09 2.669E-09

94.2813 9.638E-10 2.238E-09 2.666E-09

94.5000 8.961E-10 2.071E-09 2.686E-09

94.7188 8.595E-10 2.017E-09 2.723E-09

94.9375 8.634E-10 2.069E-09 2.809E-09

95.1563 9.101E-10 2.169E-09 2.929E-09

95.3750 1.015E-09 2.266E-09 3.006E-09

95.5938 1.160E-09 2.348E-09 3.011E-09

95.8125 1.261E-09 2.441E-09 3.010E-09

96.0313 1.238E-09 2.565E-09 3.090E-09

96.2500 1.094E-09 2.696E-09 3.253E-09

96.4688 9.066E-10 2.785E-09 3.417E-09

96.6875 7.472E-10 2.780E-09 3.491E-09

96.9063 6.351E-10 2.639E-09 3.422E-09

97.1250 5.674E-10 2.336E-09 3.231E-09

97.3438 5.460E-10 1.915E-09 3.023E-09

97.5625 5.659E-10 1.503E-09 2.952E-09

97.7813 6.094E-10 1.236E-09 3.085E-09

98.0000 6.700E-10 1.166E-09 3.335E-09

98.2188 7.591E-10 1.265E-09 3.543E-09

98.4375 8.718E-10 1.495E-09 3.613E-09

98.6563 9.601E-10 1.797E-09 3.530E-09

98.8750 9.648E-10 2.057E-09 3.275E-09

99.0938 8.704E-10 2.177E-09 2.838E-09

99.3125 7.213E-10 2.191E-09 2.294E-09

99.5313 5.898E-10 2.218E-09 1.828E-09

99.7500 5.359E-10 2.300E-09 1.632E-09

99.9688 5.812E-10 2.358E-09 1.775E-09

100.1875 6.910E-10 2.340E-09 2.155E-09

100.4063 7.742E-10 2.338E-09 2.544E-09

100.6250 7.330E-10 2.478E-09 2.726E-09

100.8438 5.535E-10 2.739E-09 2.639E-09

101.0625 3.471E-10 2.930E-09 2.408E-09

101.2813 2.649E-10 2.876E-09 2.210E-09

101.5000 3.510E-10 2.583E-09 2.103E-09

101.7188 5.025E-10 2.230E-09 2.015E-09

101.9375 5.924E-10 2.012E-09 1.896E-09

102.1563 6.026E-10 2.000E-09 1.811E-09

102.3750 6.142E-10 2.105E-09 1.886E-09

102.5938 6.951E-10 2.166E-09 2.161E-09

102.8125 8.325E-10 2.073E-09 2.555E-09

103.0313 9.651E-10 1.830E-09 2.930E-09

103.2500 1.042E-09 1.531E-09 3.187E-09

103.4688 1.049E-09 1.294E-09 3.301E-09

103.6875 9.971E-10 1.202E-09 3.314E-09

103.9063 9.169E-10 1.268E-09 3.312E-09

104.1250 8.584E-10 1.456E-09 3.394E-09

104.3438 8.856E-10 1.773E-09 3.651E-09

104.5625 1.036E-09 2.330E-09 4.140E-09

104.7813 1.288E-09 3.226E-09 4.820E-09

105.0000 1.589E-09 4.317E-09 5.482E-09

105.2188 1.895E-09 5.190E-09 5.814E-09

105.4375 2.144E-09 5.446E-09 5.628E-09

105.6563 2.231E-09 5.012E-09 5.028E-09

105.8750 2.061E-09 4.153E-09 4.283E-09

106.0938 1.666E-09 3.194E-09 3.552E-09

106.3125 1.193E-09 2.304E-09 2.808E-09

106.5313 7.713E-10 1.527E-09 2.023E-09

106.7500 4.408E-10 9.332E-10 1.324E-09

106.9688 2.234E-10 6.436E-10 9.012E-10

107.1875 1.729E-10 7.146E-10 8.281E-10

107.4063 2.937E-10 1.058E-09 1.027E-09

107.6250 4.713E-10 1.488E-09 1.360E-09

107.8438 5.610E-10 1.810E-09 1.704E-09

108.0625 5.317E-10 1.886E-09 1.964E-09

108.2813 4.678E-10 1.694E-09 2.106E-09

108.5000 4.438E-10 1.385E-09 2.163E-09

108.7188 4.408E-10 1.211E-09 2.193E-09

108.9375 3.880E-10 1.331E-09 2.237E-09

109.1563 2.444E-10 1.672E-09 2.313E-09

109.3750 3.378E-11 2.007E-09 2.430E-09

109.5938 -1.738E-10 2.184E-09 2.566E-09

109.8125 -3.033E-10 2.251E-09 2.662E-09

110.0313 -3.145E-10 2.360E-09 2.669E-09

110.2500 -2.202E-10 2.549E-09 2.609E-09

110.4688 -7.387E-11 2.675E-09 2.559E-09

110.6875 6.330E-11 2.577E-09 2.543E-09

110.9063 1.497E-10 2.288E-09 2.492E-09

111.1250 1.734E-10 2.013E-09 2.324E-09

111.3438 1.526E-10 1.906E-09 2.063E-09

111.5625 1.316E-10 1.928E-09 1.835E-09

111.7813 1.588E-10 1.951E-09 1.742E-09

112.0000 2.489E-10 1.937E-09 1.770E-09

112.2188 3.618E-10 1.939E-09 1.834E-09

112.4375 4.366E-10 1.981E-09 1.873E-09

112.6563 4.502E-10 2.022E-09 1.878E-09

112.8750 4.311E-10 2.027E-09 1.857E-09

113.0938 4.190E-10 2.000E-09 1.819E-09

113.3125 4.384E-10 1.945E-09 1.804E-09

113.5313 5.155E-10 1.852E-09 1.880E-09

113.7500 6.832E-10 1.731E-09 2.087E-09

113.9688 9.337E-10 1.626E-09 2.386E-09

114.1875 1.180E-09 1.578E-09 2.679E-09

114.4063 1.303E-09 1.594E-09 2.873E-09

114.6250 1.248E-09 1.654E-09 2.918E-09

114.8438 1.064E-09 1.734E-09 2.793E-09

115.0625 8.445E-10 1.815E-09 2.504E-09

115.2813 6.531E-10 1.895E-09 2.093E-09

115.5000 5.058E-10 2.007E-09 1.635E-09

115.7188 4.074E-10 2.187E-09 1.210E-09

115.9375 3.639E-10 2.410E-09 8.862E-10

116.1563 3.613E-10 2.567E-09 7.093E-10

116.3750 3.654E-10 2.551E-09 7.106E-10

116.5938 3.577E-10 2.369E-09 9.020E-10

116.8125 3.424E-10 2.138E-09 1.236E-09

117.0313 3.056E-10 1.954E-09 1.616E-09

117.2500 2.094E-10 1.812E-09 1.947E-09

117.4688 5.196E-11 1.678E-09 2.114E-09

117.6875 -1.044E-10 1.585E-09 2.063E-09

117.9063 -1.829E-10 1.608E-09 1.999E-09

118.1250 -1.485E-10 1.783E-09 1.969E-09

118.3438 -4.693E-11 2.021E-09 1.577E-09

118.5625 5.057E-11 2.046E-09 3.121E-09

118.7813 4.358E-10 1.550E-09 1.625E-08

119.0000 2.182E-09 8.272E-10 5.224E-08

119.2188 6.425E-09 1.288E-09 1.041E-07

119.4375 1.236E-08 4.585E-09 1.362E-07

119.6563 1.643E-08 1.050E-08 1.107E-07

119.8750 1.512E-08 1.608E-08 3.074E-08

120.0938 8.837E-09 1.777E-08 -5.307E-08

120.3125 2.004E-09 1.472E-08 -8.945E-08

120.5313 -1.319E-09 9.351E-09 -7.218E-08

120.7500 -1.002E-09 4.820E-09 -3.434E-08

120.9688 3.521E-10 2.470E-09 -7.552E-09

121.1875 8.472E-10 1.737E-09 1.635E-09

121.4063 5.623E-10 1.636E-09 2.284E-09

121.6250 2.537E-10 1.701E-09 1.877E-09

121.8438 1.681E-10 1.843E-09 1.913E-09

122.0625 1.766E-10 1.980E-09 1.922E-09

122.2813 2.405E-10 1.975E-09 1.875E-09

122.5000 4.092E-10 1.763E-09 1.910E-09

122.7188 6.476E-10 1.419E-09 2.066E-09

122.9375 8.503E-10 1.108E-09 2.358E-09

123.1563 9.450E-10 9.578E-10 2.695E-09

123.3750 9.365E-10 9.505E-10 2.892E-09

123.5938 8.940E-10 9.649E-10 2.867E-09

123.8125 8.980E-10 9.094E-10 2.717E-09

124.0313 9.651E-10 8.056E-10 2.573E-09

124.2500 1.044E-09 7.469E-10 2.451E-09

124.4688 1.101E-09 8.080E-10 2.271E-09

124.6875 1.180E-09 9.979E-10 2.016E-09

124.9063 1.331E-09 1.271E-09 1.803E-09

125.1250 1.516E-09 1.552E-09 1.792E-09

125.3438 1.619E-09 1.755E-09 2.008E-09

125.5625 1.542E-09 1.816E-09 2.301E-09

125.7813 1.290E-09 1.733E-09 2.495E-09

126.0000 9.809E-10 1.573E-09 2.550E-09

126.2188 7.760E-10 1.415E-09 2.559E-09

126.4375 7.666E-10 1.314E-09 2.594E-09

126.6563 8.989E-10 1.284E-09 2.600E-09

126.8750 1.024E-09 1.316E-09 2.469E-09

127.0938 1.036E-09 1.385E-09 2.184E-09

127.3125 9.556E-10 1.477E-09 1.854E-09

127.5313 8.653E-10 1.607E-09 1.629E-09

127.7500 8.053E-10 1.793E-09 1.610E-09

127.9688 7.403E-10 2.000E-09 1.793E-09

128.1875 6.292E-10 2.148E-09 2.077E-09

128.4063 4.963E-10 2.188E-09 2.296E-09

128.6250 4.173E-10 2.141E-09 2.326E-09

128.8438 4.355E-10 2.053E-09 2.190E-09

129.0625 5.092E-10 1.936E-09 2.043E-09

129.2813 5.593E-10 1.771E-09 2.014E-09

129.5000 5.679E-10 1.566E-09 2.067E-09

129.7188 6.026E-10 1.386E-09 2.054E-09

129.9375 7.213E-10 1.310E-09 1.885E-09

130.1563 8.665E-10 1.349E-09 1.623E-09

130.3750 8.941E-10 1.439E-09 1.410E-09

130.5938 7.213E-10 1.522E-09 1.327E-09

130.8125 4.267E-10 1.602E-09 1.348E-09

131.0313 1.894E-10 1.704E-09 1.397E-09

131.2500 1.369E-10 1.801E-09 1.423E-09

131.4688 2.574E-10 1.839E-09 1.425E-09

131.6875 4.445E-10 1.836E-09 1.445E-09

131.9063 6.057E-10 1.875E-09 1.540E-09

132.1250 7.254E-10 1.994E-09 1.740E-09

132.3438 8.382E-10 2.116E-09 1.995E-09

132.5625 9.632E-10 2.127E-09 2.188E-09

132.7813 1.077E-09 1.988E-09 2.244E-09

133.0000 1.142E-09 1.765E-09 2.204E-09

133.2188 1.134E-09 1.542E-09 2.173E-09

133.4375 1.055E-09 1.362E-09 2.205E-09

133.6563 9.271E-10 1.229E-09 2.269E-09

133.8750 8.033E-10 1.151E-09 2.309E-09

134.0938 7.583E-10 1.154E-09 2.322E-09

134.3125 8.187E-10 1.237E-09 2.335E-09

134.5313 8.093E-10 1.226E-09 2.218E-09

134.7500 2.086E-10 5.936E-10 1.443E-09

134.9688 -1.672E-09 -1.386E-09 -7.692E-10

135.1875 -4.997E-09 -4.901E-09 -4.633E-09

135.4063 -8.728E-09 -8.854E-09 -8.955E-09

135.6250 -1.094E-08 -1.117E-08 -1.145E-08

135.8438 -1.020E-08 -1.032E-08 -1.042E-08

136.0625 -6.667E-09 -6.525E-09 -6.208E-09

136.2813 -1.843E-09 -1.554E-09 -9.406E-10

136.5000 2.563E-09 2.700E-09 3.088E-09

136.7188 5.430E-09 5.208E-09 4.855E-09

136.9375 6.307E-09 5.872E-09 4.767E-09

137.1563 5.368E-09 5.155E-09 3.887E-09

137.3750 3.406E-09 3.784E-09 3.089E-09

137.5938 1.489E-09 2.473E-09 2.675E-09

137.8125 3.425E-10 1.636E-09 2.503E-09

138.0313 2.421E-11 1.304E-09 2.334E-09

138.2500 1.779E-10 1.285E-09 2.047E-09

138.4688 4.424E-10 1.374E-09 1.651E-09

138.6875 6.272E-10 1.449E-09 1.242E-09

138.9063 6.804E-10 1.475E-09 9.692E-10

139.1250 6.301E-10 1.469E-09 9.591E-10

139.3438 5.398E-10 1.455E-09 1.200E-09

139.5625 4.533E-10 1.431E-09 1.514E-09

139.7813 3.664E-10 1.369E-09 1.676E-09

140.0000 2.607E-10 1.258E-09 1.589E-09

140.2188 1.560E-10 1.132E-09 1.355E-09

140.4375 1.127E-10 1.053E-09 1.160E-09

140.6563 1.772E-10 1.058E-09 1.101E-09

140.8750 3.347E-10 1.131E-09 1.128E-09

141.0938 5.204E-10 1.223E-09 1.147E-09

141.3125 6.707E-10 1.292E-09 1.149E-09

141.5313 7.579E-10 1.320E-09 1.192E-09

141.7500 7.880E-10 1.295E-09 1.295E-09

141.9688 7.838E-10 1.209E-09 1.385E-09

142.1875 7.655E-10 1.068E-09 1.375E-09

142.4063 7.372E-10 9.224E-10 1.269E-09

142.6250 6.906E-10 8.462E-10 1.172E-09

142.8438 6.308E-10 8.830E-10 1.204E-09

143.0625 5.852E-10 1.002E-09 1.409E-09

143.2813 5.723E-10 1.119E-09 1.730E-09

143.5000 5.727E-10 1.168E-09 2.037E-09

143.7188 5.526E-10 1.147E-09 2.192E-09

143.9375 5.121E-10 1.110E-09 2.110E-09

144.1563 4.901E-10 1.116E-09 1.815E-09

144.3750 5.230E-10 1.174E-09 1.440E-09

144.5938 6.162E-10 1.240E-09 1.149E-09

144.8125 7.529E-10 1.270E-09 1.037E-09

145.0313 9.030E-10 1.272E-09 1.095E-09

145.2500 1.016E-09 1.287E-09 1.256E-09

145.4688 1.035E-09 1.327E-09 1.458E-09

145.6875 9.354E-10 1.350E-09 1.662E-09

145.9063 7.519E-10 1.321E-09 1.847E-09

146.1250 5.467E-10 1.270E-09 2.005E-09

146.3438 3.751E-10 1.287E-09 2.124E-09

146.5625 2.864E-10 1.425E-09 2.174E-09

146.7813 3.299E-10 1.635E-09 2.112E-09

147.0000 5.128E-10 1.784E-09 1.934E-09

147.2188 7.491E-10 1.759E-09 1.695E-09

147.4375 8.885E-10 1.563E-09 1.458E-09

147.6563 8.277E-10 1.312E-09 1.248E-09

147.8750 5.938E-10 1.143E-09 1.087E-09

148.0938 3.121E-10 1.125E-09 1.036E-09

148.3125 1.037E-10 1.245E-09 1.153E-09

148.5313 2.243E-11 1.434E-09 1.383E-09

148.7500 6.704E-11 1.589E-09 1.563E-09

148.9688 2.124E-10 1.630E-09 1.562E-09

149.1875 4.138E-10 1.577E-09 1.410E-09

149.4063 6.072E-10 1.572E-09 1.244E-09

149.6250 7.354E-10 1.736E-09 1.142E-09

149.8438 7.795E-10 2.015E-09 1.068E-09

150.0625 7.634E-10 2.204E-09 9.730E-10

150.2813 7.294E-10 2.149E-09 9.128E-10

150.5000 7.136E-10 1.883E-09 9.968E-10

150.7188 7.330E-10 1.567E-09 1.237E-09

150.9375 7.789E-10 1.334E-09 1.485E-09

151.1563 8.188E-10 1.217E-09 1.558E-09

151.3750 8.153E-10 1.184E-09 1.408E-09

151.5938 7.654E-10 1.188E-09 1.170E-09

151.8125 7.215E-10 1.199E-09 1.038E-09

152.0313 7.473E-10 1.193E-09 1.107E-09

152.2500 8.329E-10 1.150E-09 1.299E-09

152.4688 8.764E-10 1.049E-09 1.445E-09

152.6875 7.840E-10 8.765E-10 1.430E-09

152.9063 5.848E-10 6.592E-10 1.280E-09

153.1250 4.133E-10 4.844E-10 1.104E-09

153.3438 3.696E-10 4.579E-10 9.896E-10

153.5625 4.266E-10 6.121E-10 9.478E-10

153.7813 4.882E-10 8.620E-10 9.470E-10

154.0000 5.098E-10 1.072E-09 9.762E-10

154.2188 5.303E-10 1.160E-09 1.068E-09

154.4375 6.004E-10 1.130E-09 1.262E-09

154.6563 7.086E-10 1.022E-09 1.556E-09

154.8750 7.851E-10 8.812E-10 1.887E-09

155.0938 7.663E-10 7.560E-10 2.154E-09

155.3125 6.538E-10 7.020E-10 2.238E-09

155.5313 5.091E-10 7.467E-10 2.054E-09

155.7500 3.917E-10 8.598E-10 1.637E-09

155.9688 3.095E-10 9.675E-10 1.179E-09

156.1875 2.382E-10 1.006E-09 9.223E-10

156.4063 1.696E-10 9.599E-10 9.695E-10

156.6250 1.160E-10 8.620E-10 1.207E-09

156.8438 7.655E-11 7.591E-10 1.433E-09

157.0625 3.009E-11 6.921E-10 1.536E-09

157.2813 -2.481E-11 6.931E-10 1.520E-09

157.5000 -3.633E-11 7.803E-10 1.425E-09

157.7188 7.751E-11 9.444E-10 1.285E-09

157.9375 3.526E-10 1.147E-09 1.158E-09

158.1563 7.129E-10 1.339E-09 1.120E-09

158.3750 9.986E-10 1.467E-09 1.204E-09

158.5938 1.092E-09 1.478E-09 1.367E-09

158.8125 1.014E-09 1.344E-09 1.531E-09

159.0313 8.754E-10 1.105E-09 1.635E-09

159.2500 7.462E-10 8.723E-10 1.627E-09

159.4688 6.220E-10 7.536E-10 1.467E-09

159.6875 5.026E-10 7.889E-10 1.179E-09

159.9063 4.359E-10 9.437E-10 8.849E-10

160.1250 4.426E-10 1.157E-09 7.360E-10

160.3438 4.461E-10 1.378E-09 7.841E-10

160.5625 3.422E-10 1.573E-09 9.487E-10

160.7813 1.335E-10 1.707E-09 1.102E-09

161.0000 -5.333E-11 1.730E-09 1.167E-09

161.2188 -9.411E-11 1.610E-09 1.144E-09

161.4375 1.680E-11 1.383E-09 1.071E-09

161.6563 1.846E-10 1.152E-09 9.943E-10

161.8750 3.173E-10 1.013E-09 9.592E-10

162.0938 3.945E-10 9.786E-10 1.007E-09

162.3125 4.551E-10 9.927E-10 1.150E-09

162.5313 5.479E-10 1.002E-09 1.350E-09

162.7500 6.915E-10 1.004E-09 1.533E-09

162.9688 8.534E-10 1.057E-09 1.652E-09

163.1875 9.550E-10 1.240E-09 1.725E-09

163.4063 9.178E-10 1.562E-09 1.790E-09

163.6250 7.416E-10 1.915E-09 1.847E-09

163.8438 5.387E-10 2.142E-09 1.863E-09

164.0625 4.586E-10 2.164E-09 1.824E-09

164.2813 5.550E-10 2.019E-09 1.749E-09

164.5000 7.394E-10 1.784E-09 1.665E-09

164.7188 8.730E-10 1.507E-09 1.586E-09

164.9375 8.889E-10 1.218E-09 1.511E-09

165.1563 8.174E-10 9.667E-10 1.438E-09

165.3750 7.245E-10 8.217E-10 1.368E-09

165.5938 6.611E-10 8.332E-10 1.319E-09

165.8125 6.563E-10 9.881E-10 1.301E-09

166.0313 7.134E-10 1.207E-09 1.295E-09

166.2500 7.942E-10 1.399E-09 1.269E-09

166.4688 8.301E-10 1.528E-09 1.213E-09

166.6875 7.761E-10 1.615E-09 1.133E-09

166.9063 6.566E-10 1.679E-09 1.031E-09

167.1250 5.525E-10 1.697E-09 9.116E-10

167.3438 5.371E-10 1.625E-09 8.324E-10

167.5625 6.167E-10 1.454E-09 8.725E-10

167.7813 7.265E-10 1.233E-09 1.027E-09

168.0000 7.828E-10 1.034E-09 1.162E-09

168.2188 7.425E-10 8.790E-10 1.146E-09

168.4375 6.210E-10 7.446E-10 1.009E-09

168.6563 4.729E-10 6.156E-10 9.171E-10

168.8750 3.678E-10 5.326E-10 9.816E-10

169.0938 3.630E-10 5.602E-10 1.150E-09

169.3125 4.545E-10 7.171E-10 1.283E-09

169.5313 5.497E-10 9.494E-10 1.294E-09

169.7500 5.278E-10 1.170E-09 1.202E-09

169.9688 3.586E-10 1.316E-09 1.109E-09

170.1875 1.450E-10 1.379E-09 1.138E-09

170.4063 2.458E-11 1.386E-09 1.347E-09

170.6250 3.840E-11 1.372E-09 1.659E-09

170.8438 1.120E-10 1.346E-09 1.894E-09

171.0625 1.518E-10 1.292E-09 1.911E-09

171.2813 1.371E-10 1.189E-09 1.724E-09

171.5000 1.198E-10 1.050E-09 1.467E-09

171.7188 1.517E-10 9.444E-10 1.268E-09

171.9375 2.186E-10 9.640E-10 1.157E-09

172.1563 2.529E-10 1.143E-09 1.084E-09

172.3750 2.129E-10 1.409E-09 1.013E-09

172.5938 1.397E-10 1.620E-09 9.708E-10

172.8125 1.093E-10 1.674E-09 9.909E-10

173.0313 1.385E-10 1.579E-09 1.049E-09

173.2500 1.706E-10 1.431E-09 1.071E-09

173.4688 1.597E-10 1.331E-09 1.038E-09

173.6875 1.357E-10 1.322E-09 1.043E-09

173.9063 1.657E-10 1.389E-09 1.195E-09

174.1250 2.677E-10 1.477E-09 1.473E-09

174.3438 3.842E-10 1.527E-09 1.718E-09

174.5625 4.434E-10 1.509E-09 1.771E-09

174.7813 4.364E-10 1.431E-09 1.604E-09

175.0000 4.180E-10 1.319E-09 1.322E-09

175.2188 4.343E-10 1.189E-09 1.042E-09

175.4375 4.652E-10 1.042E-09 8.019E-10

175.6563 4.518E-10 8.755E-10 6.263E-10

175.8750 3.774E-10 6.958E-10 5.788E-10

176.0938 3.079E-10 5.473E-10 6.831E-10

176.3125 3.306E-10 5.017E-10 9.241E-10

176.5313 4.664E-10 5.929E-10 1.242E-09

176.7500 6.474E-10 7.884E-10 1.455E-09

176.9688 7.663E-10 1.013E-09 1.536E-09

177.1875 7.715E-10 1.164E-09 1.636E-09

177.4063 7.031E-10 1.180E-09 1.401E-09

177.6250 5.510E-10 1.137E-09 9.924E-10

177.8438 2.822E-10 1.115E-09 2.075E-09

178.0625 2.485E-10 1.128E-09 2.468E-09

178.2813 7.159E-10 1.390E-09 1.581E-09

178.5000 6.236E-10 1.746E-09 4.851E-08

178.7188 8.663E-10 8.667E-10 2.818E-07

178.9375 1.374E-08 1.000E-09 8.178E-07

179.1563 6.294E-08 1.860E-08 1.499E-06

179.3750 1.576E-07 8.263E-08 1.833E-06

179.5938 2.612E-07 2.040E-07 1.382E-06

179.8125 3.053E-07 3.411E-07 2.739E-07

180.0313 2.512E-07 4.146E-07 -8.071E-07

180.2500 1.331E-07 3.737E-07 -1.241E-06

180.4688 2.638E-08 2.463E-07 -9.962E-07

180.6875 -2.235E-08 1.114E-07 -5.028E-07

180.9063 -2.295E-08 2.814E-08 -1.510E-07

181.1250 -9.534E-09 -6.341E-11 -2.019E-08

181.3438 -1.926E-09 -1.638E-09 -1.119E-09

181.5625 -2.732E-10 8.093E-10 -1.283E-09

181.7813 2.829E-10 1.665E-09 1.364E-09

182.0000 1.301E-09 2.447E-09 3.156E-09

182.2188 2.375E-09 3.564E-09 3.587E-09

182.4375 3.134E-09 4.142E-09 3.693E-09

182.6563 3.285E-09 3.812E-09 3.257E-09

182.8750 2.718E-09 2.924E-09 2.382E-09

183.0938 1.768E-09 2.008E-09 1.558E-09

183.3125 9.204E-10 1.386E-09 1.004E-09

183.5313 4.159E-10 1.059E-09 8.081E-10

183.7500 2.282E-10 8.789E-10 9.661E-10

183.9688 2.149E-10 7.624E-10 1.279E-09

184.1875 2.380E-10 7.252E-10 1.490E-09

184.4063 2.284E-10 7.769E-10 1.442E-09

184.6250 1.823E-10 8.556E-10 1.133E-09

184.8438 1.216E-10 8.682E-10 7.418E-10

185.0625 7.662E-11 7.966E-10 5.317E-10

185.2813 8.539E-11 7.398E-10 6.430E-10

185.5000 1.767E-10 8.087E-10 9.816E-10

185.7188 3.357E-10 9.844E-10 1.311E-09

185.9375 4.915E-10 1.119E-09 1.455E-09

186.1563 5.604E-10 1.080E-09 1.396E-09

186.3750 5.207E-10 8.750E-10 1.203E-09

186.5938 4.345E-10 6.482E-10 9.319E-10

186.8125 3.796E-10 5.660E-10 6.404E-10

187.0313 3.626E-10 6.862E-10 4.289E-10

187.2500 3.124E-10 9.021E-10 3.968E-10

187.4688 1.643E-10 1.031E-09 5.668E-10

187.6875 -5.835E-11 9.876E-10 8.609E-10

187.9063 -2.540E-10 8.591E-10 1.148E-09

188.1250 -3.279E-10 7.949E-10 1.334E-09

188.3438 -2.652E-10 8.513E-10 1.433E-09

188.5625 -1.323E-10 9.778E-10 1.529E-09

188.7813 -1.999E-11 1.118E-09 1.656E-09

189.0000 1.671E-11 1.253E-09 1.736E-09

189.2188 -1.998E-11 1.343E-09 1.668E-09

189.4375 -8.443E-11 1.295E-09 1.461E-09

189.6563 -1.138E-10 1.065E-09 1.244E-09

189.8750 -5.867E-11 7.488E-10 1.141E-09

190.0938 8.167E-11 5.254E-10 1.171E-09

190.3125 2.420E-10 4.941E-10 1.268E-09

190.5313 3.268E-10 5.980E-10 1.370E-09

190.7500 2.830E-10 7.177E-10 1.449E-09

190.9688 1.465E-10 8.035E-10 1.480E-09

191.1875 1.609E-11 8.946E-10 1.432E-09

191.4063 -2.440E-11 1.028E-09 1.311E-09

191.6250 2.917E-11 1.166E-09 1.196E-09

191.8438 1.040E-10 1.221E-09 1.183E-09

192.0625 1.194E-10 1.164E-09 1.283E-09

192.2813 6.346E-11 1.073E-09 1.404E-09

192.5000 3.394E-12 1.062E-09 1.433E-09

192.7188 1.537E-11 1.159E-09 1.328E-09

192.9375 1.033E-10 1.269E-09 1.146E-09

193.1563 1.960E-10 1.276E-09 1.012E-09

193.3750 2.275E-10 1.153E-09 1.050E-09

193.5938 2.070E-10 9.865E-10 1.296E-09

193.8125 1.919E-10 8.893E-10 1.623E-09

194.0313 1.962E-10 9.244E-10 1.812E-09

194.2500 1.628E-10 1.079E-09 1.730E-09

194.4688 4.850E-11 1.287E-09 1.448E-09

194.6875 -8.619E-11 1.461E-09 1.161E-09

194.9063 -1.182E-10 1.530E-09 1.005E-09

195.1250 4.267E-12 1.459E-09 9.759E-10

195.3438 2.096E-10 1.269E-09 1.011E-09

195.5625 3.963E-10 1.042E-09 1.091E-09

195.7813 5.341E-10 8.880E-10 1.234E-09

196.0000 6.382E-10 8.763E-10 1.431E-09

196.2188 6.827E-10 9.752E-10 1.609E-09

196.4375 6.039E-10 1.089E-09 1.685E-09

196.6563 3.929E-10 1.156E-09 1.620E-09

196.8750 1.409E-10 1.205E-09 1.448E-09

197.0938 -2.432E-11 1.303E-09 1.248E-09

197.3125 -3.369E-11 1.466E-09 1.097E-09

197.5313 8.634E-11 1.630E-09 1.027E-09

197.7500 2.402E-10 1.715E-09 1.022E-09

197.9688 3.250E-10 1.689E-09 1.050E-09

198.1875 3.029E-10 1.575E-09 1.090E-09

198.4063 2.355E-10 1.408E-09 1.126E-09

198.6250 2.347E-10 1.212E-09 1.134E-09

198.8438 3.632E-10 1.022E-09 1.079E-09

199.0625 5.826E-10 9.045E-10 9.289E-10

199.2813 7.938E-10 9.175E-10 6.812E-10

199.5000 9.092E-10 1.043E-09 3.868E-10

199.7188 8.896E-10 1.198E-09 1.468E-10

199.9375 7.498E-10 1.310E-09 6.105E-11

200.1563 5.587E-10 1.357E-09 1.571E-10

200.3750 4.140E-10 1.338E-09 3.702E-10

200.5938 3.765E-10 1.232E-09 5.979E-10

200.8125 4.205E-10 1.035E-09 7.737E-10

201.0313 4.642E-10 8.104E-10 8.866E-10

201.2500 4.500E-10 6.708E-10 9.462E-10

201.4688 3.841E-10 6.979E-10 9.505E-10

201.6875 3.105E-10 8.698E-10 8.981E-10

201.9063 2.746E-10 1.073E-09 8.124E-10

202.1250 3.068E-10 1.189E-09 7.362E-10

202.3438 4.029E-10 1.179E-09 7.066E-10

202.5625 5.061E-10 1.094E-09 7.468E-10

202.7813 5.337E-10 1.005E-09 8.658E-10

203.0000 4.517E-10 9.377E-10 1.036E-09

203.2188 3.182E-10 8.740E-10 1.177E-09

203.4375 2.397E-10 7.993E-10 1.206E-09

203.6563 2.840E-10 7.236E-10 1.118E-09

203.8750 4.364E-10 6.740E-10 1.002E-09

204.0938 6.191E-10 6.842E-10 9.548E-10

204.3125 7.335E-10 7.791E-10 9.970E-10

204.5313 7.036E-10 9.453E-10 1.062E-09

204.7500 5.215E-10 1.117E-09 1.073E-09

204.9688 2.622E-10 1.211E-09 1.013E-09

205.1875 3.625E-11 1.185E-09 9.250E-10

205.4063 -8.196E-11 1.057E-09 8.517E-10

205.6250 -7.140E-11 8.830E-10 7.973E-10

205.8438 6.376E-11 7.335E-10 7.488E-10

206.0625 2.941E-10 6.647E-10 7.170E-10

206.2813 5.359E-10 6.941E-10 7.238E-10

206.5000 6.754E-10 7.800E-10 7.543E-10

206.7188 6.570E-10 8.382E-10 7.588E-10

206.9375 5.271E-10 7.971E-10 7.162E-10

207.1563 3.768E-10 6.541E-10 6.667E-10

207.3750 2.623E-10 4.842E-10 6.615E-10

207.5938 1.929E-10 3.928E-10 6.965E-10

207.8125 1.679E-10 4.458E-10 7.193E-10

208.0313 1.978E-10 6.250E-10 6.930E-10

208.2500 2.900E-10 8.412E-10 6.354E-10

208.4688 4.165E-10 9.817E-10 5.832E-10

208.6875 5.006E-10 9.405E-10 5.184E-10

208.9063 4.902E-10 6.431E-10 3.580E-10

209.1250 4.941E-10 1.556E-10 8.047E-11

209.3438 7.726E-10 -1.811E-10 -1.072E-10

209.5625 1.441E-09 1.274E-10 1.736E-10

209.7813 2.158E-09 1.248E-09 1.087E-09

210.0000 2.291E-09 2.646E-09 2.235E-09

210.2188 1.573E-09 3.439E-09 2.893E-09

210.4375 5.792E-10 3.285E-09 2.810E-09

210.6563 3.778E-10 2.825E-09 2.688E-09

210.8750 1.538E-09 3.032E-09 3.559E-09

211.0938 3.469E-09 4.110E-09 5.525E-09

211.3125 4.838E-09 5.202E-09 7.329E-09

211.5313 4.727E-09 5.258E-09 7.457E-09

211.7500 3.350E-09 4.088E-09 5.635E-09

211.9688 1.695E-09 2.464E-09 3.065E-09

212.1875 5.862E-10 1.302E-09 1.213E-09

212.4063 1.725E-10 8.993E-10 6.024E-10

212.6250 1.575E-10 9.421E-10 7.665E-10

212.8438 2.496E-10 1.043E-09 1.006E-09

213.0625 3.337E-10 1.094E-09 9.876E-10

213.2813 3.864E-10 1.169E-09 7.834E-10

213.5000 3.986E-10 1.282E-09 6.106E-10

213.7188 3.808E-10 1.326E-09 6.184E-10

213.9375 3.569E-10 1.203E-09 8.066E-10

214.1563 3.280E-10 9.274E-10 1.056E-09

214.3750 2.821E-10 6.033E-10 1.217E-09

214.5938 2.513E-10 3.459E-10 1.211E-09

214.8125 3.092E-10 2.196E-10 1.079E-09

215.0313 4.723E-10 2.231E-10 9.621E-10

215.2500 6.354E-10 3.102E-10 9.801E-10

215.4688 6.615E-10 4.227E-10 1.116E-09

215.6875 5.362E-10 5.076E-10 1.236E-09

215.9063 3.871E-10 5.231E-10 1.233E-09

216.1250 3.375E-10 4.599E-10 1.127E-09

216.3438 3.796E-10 3.664E-10 1.009E-09

216.5625 4.150E-10 3.232E-10 9.345E-10

216.7813 3.827E-10 3.718E-10 9.125E-10

217.0000 3.082E-10 4.753E-10 9.476E-10

217.2188 2.476E-10 5.629E-10 1.028E-09

217.4375 2.283E-10 5.971E-10 1.090E-09

217.6563 2.445E-10 5.850E-10 1.061E-09

217.8750 2.709E-10 5.495E-10 9.351E-10

218.0938 2.701E-10 5.258E-10 7.969E-10

218.3125 2.146E-10 5.703E-10 7.351E-10

218.5313 1.210E-10 7.172E-10 7.561E-10

218.7500 4.850E-11 9.049E-10 7.896E-10

218.9688 4.783E-11 9.915E-10 7.769E-10

219.1875 1.174E-10 8.864E-10 7.322E-10

219.4063 2.145E-10 6.528E-10 7.017E-10

219.6250 2.981E-10 4.445E-10 6.839E-10

219.8438 3.482E-10 3.512E-10 6.326E-10

220.0625 3.569E-10 3.443E-10 5.461E-10

220.2813 3.239E-10 3.633E-10 5.139E-10

220.5000 2.636E-10 3.984E-10 6.407E-10

220.7188 2.069E-10 4.662E-10 9.267E-10

220.9375 1.953E-10 5.437E-10 1.240E-09

221.1563 2.663E-10 5.736E-10 1.410E-09

221.3750 4.170E-10 5.384E-10 1.360E-09

221.5938 5.723E-10 4.976E-10 1.142E-09

221.8125 6.231E-10 5.299E-10 8.662E-10

222.0313 5.280E-10 6.404E-10 6.022E-10

222.2500 3.590E-10 7.514E-10 3.758E-10

222.4688 2.236E-10 8.017E-10 2.345E-10

222.6875 1.573E-10 8.279E-10 2.636E-10

222.9063 1.174E-10 9.122E-10 5.050E-10

223.1250 6.126E-11 1.055E-09 8.777E-10

223.3438 -4.300E-12 1.146E-09 1.207E-09

223.5625 -4.369E-11 1.076E-09 1.344E-09

223.7813 -2.719E-11 8.613E-10 1.263E-09

224.0000 5.252E-11 6.267E-10 1.046E-09

224.2188 1.636E-10 5.129E-10 7.899E-10

224.4375 2.340E-10 5.922E-10 5.423E-10

224.6563 2.063E-10 8.426E-10 3.151E-10

224.8750 9.830E-11 1.148E-09 1.365E-10

225.0938 -3.433E-11 1.319E-09 3.521E-11

225.3125 -2.086E-10 1.169E-09 -4.127E-11

225.5313 -5.179E-10 6.332E-10 -2.322E-10

225.7500 -9.672E-10 -1.345E-10 -6.046E-10

225.9688 -1.353E-09 -8.157E-10 -9.904E-10

226.1875 -1.404E-09 -1.117E-09 -1.077E-09

226.4063 -1.055E-09 -9.549E-10 -7.206E-10

226.6250 -5.211E-10 -4.688E-10 -1.087E-10

226.8438 -7.986E-11 1.103E-10 4.505E-10

227.0625 1.846E-10 6.084E-10 8.641E-10

227.2813 4.036E-10 9.791E-10 1.285E-09

227.5000 7.419E-10 1.291E-09 1.817E-09

227.7188 1.224E-09 1.631E-09 2.298E-09

227.9375 1.709E-09 1.967E-09 2.446E-09

228.1563 2.000E-09 2.128E-09 2.170E-09

228.3750 1.984E-09 1.957E-09 1.675E-09

228.5938 1.690E-09 1.504E-09 1.255E-09

228.8125 1.248E-09 9.991E-10 1.047E-09

229.0313 7.924E-10 6.624E-10 9.902E-10

229.2500 4.063E-10 5.623E-10 9.666E-10

229.4688 1.226E-10 6.320E-10 9.171E-10

229.6875 -6.324E-11 7.541E-10 8.514E-10

229.9063 -1.816E-10 8.198E-10 7.889E-10

230.1250 -2.609E-10 7.738E-10 7.178E-10

230.3438 -2.961E-10 6.532E-10 6.155E-10

230.5625 -2.576E-10 5.644E-10 5.045E-10

230.7813 -1.431E-10 5.906E-10 4.555E-10

231.0000 -7.898E-12 7.142E-10 5.093E-10

231.2188 7.498E-11 8.383E-10 6.150E-10

231.4375 8.618E-11 8.806E-10 6.828E-10

231.6563 7.720E-11 8.419E-10 6.832E-10

231.8750 1.122E-10 7.850E-10 6.555E-10

232.0938 2.033E-10 7.607E-10 6.342E-10

232.3125 3.080E-10 7.652E-10 6.161E-10

232.5313 3.783E-10 7.655E-10 6.047E-10

232.7500 3.968E-10 7.432E-10 6.381E-10

232.9688 3.722E-10 7.043E-10 7.442E-10

233.1875 3.225E-10 6.590E-10 8.812E-10

233.4063 2.722E-10 6.118E-10 9.455E-10

233.6250 2.505E-10 5.711E-10 8.472E-10

233.8438 2.730E-10 5.566E-10 5.944E-10

234.0625 3.236E-10 5.926E-10 3.089E-10

234.2813 3.673E-10 6.862E-10 1.482E-10

234.5000 3.832E-10 7.996E-10 1.938E-10

234.7188 3.797E-10 8.489E-10 4.003E-10

234.9375 3.752E-10 7.569E-10 6.363E-10

235.1563 3.757E-10 5.276E-10 7.694E-10

235.3750 3.753E-10 2.563E-10 7.418E-10

235.5938 3.711E-10 5.669E-11 6.092E-10

235.8125 3.676E-10 -1.027E-11 5.096E-10

236.0313 3.652E-10 6.041E-11 5.484E-10

236.2500 3.504E-10 2.434E-10 6.972E-10

236.4688 3.065E-10 4.820E-10 8.239E-10

236.6875 2.391E-10 6.890E-10 8.350E-10

236.9063 1.840E-10 7.983E-10 7.665E-10

237.1250 1.796E-10 8.229E-10 7.269E-10

237.3438 2.288E-10 8.382E-10 7.751E-10

237.5625 2.948E-10 9.058E-10 8.793E-10

237.7813 3.362E-10 1.027E-09 9.717E-10

238.0000 3.428E-10 1.158E-09 1.014E-09

238.2188 3.335E-10 1.234E-09 1.033E-09

238.4375 3.273E-10 1.184E-09 1.163E-09

238.6563 3.351E-10 9.527E-10 1.640E-09

238.8750 3.945E-10 5.710E-10 2.571E-09

239.0938 5.956E-10 1.835E-10 3.580E-09

239.3125 1.010E-09 -5.498E-12 3.820E-09

239.5313 1.545E-09 1.318E-10 2.663E-09

239.7500 1.909E-09 5.236E-10 4.732E-10

239.9688 1.819E-09 9.111E-10 -1.513E-09

240.1875 1.281E-09 1.033E-09 -2.222E-09

240.4063 6.074E-10 8.399E-10 -1.601E-09

240.6250 1.364E-10 5.161E-10 -4.218E-10

240.8438 -2.092E-11 2.881E-10 5.580E-10

241.0625 3.763E-11 2.289E-10 1.079E-09

241.2813 1.915E-10 2.618E-10 1.224E-09

241.5000 3.932E-10 3.016E-10 1.104E-09

241.7188 6.154E-10 3.429E-10 7.978E-10

241.9375 7.919E-10 4.204E-10 4.106E-10

242.1563 8.512E-10 5.356E-10 7.517E-11

242.3750 7.863E-10 6.424E-10 -1.121E-10

242.5938 6.587E-10 6.873E-10 -1.205E-10

242.8125 5.316E-10 6.504E-10 2.146E-11

243.0313 4.224E-10 5.520E-10 2.397E-10

243.2500 3.283E-10 4.309E-10 4.452E-10

243.4688 2.760E-10 3.214E-10 5.804E-10

243.6875 3.178E-10 2.445E-10 6.416E-10

243.9063 4.724E-10 2.133E-10 6.581E-10

244.1250 6.823E-10 2.401E-10 6.531E-10

244.3438 8.387E-10 3.322E-10 6.250E-10

244.5625 8.596E-10 4.695E-10 5.666E-10

244.7813 7.481E-10 5.910E-10 4.953E-10

245.0000 5.774E-10 6.285E-10 4.477E-10

245.2188 4.231E-10 5.792E-10 4.379E-10

245.4375 3.136E-10 5.254E-10 4.351E-10

245.6563 2.394E-10 5.513E-10 4.029E-10

245.8750 1.899E-10 6.433E-10 3.474E-10

246.0938 1.735E-10 7.060E-10 3.039E-10

246.3125 2.026E-10 6.748E-10 2.826E-10

246.5313 2.665E-10 5.862E-10 2.577E-10

246.7500 3.234E-10 5.357E-10 2.176E-10

246.9688 3.267E-10 5.944E-10 1.992E-10

247.1875 2.608E-10 7.613E-10 2.456E-10

247.4063 1.486E-10 9.684E-10 3.437E-10

247.6250 3.217E-11 1.127E-09 4.308E-10

247.8438 -4.252E-11 1.196E-09 4.611E-10

248.0625 -3.184E-11 1.207E-09 4.401E-10

248.2813 8.648E-11 1.214E-09 3.914E-10

248.5000 2.828E-10 1.216E-09 3.161E-10

248.7188 4.698E-10 1.159E-09 2.005E-10

248.9375 5.470E-10 1.021E-09 4.861E-11

249.1563 4.732E-10 8.649E-10 -1.101E-10

249.3750 3.106E-10 7.975E-10 -2.402E-10

249.5938 1.938E-10 8.566E-10 -3.174E-10

249.8125 2.276E-10 9.668E-10 -3.265E-10

250.0313 3.965E-10 1.015E-09 -2.555E-10

250.2500 5.740E-10 9.663E-10 -1.076E-10

250.4688 6.340E-10 8.825E-10 8.195E-11

250.6875 5.536E-10 8.277E-10 2.595E-10

250.9063 4.091E-10 7.853E-10 3.931E-10

251.1250 2.869E-10 6.958E-10 4.864E-10

251.3438 2.168E-10 5.530E-10 5.475E-10

251.5625 1.795E-10 4.283E-10 5.631E-10

251.7813 1.467E-10 3.887E-10 5.205E-10

252.0000 1.016E-10 4.155E-10 4.399E-10

252.2188 3.926E-11 4.164E-10 3.699E-10

252.4375 -3.219E-11 3.162E-10 3.543E-10

252.6563 -9.145E-11 1.372E-10 4.022E-10

252.8750 -1.143E-10 -1.126E-11 4.780E-10

253.0938 -8.665E-11 -2.658E-11 5.208E-10

253.3125 -9.171E-12 9.633E-11 4.938E-10

253.5313 1.025E-10 2.678E-10 4.155E-10

253.7500 2.144E-10 3.866E-10 3.305E-10

253.9688 2.790E-10 4.081E-10 2.562E-10

254.1875 2.605E-10 3.440E-10 1.823E-10

254.4063 1.673E-10 2.390E-10 1.179E-10

254.6250 5.547E-11 1.607E-10 1.020E-10

254.8438 -1.181E-11 1.782E-10 1.549E-10

255.0625 -1.237E-11 3.103E-10 2.440E-10

255.2813 2.765E-11 5.020E-10 3.217E-10

255.5000 7.271E-11 6.722E-10 3.813E-10

255.7188 1.150E-10 7.813E-10 4.514E-10

255.9375 1.686E-10 8.367E-10 5.383E-10

256.1563 2.338E-10 8.484E-10 5.996E-10

256.3750 2.824E-10 8.152E-10 5.789E-10

256.5938 2.860E-10 7.541E-10 4.596E-10

256.8125 2.551E-10 7.080E-10 2.827E-10

257.0313 2.361E-10 7.089E-10 1.228E-10

257.2500 2.632E-10 7.561E-10 4.811E-11

257.4688 3.209E-10 8.400E-10 8.395E-11

257.6875 3.661E-10 9.551E-10 1.921E-10

257.9063 3.813E-10 1.068E-09 2.871E-10

258.1250 3.871E-10 1.101E-09 2.999E-10

258.3438 4.028E-10 1.003E-09 2.422E-10

258.5625 4.162E-10 8.208E-10 2.017E-10

258.7813 4.016E-10 6.887E-10 2.573E-10

259.0000 3.501E-10 7.031E-10 3.999E-10

259.2188 2.668E-10 8.273E-10 5.404E-10

259.4375 1.575E-10 9.272E-10 5.936E-10

259.6563 4.186E-11 8.980E-10 5.506E-10

259.8750 -3.489E-11 7.437E-10 4.697E-10

260.0938 -3.329E-11 5.393E-10 4.088E-10

260.3125 3.923E-11 3.518E-10 3.811E-10

260.5313 1.396E-10 2.177E-10 3.761E-10

260.7500 2.330E-10 1.681E-10 3.946E-10

260.9688 3.011E-10 2.223E-10 4.347E-10

261.1875 3.196E-10 3.477E-10 4.594E-10

261.4063 2.714E-10 4.600E-10 4.240E-10

261.6250 1.872E-10 4.907E-10 3.373E-10

261.8438 1.320E-10 4.500E-10 2.600E-10

262.0625 1.343E-10 4.086E-10 2.370E-10

262.2813 1.546E-10 4.206E-10 2.680E-10

262.5000 1.394E-10 4.627E-10 3.429E-10

262.7188 7.754E-11 4.444E-10 4.443E-10

262.9375 -4.898E-12 2.847E-10 4.942E-10

263.1563 -6.642E-11 5.860E-12 3.652E-10

263.3750 -3.967E-11 -2.403E-10 8.935E-12

263.5938 1.562E-10 -2.671E-10 -4.537E-10

263.8125 5.237E-10 -1.465E-11 -8.240E-10

264.0313 9.048E-10 3.772E-10 -9.947E-10

264.2500 1.059E-09 6.719E-10 -9.884E-10

264.4688 8.764E-10 7.218E-10 -8.619E-10

264.6875 4.916E-10 5.564E-10 -6.460E-10

264.9063 1.625E-10 3.169E-10 -3.849E-10

265.1250 3.251E-11 1.399E-10 -1.665E-10

265.3438 3.817E-11 9.250E-11 -7.119E-11

265.5625 2.582E-11 1.663E-10 -1.023E-10

265.7813 -8.046E-11 3.020E-10 -1.806E-10

266.0000 -2.205E-10 4.434E-10 -2.034E-10

266.2188 -2.644E-10 5.839E-10 -1.186E-10

266.4375 -1.245E-10 7.441E-10 4.535E-11

266.6563 1.693E-10 9.007E-10 2.202E-10

266.8750 4.825E-10 9.692E-10 3.665E-10

267.0938 6.704E-10 8.764E-10 4.835E-10

267.3125 6.816E-10 6.355E-10 5.598E-10

267.5313 5.803E-10 3.458E-10 5.590E-10

267.7500 4.768E-10 1.317E-10 4.781E-10

267.9688 4.355E-10 7.953E-11 3.871E-10

268.1875 4.377E-10 1.956E-10 3.783E-10

268.4063 4.192E-10 4.056E-10 4.915E-10

268.6250 3.400E-10 5.997E-10 7.069E-10

268.8438 2.181E-10 6.992E-10 9.833E-10

269.0625 1.006E-10 6.935E-10 1.266E-09

269.2813 1.616E-11 6.309E-10 1.468E-09

269.5000 -3.328E-11 5.770E-10 1.494E-09

269.7188 -4.226E-11 5.688E-10 1.305E-09

269.9375 1.094E-11 5.904E-10 9.606E-10

270.1563 1.353E-10 5.852E-10 5.820E-10

270.3750 2.902E-10 4.956E-10 2.628E-10

270.5938 3.869E-10 3.046E-10 1.684E-11

270.8125 3.435E-10 5.401E-11 -1.906E-10

271.0313 1.623E-10 -1.726E-10 -3.642E-10

271.2500 -4.611E-11 -3.001E-10 -4.465E-10

271.4688 -1.430E-10 -3.138E-10 -3.741E-10

271.6875 -8.463E-11 -2.697E-10 -1.529E-10

271.9063 4.823E-11 -2.424E-10 1.334E-10

272.1250 1.472E-10 -2.538E-10 3.770E-10

272.3438 1.754E-10 -2.514E-10 5.045E-10

272.5625 1.683E-10 -1.608E-10 5.221E-10

272.7813 1.828E-10 4.967E-11 5.330E-10

273.0000 2.692E-10 3.646E-10 6.717E-10

273.2188 4.524E-10 7.523E-10 9.653E-10

273.4375 6.927E-10 1.156E-09 1.271E-09

273.6563 8.865E-10 1.463E-09 1.398E-09

273.8750 9.407E-10 1.560E-09 1.286E-09

274.0938 8.430E-10 1.442E-09 1.028E-09

274.3125 6.470E-10 1.248E-09 7.470E-10

274.5313 4.188E-10 1.133E-09 4.949E-10

274.7500 2.242E-10 1.125E-09 2.784E-10

274.9688 1.337E-10 1.140E-09 1.038E-10

275.1875 1.808E-10 1.092E-09 -3.491E-11

275.4063 3.091E-10 9.561E-10 -1.662E-10

275.6250 3.959E-10 7.711E-10 -2.910E-10

275.8438 3.491E-10 6.113E-10 -3.487E-10

276.0625 1.759E-10 5.441E-10 -2.808E-10

276.2813 -3.916E-11 5.685E-10 -1.289E-10

276.5000 -2.069E-10 6.055E-10 -2.658E-11

276.7188 -2.840E-10 5.801E-10 -6.693E-11

276.9375 -2.758E-10 5.048E-10 -1.865E-10

277.1563 -2.154E-10 4.539E-10 -2.020E-10

277.3750 -1.391E-10 4.646E-10 1.452E-11

277.5938 -6.648E-11 4.949E-10 3.939E-10

277.8125 5.846E-12 4.852E-10 7.176E-10

278.0313 9.019E-11 4.395E-10 8.202E-10

278.2500 1.898E-10 4.299E-10 7.278E-10

278.4688 2.987E-10 5.187E-10 5.905E-10

278.6875 4.091E-10 6.827E-10 5.169E-10

278.9063 5.043E-10 8.286E-10 5.139E-10

279.1250 5.461E-10 8.820E-10 5.477E-10

279.3438 4.937E-10 8.472E-10 5.910E-10

279.5625 3.482E-10 7.776E-10 6.144E-10

279.7813 1.723E-10 7.107E-10 6.039E-10

280.0000 4.577E-11 6.434E-10 5.994E-10

280.2188 -3.833E-12 5.539E-10 6.516E-10

280.4375 -1.729E-11 4.322E-10 7.262E-10

280.6563 -4.923E-11 3.032E-10 7.168E-10

280.8750 -1.078E-10 2.216E-10 5.855E-10

281.0938 -1.559E-10 2.296E-10 4.272E-10

281.3125 -1.634E-10 3.156E-10 3.480E-10

281.5313 -1.439E-10 4.258E-10 3.278E-10

281.7500 -1.385E-10 5.173E-10 2.626E-10

281.9688 -1.659E-10 5.845E-10 1.122E-10

282.1875 -1.917E-10 6.387E-10 -5.658E-11

282.4063 -1.551E-10 6.829E-10 -1.611E-10

282.6250 -3.247E-11 7.118E-10 -1.890E-10

282.8438 1.275E-10 7.207E-10 -1.893E-10

283.0625 2.433E-10 6.989E-10 -2.096E-10

283.2813 2.721E-10 6.278E-10 -2.483E-10

283.5000 2.406E-10 5.015E-10 -2.532E-10

283.7188 2.149E-10 3.578E-10 -1.744E-10

283.9375 2.476E-10 2.704E-10 -2.126E-11

284.1563 3.400E-10 2.966E-10 1.447E-10

284.3750 4.391E-10 4.280E-10 2.771E-10

284.5938 4.748E-10 5.977E-10 3.789E-10

284.8125 4.176E-10 7.340E-10 4.626E-10

285.0313 3.022E-10 8.091E-10 5.112E-10

285.2500 1.914E-10 8.409E-10 5.059E-10

285.4688 1.203E-10 8.526E-10 4.673E-10

285.6875 7.914E-11 8.317E-10 4.384E-10

285.9063 4.610E-11 7.368E-10 4.381E-10

286.1250 2.372E-11 5.536E-10 4.657E-10

286.3438 3.462E-11 3.383E-10 5.436E-10

286.5625 8.470E-11 1.856E-10 7.109E-10

286.7813 1.434E-10 1.511E-10 9.546E-10

287.0000 1.692E-10 2.081E-10 1.173E-09

287.2188 1.510E-10 2.834E-10 1.244E-09

287.4375 1.203E-10 3.319E-10 1.135E-09

287.6563 1.188E-10 3.723E-10 9.278E-10

287.8750 1.533E-10 4.429E-10 7.385E-10

288.0938 1.840E-10 5.280E-10 6.198E-10

288.3125 1.684E-10 5.520E-10 5.568E-10

288.5313 1.200E-10 4.641E-10 5.303E-10

288.7500 1.039E-10 3.103E-10 5.498E-10

288.9688 1.609E-10 1.986E-10 6.180E-10

289.1875 2.495E-10 1.953E-10 6.883E-10

289.4063 2.895E-10 2.766E-10 6.870E-10

289.6250 2.573E-10 3.756E-10 5.757E-10

289.8438 2.101E-10 4.505E-10 3.792E-10

290.0625 2.120E-10 4.995E-10 1.659E-10

290.2813 2.630E-10 5.302E-10 1.627E-11

290.5000 3.111E-10 5.344E-10 -8.023E-12

290.7188 3.133E-10 4.961E-10 9.906E-11

290.9375 2.662E-10 4.101E-10 2.775E-10

291.1563 1.879E-10 2.861E-10 4.495E-10

291.3750 8.953E-11 1.369E-10 5.794E-10

291.5938 -3.123E-11 -1.693E-11 6.714E-10

291.8125 -1.725E-10 -1.299E-10 7.175E-10

292.0313 -3.094E-10 -1.443E-10 6.835E-10

292.2500 -3.971E-10 -4.768E-11 5.561E-10

292.4688 -3.954E-10 9.527E-11 3.848E-10

292.6875 -2.961E-10 1.952E-10 2.591E-10

292.9063 -1.302E-10 2.215E-10 2.412E-10

293.1250 5.071E-11 2.089E-10 3.153E-10

293.3438 2.023E-10 1.878E-10 4.007E-10

293.5625 3.071E-10 1.511E-10 4.259E-10

293.7813 3.742E-10 1.036E-10 3.993E-10

294.0000 4.210E-10 9.904E-11 3.884E-10

294.2188 4.527E-10 1.852E-10 4.277E-10

294.4375 4.604E-10 3.276E-10 4.748E-10

294.6563 4.418E-10 4.359E-10 4.720E-10

294.8750 4.108E-10 4.612E-10 4.120E-10

295.0938 3.777E-10 4.317E-10 3.275E-10

295.3125 3.274E-10 3.952E-10 2.550E-10

295.5313 2.367E-10 3.695E-10 2.246E-10

295.7500 1.142E-10 3.543E-10 2.541E-10

295.9688 8.327E-12 3.624E-10 3.439E-10

296.1875 -3.515E-11 4.199E-10 4.828E-10

296.4063 -1.735E-11 5.338E-10 6.523E-10

296.6250 1.551E-11 6.587E-10 8.415E-10

296.8438 4.485E-12 7.176E-10 1.011E-09

297.0625 -8.075E-11 6.656E-10 1.005E-09

297.2813 -1.957E-10 5.232E-10 7.725E-10

297.5000 -2.508E-10 3.643E-10 6.713E-10

297.7188 -2.394E-10 2.721E-10 6.437E-10

297.9375 -2.242E-10 2.423E-10 5.243E-10

298.1563 -1.877E-10 2.032E-10 8.468E-09

298.3750 -1.971E-10 2.384E-10 5.372E-08

298.5938 -3.049E-10 4.043E-10 1.764E-07

298.8125 1.347E-09 -6.519E-13 3.750E-07

299.0313 1.029E-08 -1.754E-09 5.560E-07

299.2500 3.130E-08 -2.559E-09 5.685E-07

299.4688 5.845E-08 4.008E-09 3.325E-07

299.6875 7.297E-08 2.219E-08 -5.945E-08

299.9063 5.881E-08 4.557E-08 -3.957E-07

300.1250 2.150E-08 5.920E-08 -5.144E-07

300.3438 -1.436E-08 5.342E-08 -4.197E-07

300.5625 -2.857E-08 3.359E-08 -2.386E-07

300.7813 -2.204E-08 1.377E-08 -9.289E-08

301.0000 -9.485E-09 2.973E-09 -2.211E-08

301.2188 -1.912E-09 2.268E-10 -2.269E-09

301.4375 -3.868E-11 4.399E-10 -8.585E-11

301.6563 -1.954E-10 6.484E-10 -4.134E-11

301.8750 -3.216E-10 5.664E-10 1.673E-10

302.0938 -3.123E-10 4.837E-10 1.364E-10

302.3125 -3.200E-10 4.063E-10 8.777E-11

302.5313 -2.907E-10 3.169E-10 2.096E-10

302.7500 -2.132E-10 2.663E-10 4.008E-10

302.9688 -1.343E-10 2.635E-10 5.468E-10

303.1875 -8.300E-11 2.835E-10 5.914E-10

303.4063 -7.236E-11 3.119E-10 5.135E-10

303.6250 -9.297E-11 3.412E-10 3.534E-10

303.8438 -1.075E-10 3.690E-10 2.087E-10

304.0625 -7.724E-11 3.939E-10 1.877E-10

304.2813 1.613E-11 3.946E-10 3.463E-10

304.5000 1.589E-10 3.352E-10 6.231E-10

304.7188 3.000E-10 2.070E-10 8.459E-10

304.9375 3.815E-10 5.453E-11 8.613E-10

305.1563 3.846E-10 -4.976E-11 6.811E-10

305.3750 3.289E-10 -6.164E-11 4.649E-10

305.5938 2.376E-10 7.668E-12 3.447E-10

305.8125 1.202E-10 1.085E-10 3.042E-10

306.0313 -1.598E-11 1.848E-10 2.472E-10

306.2500 -1.544E-10 2.028E-10 1.356E-10

306.4688 -2.683E-10 1.771E-10 1.179E-11

306.6875 -3.263E-10 1.640E-10 -8.623E-11

306.9063 -3.088E-10 2.015E-10 -1.657E-10

307.1250 -2.235E-10 2.562E-10 -2.288E-10

307.3438 -1.023E-10 2.605E-10 -2.355E-10

307.5625 1.598E-11 2.036E-10 -1.468E-10

307.7813 1.017E-10 1.533E-10 1.821E-11

308.0000 1.465E-10 1.827E-10 1.913E-10

308.2188 1.672E-10 3.010E-10 3.175E-10

308.4375 1.862E-10 4.606E-10 3.871E-10

308.6563 2.021E-10 6.047E-10 4.110E-10

308.8750 1.921E-10 7.066E-10 3.855E-10

309.0938 1.503E-10 7.791E-10 3.005E-10

309.3125 1.078E-10 8.459E-10 1.746E-10

309.5313 1.036E-10 8.898E-10 6.683E-11

309.7500 1.408E-10 8.451E-10 4.629E-11

309.9688 1.795E-10 6.639E-10 1.433E-10

310.1875 1.776E-10 3.899E-10 3.170E-10

310.4063 1.348E-10 1.427E-10 4.660E-10

310.6250 9.035E-11 1.921E-11 4.955E-10

310.8438 8.006E-11 1.825E-11 4.032E-10

311.0625 1.020E-10 6.426E-11 2.818E-10

311.2813 1.188E-10 8.932E-11 2.122E-10

311.5000 8.395E-11 8.656E-11 1.796E-10

311.7188 -3.185E-11 9.460E-11 1.199E-10

311.9375 -2.178E-10 1.413E-10 1.410E-11

312.1563 -4.069E-10 2.112E-10 -8.996E-11

312.3750 -4.952E-10 2.676E-10 -1.348E-10

312.5938 -4.134E-10 2.990E-10 -1.101E-10

312.8125 -1.993E-10 3.339E-10 -5.853E-11

313.0313 2.007E-11 4.036E-10 -3.472E-11

313.2500 1.371E-10 4.928E-10 -5.890E-11

313.4688 1.436E-10 5.475E-10 -1.061E-10

313.6875 9.302E-11 5.438E-10 -1.376E-10

313.9063 1.379E-11 5.228E-10 -1.436E-10

314.1250 -9.919E-11 5.356E-10 -1.504E-10

314.3438 -2.289E-10 5.723E-10 -1.802E-10

314.5625 -3.246E-10 5.779E-10 -2.059E-10

314.7813 -3.453E-10 5.363E-10 -1.667E-10

315.0000 -2.843E-10 4.944E-10 -4.637E-11

315.2188 -1.604E-10 4.869E-10 7.673E-11

315.4375 -1.261E-11 4.832E-10 9.552E-11

315.6563 1.076E-10 4.472E-10 -8.518E-12

315.8750 1.570E-10 4.059E-10 -1.499E-10

316.0938 1.325E-10 4.257E-10 -2.226E-10

316.3125 8.455E-11 5.303E-10 -1.641E-10

316.5313 7.553E-11 6.577E-10 2.498E-11

316.7500 1.029E-10 7.165E-10 2.692E-10

316.9688 8.846E-11 6.820E-10 4.485E-10

317.1875 -3.230E-11 5.992E-10 4.773E-10

317.4063 -2.298E-10 4.941E-10 3.648E-10

317.6250 -4.131E-10 3.504E-10 1.964E-10

317.8438 -5.223E-10 1.805E-10 4.850E-11

318.0625 -5.514E-10 4.990E-11 -6.191E-11

318.2813 -4.949E-10 1.976E-11 -1.285E-10

318.5000 -3.372E-10 9.606E-11 -1.052E-10

318.7188 -1.006E-10 2.352E-10 4.983E-11

318.9375 1.450E-10 3.783E-10 3.099E-10

319.1563 3.295E-10 4.716E-10 5.840E-10

319.3750 4.149E-10 4.764E-10 7.665E-10

319.5938 4.020E-10 3.985E-10 7.997E-10

319.8125 3.271E-10 2.980E-10 7.021E-10

320.0313 2.430E-10 2.378E-10 5.377E-10

320.2500 1.883E-10 2.347E-10 3.763E-10

320.4688 1.594E-10 2.630E-10 2.718E-10

320.6875 1.107E-10 2.843E-10 2.316E-10

320.9063 1.089E-11 2.809E-10 2.088E-10

321.1250 -1.070E-10 2.641E-10 1.369E-10

321.3438 -1.796E-10 2.469E-10 -8.420E-12

321.5625 -1.757E-10 2.381E-10 -1.567E-10

321.7813 -1.176E-10 2.455E-10 -1.963E-10

322.0000 -6.545E-11 2.560E-10 -1.032E-10

322.2188 -5.751E-11 2.591E-10 5.897E-11

322.4375 -6.632E-11 2.733E-10 2.282E-10

322.6563 -4.519E-11 2.932E-10 3.399E-10

322.8750 9.420E-12 2.723E-10 3.340E-10

323.0938 6.757E-11 2.094E-10 2.378E-10

323.3125 9.945E-11 1.719E-10 1.335E-10

323.5313 1.010E-10 2.224E-10 6.662E-11

323.7500 9.133E-11 3.564E-10 5.327E-11

323.9688 6.810E-11 5.076E-10 8.658E-11

324.1875 2.111E-11 6.486E-10 1.370E-10

324.4063 -8.364E-12 8.271E-10 2.016E-10

324.6250 2.485E-11 1.018E-09 2.828E-10

324.8438 1.090E-10 1.111E-09 3.573E-10

325.0625 2.149E-10 1.105E-09 4.106E-10

325.2813 2.983E-10 1.067E-09 3.992E-10

325.5000 2.970E-10 9.731E-10 2.676E-10

325.7188 2.244E-10 8.193E-10 7.509E-11

325.9375 1.653E-10 6.918E-10 -8.715E-11

326.1563 1.721E-10 6.439E-10 -2.184E-10

326.3750 2.441E-10 6.633E-10 -3.078E-10

326.5938 3.287E-10 6.842E-10 -3.496E-10

326.8125 3.585E-10 6.368E-10 -3.874E-10

327.0313 3.514E-10 5.713E-10 -3.561E-10

327.2500 3.274E-10 5.239E-10 -2.165E-10

327.4688 2.442E-10 4.002E-10 -1.342E-10

327.6875 1.831E-10 2.444E-10 -1.372E-10

327.9063 2.592E-10 1.897E-10 -8.547E-11

328.1250 3.484E-10 1.531E-10 -3.123E-11

328.3438 3.076E-10 8.279E-11 4.235E-13

328.5625 1.866E-10 7.070E-11 1.169E-10

328.7813 5.778E-11 7.788E-11 2.148E-10

329.0000 4.024E-11 1.602E-10 2.248E-10

329.2188 2.190E-10 4.462E-10 2.174E-10

329.4375 3.723E-10 6.776E-10 5.939E-11

329.6563 3.225E-10 6.939E-10 -1.342E-10

329.8750 1.642E-10 7.165E-10 -4.561E-11

330.0938 -1.101E-10 6.747E-10 1.599E-11

330.3125 -3.882E-10 5.251E-10 -1.294E-10

330.5313 -3.737E-10 5.058E-10 -9.946E-11

330.7500 -2.534E-10 3.636E-10 -1.269E-10

330.9688 -2.084E-10 1.082E-10 -2.983E-10

331.1875 4.055E-11 3.290E-10 -4.208E-11

331.4063 2.605E-10 5.084E-10 1.461E-11

331.6250 1.824E-10 1.381E-10 -5.288E-10

331.8438 1.954E-10 4.231E-11 -3.759E-10

332.0625 -2.827E-11 -9.189E-11 5.205E-11

332.2813 -6.774E-10 -6.951E-10 -3.484E-10

332.5000 -6.139E-10 -3.795E-10 -2.621E-10

332.7188 -1.727E-10 -1.771E-12 -1.814E-10

332.9375 -3.887E-10 -9.434E-10 -1.268E-09

333.1563 -1.916E-10 -5.971E-10 -8.748E-10

333.3750 -1.645E-10 5.036E-10 -3.665E-11

333.5938 -1.447E-09 -7.060E-10 -1.748E-09

333.8125 -1.087E-09 -7.283E-10 -1.395E-09

334.0313 1.026E-10 4.534E-10 7.960E-11

334.2500 -1.694E-09 -1.684E-09 -3.365E-09

334.4688 -1.733E-09 -1.305E-09 -3.223E-09

334.6875 1.486E-10 1.966E-11 9.713E-11

334.9063 -3.367E-09 -7.329E-09 -6.915E-09

335.1250 -3.815E-09 -7.576E-09 -8.046E-09

335.3438 -3.400E-10 -2.968E-10 -1.166E-10

335.5625 -9.814E-09 -1.432E-08 -1.390E-08

335.7813 -1.251E-08 -1.914E-08 -1.761E-08

336.0000 -2.012E-10 -2.856E-10 -1.470E-10

336.2188 -1.935E-08 -2.980E-08 -4.232E-08

336.4375 -3.029E-08 -4.745E-08 -6.338E-08

336.6563 -6.582E-11 -1.692E-10 -7.987E-10

336.8750 -6.646E-08 -9.856E-08 -1.121E-07

337.0938 -1.220E-07 -1.812E-07 -2.106E-07

337.3125 -2.766E-09 -2.646E-09 -2.870E-09

337.5313 -2.612E-07 -4.147E-07 -4.912E-07

337.7500 -6.244E-07 -9.812E-07 -1.161E-06

337.9688 -2.910E-08 -4.806E-08 -5.737E-08

338.1875 -1.817E-06 -2.783E-06 -3.276E-06

338.4063 -6.391E-06 -9.831E-06 -1.158E-05

338.6250 -7.828E-07 -1.196E-06 -1.408E-06

338.8438 -4.125E-05 -6.379E-05 -7.526E-05

339.0625 -4.427E-04 -6.840E-04 -8.068E-04

339.2813 -1.763E-03 -2.723E-03 -3.211E-03

339.5000 -4.252E-03 -6.568E-03 -7.747E-03

339.7188 -7.175E-03 -1.108E-02 -1.307E-02

339.9375 -8.963E-03 -1.384E-02 -1.633E-02

340.1563 -8.455E-03 -1.306E-02 -1.540E-02

340.3750 -5.993E-03 -9.257E-03 -1.092E-02

340.5938 -3.086E-03 -4.767E-03 -5.623E-03

340.8125 -1.062E-03 -1.641E-03 -1.936E-03

341.0313 -1.947E-04 -3.010E-04 -3.553E-04

341.2500 -6.050E-06 -9.383E-06 -1.113E-05

341.4688 -4.604E-06 -7.077E-06 -8.297E-06

341.6875 -4.941E-06 -7.600E-06 -8.925E-06

341.9063 -4.091E-07 -6.248E-07 -7.314E-07

342.1250 -3.357E-07 -5.227E-07 -6.162E-07

342.3438 -5.856E-07 -9.076E-07 -1.063E-06

342.5625 -7.595E-08 -1.177E-07 -1.350E-07

342.7813 -5.074E-08 -7.759E-08 -9.567E-08

343.0000 -1.218E-07 -1.869E-07 -2.256E-07

343.2188 -2.035E-08 -3.054E-08 -3.766E-08

343.4375 -1.237E-08 -1.866E-08 -2.307E-08

343.6563 -3.738E-08 -5.739E-08 -7.019E-08

343.8750 -8.178E-09 -1.205E-08 -1.602E-08

344.0938 -3.262E-09 -4.176E-09 -4.811E-09

344.3125 -1.317E-08 -1.935E-08 -2.258E-08

344.5313 -3.341E-09 -4.556E-09 -5.993E-09

344.7500 -7.906E-10 -1.585E-09 -1.910E-09

344.9688 -5.157E-09 -9.125E-09 -9.545E-09

345.1875 -1.372E-09 -3.116E-09 -2.821E-09

345.4063 -2.273E-10 -4.803E-10 -1.439E-09

345.6250 -2.656E-09 -3.580E-09 -6.092E-09

345.8438 -9.897E-10 -1.055E-09 -2.661E-09

346.0625 8.204E-11 1.305E-10 -3.132E-10

346.2813 -9.141E-10 -1.693E-09 -1.917E-09

346.5000 -3.372E-10 -5.655E-10 -5.962E-10

346.7188 -1.905E-10 1.537E-10 -2.172E-11

346.9375 -9.979E-10 -9.178E-10 -1.429E-09

347.1563 -4.795E-10 -4.354E-10 -7.458E-10

347.3750 5.409E-11 -2.223E-11 -1.181E-10

347.5938 -2.981E-10 -6.557E-10 -8.973E-10

347.8125 -1.968E-10 -2.758E-10 -5.998E-10

348.0313 -1.454E-11 3.095E-10 -6.865E-11

348.2500 -2.495E-10 1.799E-10 -3.610E-10

348.4688 -1.603E-10 3.954E-10 -2.447E-10

348.6875 5.148E-11 5.875E-10 -1.830E-10

348.9063 4.897E-12 2.852E-10 -7.082E-10

349.1250 7.704E-11 3.258E-10 -7.717E-10

349.3438 1.778E-10 6.246E-10 -5.334E-10

349.5625 1.943E-10 7.502E-10 -6.501E-10

349.7813 4.730E-10 1.010E-09 -6.971E-10

350.0000 8.354E-10 1.192E-09 -6.038E-10

**Data plotted in FIG 4(a)**

Time from introduction of PMB (min) Test resonator. Spectral power [10^(-6)Hz^2] Control resonator. Spectral power [10^(-6)Hz^2] Time from introduction of PMB (min) Coverage area [10^(-3) mm^2]

-41.417 0.138 0.161 -50.480 1.393

-37.950 0.140 0.164 -50.127 1.439

-34.767 0.165 0.200 -49.278 1.420

-29.867 0.186 0.219 -48.275 1.500

-26.717 0.204 0.232 -47.278 1.516

-22.667 0.225 0.224 -46.278 1.518

-19.383 0.236 0.254 -45.278 1.583

-16.033 0.227 0.265 -44.278 1.609

-15.533 0.227 0.265 -43.278 1.688

-11.733 0.263 0.296 -42.278 1.703

-8.433 0.265 0.329 -41.278 1.806

-5.317 0.290 0.365 -40.278 1.844

7.233 -0.004 0.472 -39.278 1.836

10.550 0.000 0.514 -38.278 1.924

13.567 -0.005 0.524 -37.278 1.986

17.350 0.003 0.569 -36.278 1.965

20.883 -0.001 0.627 -35.278 2.028

24.083 0.000 0.667 -34.278 2.158

28.050 0.006 0.681 -33.278 2.188

31.250 0.004 0.774 -32.278 2.255

34.600 0.005 0.854 -31.278 2.244

37.950 0.005 0.861 -30.278 2.390

41.233 0.006 0.874 -29.278 2.399

44.817 -0.000 0.963 -28.278 2.423

48.267 0.001 0.975 -27.278 2.494

51.767 -0.004 1.017 -26.278 2.417

54.967 0.007 1.098 -25.278 2.508

58.167 -0.002 1.150 -24.278 2.447

61.917 0.005 1.167 -23.278 2.589

65.033 -0.003 1.211 -22.278 2.551

-21.278 2.568

-20.278 2.625

-19.278 2.649

-18.278 2.687

-17.278 2.712

-16.278 2.821

-15.275 2.936

-14.278 2.932

-13.278 2.960

-12.278 3.053

-11.278 3.129

-10.278 3.250

-9.278 3.215

-8.278 3.276

-7.278 3.401

-6.278 3.470

-6.205 3.468

-5.858 3.489

-5.003 3.581

4.812 3.792

5.159 3.701

6.013 3.641

7.013 3.579

8.013 3.530

9.013 3.477

10.013 3.597

11.013 3.528

12.013 3.496

13.013 3.430

14.013 3.328

15.013 3.273

16.013 3.201

17.013 3.173

18.013 3.381

19.013 3.344

20.013 3.300

21.013 3.225

22.013 3.195

23.013 3.210

24.013 3.163

25.013 3.138

26.013 3.064

27.013 3.020

28.013 3.015

29.013 2.979

30.013 3.265

31.013 2.992

32.013 3.025

33.013 2.951

34.013 3.286

35.013 3.304

36.020 3.299

37.013 3.236

38.013 3.248

39.013 3.205

40.013 3.122

41.013 3.086

42.013 3.030

43.013 2.998

44.013 3.022

45.013 3.011

46.013 2.985

47.013 3.004

48.013 2.991

49.013 3.000

50.013 2.977

51.013 2.896

52.013 2.967

53.013 2.963

54.013 2.948

55.013 2.960

56.013 2.972

57.013 2.973

58.013 3.042

59.013 2.996

60.013 3.131

61.013 3.123

62.013 3.104

63.013 3.012

64.013 2.985

65.013 2.960

66.013 2.989

67.013 3.002

68.013 2.940

68.749 2.973

69.094 2.859

**FIG 4(b)**

Time from introduction of PMB (min) Test resonator. Spectral power [10^(-6)Hz^2] Control resonator. Spectral power [10^(-6)Hz^2] Time from introduction of PMB (min) Coverage area [10^(-3) mm^2]

-38.400 0.233 0.212 -46.214 1.998

-35.000 0.255 0.251 -45.214 2.034

-31.900 0.301 0.260 -44.214 2.080

-28.433 0.266 0.298 -43.214 2.098

-24.950 0.317 0.277 -42.214 2.137

-21.900 0.354 0.328 -41.214 2.181

-18.483 0.369 0.322 -40.214 2.224

-15.250 0.385 0.371 -39.214 2.268

-12.033 0.414 0.384 -38.214 2.309

-8.750 0.477 0.424 -37.214 2.366

-5.650 0.465 0.486 -36.214 2.407

7.983 -0.009 0.517 -35.214 2.551

11.200 0.006 0.560 -34.214 2.582

14.367 0.012 0.586 -33.214 2.614

17.567 0.015 0.625 -32.214 2.643

20.717 0.009 0.685 -31.214 2.670

24.367 0.015 0.733 -30.214 2.686

27.683 0.001 0.806 -29.214 2.624

31.133 0.001 0.721 -28.214 2.652

34.367 -0.011 0.803 -27.214 2.682

37.583 -0.016 0.827 -26.214 2.695

40.933 -0.017 0.811 -25.214 2.715

44.233 -0.023 0.842 -24.214 2.757

47.417 -0.023 0.773 -23.214 2.778

50.617 -0.011 0.831 -22.214 2.809

53.783 -0.023 0.811 -21.214 2.759

57.050 -0.021 0.778 -20.214 2.788

60.283 -0.025 0.801 -19.214 2.843

63.567 -0.030 0.807 -18.214 2.883

66.750 -0.022 0.839 -17.214 2.926

69.883 -0.028 0.812 -16.214 2.945

73.217 -0.025 0.822 -15.200 3.014

76.400 -0.021 0.836 -14.214 3.153

79.800 -0.027 0.839 -13.211 3.187

83.067 -0.026 0.891 -12.214 3.260

86.717 -0.012 0.901 -11.214 3.296

89.967 -0.009 0.956 -10.214 3.337

93.150 -0.016 0.954 -9.214 3.381

96.350 -0.023 1.059 -8.214 3.426

99.733 -0.025 1.033 -7.214 3.474

103.033 -0.014 1.132 -6.214 3.522

-5.214 3.576

-4.214 3.637

-3.214 3.665

7.114 3.570

8.114 3.511

9.114 3.425

10.114 3.342

11.114 3.419

12.114 3.341

13.114 3.261

14.114 3.187

15.114 3.295

16.114 3.248

17.114 3.201

18.114 3.160

19.114 3.122

20.114 3.091

21.114 3.062

22.114 3.204

23.114 3.186

24.114 3.164

25.114 3.144

26.114 3.129

27.114 3.113

28.114 3.110

29.114 3.089

30.114 3.074

31.114 3.063

32.121 3.059

33.114 3.053

34.114 3.048

35.114 3.034

36.114 3.032

37.114 3.021

38.114 3.024

39.114 3.017

40.114 3.009

41.114 3.005

42.114 2.998

43.114 2.997

44.114 2.993

45.116 2.988

46.114 2.979

47.114 2.986

48.114 2.986

49.114 2.977

50.114 2.973

51.114 2.978

52.114 2.969

53.114 2.967

54.114 2.971

55.114 2.961

56.114 2.963

57.114 2.954

58.114 2.957

59.114 2.951

60.114 2.948

61.114 2.947

62.114 2.945

63.114 2.936

64.114 2.938

65.114 2.938

66.114 2.928

67.125 2.931

68.114 2.922

69.114 2.917

70.114 2.920

71.114 2.916

72.114 2.912

73.114 2.917

74.114 2.908

75.114 2.903

76.114 2.898

77.114 2.900

78.114 2.897

79.114 2.893

80.114 2.881

81.114 2.879

82.114 2.870

83.114 2.867

84.114 2.868

85.114 2.856

86.114 2.855

87.114 2.860

88.114 2.853

89.114 2.846

90.114 2.846

91.114 2.841

92.114 2.836

93.114 2.839

94.114 2.837

95.114 2.833

96.114 2.825

97.114 2.826

98.114 2.819

99.114 2.810

100.114 2.816

101.114 2.813

102.124 2.809

103.114 2.807

104.117 2.802

**Data plotted in FIG 6(a)**

Time from introduction of ampicillin (min) Test resonator. Spectral power [10^(-6)Hz^2] Control resonator. Spectral power [10^(-6)Hz^2] Time from introduction of ampicillin (min) Coverage area [10^(-3) mm^2]

-31.517 0.316 0.337 -24.730 2.525

-22.850 0.367 0.381 -23.229 2.530

-19.783 0.411 0.417 -22.229 2.568

-15.917 0.428 0.422 -21.229 2.614

-12.433 0.531 0.498 -20.229 2.666

-8.700 0.506 0.520 -19.229 2.699

-5.583 0.520 0.579 -18.229 2.746

7.400 -- 0.697 -17.229 2.777

11.150 0.538 0.765 -16.214 2.822

14.283 0.464 0.768 -15.229 2.870

17.317 0.412 0.864 -14.225 2.934

20.850 0.356 0.900 -13.229 2.979

23.950 0.291 0.924 -12.229 3.136

27.267 -- 0.951 -11.229 3.190

30.800 0.173 0.945 -10.229 3.235

33.850 0.135 0.966 -9.229 3.266

38.783 0.126 0.947 -8.229 3.325

43.667 0.112 0.956 -7.229 3.359

46.800 0.110 0.906 -6.229 3.427

50.550 0.121 0.901 -5.229 3.488

54.400 0.108 0.914 -4.229 3.535

57.683 0.118 0.879 4.463 4.134

60.683 0.084 0.872 5.775 4.253

63.967 0.087 0.877 6.775 4.324

67.000 0.085 0.878 7.775 4.378

69.967 0.094 0.882 8.775 4.419

73.200 0.090 0.878 10.241 4.542

77.567 0.086 0.922 11.238 4.643

81.350 0.092 0.923 12.238 4.692

84.367 0.091 0.957 13.238 4.550

87.867 0.091 0.899 14.238 4.584

91.500 0.096 0.901 15.238 4.632

94.783 0.102 0.992 16.238 4.659

100.283 0.089 -- 17.238 4.695

103.550 0.087 1.119 18.238 4.734

106.850 0.095 1.182 19.238 4.511

110.000 0.099 -- 20.238 4.465

117.050 0.104 1.192 21.238 4.209

22.238 4.172

23.238 3.896

24.238 3.845

25.238 3.578

26.238 3.314

27.238 2.982

28.238 2.822

29.238 2.595

30.238 2.409

31.238 2.209

32.238 2.016

33.238 1.934

34.238 1.819

35.238 1.709

36.238 1.605

37.238 1.505

38.238 1.399

39.238 1.307

40.245 1.258

41.238 1.189

42.238 1.158

43.238 1.114

44.238 1.044

45.238 1.006

46.238 0.994

47.238 0.965

48.238 0.951

49.238 0.945

50.238 0.935

51.238 0.934

52.238 0.930

53.238 0.880

54.238 0.859

55.238 0.855

56.238 0.852

57.238 0.819

58.238 0.818

59.238 0.812

60.238 0.802

61.238 0.827

62.238 0.772

63.238 0.813

64.238 0.774

65.238 0.766

66.238 0.759

67.238 0.750

68.238 0.757

69.238 0.717

70.238 0.708

71.238 0.708

72.238 0.713

73.244 0.715

74.238 0.701

75.238 0.702

76.238 0.705

77.238 0.710

78.238 0.729

79.238 0.732

80.238 0.719

81.238 0.709

82.238 0.710

83.238 0.710

84.238 0.684

85.238 0.663

86.238 0.659

87.238 0.660

88.238 0.651

89.238 0.634

90.238 0.628

91.238 0.626

92.238 0.609

93.238 0.621

94.238 0.623

95.238 0.589

96.238 0.612

97.238 0.605

98.238 0.601

99.238 0.594

100.238 0.593

101.238 0.570

102.238 0.562

103.238 0.564

104.238 0.580

105.238 0.556

106.245 0.568

107.238 0.570

108.238 0.567

109.238 0.563

**Data plotted in FIG 6(b)**

Time from introduction of ampicillin (min) Test resonator. Spectral power [10^(-6)Hz^2] Control resonator. Spectral power [10^(-6)Hz^2] Time from introduction of ampicillin (min) Coverage area [10^(-3) mm^2]

-39.117 0.250 0.281 -42.366 2.659

-36.117 0.266 0.320 -41.061 2.621

-32.700 0.317 0.367 -40.061 2.652

-29.333 0.350 0.390 -39.061 2.686

-26.133 0.344 0.404 -38.047 2.734

-22.883 0.374 0.450 -37.061 2.782

-19.250 0.410 0.507 -36.057 2.815

-15.883 0.459 0.562 -35.061 2.851

-12.900 0.523 0.583 -34.061 2.899

-9.900 0.574 0.645 -33.061 2.945

-6.833 0.618 0.700 -32.061 2.995

-3.850 0.636 0.709 -31.061 3.046

6.650 0.868 0.856 -30.061 3.091

9.850 0.850 0.872 -29.061 3.118

12.983 0.899 0.911 -28.061 3.149

16.133 0.815 1.077 -27.061 3.190

19.117 0.792 -- -26.061 3.391

22.150 0.759 1.105 -25.061 3.437

27.067 0.576 1.251 -24.061 3.490

31.117 0.501 1.244 -23.061 3.552

36.700 0.387 1.264 -22.061 3.624

40.683 0.327 1.254 -21.061 3.691

44.017 0.271 -- -20.061 3.751

47.100 0.229 1.277 -19.061 3.809

52.950 0.239 -- -18.061 3.881

56.100 0.228 1.218 -17.061 3.929

59.233 0.197 -- -16.061 3.979

62.333 0.202 -- -15.061 4.027

65.367 0.196 1.223 -14.061 4.066

69.583 0.188 1.195 -13.061 4.128

72.783 0.177 -- -12.061 4.181

75.850 0.198 1.270 -11.061 4.234

79.233 0.201 1.341 -10.061 4.507

82.350 0.187 -- -9.061 4.570

85.550 0.205 1.304 -8.061 4.644

90.800 0.192 -- -7.061 4.716

93.867 0.217 1.485 -6.061 4.793

-5.061 4.846

-4.061 4.920

5.228 5.518

6.227 5.594

7.227 5.640

8.227 5.712

9.227 5.741

10.227 6.094

11.227 6.144

12.227 6.214

13.227 6.242

14.227 6.257

15.241 6.338

16.227 6.365

17.231 6.434

18.227 6.499

19.227 6.566

20.227 6.638

21.227 6.710

22.227 6.802

23.227 6.802

24.227 6.811

25.227 6.840

26.227 6.826

27.227 6.703

28.227 6.665

29.227 6.542

30.227 6.362

31.227 5.964

32.227 5.822

33.227 5.638

34.227 5.494

35.227 4.970

36.227 4.781

37.227 4.562

38.227 4.111

39.227 3.845

40.227 3.658

41.227 3.228

42.227 3.005

43.227 2.796

44.227 2.616

45.227 2.462

46.227 2.222

47.227 2.074

48.227 1.906

49.227 1.740

50.236 1.634

51.227 1.452

52.227 1.418

53.227 1.291

54.227 1.207

55.227 1.205

56.227 1.174

57.227 1.152

58.227 1.114

59.227 1.092

60.227 1.054

61.227 1.020

62.227 0.977

63.227 0.948

64.227 1.001

65.227 0.972

66.227 0.953

67.227 0.941

68.227 0.917

69.227 0.898

70.227 0.878

71.227 0.859

72.227 0.823

73.227 0.794

74.227 0.766

75.227 0.758

76.227 0.840

77.227 0.830

78.227 0.823

79.227 0.814

80.227 0.797

81.240 0.787

82.227 0.792

83.227 0.763

84.227 0.754

85.227 0.749

86.227 0.744

87.227 0.742

88.227 0.718

89.227 0.701

90.227 0.696

91.227 0.696
